# Chemical Consequences of the Mechanical Bond: A Tandem Active Template‐Rearrangement Reaction

**DOI:** 10.1002/anie.201813950

**Published:** 2019-02-14

**Authors:** Florian Modicom, Ellen M. G. Jamieson, Elise Rochette, Stephen M. Goldup

**Affiliations:** ^1^ Chemistry University of Southampton, Highfield Southampton SO17 1BJ UK

**Keywords:** mechanical bonds, rearrangement, rotaxanes, supramolecular chemistry, triazoles

## Abstract

We report the unexpected discovery of a tandem active template CuAAC‐rearrangement process, in which N_2_ is extruded on the way to the 1,2,3‐triazole product to give instead acrylamide rotaxanes. Mechanistic investigations suggest this process is dictated by the mechanical bond, which stabilizes the Cu^I^‐triazolide intermediate of the CuAAC reaction and diverts it down the rearrangement pathway; when no mechanical bond is formed, the CuAAC product is isolated.

The Cu‐mediated alkyne–azide cycloaddition (CuAAC) reaction^1^ is now ubiquitous in the synthesis of non‐natural products for a wide range of applications.^2^ This success is largely due to the availability of the required starting materials, broad substrate scope, high yield and mild conditions of the reaction itself, often cited as the archetypal click reaction.^3^ Furthermore, the 1,2,3‐triazole link formed from simple azides and alkynes is chemically robust, and is thus an excellent structural unit.^4^

The active template (AT) approach to interlocked molecules,^5^ introduced by Leigh and co‐workers, harnesses the ability of endotopic functional groups within a macrocycle to mediate a new covalent bond forming reaction through the ring and thus generate a mechanical bond. The first and best studied AT process is the AT‐CuAAC reaction,^6^ which employs an endotopically ligated Cu^I^ ion and inherits the benefits of the parent CuAAC process to produce complex rotaxanes,^7^ catenanes,^8^ and knots^9^ in excellent yield with broad substrate scope, and has been applied to the synthesis of mechanically interlocked ligands,^10^ pro‐drugs,^11^ catalysts,^12^ hosts,^13^ sensors,^14^ and molecular machines.^15^

However, to date, all AT reactions generate products in which the bond forming reaction used determines the functional group present in the product; all AT‐CuAAC products reported retain the 1,2,3‐triazole link produced in the cycloaddition process. Here we report the unexpected observation and subsequent optimization of a domino AT‐CuAAC‐rearrangement process to produce acrylamide‐derived rotaxanes with up to 100 % selectivity, and mechanistic studies that rationalize the reaction outcome. Not only does this new transformation expand the range of interlocked molecules available using this simple methodology, it also serves to highlight the ability of mechanical bonding to augment chemical reactivity to produce new reaction outcomes.

We set out to synthesize rotaxane **4** under our optimized AT‐CuAAC conditions7c, 16 with readily available small bipyridine macrocycle **1a**,^17^ azide **2a**, and propargylic alkyne **3a** (Scheme 1). However, apart from **4**, a second interlocked product was observed in trace amounts prior to purification. The amount of the interlocked impurity varied between runs and this effect was eventually traced to the presence of adventitious water; when strictly anhydrous conditions were used, **4** was the only observed interlocked product. Conversely, when water was intentionally added, the new product was found in about 1:9 ratio with **4**, allowing us to isolate and characterize it to determine its structure.

**Scheme 1:**
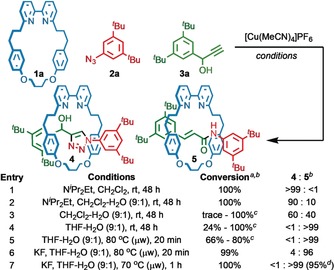
Formation of rotaxanes **4** and **5**. [a] Consumption of **1 a**. [b] Determined by ^1^H NMR analysis of the crude reaction product. [c] Conversion varied considerably run‐to‐run. [d] Yield of isolated product.^18^

LCMS analysis confirmed that the isolated material was a single component with *m*/*z*=926.6199, corresponding to [**4**‐N_2_+H]^+^, suggesting dinitrogen had been extruded. Strikingly, the ^1^H NMR spectrum of the unknown product did not display desymmetrization of the macrocycle component as would be expected if the stereogenic center derived from the propargylic alcohol was present in the axle.16a Also, the ^1^H NMR spectrum of the unknown product contained coupled doublets at 6.85 and 6.29 ppm (*J*=15.5 Hz), consistent with *trans*‐related vinyl protons. Ultimately, slow diffusion of Et_2_O into a CH_2_Cl_2_ solution of the unknown compound produced crystals suitable for single‐crystal X‐ray diffraction analysis (Figure 1), revealing the byproduct to be rotaxane **5**, which is derived from **4** by loss of N_2_ and rearrangement of the axle to yield an acrylamide unit and is consistent with the LCMS and ^1^H NMR analysis.

**Figure 1:**
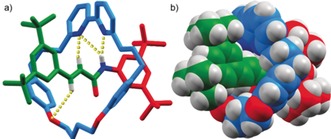
Solid‐state structure of rotaxane **5** in a) stick representation and b) space‐filling representation. Selected intercomponent distances [Å]: H−O 2.94, H−N 2.79, NH−N 2.65, NH−N 2.26.

Having identified **5**, we turned our attention to optimizing its formation. When N^*i*^Pr_2_Et was omitted from the reaction (entry 3), **5** was observed as the major product, albeit with extended reactions times. Replacing CH_2_Cl_2_ with THF yielded a homogenous reaction mixture from which the only interlocked product observed was **5** (entry 4). However, the rate of reaction varied considerably run‐to‐run due to a long induction period, as determined by ^1^H NMR monitoring of the reaction (see the Supporting Information). Heating the reaction mixture to 80 °C (μw)^18^ increased the rate of reaction but the conversion still varied run‐to run (entry 5). This poor reproducibility, and in particular the observed induction period, led us to propose that F^−^, derived from hydrolysis of the counterion of [Cu(MeCN)_4_]PF_6_, played a role in the production of **5**. Pleasingly, addition of KF to the reaction mixture led to a reproducible process (entry 6).^19^ Finally, reducing the reaction temperature to 70 °C allowed complete, rapid and selective formation of **5**, which was isolated in 95 % yield (entry 7).

With optimized conditions in hand, we investigated the effect of substrate structure on reaction selectivity to gain insight into the features required for this unexpected rearrangement process (Figure 2). Macrocycles **1 b** and **1 d** produced acrylamide products **6** and **7** respectively, although more sterically hindered macrocycle **1 d** required more forcing conditions to achieve reasonable conversion, leading to reduced selectivity (7:3 acrylamide‐triazole product). The rearrangement process proved extremely sensitive to the structure of the alkyne or azide component; benzylic azide **2 b** produced rotaxane **8** in diminished selectivity (4:1) and less hindered azide **2 c** produced **9** in poor selectivity (1:3). Similarly, alkyne **3 b**, in which a methylene unit was introduced between the propargylic carbon and the aromatic unit, led to a significant reduction in selectivity (ca. 1:1). Conversely, when hindered tertiary alcohol **3 c** was used, complete selectivity was observed for acrylamide rotaxane **11**.

**Figure 2:**
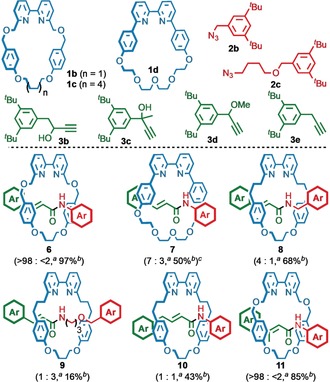
a) Structure of macrocycle, azide, and alkyne substrates explored in the AT‐CuAAC‐rearrangement process. b) Structure of acrylamide rotaxanes isolated under optimized reaction conditions (Scheme 1, entry 7). [a] Ratio of acrylamide to triazole product determined by ^1^H NMR analysis of the crude reaction mixture. [b] Yield of isolated product. [c] Reaction carried out at elevated temperature (150 °C [μW], 2 h) to achieve about 80 % conversion of **1 d**. Ar=3,5‐di‐^*t*^Bu‐C_6_H_3_.

To probe the role of the alcohol functional group we examined alkynes in which this functional group is absent. When methyl ether **3 d** was used in place of alcohol **3 a** the same rearranged rotaxane **5** was observed in excellent selectivity. However, when alkyne **3 e** was used, in which no propargylic C−O bond is present, only the corresponding triazole rotaxane was observed. Finally, when the reaction was carried out either in the absence of macrocycle **1 a** or in the presence of macrocycle **1 c**, which is too large to be retained by the aromatic stopper units, no acrylamide product was observed (see the Supporting Information).^20^

Based on the above results, steric hindrance appears to favor the acrylamide product, the presence of an alcohol or ether unit at the propargylic position of the alkyne is required, and mechanical bond formation is essential. With this information in hand we turned our attention to the mechanism of the rearrangement process. We have previously shown that the AT‐CuAAC reaction proceeds via a Cu^I^‐triazolide intermediate^21^ whose Cu−C bond is kinetically stabilized against protonation by the mechanical bond.16b To probe whether this species was also an intermediate on the way to the acrylamide product we synthesized triazolide **12** by reaction of macrocycle **1 a** with azide **2 a** and alkyne **3 c** in the presence of N^*i*^Pr_2_Et.^22^ When **12** was subjected to optimized conditions for the production of **5**, incomplete conversion to rearranged product **11** was observed. Upon re‐examinining the proposed scheme for the formation of the acrylamide product via the corresponding triazolide, we identified that the latter is formed alongside an equivalent of H^+^ (Scheme 2 b). Accordingly, when triazolide **12** was re‐subjected to our reaction conditions in the presence of 1 equiv of HPF_6_, with or without the addition of KF, even at room temperature (Scheme 2 a), acrylamide **11** was formed selectively. Subjecting the corresponding triazole rotaxane to the optimized reaction conditions did not produce **11**, confirming that **11** is not formed by from the simple AT‐CuAAC product.

**Scheme 2:**
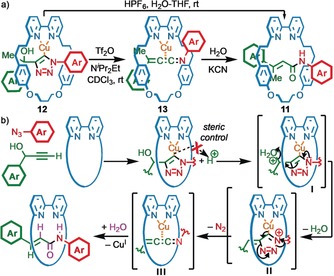
a) Stepwise formation of rotaxane **11** via triazolide **12**. b) Proposed mechanism of the rearrangement process.

These results, combined with previous observations in the CuAAC reaction of azides bearing sulfonyl, phosphoryl, or acyl groups,^23^ allow us to propose a mechanism (Scheme 2 b) for the formation of the acrylamide products. The first step of the reaction is the formation of a triazolide intermediate.^19^ Protonation of the hydroxyl group to generate oxonium **I** and subsequent loss of H_2_O gives resonance stabilized cation **II**. Loss of N_2_ from **II** gives cumulated ketenimine species **III** that can undergo reaction with H_2_O to yield the observed product.23b,23e,23g, 24 This proposed mechanism is consistent with both the need for steric hindrance in the axle component, which kinetically stabilizes the Cu−C bond against proto‐demetallation, and the need for a propargylic hydroxy or ether unit, both of which can act as the leaving group. The proposed mechanism is also supported by preliminary molecular modelling (see the Supporting Information). Protonation of the hydroxy leaving group of a truncated model of the triazolide derived from **1 a**, **2 a**, and **3 a** was predicted to lead directly to loss of H_2_O to give a resonance stabilized carbocation. Subsequent loss of N_2_ to give the proposed Cu‐bound cumulene intermediate **III** was predicted to be exergonic by about 44 kJ mol^−1^ and proceed with a barrier of about 78 kJ mol^−1^.

The proposed mechanism is striking in that the kinetic stabilization of the Cu−C bond provided by the mechanical bond appears to allow a pathway to operate in which an organometallic species is activated by protonation at a thermodynamically less basic position. To further confirm the role of the OH as a leaving group, we monitored the reaction of **1 a**, **2 a**, and **3 c** by ^1^H NMR under anhydrous conditions in CDCl_3_. In the presence of N^*i*^Pr_2_Et (4 equiv), triazolide **12** formed rapidly. Addition of Tf_2_O (1 equiv) led to consumption of **12** to give a major new species consistent with intermediate **13** by ^1^H NMR and MS (*m*/*z*=984.8) analysis (see the Supporting Information).^25^ The species tentatively identified was **13** was surprisingly stable; treatment with H_2_O led to slow conversion to acrylamide **11**. If instead KCN_(aq)_ was added, **13** was rapidly consumed to produce rotaxane **11** in excellent selectivity, suggesting that the Cu^I^ ion held in place by a mechanical chelate between the bipyridine ligand and the cumulene π‐donor, stabilizes **13** to nucleophilic attack, presumably by rigidifying the framework. Finally, to demonstrate the generality of these observations, the same procedure was repeated in the case of **1 a**, **2 a**, and **3 a**; again, Tf_2_O produced a species identified in situ as the corresponding cumulene which was subsequently hydrolyzed to **5**.

In conclusion, we have identified and optimized a domino AT‐CuAAC‐rearrangement pathway for the synthesis of acrylamide‐based rotaxanes from propargylic alcohols and azides in good to excellent yield. Although the triazole product of the CuAAC has been shown to rearrange with extrusion of N_2_ when the azide component bears an electron‐withdrawing group, either under CuAAC conditions,^23^ or subsequently in the presence of transition‐metal catalysts,^26^ azides **2** do not fit these general substrate classes and acrylamide formation was not observed in the corresponding non‐interlocked products. The mechanical bond appears to play a key role in the mechanism by stabilizing and directing the reactivity of the Cu^I^‐triazolide intermediate to the degree that it is possible to generate a leaving group by protonation of a hydroxy group in preference to protonation of the Cu−C bond. The mechanical bond has previously been shown to alter the reactivity of the interlocked covalent sub‐components by sterically stabilizing reactive functionalities,^27^ controlling the reactivity of catalytic moieties^28^ or by modulating the intercomponent reactions of functional groups.28i, 29 To the best of our knowledge this is however the first time that the mechanical bond has been shown to alter the chemoselectivity of a reaction used in its own formation.

These results add another dimension to the active template approach, namely the ability to access products that are not formed in the non‐interlocked manifold, and suggests that even more complex reaction schemes are possible if the augmented reactivity of mechanically bonded intermediates can be harnessed. Furthermore, by employing a macrocycle as a temporary auxiliary, mechanical bonding may allow expedient access to non‐interlocked targets using such novel reactivity.29c Indeed, this proved the most direct route to the non‐interlocked axle of rotaxanes **5**–**7** which was produced by acid‐mediated cleavage of the macrocycle of rotaxane **6** (see the Supporting Information).

## Conflict of interest

The authors declare no conflict of interest.

## Supporting information

As a service to our authors and readers, this journal provides supporting information supplied by the authors. Such materials are peer reviewed and may be re‐organized for online delivery, but are not copy‐edited or typeset. Technical support issues arising from supporting information (other than missing files) should be addressed to the authors.

Supplementary
