# Supplementary material for: Chemical Consequences of the Mechanical Bond: A Tandem Active Template‐Rearrangement Reaction
Source: Angew Chem Int Ed Engl. 2019 Feb 14;58(12):3875–9. doi: 10.1002/anie.201813950 (PMC6589916; doi:10.1002/anie.201813950)
Supplement: Supplementary file 1 — Supplementary [file ANIE-58-3875-s001.pdf]

Angewandte Chemie
Eine Zeitschrift der Gesellschaft Deutscher Chemiker

Supporting Information

Chemical Consequences of the Mechanical Bond: A Tandem Active Template-Rearrangement Reaction

*Florian Modicom+, Ellen M. G. Jamieson+, Elise Rochette, and Stephen M. Goldup**

anie_201813950_sm_miscellaneous_information.pdf

1. General Experimental **S2**

2. Synthesis of substrates **S3**

*3,5-Di-tert-butylbenzaldehyde (**S1**)* *S3*
*1-(3,5-Di-tert-butylphenyl)prop-2-yn-1-ol (**3a**)* *S4*
*1,3-Di-tert-butyl-5-(1-methoxyprop-2-yn-1-yl)benzene (**3d**)* *S6*
*1-(3,5-Di-tert-butylphenyl)ethan-1-one (**S2**)* *S7*
*2-(3,5-Di-tert-butylphenyl)but-3-yn-2-ol (**3c**)* *S9*
*1,3-Di-tert-butyl-5-(prop-2-yn-1-yl)benzene (**3e**)* *S10*
*1-(3,5-Di-tert-butylphenyl)but-3-yn-2-ol (**3b**)* *S12*

3. Synthesis of acrylamide rotaxanes **S14**

General Procedure A *S14*
*Rotaxane **5*** *S14*
*Rotaxane **6*** *S18*
*Rotaxane **7*** *S22*
*Rotaxane **8*** *S26*
*Rotaxane **9*** *S30*
*Rotaxane **10*** *S34*
*Rotaxane **11*** *S38*

4. Synthesis of triazole rotaxanes **S42**

General Procedure B *S42*
*Rotaxane **4*** *S42*
*Rotaxane **S4*** *S46*
*Rotaxane **S5*** *S49*
*Rotaxane **S6*** *S52*
*Rotaxane **S7*** *S55*
*Rotaxane **S8*** *S59*
*Rotaxane **S9*** *S62*
*Rotaxane **S10*** *S65*
*Rotaxane **S11*** *S68*
*Axle **S12*** *S71*
*Thread **S13*** *S74*

5. Single crystal X-ray analysis of rotaxanes 4, 5, 6 and 8 **S77**

6. Optimisation of reaction conditions for the rearrangement reaction **S82**

7. Kinetic Study of the reaction of 1a, 2a and 3a. **S83**

8. Control experiments: axle formation under conditions optimised for the formation of acrylamide rotaxane 5 **S84**

9. Mechanistic studies: rearrangement of triazolide 12 under aqueous conditions **S86**

10. Mechanistic studies: rearrangements triggered by Tf_2O under anhydrous conditions **S89**

*i. In situ ^1H NMR analysis of the reaction of the product of **1a**, **2a** and **3c** with Tf_2O* *S89*
*ii. In situ ^1H NMR analysis of the reaction of **1a**, **2a** and **3a** followed by Tf_2O* *S92*

11. Preliminary computational analysis of the mechanism of the rearrangement process **S93**

*i. Preparation of a truncated model **Ia** of triazolide **S15*** *S93*
*ii. DFT evaluation of the pathway of N_2 loss from truncated triazolide model **Ia*** *S93*
*iii. DFT evaluation of the pathway of N_2 loss from truncated triazole model **Ib*** *S96*
iv. Conclusions *S96*

12. References **S98**

1. General Experimental

Synthesis: Unless otherwise stated, all reagents, including anhydrous solvents, were purchased from commercial sources and used without further purification. All reactions were carried out under an atmosphere of N_2 using anhydrous solvents unless otherwise stated. Petrol refers to the fraction of petroleum ether boiling in the range 40–60 °C. EDTA-NH_3 solution refers to an aqueous solution of NH_3 (17% w/w) with 0.1 M sodium-ethylenediaminetetraacetate. Flash column chromatography was performed using Biotage Isolera-4 or Biotage Isolera-1 automated chromatography system, employing Biotage SNAP or ZIP cartridges. Analytical TLC was performed on precoated silica gel plates (0.25 mm thick, 60F254, Merck, Darmstadt, Germany) and observed under UV light or with potassium permanganate solution. Microwave heating of reactions was achieved using a Biotage Initiator+ microwave system. Reactions were run at a maximum power level of 400 W in crimp-cap sealed vials (CEM Ltd.). The temperature was monitored automatically and maintained at the set level throughout the reaction after an initial ramp period, typically ~ 1 minute.

Analysis: NMR spectra were recorded on Bruker AV400, AV3-400 or AV500 instrument, at a constant temperature of 298 K. Chemical shifts are reported in parts per million from low to high field and referenced to residual solvent. Coupling constants (*J*) are reported in Hertz (Hz). Standard abbreviations indicating multiplicity were used as follows: m = multiplet, quint = quintet, q = quartet, t = triplet, d = doublet, s = singlet, app. = apparent, br = broad. Signal assignment was carried out using 2D NMR methods (HSQC, HMBC, COSY, NOESY) where necessary. All melting points were determined using a Griffin apparatus. Low resolution mass spectrometry was carried out by the mass spectrometry services at the University of Southampton (Waters TQD mass spectrometer equipped with a triple quadrupole analyser with UHPLC injection [BEH C18 column; MeCN-hexane gradient {0.2% formic acid}]). High resolution mass spectrometry was carried out by the mass spectrometry services at the University of Southampton (MaXis, Bruker Daltonics, with a Time of Flight (TOF) analyser; samples were introduced to the mass spectrometer via a Dionex Ultimate 3000 autosampler and uHPLC pump in a gradient of 20% acetonitrile in hexane to 100% acetonitrile (0.2% formic acid) over 5 min at 0.6 mL min; column: Acquity UPLC BEH C18 (Waters) 1.7 micron 50 × 2.1mm).

The following compounds were synthesised according to literature procedures:
2-(3,5-di-*tert*-butylphenyl)acetaldehyde **S3**,[1] 1-azido-3,5-di-*tert*-butylbenzene **2a**,[2] 1-(azidomethyl)-3,5-di-*tert*-butylbenzene **2b**,[3] 1-((3-azidopropoxy)methyl)-3,5-di-*tert*-butylbenzene **2c**,[4] macrocycle **1a**, **1b** **1c** and **1d**.[5]

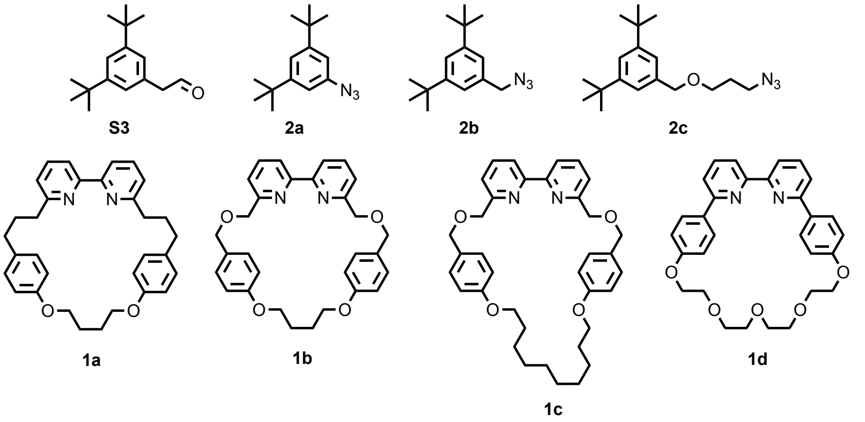

2. Synthesis of substrates

3,5-Di-tert-butylbenzaldehyde (**S1**)

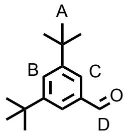

3,5-Di-tert-butyltoluene (6.1 g, 29.9 mmol) was treated with NBS (11.0 g, 62.0 mmol) and AIBN (33.8 mg, 0.2 mmol) in PhCl (150 mL), and stirred for 16 h at 140 °C. The mixture was cooled to rt and passed through a Celite plug. The solvent was removed *in vacuo.* The residue was slurried in 20 mL of 1:1 water/ethanol. Hexamethylenetetramine (12.2 g, 86.9 mmol) was added, and the mixture was heated to reflux for 4 h, cooled to rt, diluted with PhMe-Et_2O (1:1, 130 mL) and the phases separated. The organic phase was washed with brine (50 mL), dried ($MgSO_4$), filtered and the solvent removed *in vacuo.* Chromatography (petrol/Et_2O 95:5) gave **S1** as a white solid (5.0 g, 77%). M.p. 84 - 86 °C. Spectra were consistent with those previously reported.[6] ^{1}H NMR (400 MHz, $CDCl_3$) δ: 10.01 (s, 1H, H_D), 7.75-7.68 (m, 3H, H_B and H_C), 1.37 (s, 18H, H_A). ^{13}C NMR (101 MHz, $CDCl_3$) δ: 193.3, 152.0, 136.3, 129.9, 124.2, 35.2, 31.3.

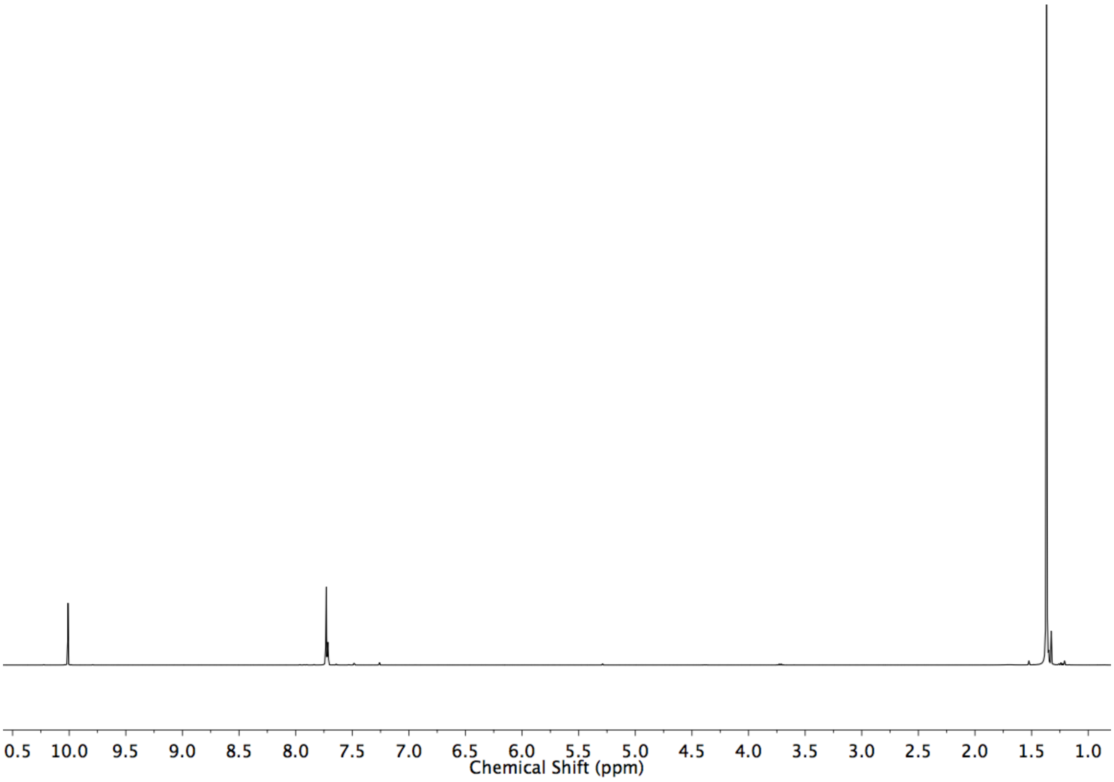

Figure S1 ^{1}H NMR ($CDCl_3$, 400 MHz) of **S1**

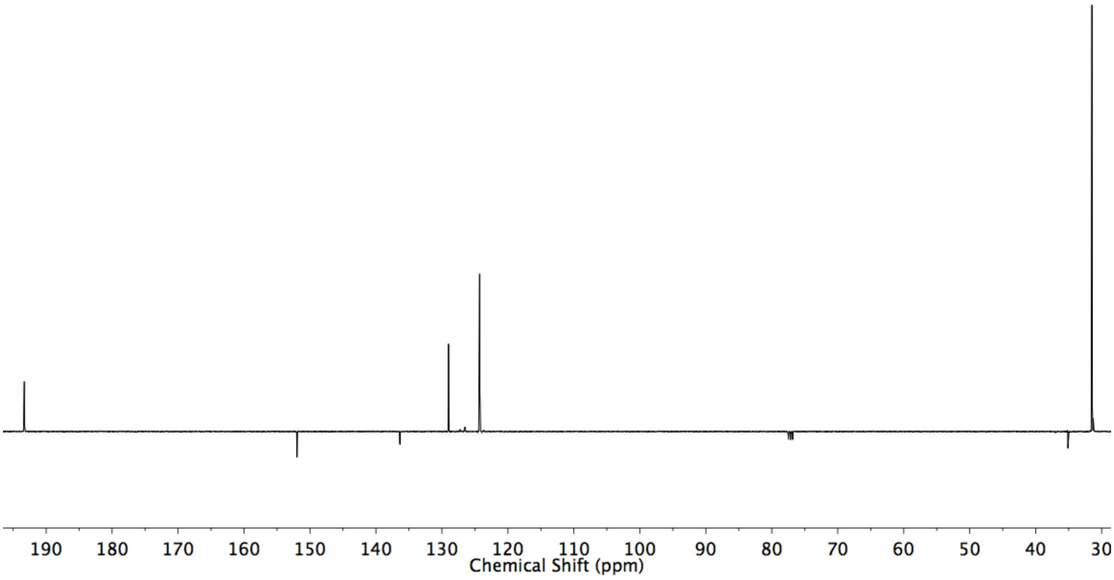

Figure S2 JMOD NMR ($CDCl_3$, 101 MHz) of **S1**

1-(3,5-Di-*tert*-butylphenyl)prop-2-yn-1-ol (**3a**)

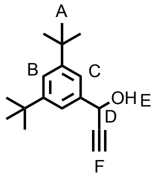

S1 (1.4 g, 6.4 mmol) was dissolved in THF (20 mL) at 0 °C under N_2. Ethynylmagnesium chloride (0.6 M in THF, 10 mmol, 16 mL) was added dropwise. The mixture was stirred for 20 h at rt. Saturated $NH_4Cl_{(aq)}$ (10 mL) was added and the solvent removed *in vacuo*. The residue was extracted with Et_2O (3 × 20 mL). The combined organic layers were washed with brine (10 mL), dried ($MgSO_4$), filtered and the solvent removed *in vacuo.* Chromatography (petrol/ethyl acetate 9:1) gave **3a** as a light-yellow oil (1.5 g, 97%). ^1H NMR (400 MHz, $CDCl_3$) δ: 7.44-7.39 (m, 3H, H_B, H_C), 5.46 (d, *J* =2.2, 1H, H_D), 2.67 (d, *J* =2.2, 1H, H_F), 2.14 (br. s, 1H, H_E), 1.34 (s, 18H, H_A). ^{13}C NMR (101 MHz, $CDCl_3$) δ: 151.2, 139.3, 122.7, 121.0, 84.1, 74.7, 65.1, 35.0, 31.6. HR-EI-MS *m/z* = 244.18118 $M^{+\cdot}$ (calc. for $C_{17}H_{24}O$ 244.18217).

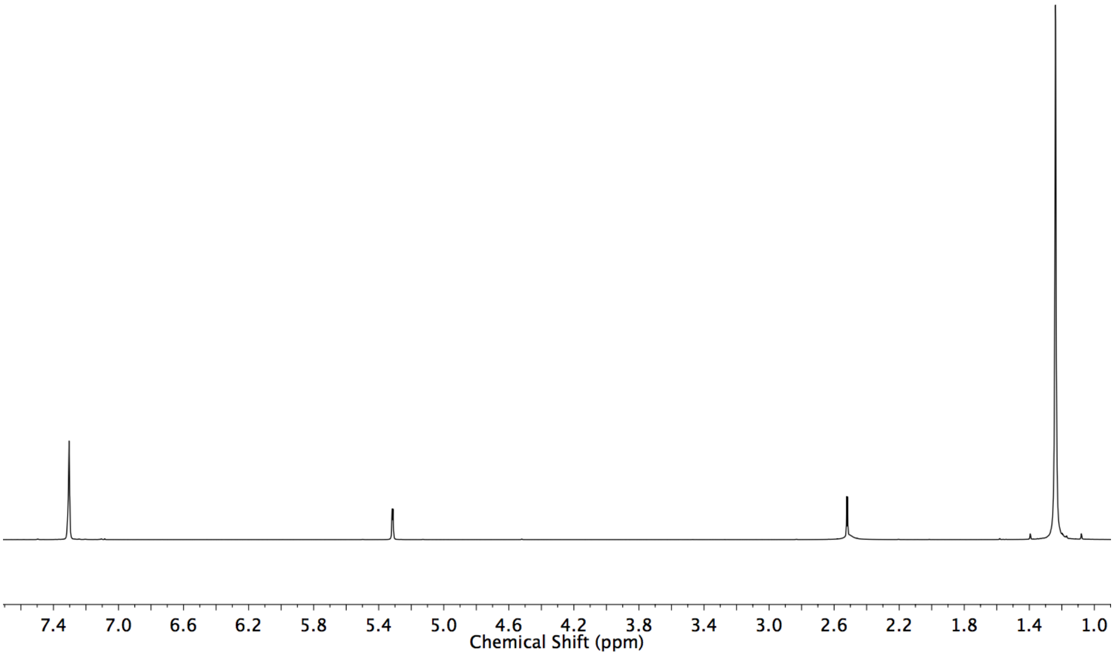

Figure S3 ^{1}H NMR ($CDCl_3$, 400 MHz) of **3a**

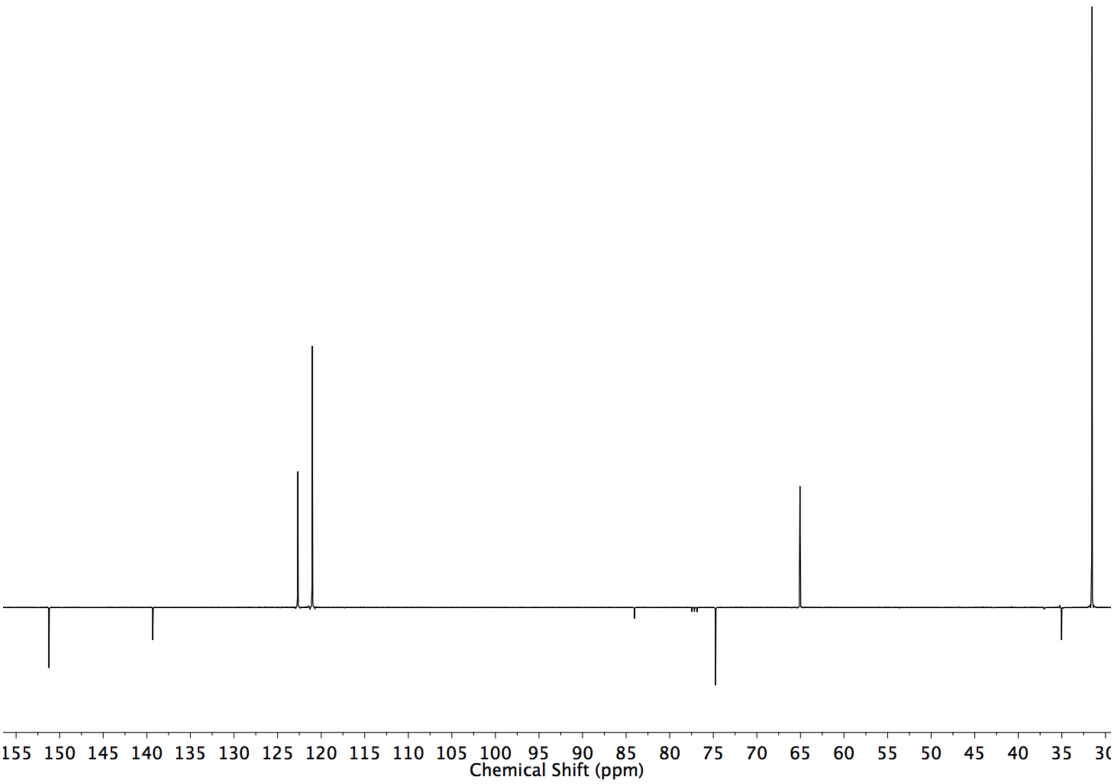

Figure S4 JMOD NMR ($CDCl_3$, 101 MHz) of **3a**

1,3-Di-*tert*-butyl-5-(1-methoxyprop-2-yn-1-yl)benzene (**3d**)

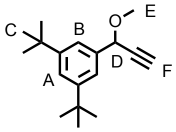

3a (50 mg, 0.2 mmol) was dissolved in THF (1 mL) at 0 °C under N_2. NaH (12 mg, 0.3 mmol) was added in one portion. The mixture was stirred for 10 minutes and MeI (26 µL, 0.4 mmol) was added. The mixture was stirred for 3 h at rt. Saturated $NH_4Cl_{(aq)}$ (5 mL) was added and the mixture extracted with Et_2O (3 × 10 mL). The combined organic layers were washed with brine (5 mL), dried ($MgSO_4$), filtered and the solvent removed *in vacuo*. Chromatography (petrol/Et_2O 95:5) gave **3d** as a yellow oil (52 mg, 98%). ^1H NMR (400 MHz, $CDCl_3$) δ: 7.40 (t, *J* =1.8, 1H, H_A), 7.34 (d, *J* =1.8, 2H, H_B), 5.05 (d, *J* =2.2, 1H, H_D), 3.47 (s, 3H, H_E), 2.65 (d, *J* =2.2, 1H, H_F), 1.33 (s, 18H, H_C). ^{13}C NMR (101 MHz, $CDCl_3$) δ: 151.1, 137.1, 122.9, 121.8, 81.9, 75.7, 73.7, 56.3, 35.1, 31.6. HR-EI-MS *m/z* = 258.19835 M^+ (calc. for $C_{18}H_{26}O$ 258.19782).

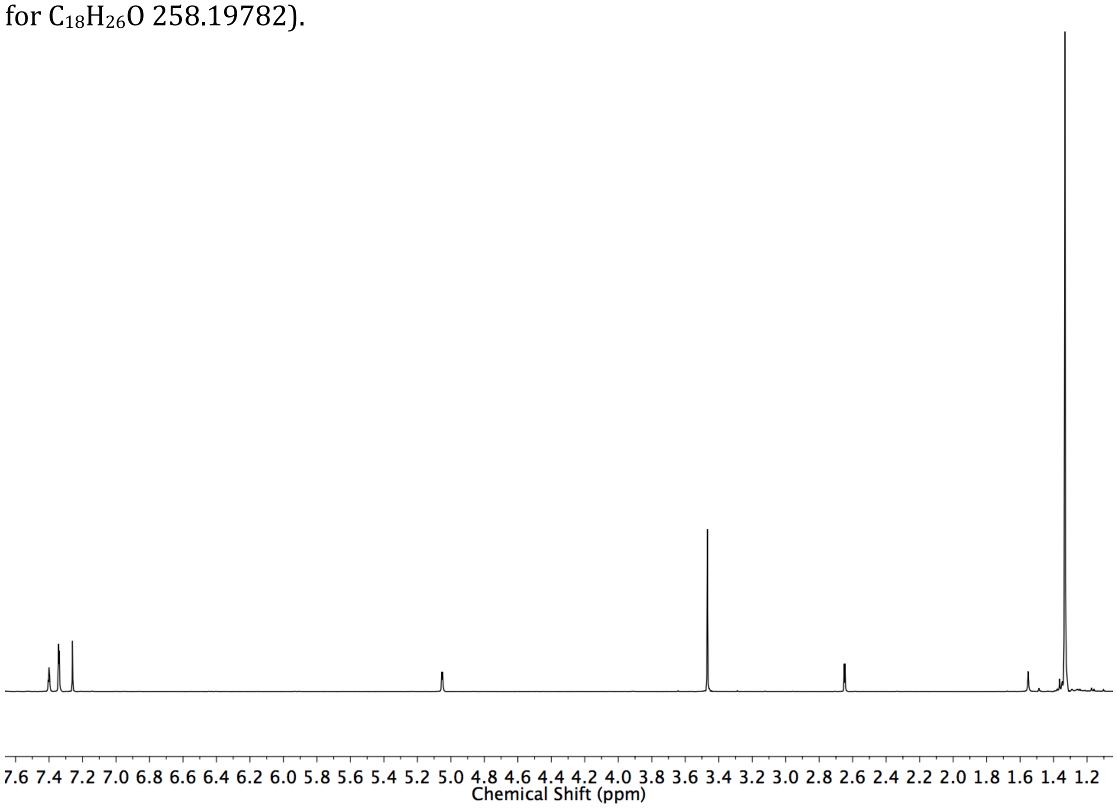

Figure S5 ^1H NMR ($CDCl_3$, 400 MHz) of **3d**

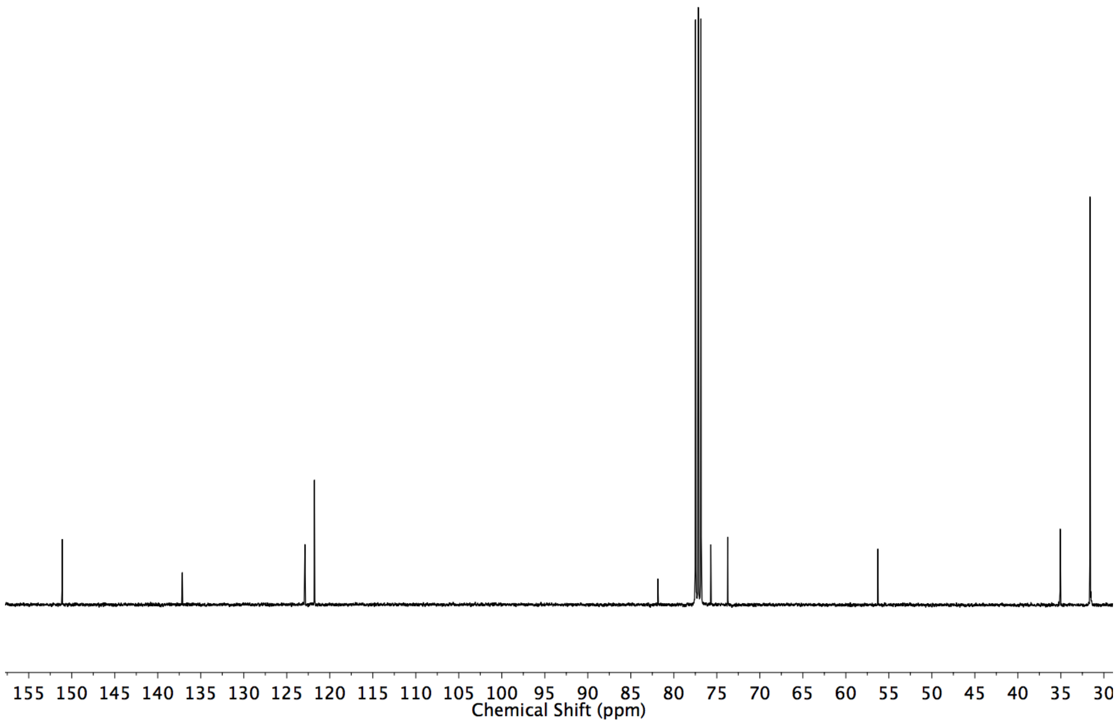

Figure S6 ^{13}C NMR ($CDCl_3$, 101 MHz) of **3d**

1-(3,5-Di-*tert*-butylphenyl)ethan-1-one (**S2**)

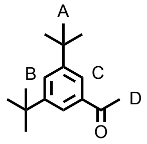

3,5-Di-tert-butylbenzoic acid (470 mg, 2 mmol) was dissolved in THF (5 mL) at −78 °C under N_2. MeLi (1.6 M in Et_2O, 2.75 mL, 4.4 mmol) was added dropwise. The mixture was slowly warmed to rt and stirred for 30 minutes then saturated $NH_4Cl_{(aq)}$ (10 mL) was added and the mixture extracted with petrol (3 × 20 mL), dried ($MgSO_4$), filtered and the solvent removed *in vacuo*. Chromatography (petrol/ethyl acetate 95:5) gave **S2** as a colourless oil (370 mg, 80%). Spectra were consistent with those previously reported.[7] ^1H NMR (400 MHz, $CDCl_3$) δ: 7.81 (t, *J* =1.8, 1H, H_B), 7.65 (d, *J* =1.8, 2H, H_C), 2.61 (s, 3H, H_D), 1.36 (s, 18H, H_A). ^{13}C NMR (101 MHz, $CDCl_3$) δ: 199.1, 151.4, 137.0, 127.5, 122.7, 35.1, 31.5, 29.9.

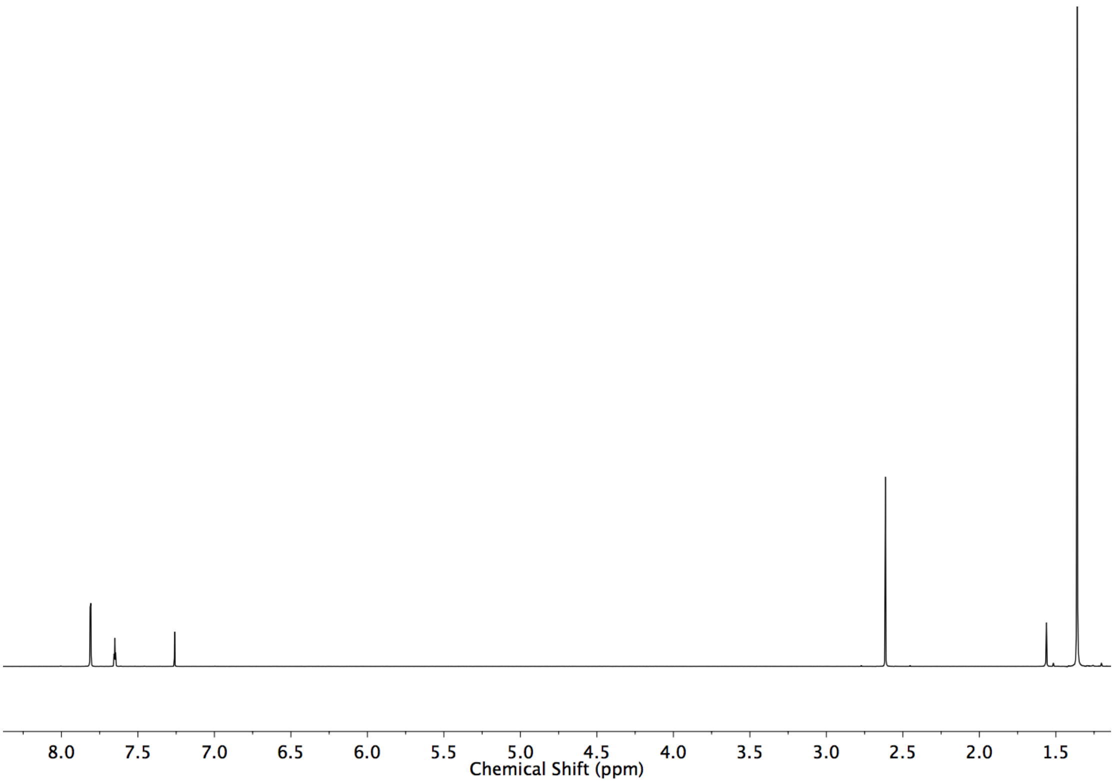

Figure S7 ^{1}H NMR ($CDCl_3$, 400 MHz) of **S2**

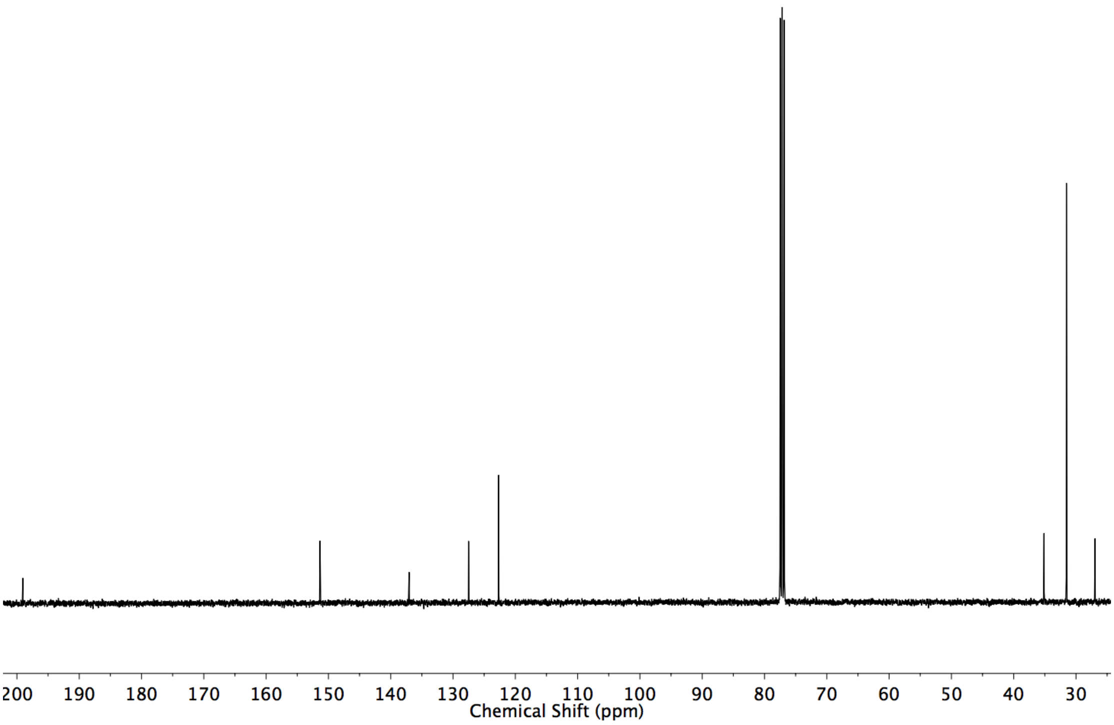

Figure S8 ^{13}C NMR ($CDCl_3$, 101 MHz) of **S2**

2-(3,5-Di-*tert*-butylphenyl)but-3-yn-2-ol (**3c**)

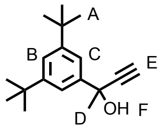

S2 (370 mg, 1.6 mmol) was dissolved in THF (3 mL) at 0 °C under N_2. Ethynylmagnesium chloride (0.6 M in THF, 2.4 mmol, 4 mL) was added dropwise. The mixture was stirred for 16 h at rt. Saturated $NH_4Cl_{(aq)}$ (5 mL) was added and the solvent removed *in vacuo*. The residue was extracted with Et_2O (3 × 20 mL). The combined organic layers were washed with brine (10 mL), dried ($MgSO_4$), filtered and the solvent removed *in vacuo*. Chromatography (petrol/ethyl acetate 9:1) gave **3c** as a colorless oil (200 mg, 48%). ^1H NMR (400 MHz, $CDCl_3$) δ: 7.53 (d, *J* =1.8, 2H, H_C), 7.38 (t, *J* =1.8, 1H, H_B), 2.68 (s, 1H, H_E), 2.37 (br. s, 1H, H_F), 1.81 (s, 3H, H_D), 1.35 (s, 18H, H_A). ^{13}C NMR (101 MHz, $CDCl_3$) δ: 150.9, 144.3, 122.1, 119.2, 87.8, 73.0, 70.6, 35.2, 33.3, 31.6. HR-EI-MS *m/z* = 258.19876 M^+ (calc. for $C_{18}H_{26}O$ 258.19782).

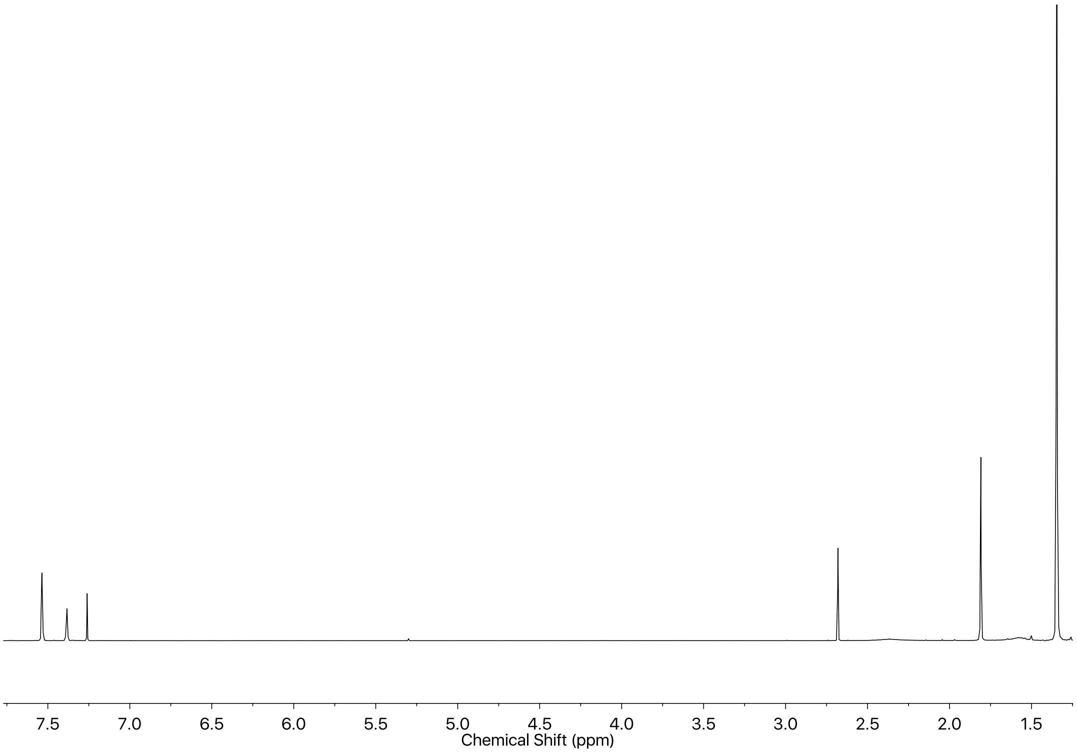

Figure S9 ^1H NMR ($CDCl_3$, 400 MHz) of **3c**

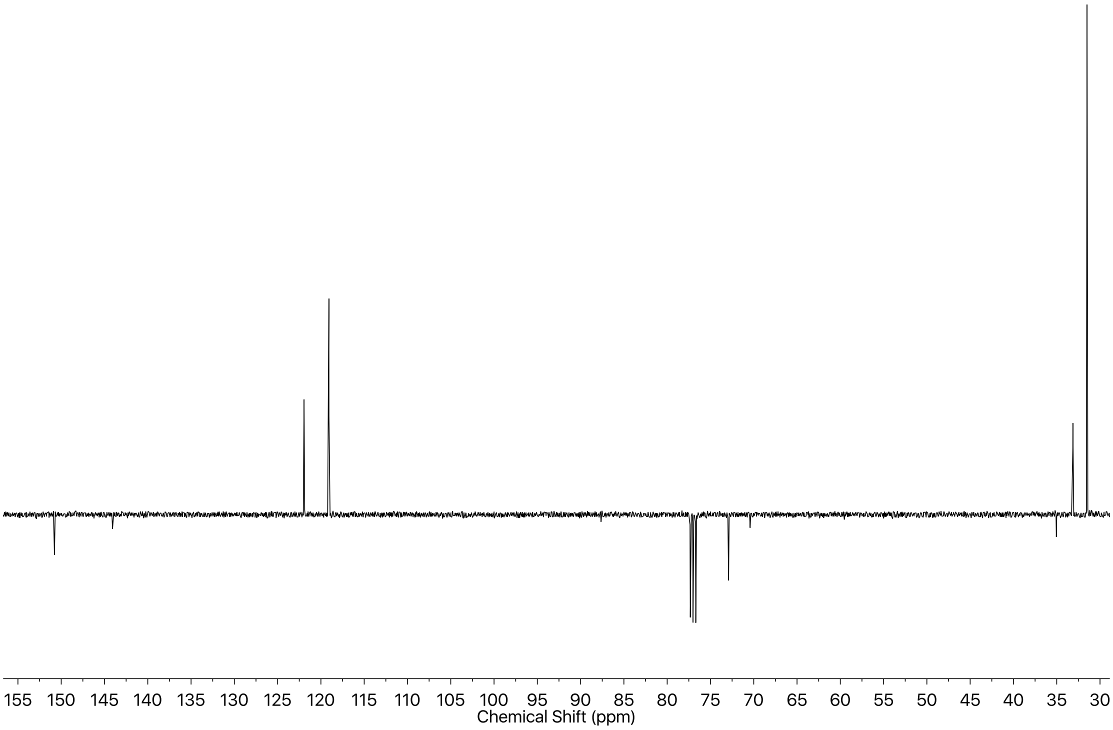

Figure S10 JMOD NMR ($CDCl_3$, 101 MHz) of **3c**

1,3-Di-*tert*-butyl-5-(prop-2-yn-1-yl)benzene (**3e**)

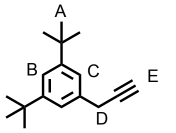

S3 (200 mg, 0.86 mmol), dimethyl(1-diazo-2-oxo-propyl)phosphonate (192 mg, 1.00 mmol), and K_2CO_3 (235 mg, 1.7 mmol) were stirred in MeOH (2 mL) for 19 h at rt. The mixture was diluted with CH_2Cl_2 (10 mL), filtered and the filtrate washed with H_2O (5 mL) and brine (5 mL). The combined aqueous layers were extracted with CH_2Cl_2 (10 mL). The combined organic layers were dried ($MgSO_4$), filtered and the solvent removed *in vacuo.* Chromatography (petrol) gave **3e** as a colorless oil (160 mg, 81%). ^{1}H NMR (400 MHz, $CDCl_3$) δ: 7.31 (t, *J* =1.8, 1H, H_B), 7.20 (d, *J* =1.8, 2H, H_C), 3.60 (d, *J* =2.6, 2H, H_D), 2.18 (t, *J* =2.6, 1H, H_E), 1.33 (s, 18H, H_A). ^{13}C NMR (101 MHz, $CDCl_3$) δ: 151.2, 135.2, 122.3, 120.9, 82.6, 70.4, 35.0, 31.6, 25.3. HR-EI-MS *m/z* = 228.18696 M^+ (calc. for $C_{17}H_{24}$ 228.18725).

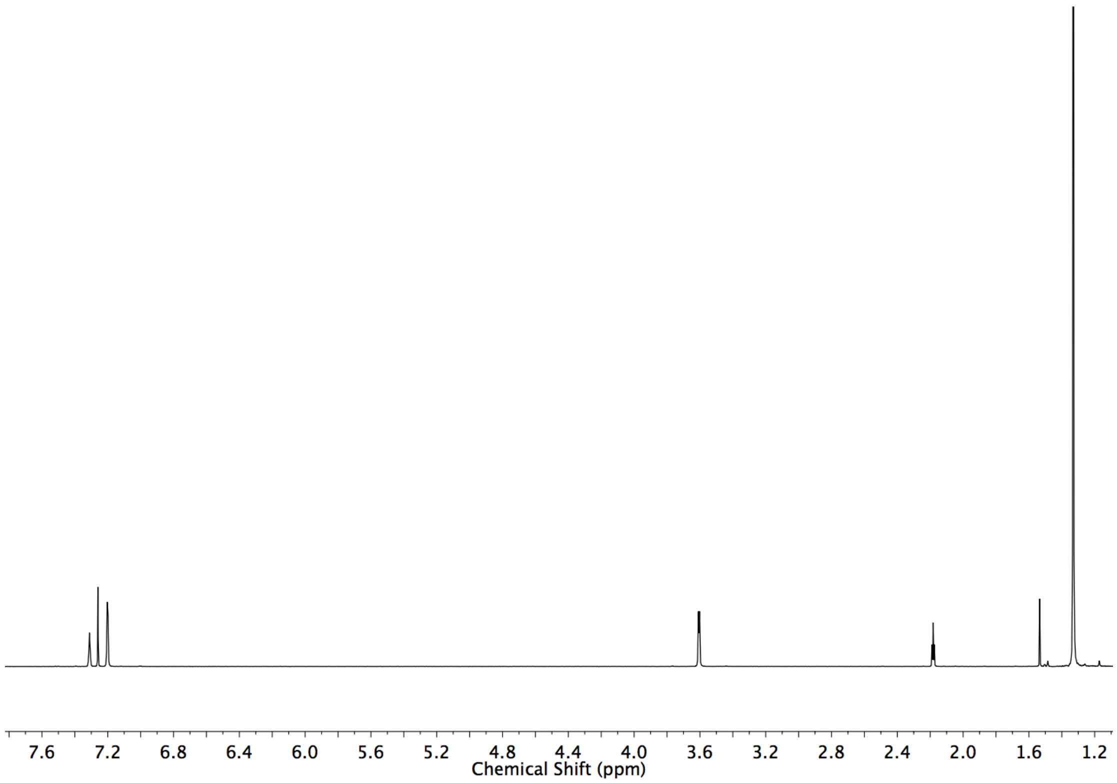

Figure S11 ^{1}H NMR ($CDCl_3$, 400 MHz) of **3e**

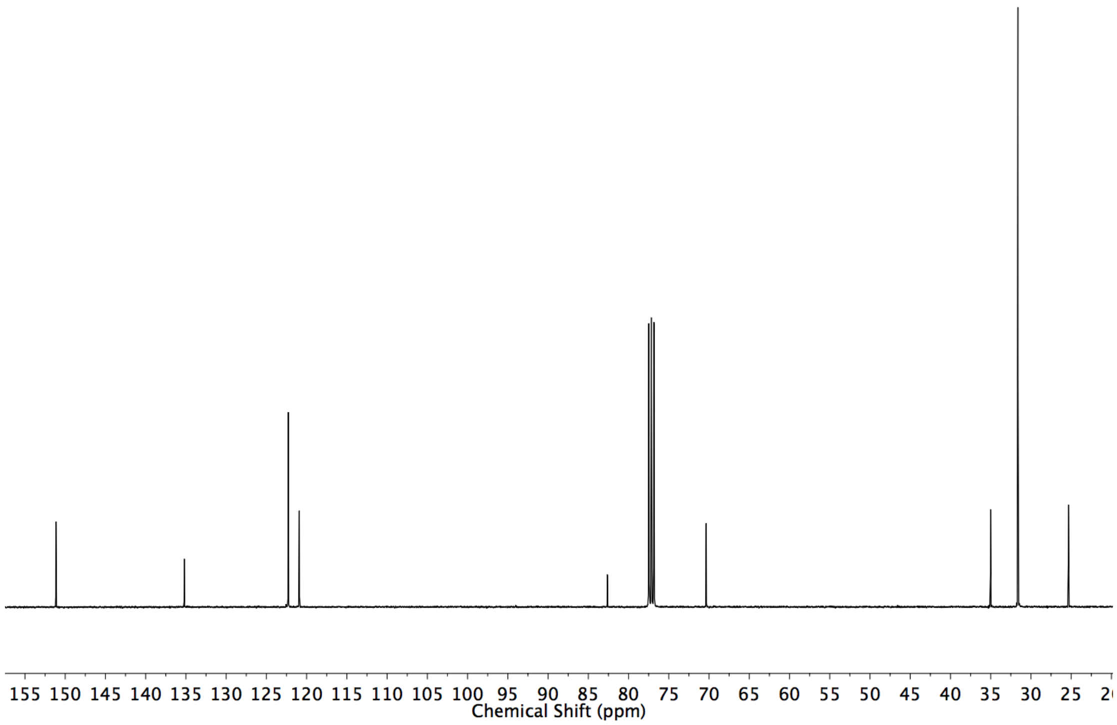

Figure S12 ^{13}C NMR ($CDCl_3$, 101 MHz) of **3e**

1-(3,5-Di-*tert*-butylphenyl)but-3-yn-2-ol (**3b**)

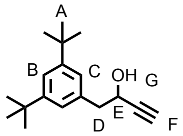

S3 (400 mg, 1.7 mmol) was dissolved in THF (3 mL) at 0 °C under N_2. Ethynylmagnesium bromide (0.5 M in THF, 2.0 mmol, 4.0 mL) was added dropwise. The reaction mixture was stirred for 20 h at rt. Saturated $NH_4Cl_{(aq)}$ (5 mL) was added and the solvent was removed *in vacuo*. The residue was extracted with Et_2O (3 × 20 mL). The combined organic layers were washed with brine (10 mL), dried ($MgSO_4$), filtered and the solvent removed *in vacuo.* Chromatography (petrol/Et_2O 95:5) gave **3b** as a colourless oil (160 mg, 36 %). ^1H NMR (400 MHz, $CDCl_3$) δ: 7.33 (t, *J* =1.9, 1H, H_B), 7.13 (d, *J* =1.9, 2H, H_C), 4.58 (app. qd, *J* =6.1, 2.0, 1H, H_E), 3.03 (qd, *J* =16.3, 6.5, 2H, H_D), 2.50 (d, *J* =2.1, 1H, H_F), 1.88 (d, *J* =6.1, 1H, H_G), 1.33 (s, 18H, H_A). ^{13}C NMR (101 MHz, $CDCl_3$) δ: 151.0, 135.1, 124.2, 121.2, 84.6, 73.7, 63.2, 44.6, 34.9, 31.6. HR-EI-MS *m/z* = 258.19765 M^+ (calc. for $C_{18}H_{26}O$ 258.19782).

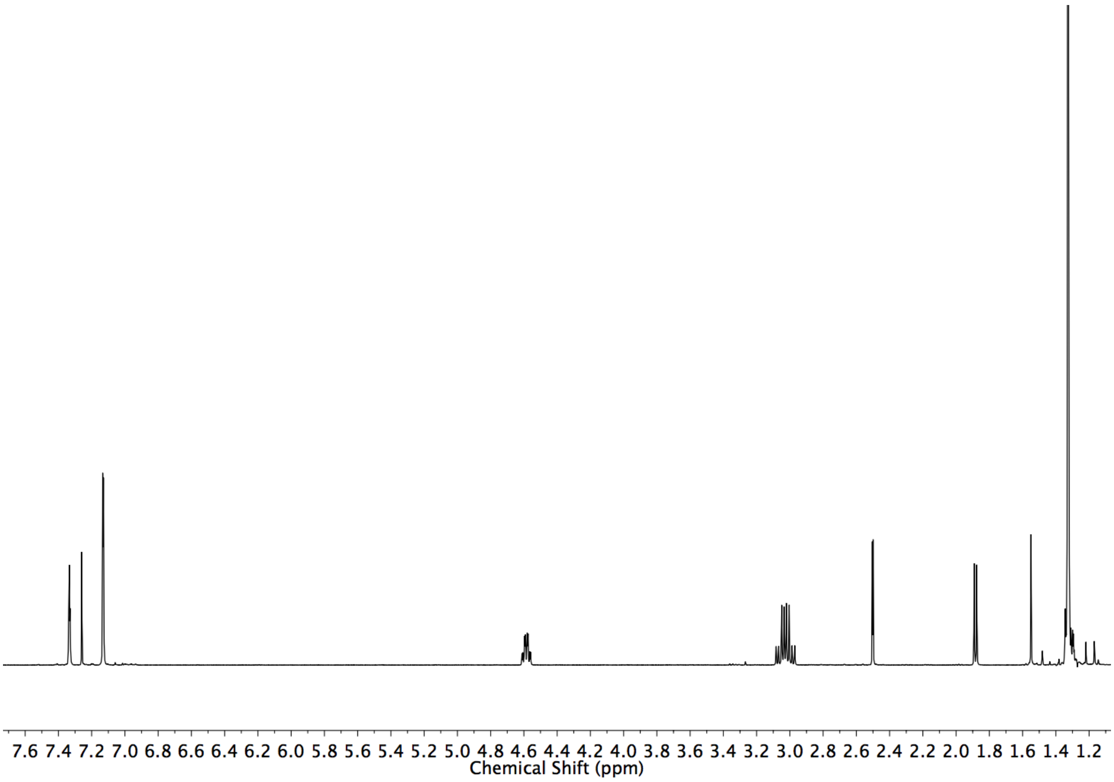

Figure S13 ^1H NMR ($CDCl_3$, 400 MHz) of **3b**

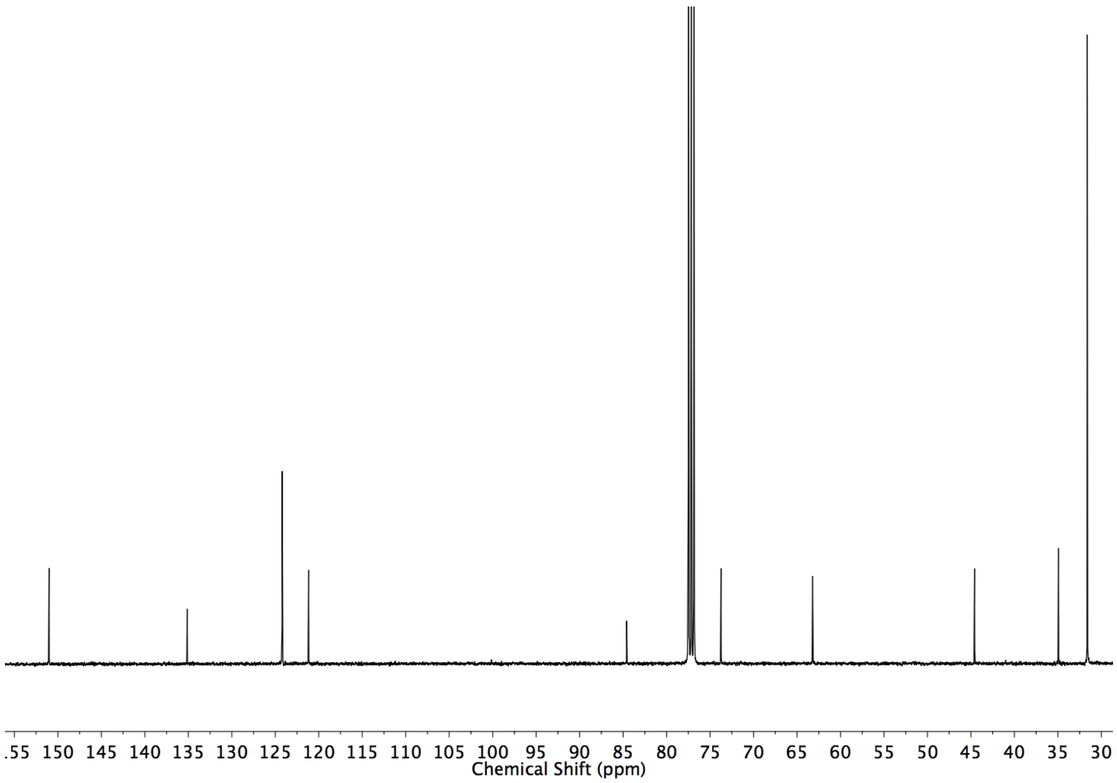

Figure S14 ^{13}C NMR ($CDCl_3$, 101 MHz) of **3b**

3. Synthesis of acrylamide rotaxanes

General Procedure A

$KF_{(aq)}$ (0.1 M, 0.8 eq.) was added to a solution of **alkyne** (1.2 eq.), **azide** (1.2 eq.), **macrocycle** (1 eq.) and $[Cu(MeCN)_4]PF_6$ (0.96 eq.) in THF (72 mL/mmol) in a microwave vial (CEM Ltd.) and the vial sealed. The orange mixture was stirred at 70 °C under microwave irradiation for 1 hour. The reaction mixgture was diluted with CH_2Cl_2 (200 mL/mmol), washed with EDTA-NH_3 solution (100 mL/mmol). The aqueous layer was extracted with CH_2Cl_2 (2 × 100 mL/mmol). The combined organic extracts were washed with brine (100 mL/mmol), dried ($MgSO_4$), filtered and the solvent removed *in vacuo*.

Rotaxane **5**

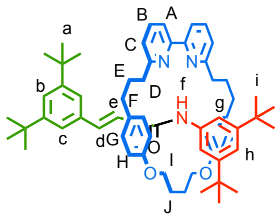

Prepared according to **general procedure A** with **1a** (24.0 mg, 0.05 mmol), $[Cu(MeCN)_4]PF_6$ (17.9 mg, 0.048 mmol), **2a** (13.8 mg, 0.06 mmol), and **3a** (14.6 mg, 0.06 mmol). Chromatography (petrol with a gradient of 0 to 20% Et_2O) gave **5** as a white foam (44.0 mg, 95%). ^{1}H NMR (500 MHz, $CDCl_3$) δ: 9.91 (S, 1H, H_f), 7.65 (t, *J* =7.8, 2H, H_B), 7.49 (dd, *J* =7.8, 1.0, 2H, H_A), 7.23 (t, *J* = 1.8, 1H, H_h), 7.14 – 7.10 (m, 4H, H_g, H_C), 6.94 – 6.92 (m, 3H, H_b, H_c), 6.85 (d, *J* =15.5, 1H, H_d), 6.69 (s, 8H, H_G, H_H), 6.29 (d, *J* =15.5, 1H, H_e), 4.70 - 4.55 (m, 2H, H_I), 4.21 - 4.10 (m, 2H, $H_{I'}$), 2.62 – 2.42 (m, 8H, H_D, H_F), 2.40 – 2.30 (m, 2H, H_J), 2.11 – 2.00 (m, 2H, $H_{J'}$), 1.88 – 1.64 (m, 4H, H_E), 1.23 (s, 18H, H_a), 1.17 (s, 18H, H_i). ^{13}C NMR (126 MHz, $CDCl_3$) δ: 163.8, 163.5, 157.4, 156.8, 150.3, 150.1, 139.6, 139.0, 137.0, 135.9, 132.4, 129.1, 123.3, 122.2, 122.0, 121.8, 119.6, 116.2, 115.0, 114.2, 66.3, 36.6, 35.2, 34.8, 34.8, 31.5, 31.5, 31.3, 25.0. HR-ESI-MS *m/z* = 926.6199 $[M+H]^+$ (calc. for $C_{63}H_{80}N_3O_3$ 926.6194).

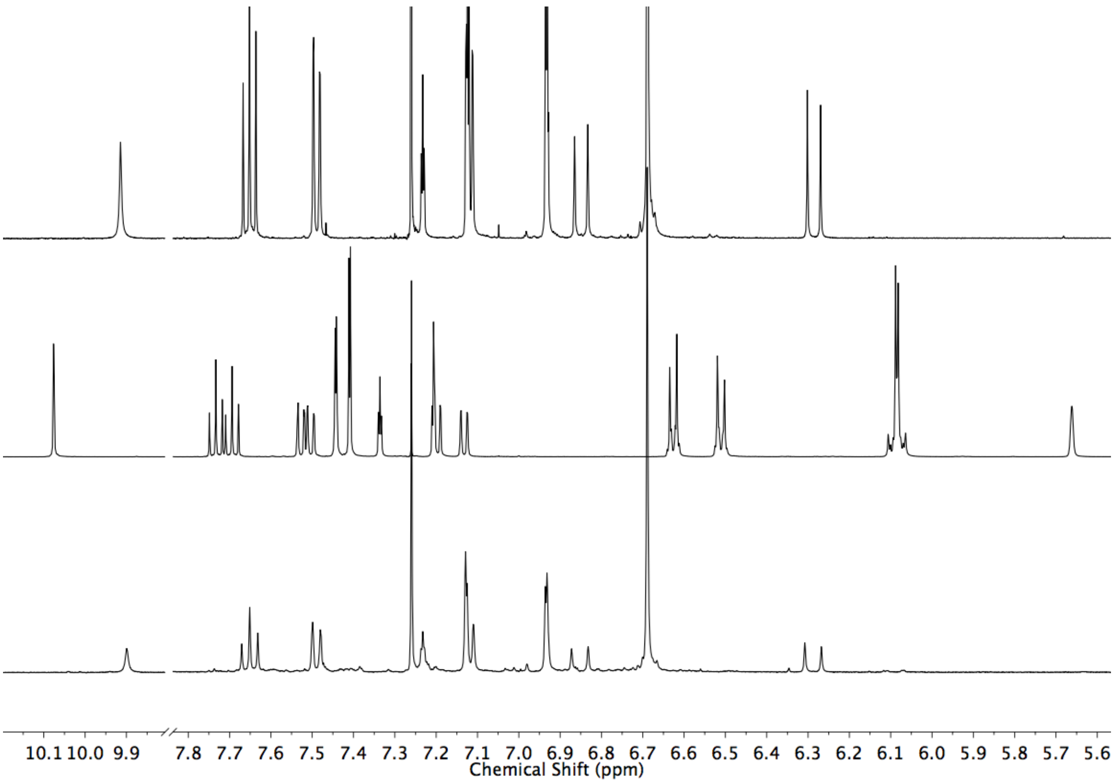

Figure S15 Stacked partial ^{1}H NMR (400 MHz, $CDCl_3$) spectra of **5** (top), **4** (middle) and the crude reaction product before chromatography (bottom). Ratio of **5** : **4** = 100:0.

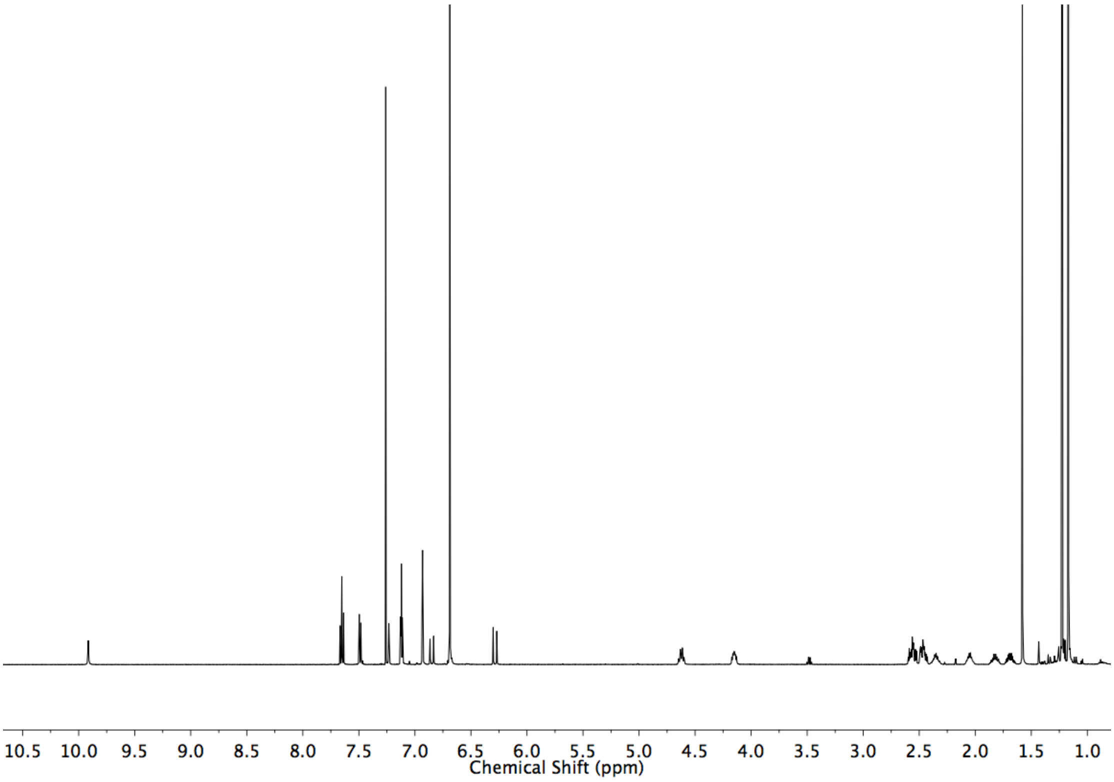

Figure S16 ^{1}H NMR ($CDCl_3$, 500 MHz) of **5**

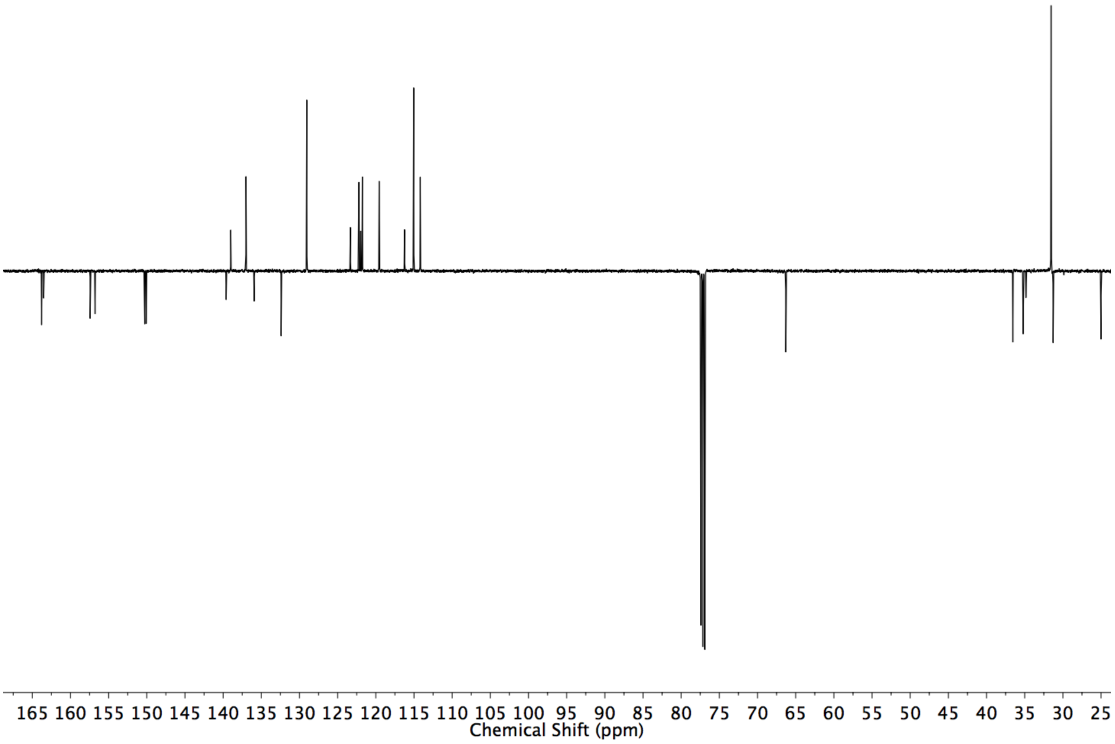

Figure S17 JMOD NMR ($CDCl_3$, 126 MHz) of **5**

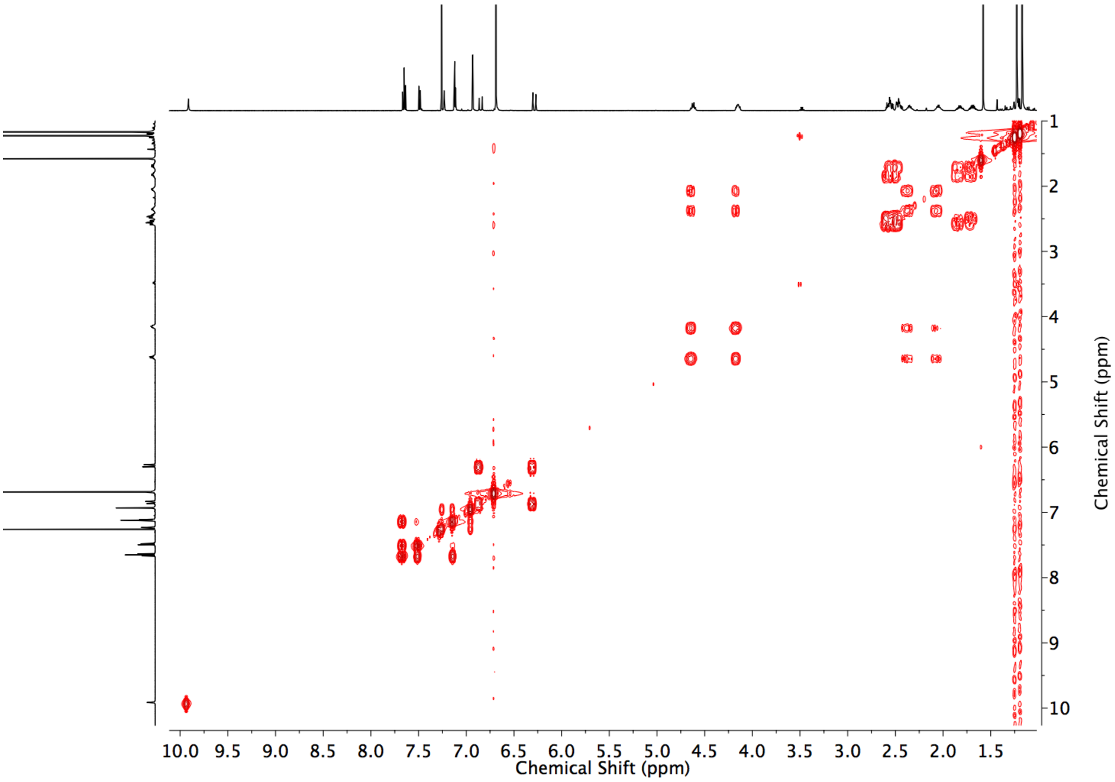

Figure S18 COSY NMR ($CDCl_3$) of **5**

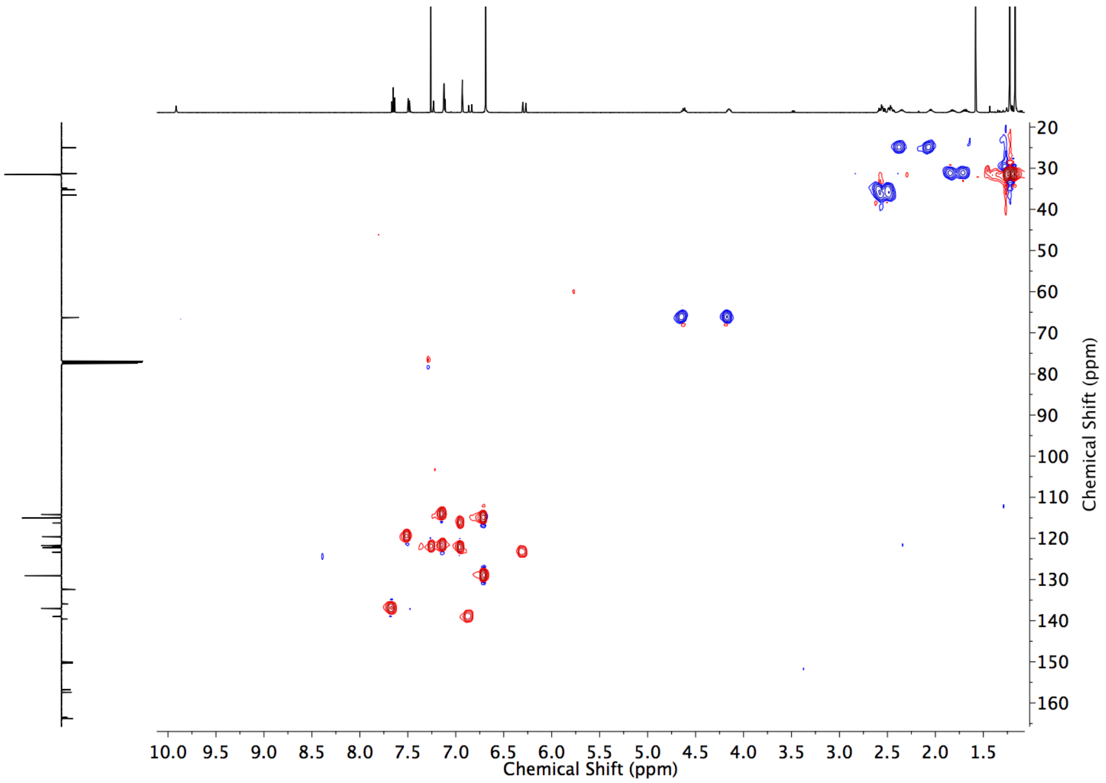

Figure S19 HSQC NMR ($CDCl_3$) of **5**

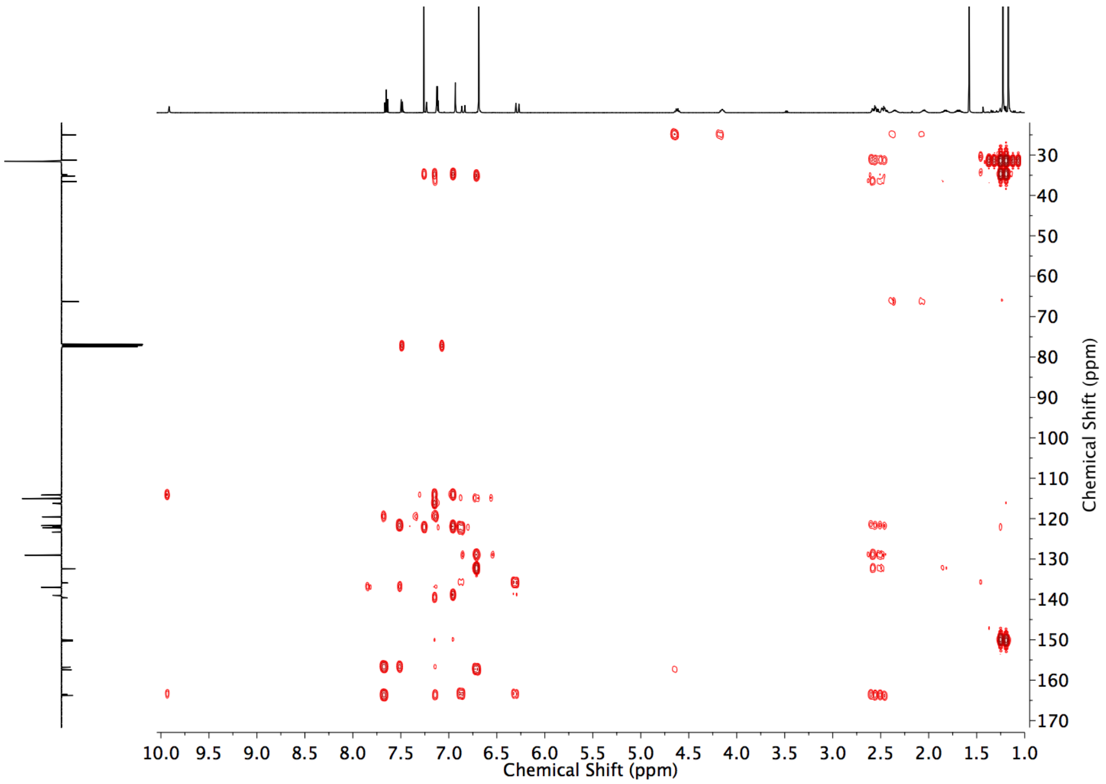

Figure S20 HMBC NMR ($CDCl_3$) of **5**

Rotaxane **6**

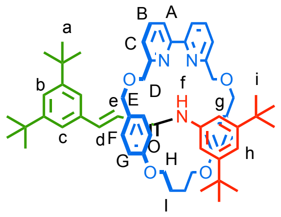

Prepared according to **general procedure A** with **1b** (48.3 mg, 0.1 mmol), $[Cu(MeCN)_4]PF_6$ (35.8 mg, 0.096 mmol), **2a** (29.3 mg, 0.12 mmol), **3a** (27.8 mg, 0.12 mmol). Chromatography (petrol with a gradient of 0 to 20% Et_2O) gave **6** as a white foam (90.0 mg, 97%). ^{1}H NMR (400 MHz, $CDCl_3$) δ: 9.94 (s, 1H, H_f), 7.77 (t, *J* =7.7, 2H, H_B), 7.67 (dd, *J* =7.7, 1.0, 2H, H_A), 7.50 (dd, *J* =7.7, 1.0, 2H, H_C), 7.27 (d, *J* =1.8, 2H, H_g), 7.23 (t, *J* =1.8, 1H, H_b), 6.98 (t, *J* =1.8, 1H, H_h), 6.91 (d, *J* =8.5, 4H, H_F), 6.81 (d, *J* =16.0, 1H, H_d), 6.79 (d, *J* =1.8, 2H, H_c), 6.70 (d, *J* =8.5, 4H, H_G), 5.92 (d, *J* =16.0, 1H, H_e), 4.77 – 4.70 (m, 2H, 2 of H_H), 4.69 (d, *J* =12.0, 2H, 2 of H_D), 4.31 (d, *J* =12.0, 2H, 2 of H_D), 4.19 (d, *J* =13.8, 2H, 2 of H_E), 4.18 – 4.12 (m, 2H, 2 of H_H), 4.02 (d, *J* =13.8, 2H, 2 of H_E), 2.46 – 2.33 (m, 2H, 2 of H_I), 2.11 – 1.99 (m, 2H, 2 of H_I), 1.21 (s, 18H, H_a or H_i), 1.19 (s 18H, H_a or H_i). ^{13}C NMR (101 MHz, $CDCl_3$) δ: 163.3, 160.1, 159.2, 154.9, 150.5, 150.3, 139.6, 139.4, 127.5, 135.6, 129.9, 128.2, 122.9, 122.3, 122.1, 120.8, 119.9, 116.4, 115.1, 114.1, 72.9, 69.5, 66.1, 34.9, 34.8, 31.5, 25.0, 24.8. HR-ESI-MS *m/z* = 930.5763 [M+H]$^+$ (calc. for $C_{63}H_{76}N_3O_5$ 930.5779).

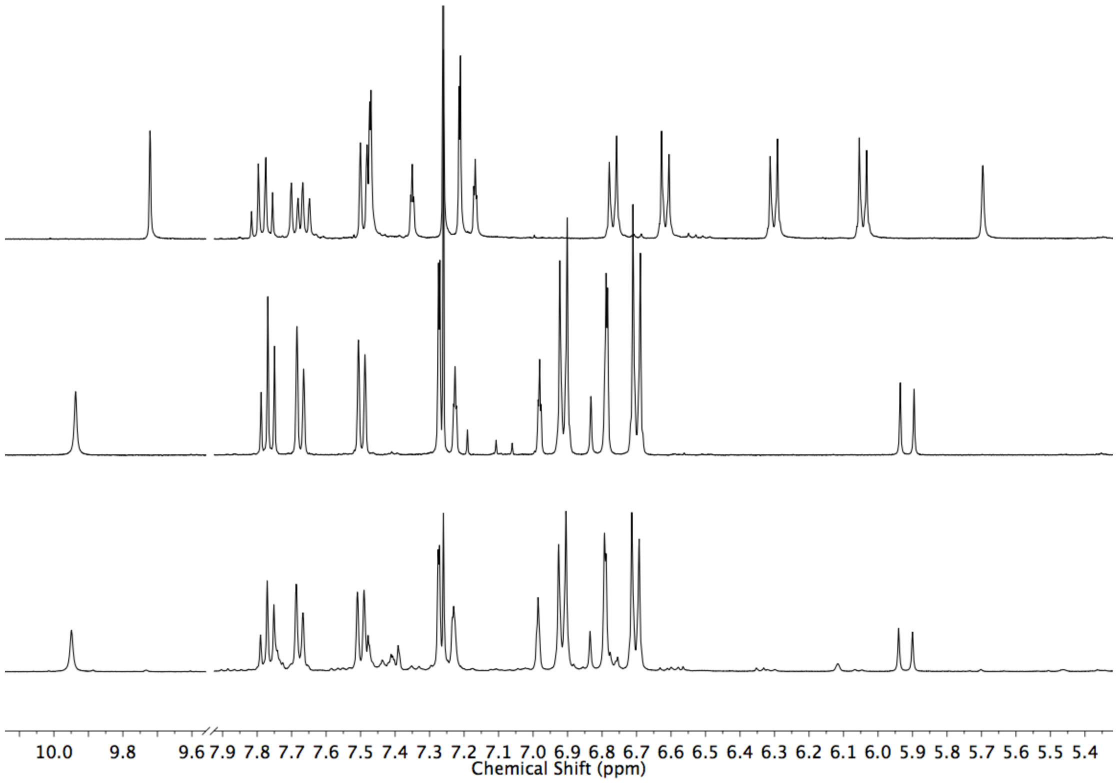

Figure S21 Stacked partial ^{1}H NMR (400 MHz, $CDCl_3$) spectra of **S4** (top), **6** (middle), the crude reaction product before chromatography (bottom). Ratio of **6** : **S4** = 100 : 0.

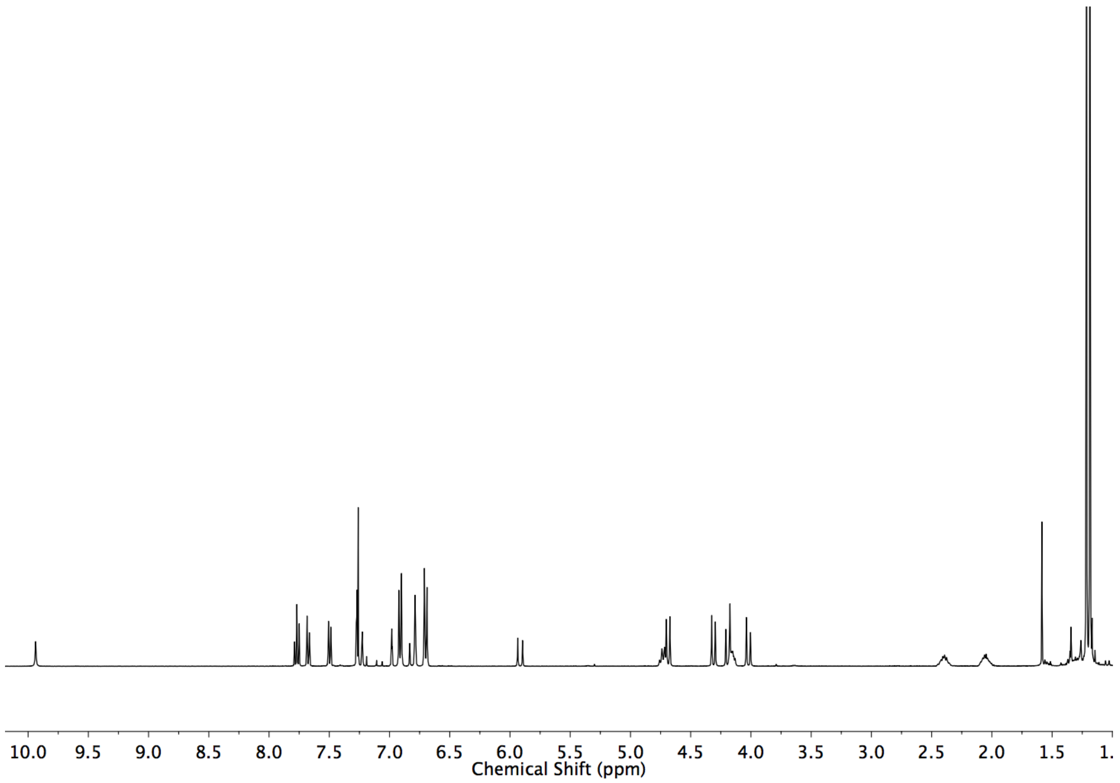

Figure S22 ^{1}H NMR ($CDCl_3$, 400 MHz) of **6**

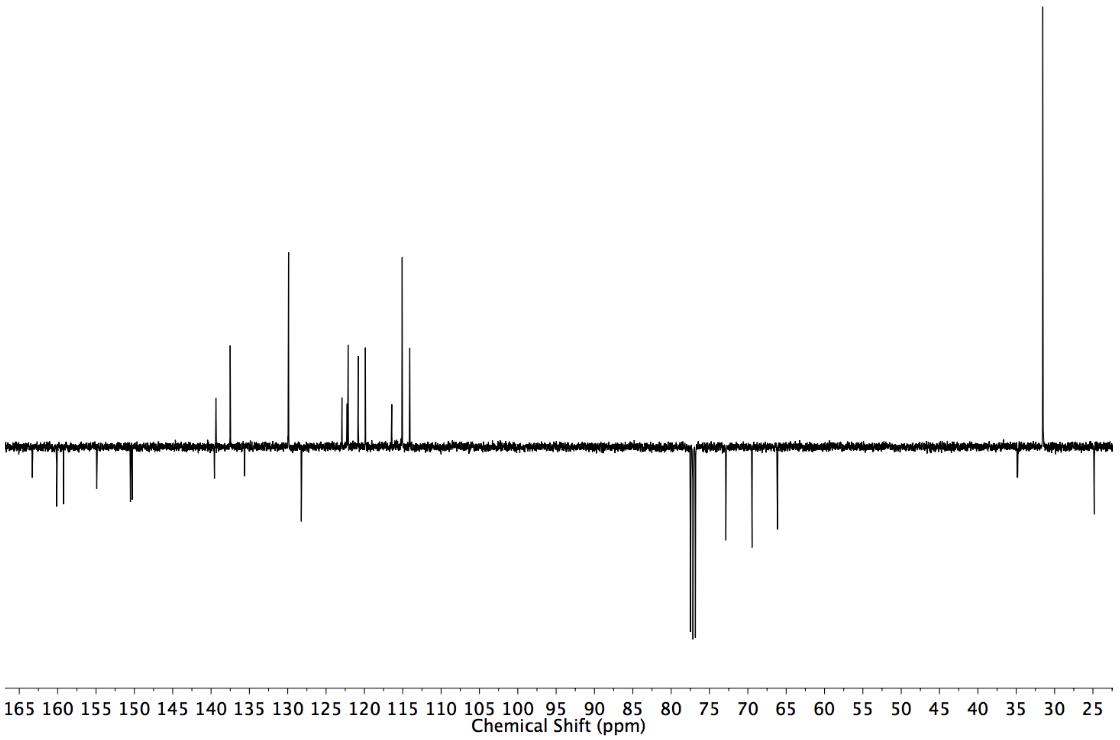

Figure S23 JMOD NMR ($CDCl_3$, 101 MHz) of **6**

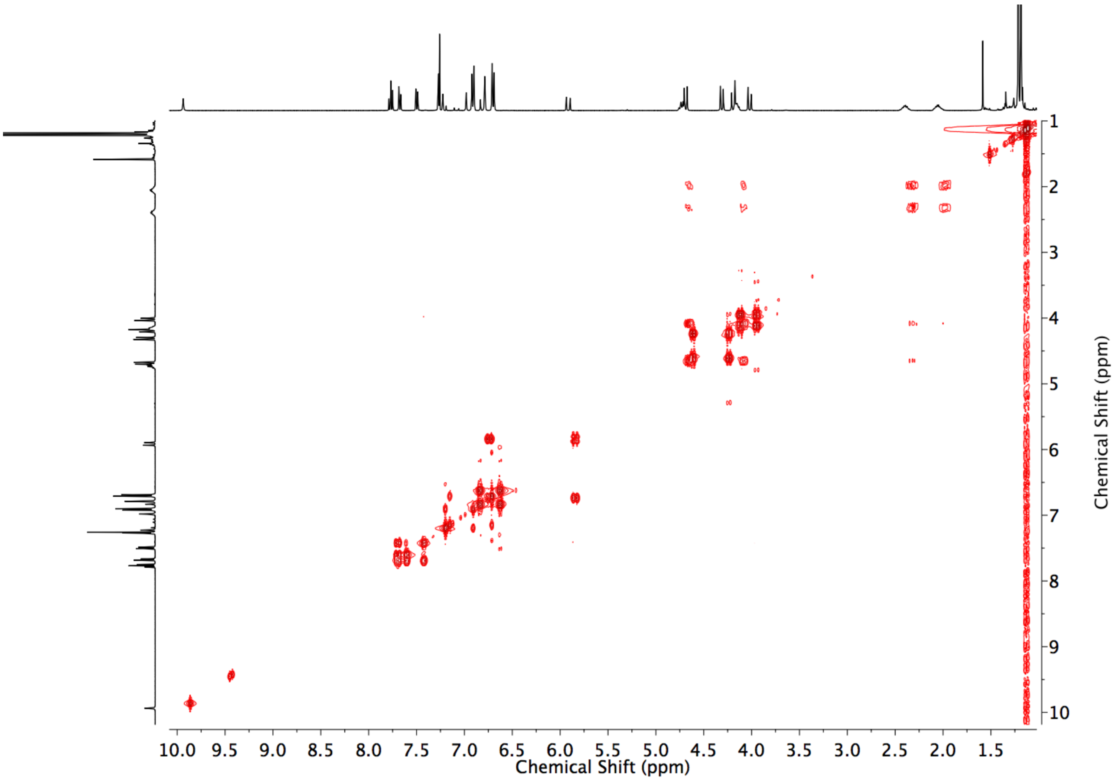

Figure S24 COSY NMR ($CDCl_3$) of **6**

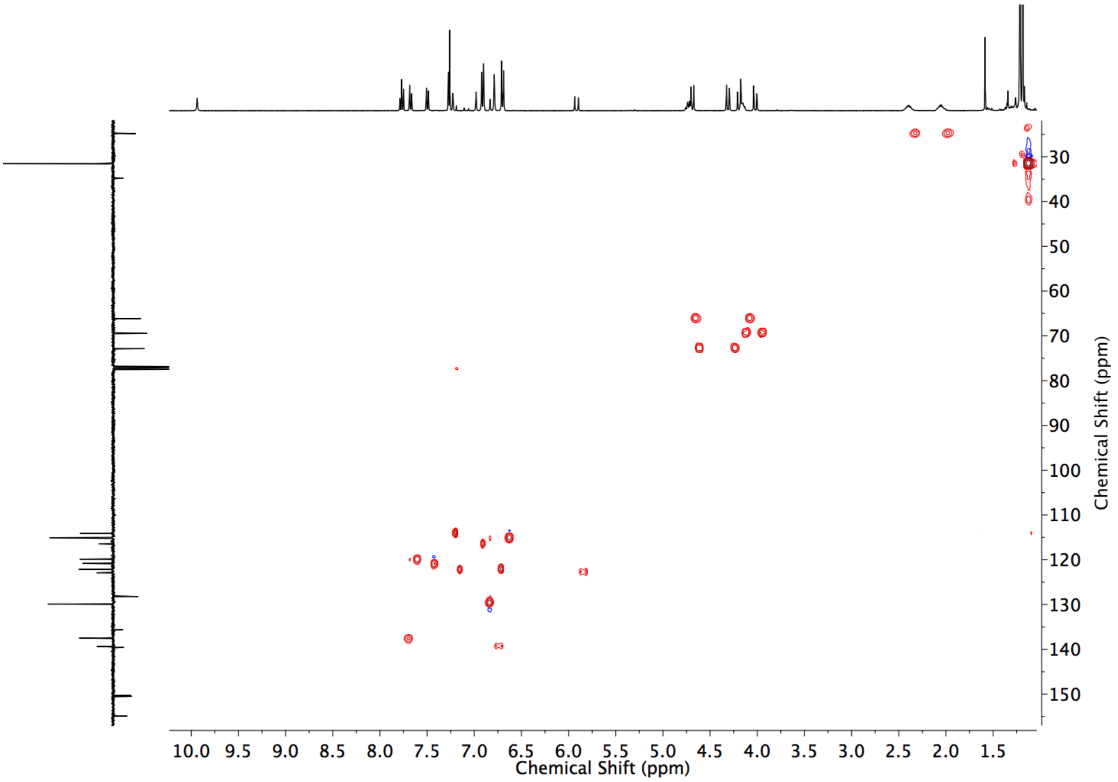

Figure S25 HSQC NMR ($CDCl_3$) of **6**

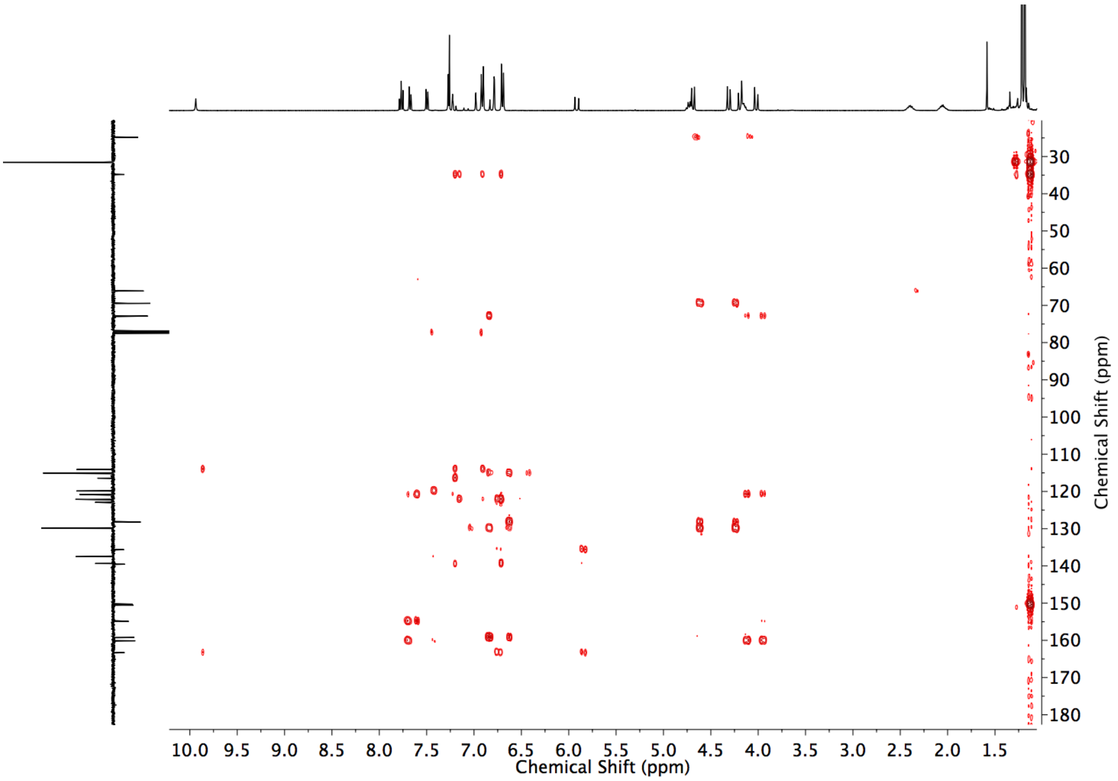

Figure S26 HMBC NMR ($CDCl_3$) of **6**

Rotaxane **7**

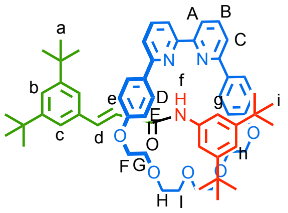

Prepared according to modified **general procedure A** (T = 150 °C, t = 2 h) with **1d** (12.5 mg, 0.025 mmol), $[Cu(MeCN)_4]PF_6$ (8.9 mg, 0.024 mmol), **2a** (6.9 mg, 0.03 mmol), and **3a** (7.3 mg, 0.03 mmol). Chromatography (petrol with a gradient of 0 to 50% Et_2O) gave **7** as a white foam (11.8 mg, 50%). ^{1}H NMR (500 MHz, $CDCl_3$) δ: 9.78 (s, 1H, H_f), 7.90 (t, t, *J* =7.8, 2H, H_B), 7.80 (dd, *J* =7.8, 0.9, 2H, H_A), 7.57 (dd, *J* =7.8, 0.9, 2H, H_C), 7.42 (d, *J* =8.5, 4H, H_D), 7.30 (t, *J* =1.8, 1H, H_b), 7.27 (d, *J* =16.0, H_d), 7.06 (d, *J* =1.8, 2H, Hc), 6.90 (apps, 3H, H_g, H_h), 6.63 (d, *J* =8.5, 4H, H_E), 6.46 (d, *J* =16.0, 1H, H_e), 4.26 – 4.14 (m, 4H, H_F), 3.96 – 3.64 (m, 12H, H_G, H_H, H_I), 1.33 (s, 18H, H_a), 1.12 (s, 18H, H_h). ^{13}C NMR (126 MHz, $CDCl_3$) δ: 163.6, 159.9, 158.9, 157.2, 150.6, 150.2, 138.7, 138.5, 137.7, 135.5, 132.4, 129.4, 124.3, 122.8, 122.1, 120.5, 119.6, 116.7, 115.1, 113.6, 70.3, 70.1, 68.3, 66.9, 34.9, 34.7, 31.5 (×2). HR-ESI-MS *m/z* = 946.5712 $[M+H]^+$ (calc. for $C_{61}H_{76}N_3O_6$ 946.5729).

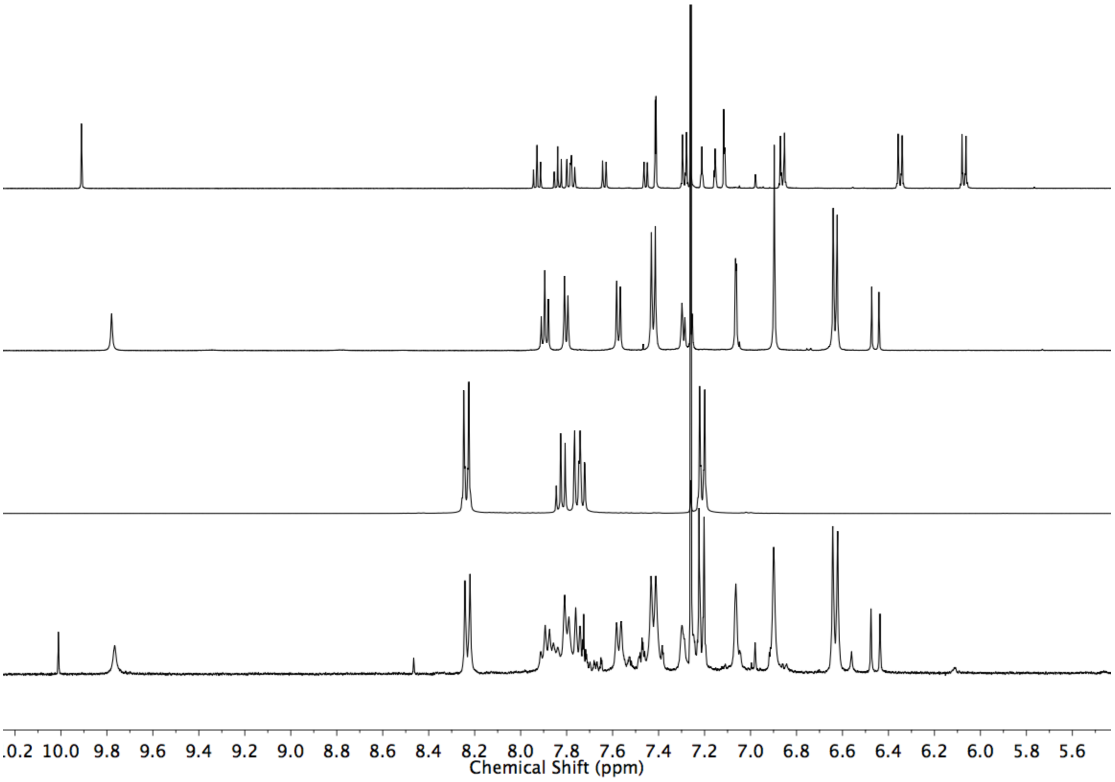

Figure S27 Stacked partial ^{1}H NMR (400 MHz, $CDCl_3$) spectra of **S5** (top), **7** (upper middle), macrocycle **1d** (lower middle) and the crude reaction product before chromatography (bottom). Ratio of **7** : oxidised product of **S5** = 80 : 20 (under these conditions the triazole derived rotaxane was observed to spontaneously oxidise to the ketone).

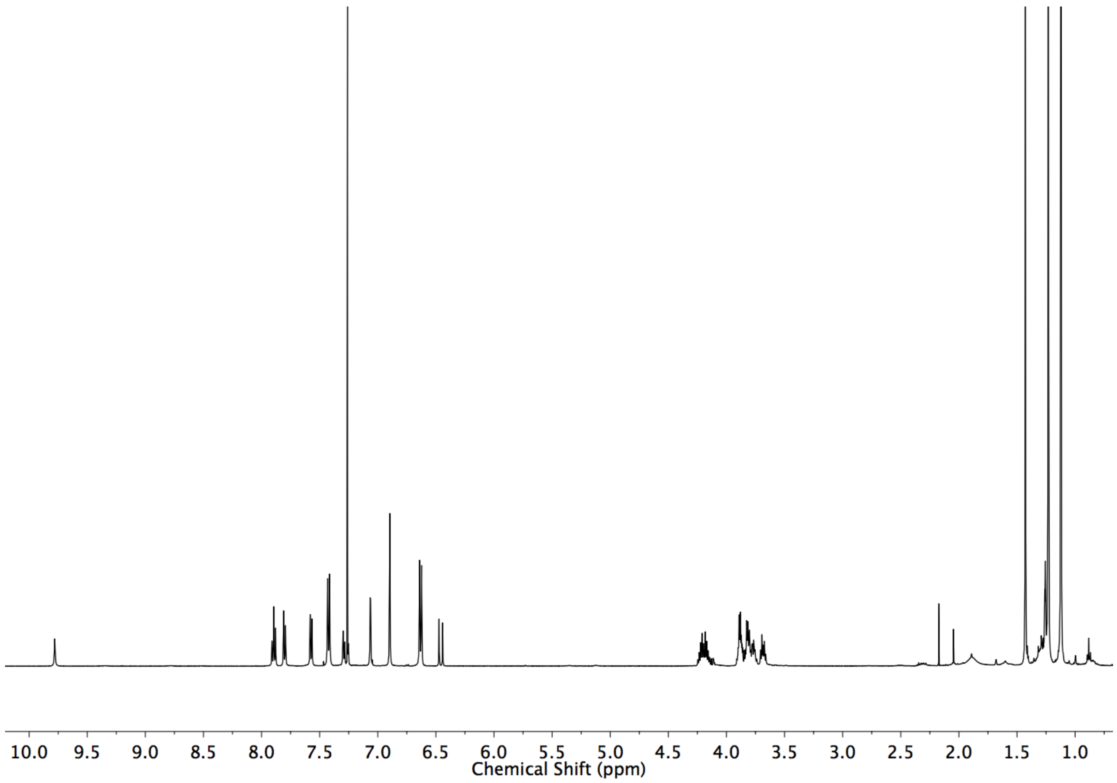

Figure S28 ^{1}H NMR ($CDCl_3$, 500 MHz) of **7**

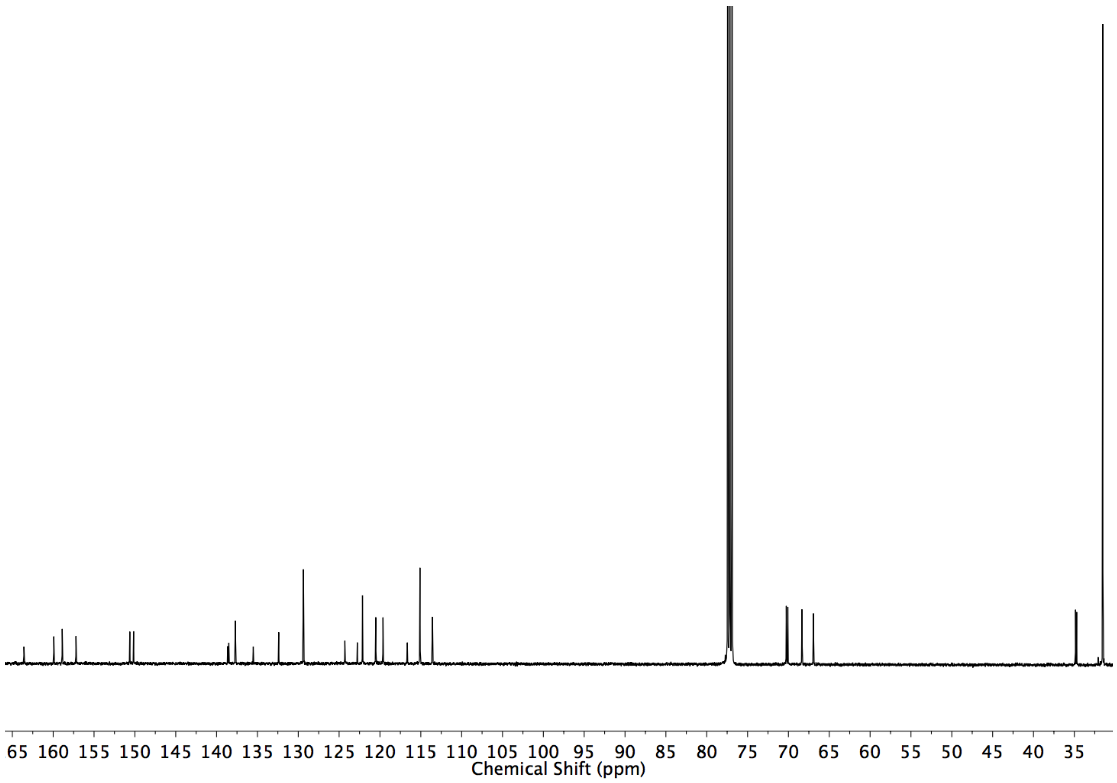

Figure S29 ^{13}C NMR ($CDCl_3$, 126 MHz) of **7**

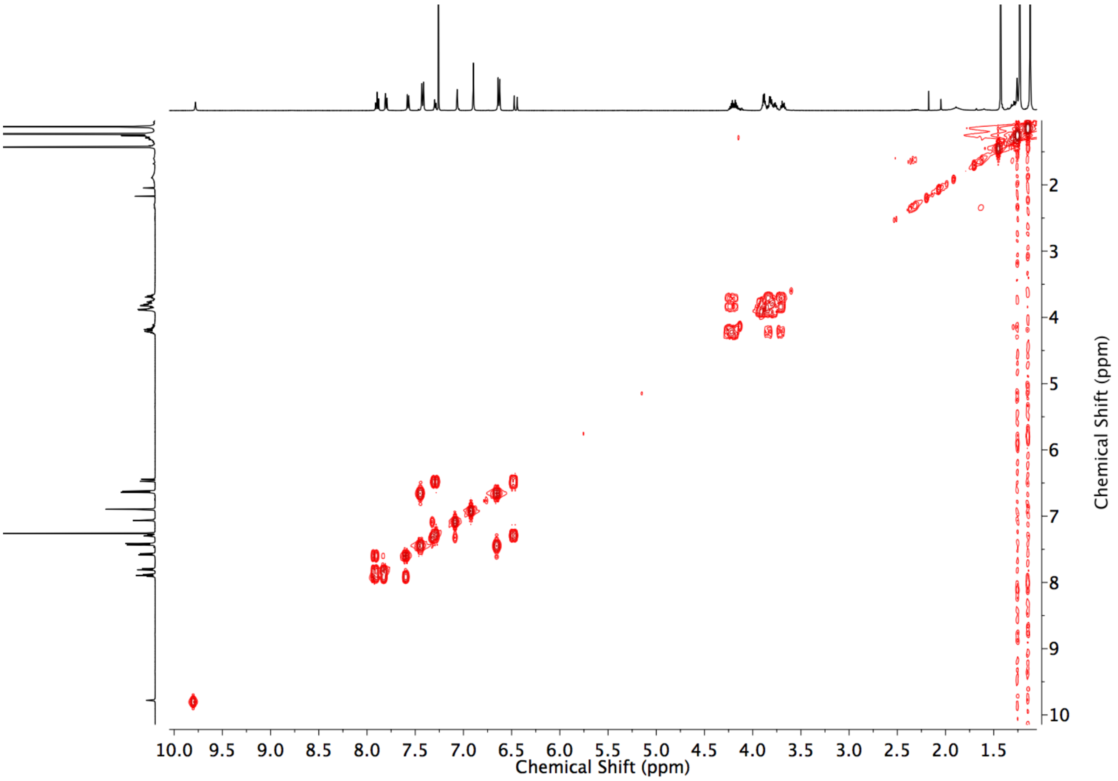

Figure S30 COSY NMR ($CDCl_3$) of **7**

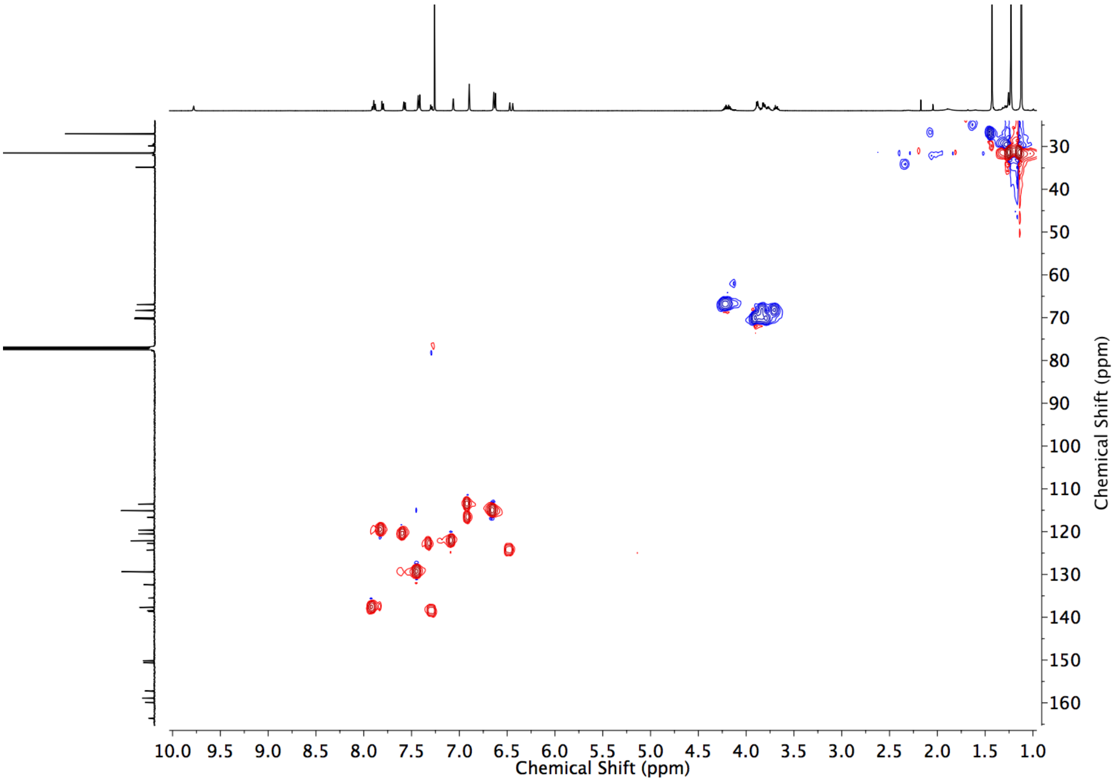

Figure S31 HSQC NMR ($CDCl_3$) of **7**

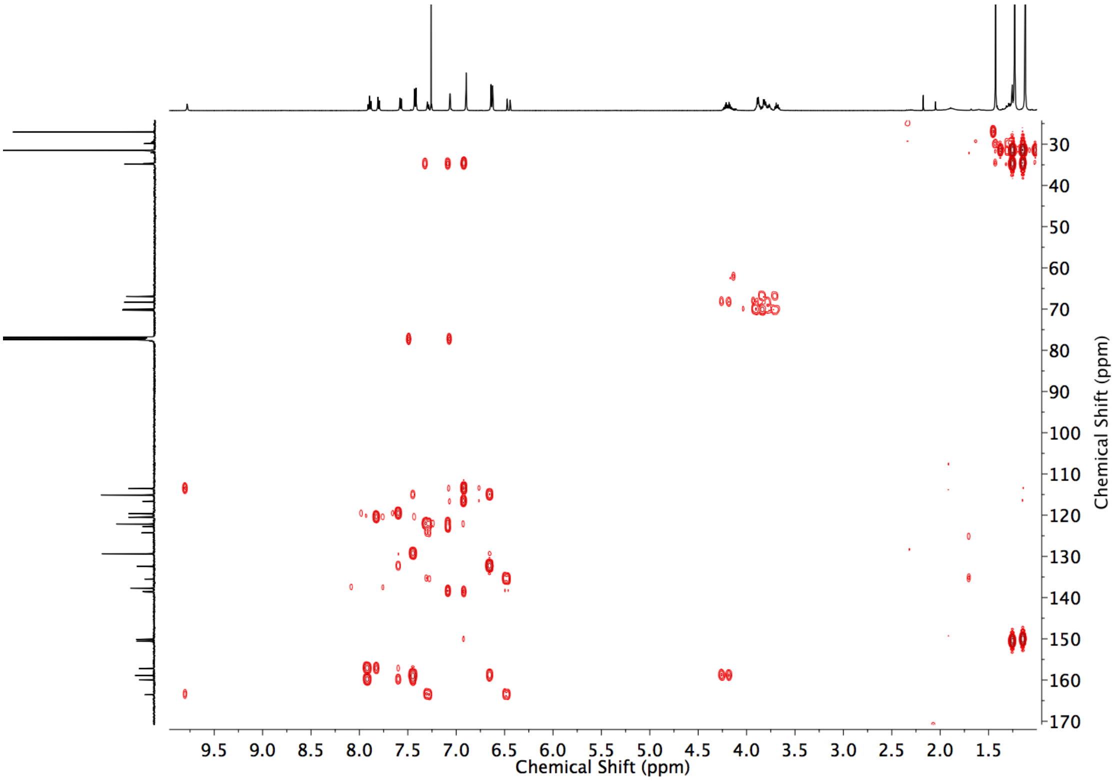

Figure S32 HMBC NMR ($CDCl_3$) of **7**

Rotaxane **8**

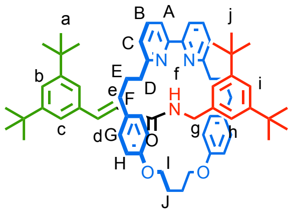

Prepared according to **general procedure A** with **1a** (12.0 mg, 0.025 mmol), $[Cu(MeCN)_4]PF_6$ (8.9 mg, 0.024 mmol), **2b** (7.3 mg, 0.03 mmol), and **3a** (7.3 mg, 0.03 mmol). Chromatography (petrol with a gradient of 0 to 20% Et_2O) gave **8** as a white foam (16.0 mg, 68 %). ^{1}H NMR (500 MHz, $CDCl_3$) δ: 8.77 (t, *J* =4.8, 1H, H_f), 7.60 (t, *J* =7.8, 2H, H_B), 7.44 (dd, *J* =7.8, 1.0, 2H, H_A), 7.21 (t, *J* =1.8, 1H, H_b), 7.19 (d, *J* =16.0, 1H, H_d), 7.07 (dd, *J* =7.8, 1.0, 2H, H_C), 7.04 (j, *J* =1.9, 1H, H_i), 6.88 (d, *J* =1.9, 2H, H_c), 6.79 (s, 8H, H_G, H_H), 6.77 (d, *J* =1.8, 2H, H_h), 6.35 (d, *J* =16.0, 1H, H_e), 4.33 – 4.23 (m, 4H, H_I), 3.41 (d, *J* =4.8, 2H, H_g), 2.61 – 2.47 (m, 4H, H_F), 2.46 – 2.34 (m, 4H, H_D), 2.17 – 2.10 (m, 4H, H_J), 1.83 – 1.70 (m, 4H, H_E), 1.16 (s, 18H, H_a), 1.06 (s, 18H, H_j). ^{13}C NMR (126 MHz, $CDCl_3$) δ: 165.4, 163.6, 157.3, 156.5, 150.4, 149.8, 137.6, 137.2, 136.9, 135.7, 132.6, 129.4, 123.2, 122.9, 122.3, 121.9, 121.9, 120.5, 119.5, 115.2, 66.6, 44.2, 36.5, 35.2, 34.8, 34.7, 31.5, 31.4, 31.4, 25.0; HR-ESI-MS *m/z* = 940.6351 $[M+H]^+$ (calc. for $C_{64}H_{82}N_3O_3$ 940.6351).

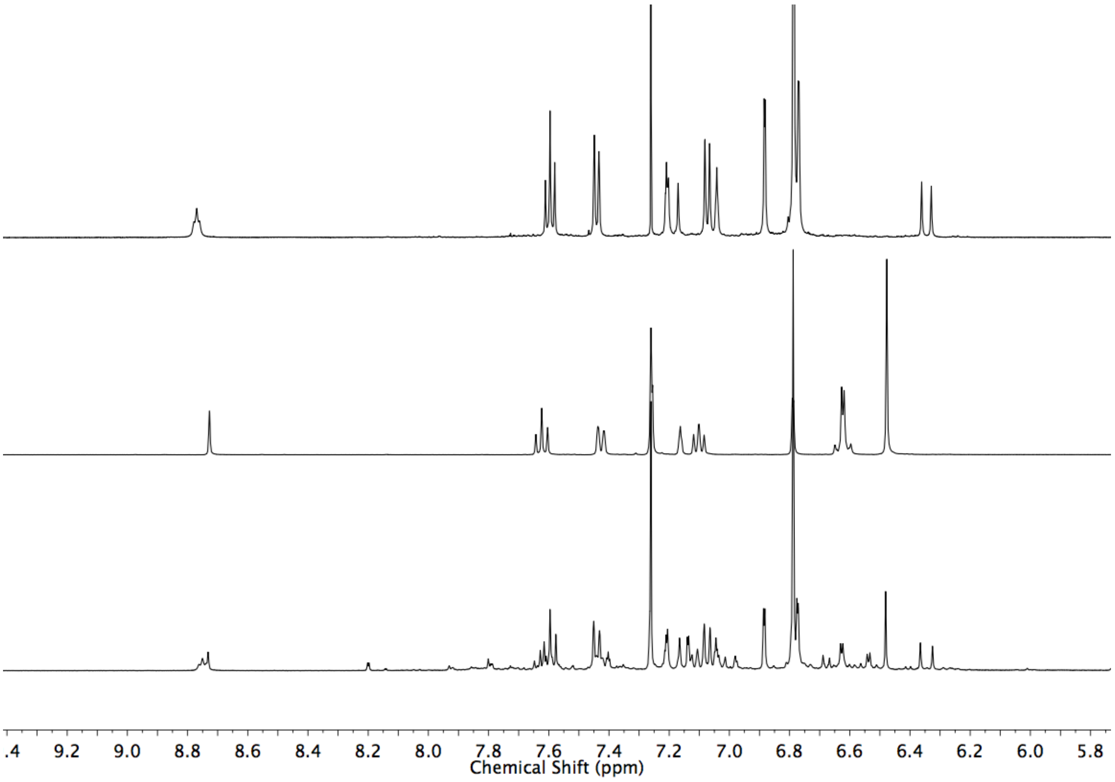

Figure S33 Stacked partial ^{1}H NMR (400 MHz, $CDCl_3$) spectra of **8** (top), **S6** (middle) and the crude reaction product before chromatography (bottom). Ratio of **8** : **S6** = 75 : 25.

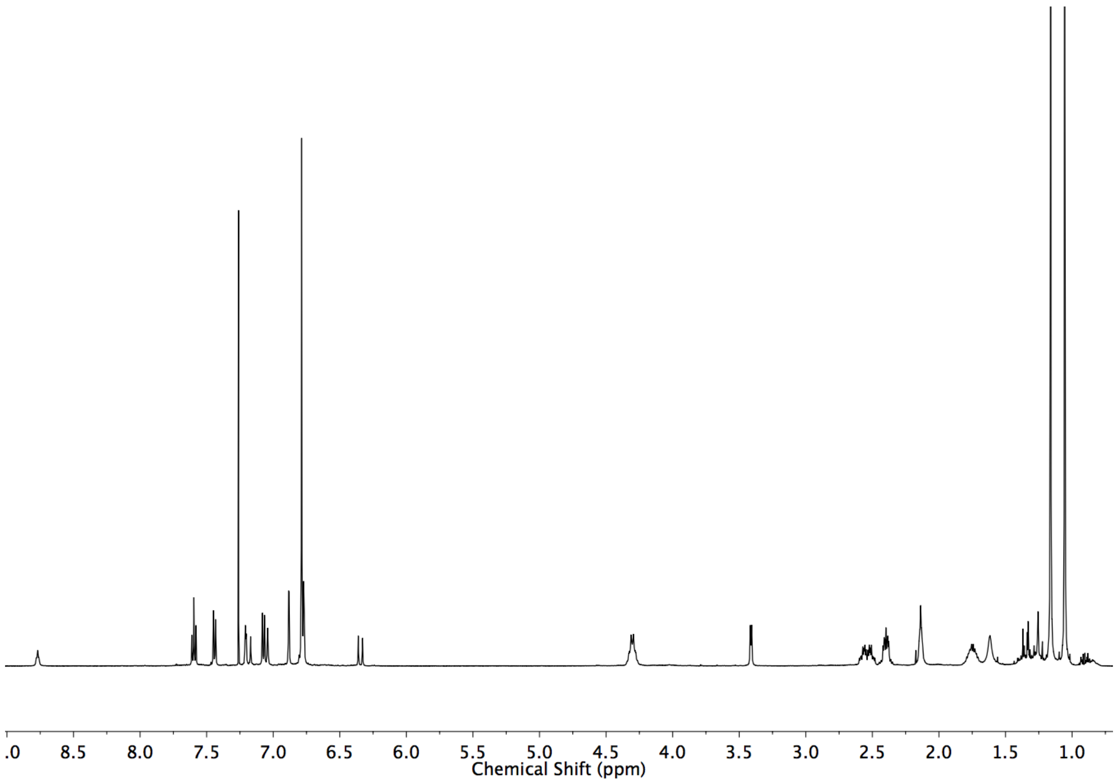

Figure S34 ^{1}H NMR ($CDCl_3$, 500 MHz) of **8**

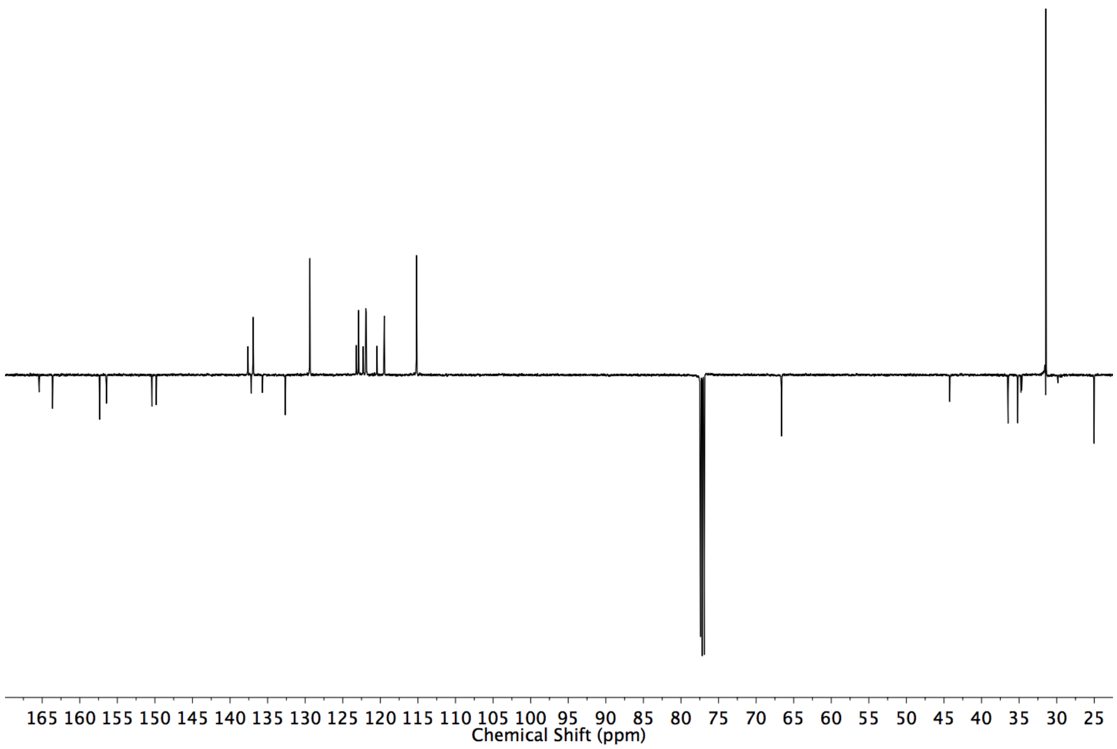

Figure S35 JMOD NMR ($CDCl_3$, 126 MHz) of **8**

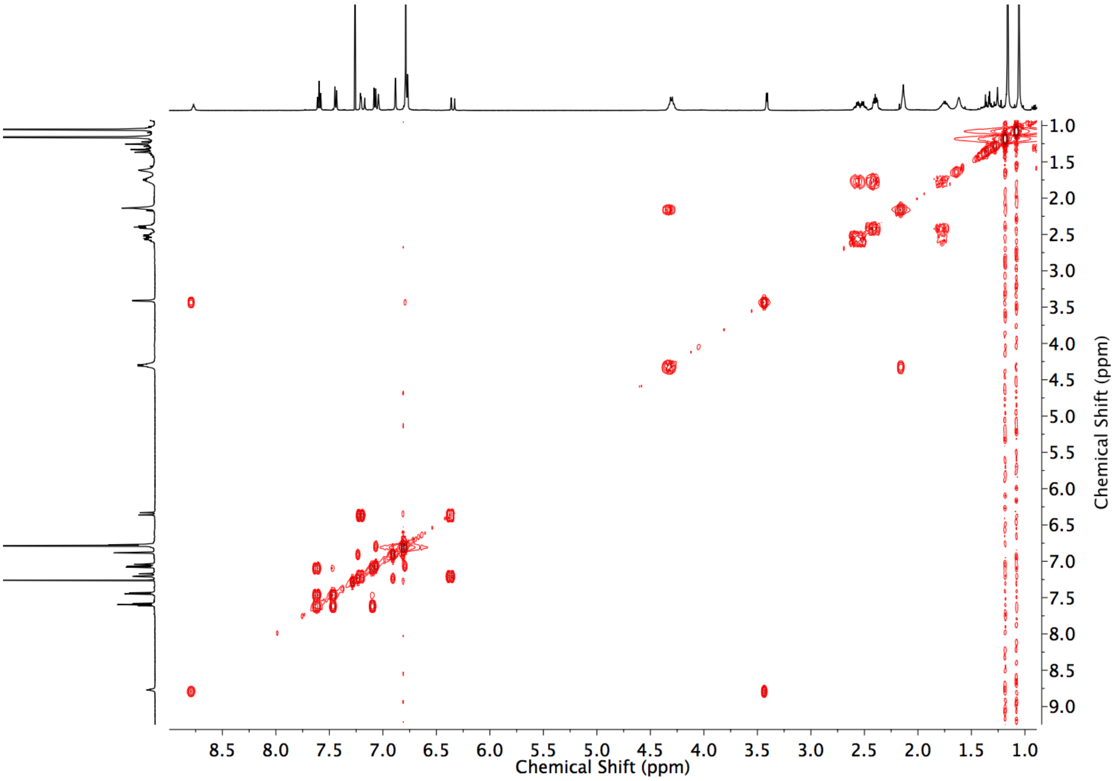

Figure S36 COSY NMR ($CDCl_3$) of **8**

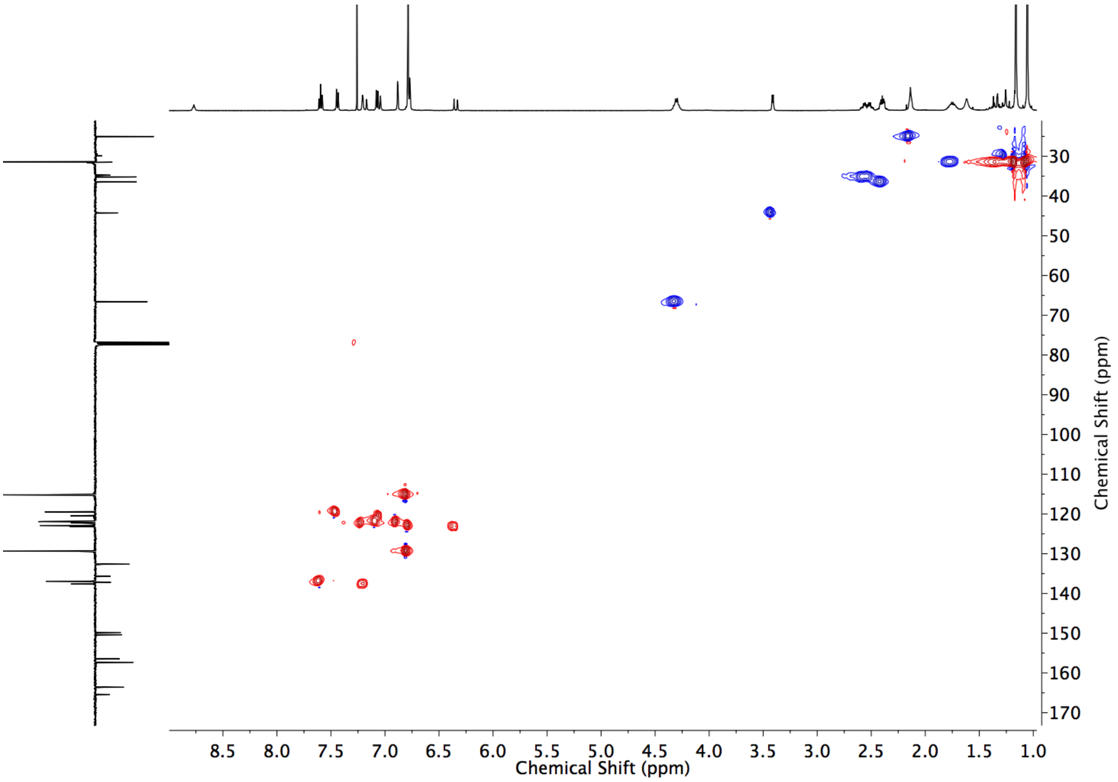

Figure S37 HSQC NMR ($CDCl_3$) of **8**

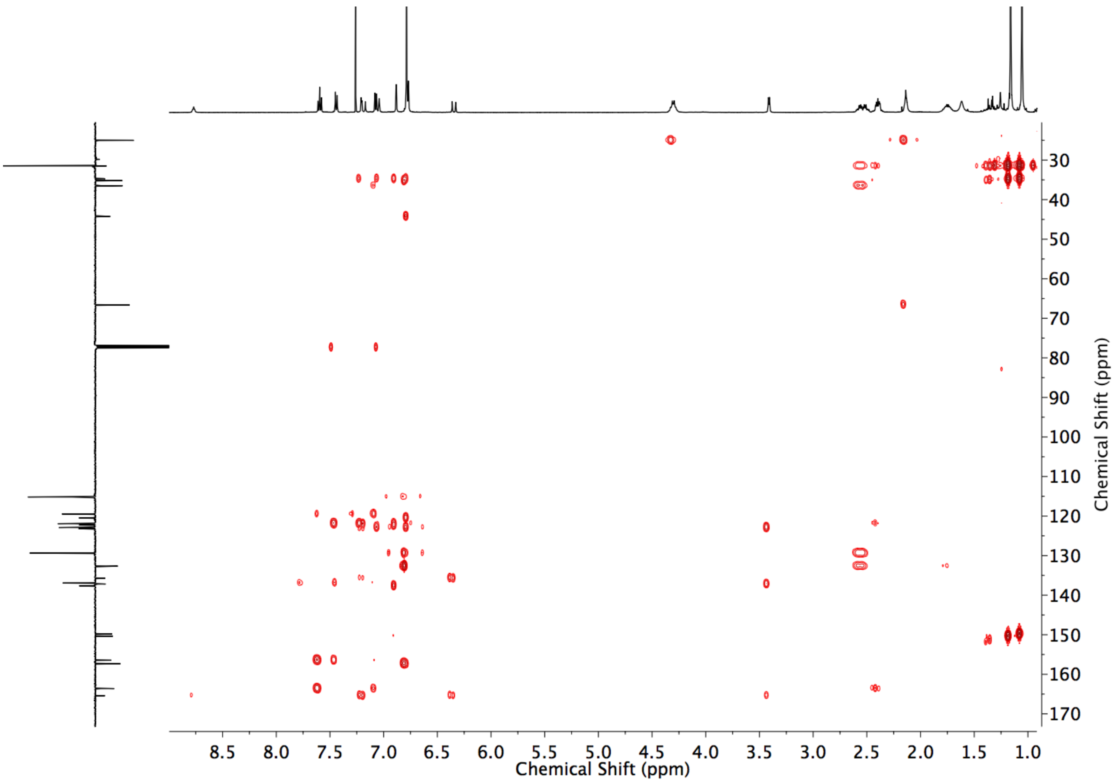

Figure S38 HMBC NMR ($CDCl_3$) of **8**

Rotaxane **9**

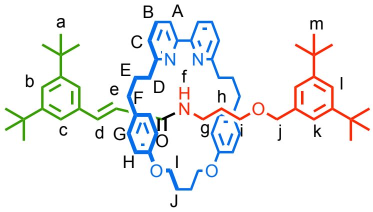

Prepared according to **general procedure A** with **1a** (12.0 mg, 0.025 mmol), $[Cu(MeCN)_4]PF_6$ (8.9 mg, 0.024 mmol), **2c** (9.1 mg, 0.03 mmol), and **3a** (7.3 mg, 0.03 mmol). Chromatography (petrol with a gradient of 0 to 50% Et_2O) gave **9** as a colourless oil (4.0 mg, 16 %). ^1H NMR (500 MHz, $CDCl_3$) δ: 8.43 (t, *J* =5.4, 1H, H_f), 7.65 (t, *J* =7.8, 2H, H_B), 7.48 (dd, *J* =7.8, 0.9, 2H, H_A), 7.31 (t, *J* =1.9, 1H, H_l), 7.27 (d, *J* =16.0, 1H, H_d), 7.23 (t, *J* =1.9, 1H, H_b), 7.18 (dd, *J* =7.8, 0.9, 2H, H_C), 7.02 (d, *J* =1.9, 2H, H_k), 6.92 (d, *J* =1.9, 2H, H_c), 6.90 (d, *J* =8.5, 4H, H_G), 6.82 (d, *J* =8.5, 4H, H_F), 6.32 (d, *J* =16.0, 1H, H_e), 4.29 – 4.13 (m, 4H, H_I), 4.08 (s, 2H, H_j), 3.02 (t, *J* =7.2, 2H, H_i), 2.70 – 2.53 (m, 8H, H_D, H_F), 2.29 – 2.23 (m, 2H, H_g), 2.18 – 1.99 (m, 4H, H_J), 1.97 – 1.75 (m, 4H, H_E), 1.30 (s, 18H, H_a), 1.16 (s, 18H, H_m), 0.97 (t, *J* =7.8, 2H, H_h). ^{13}C NMR (126 MHz, $CDCl_3$) δ: 165.4, 163.5, 157.5, 156.6, 150.7, 150.6, 137.9, 137.8, 137.1, 135.5, 132.8, 129.6, 123.2, 122.6, 122.1, 121.9, 121.9, 121.7, 119.8, 115.1, 73.5, 68.8, 66.5, 36.6, 35.5, 35.3, 34.9, 34.8, 31.6, 31.5, 31.4, 28.8, 25.0. HR-ESI-MS *m/z* = 998.6749 $[M+H]^+$ (calc. for $C_{64}H_{82}N_3O_3$ 998.6769).

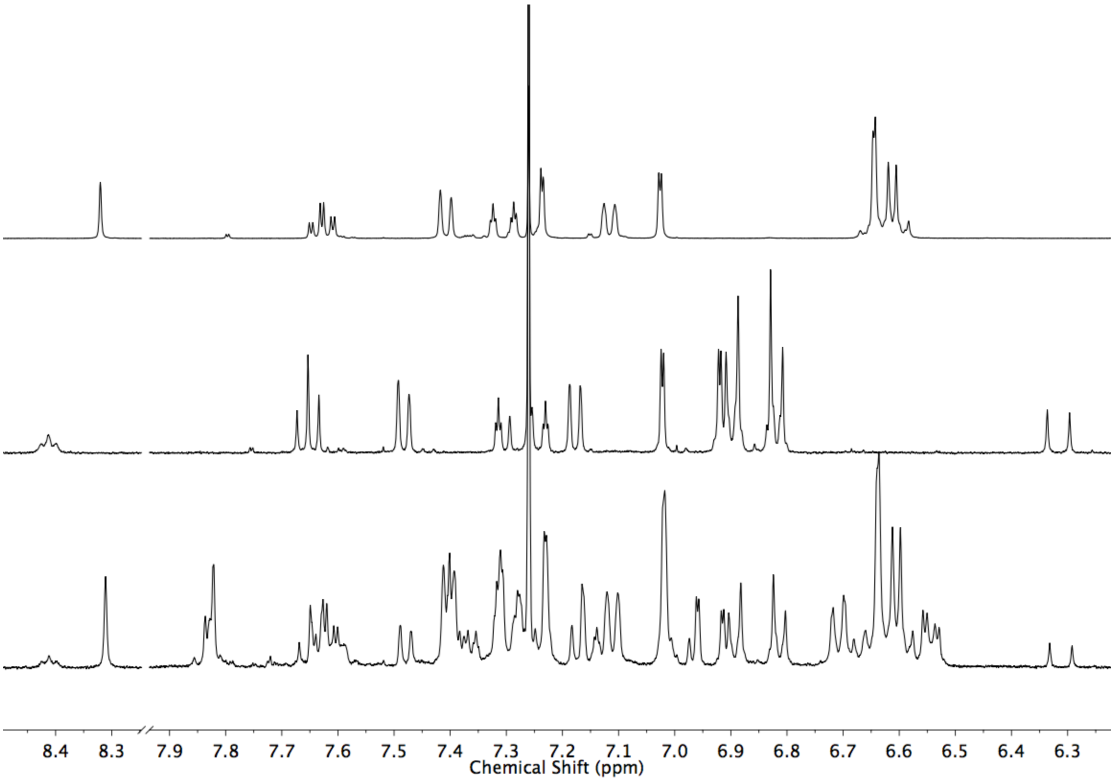

Figure S39 Stacked partial ^1H NMR (400 MHz, $CDCl_3$) spectra of **S7** (top), **9** (middle) and the crude reaction product before chromatography (bottom). Ratio of **9** : **S7** = 35 : 65.

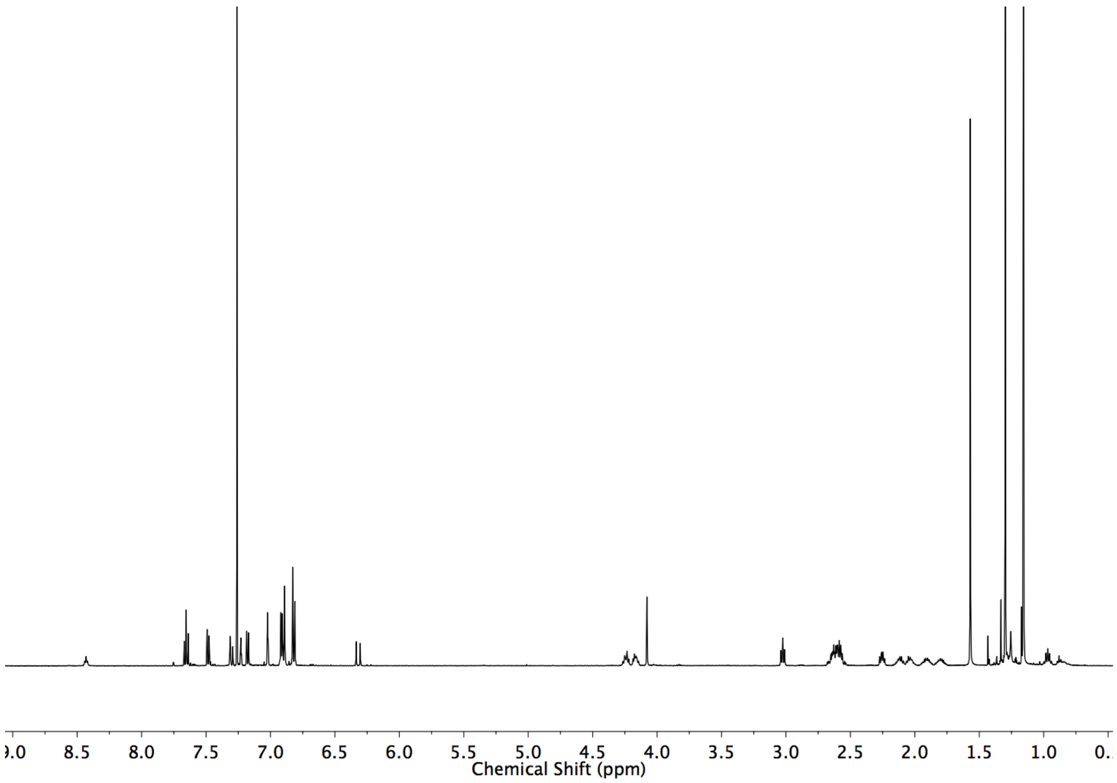

Figure S40 ^1H NMR ($CDCl_3$, 500 MHz) of **9**

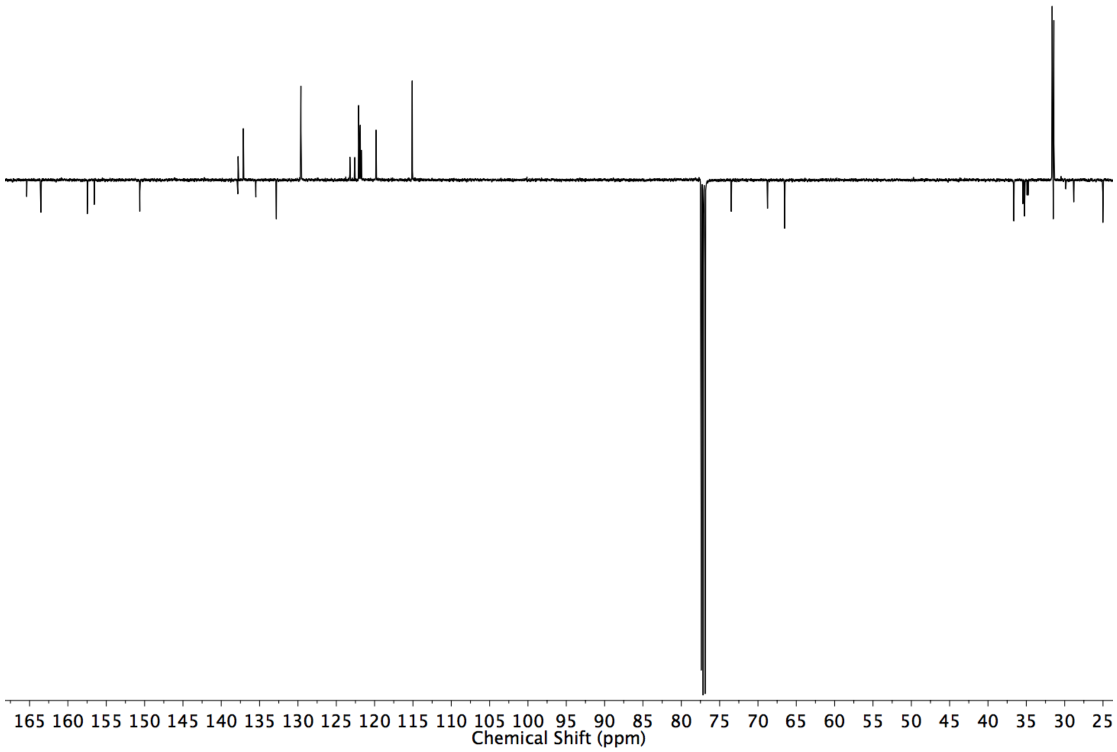

Figure S41 JMOD NMR ($CDCl_3$, 126 MHz) of **9**

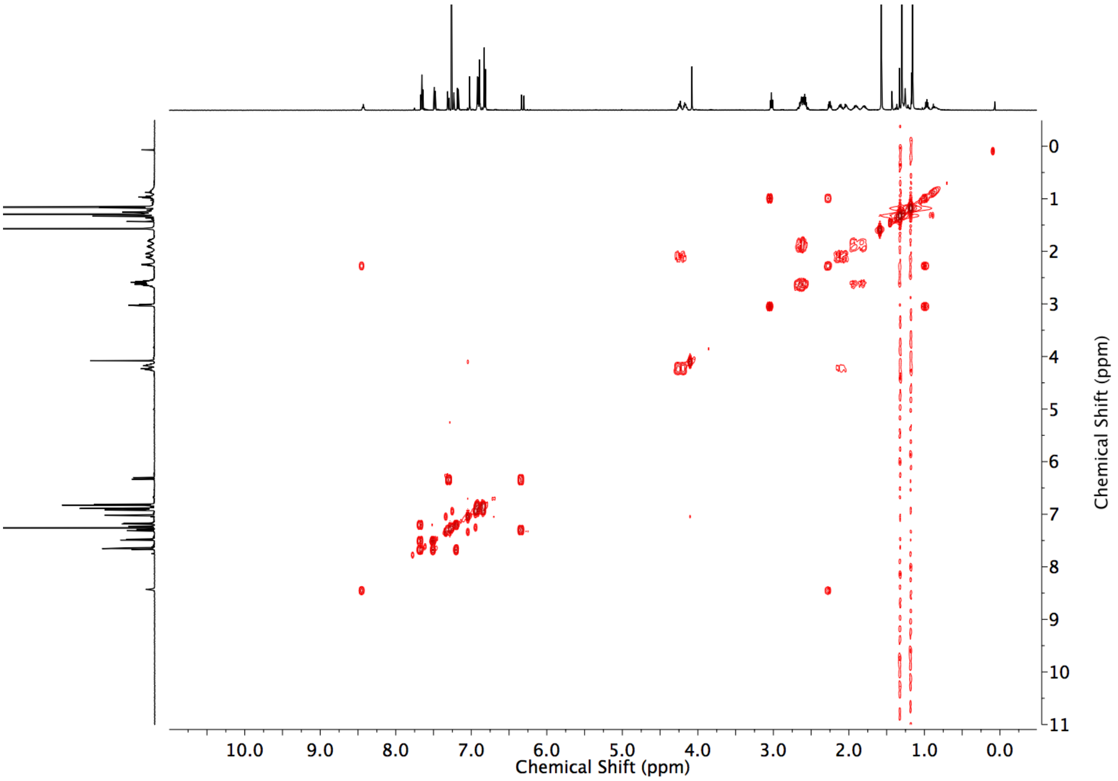

Figure S42 COSY NMR ($CDCl_3$) of **9**

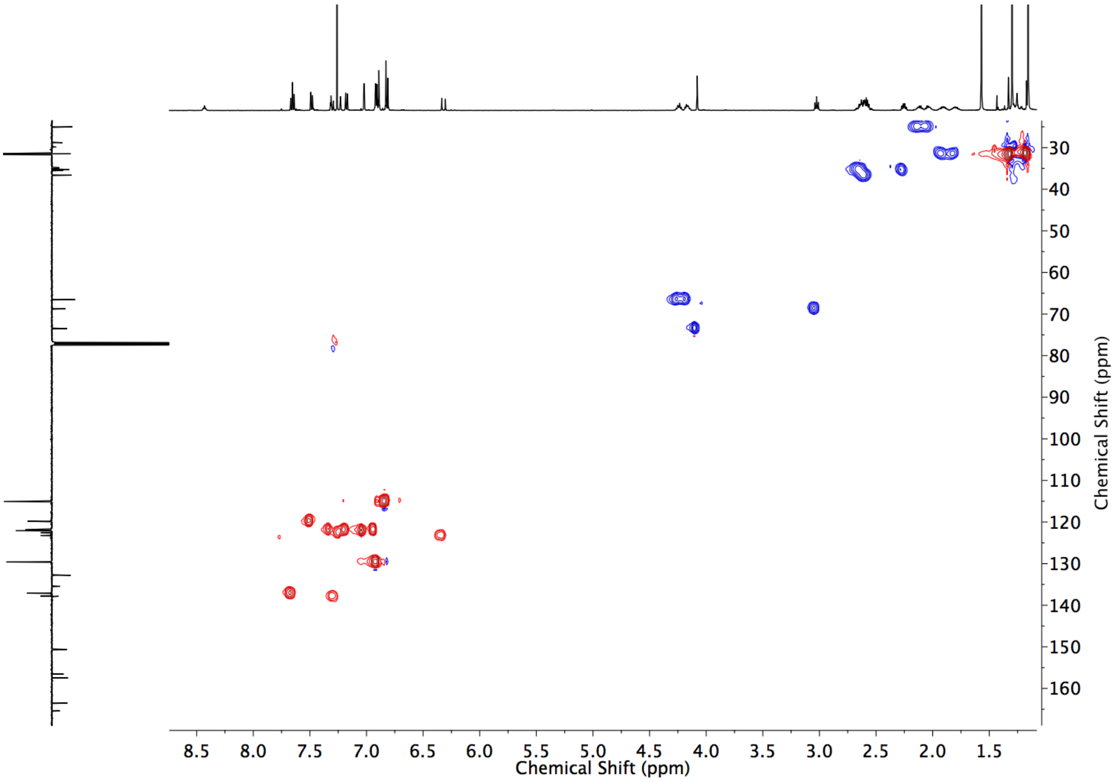

Figure S43 HSQC NMR ($CDCl_3$) of **9**

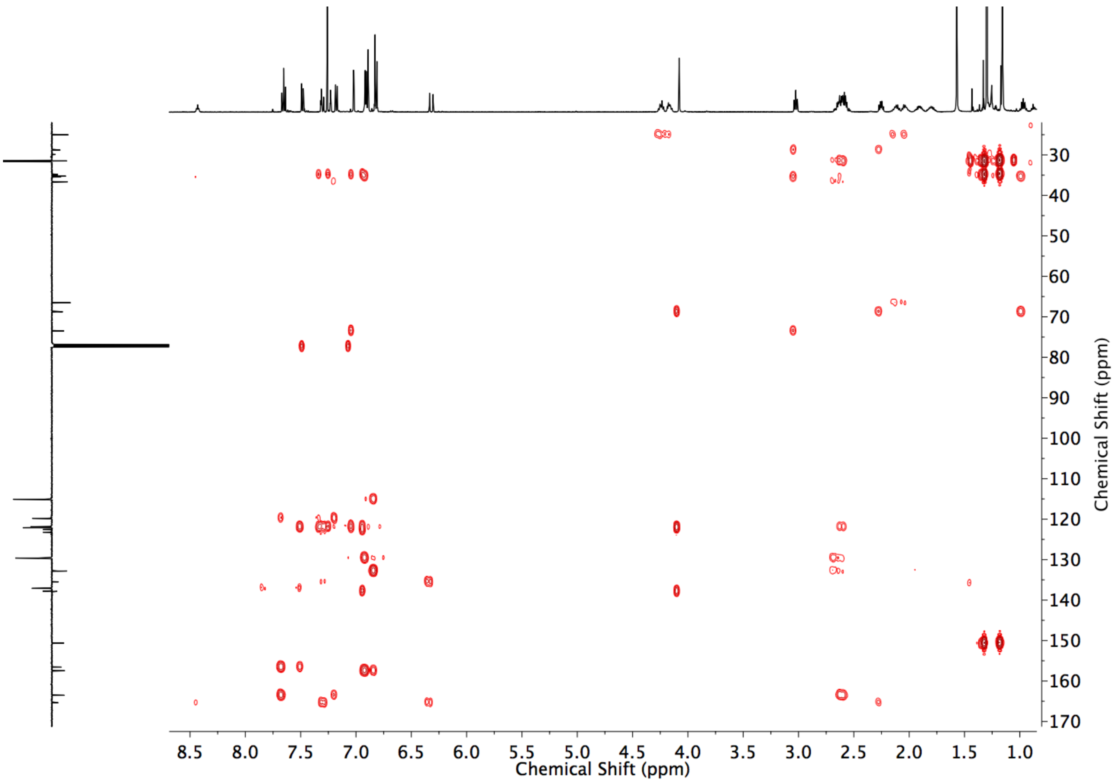

Figure S44 HMBC NMR ($CDCl_3$) of **9**

Rotaxane **10**

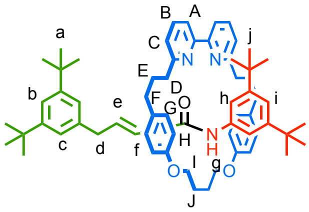

Prepared according to **general procedure A** with **1a** (12.0 mg, 0.025 mmol), $[Cu(MeCN)_4]PF_6$ (8.9 mg, 0.024 mmol), **2a** (6.9 mg, 0.03 mmol), and **3b** (7.8 mg, 0.03 mmol). Chromatography (petrol with a gradient of 0 to 20% Et_2O) gave **10** as a colourless oil (10.0 mg, 43%). ^1H NMR (400 MHz, $CDCl_3$) δ: 9.92 (s, 1H, H_g), 7.62 (t, *J* =7.9, 2H, H_B), 7.48 (dd, *J* =7.9, 0.9, 2H, H_A), 7.15 (t, *J* =1.9, 1H, H_b), 7.10 (dd, *J* =7.9, 0.9, 2H, H_C), 6.97 (d, *J* =1.9, 2H, H_h), 6.85 (t, *J* =1.9, 1H, H_i), 6.82 (d, *J* =1.9, 2H, H_c), 6.77 (d, *J* =8.5, 4H, H_G), 6.70 (d, *J* =8.5, 4H, H_H), 6.42 (dt, *J* =15.5, 6.8, 1H, H_e), 5.46 (dt, *J* =15.5, 1.6, 1H, H_f), 4.50 – 4.38 (m, 2H, 2 of H_I), 4.17 – 4.06 (m, 2H, 2 of H_I), 2.93 (d, *J* =6.8, 2H, H_d), 2.63 – 2.41 (m, 8H, H_D, H_F), 2.29 – 2.16 (m, 2H, 2 of H_J), 2.07 – 1.95 (m, 2H, 2 of H_J), 1.90 – 1.67 (m, 4H, H_E), 1.21 (s, 18H, H_a or H_j), 1.10 (s, 18H, H_a or H_j). ^{13}C NMR (101 MHz, $CDCl_3$) δ: 163.6, 163.3, 157.6, 156.3, 150.4, 150.0, 141.3, 139.4, 138.5, 137.0, 132.6, 129.2, 125.6, 122.9, 121.9, 120.2, 119.6, 116.0, 115.1, 114.3, 66.5, 39.4, 36.6, 35.2, 34.8 (×2), 31.6, 31.5, 31.1, 25.2. HR-ESI-MS *m/z* = 940.6367 $[M+H]^+$ (calc. for $C_{64}H_{82}N_3O_3$ 940.6351).

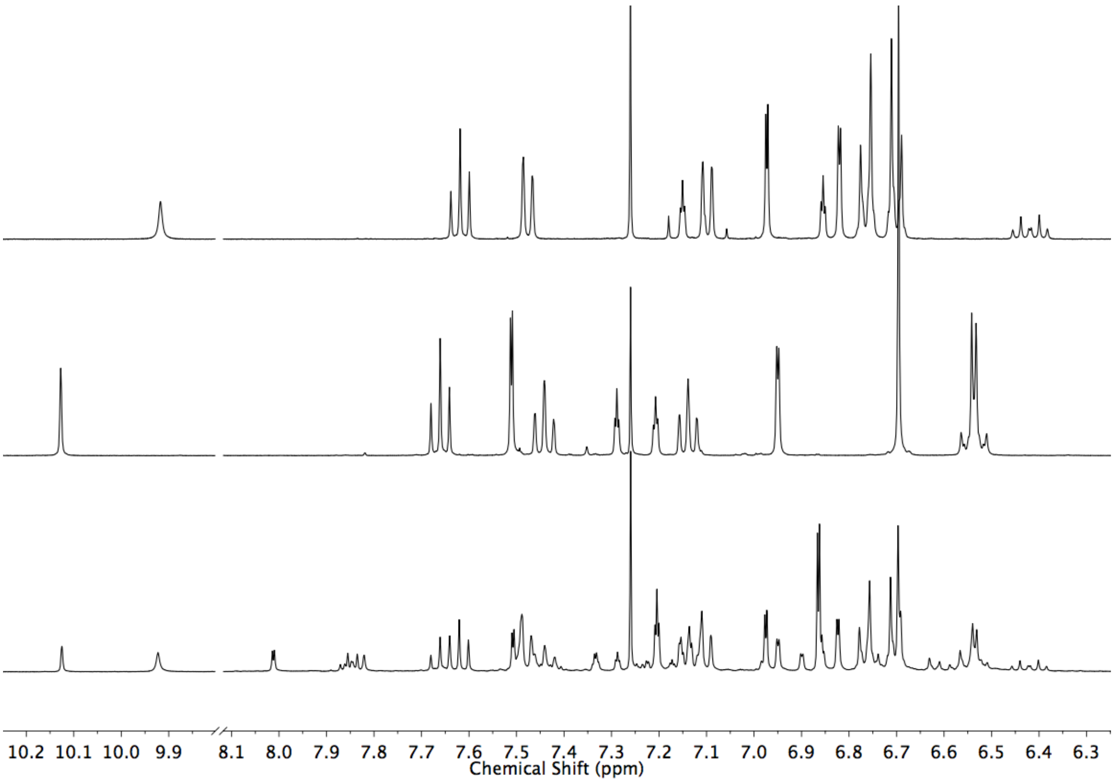

Figure S45 Stacked partial ^1H NMR (400 MHz, $CDCl_3$) spectra of **10** (top), **S8** (middle) and the crude reaction product before chromatography (bottom). Ratio of **10** : **S8** = 60 : 40.

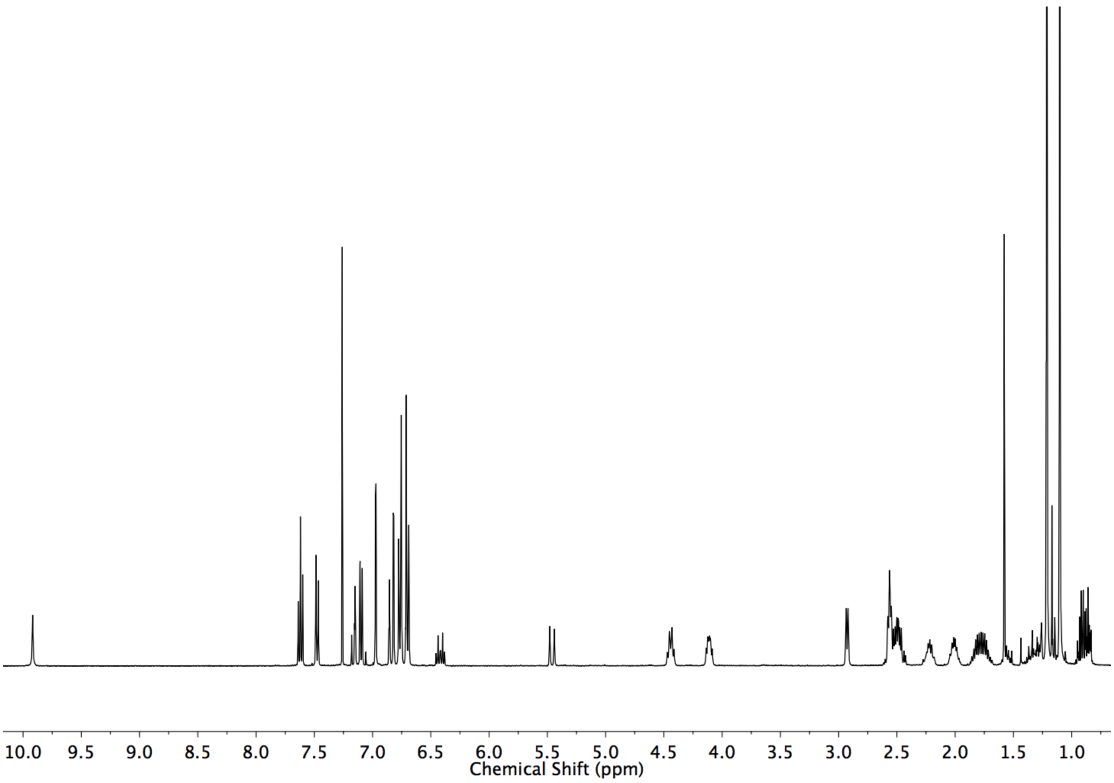

Figure S46 ^{1}H NMR ($CDCl_3$, 400 MHz) of **10**

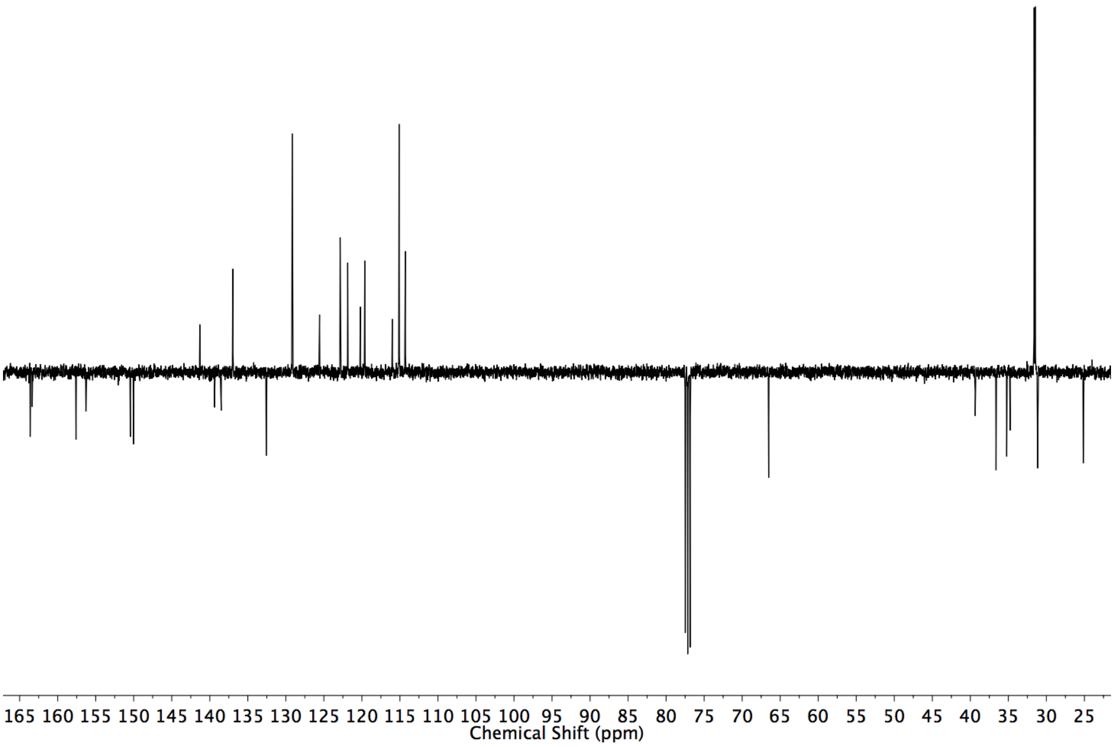

Figure S47 JMOD NMR ($CDCl_3$, 101 MHz) of **10**

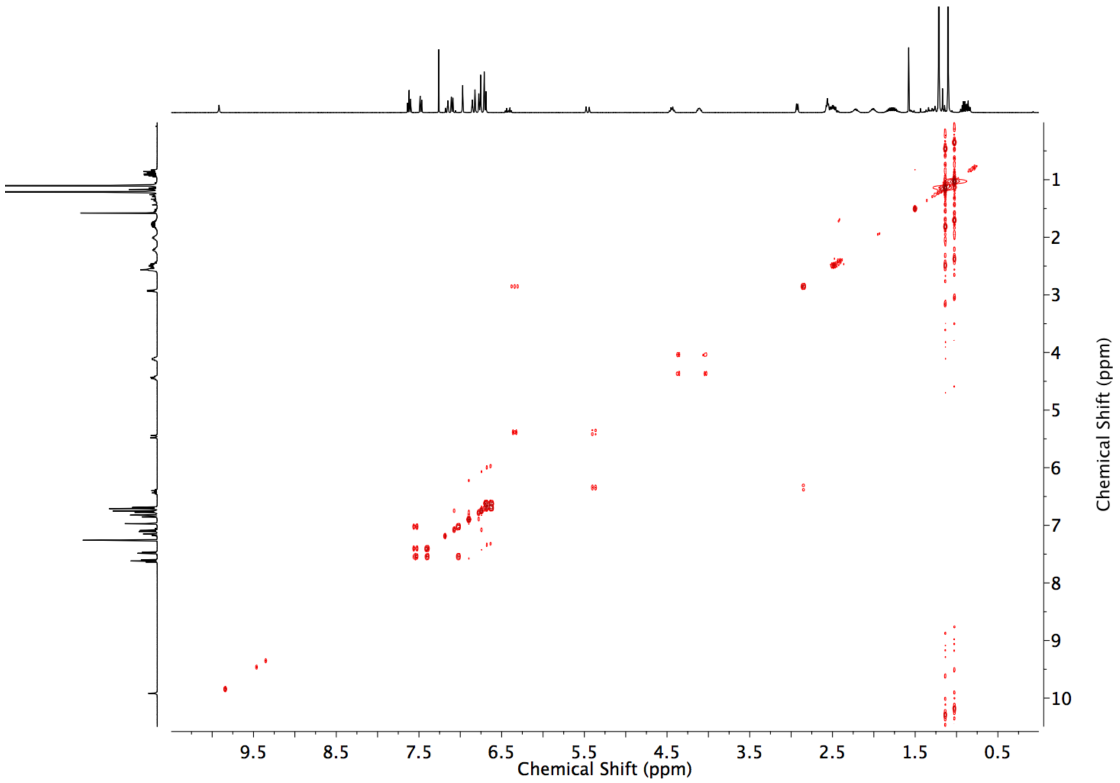

Figure S48 COSY NMR ($CDCl_3$) of **10**

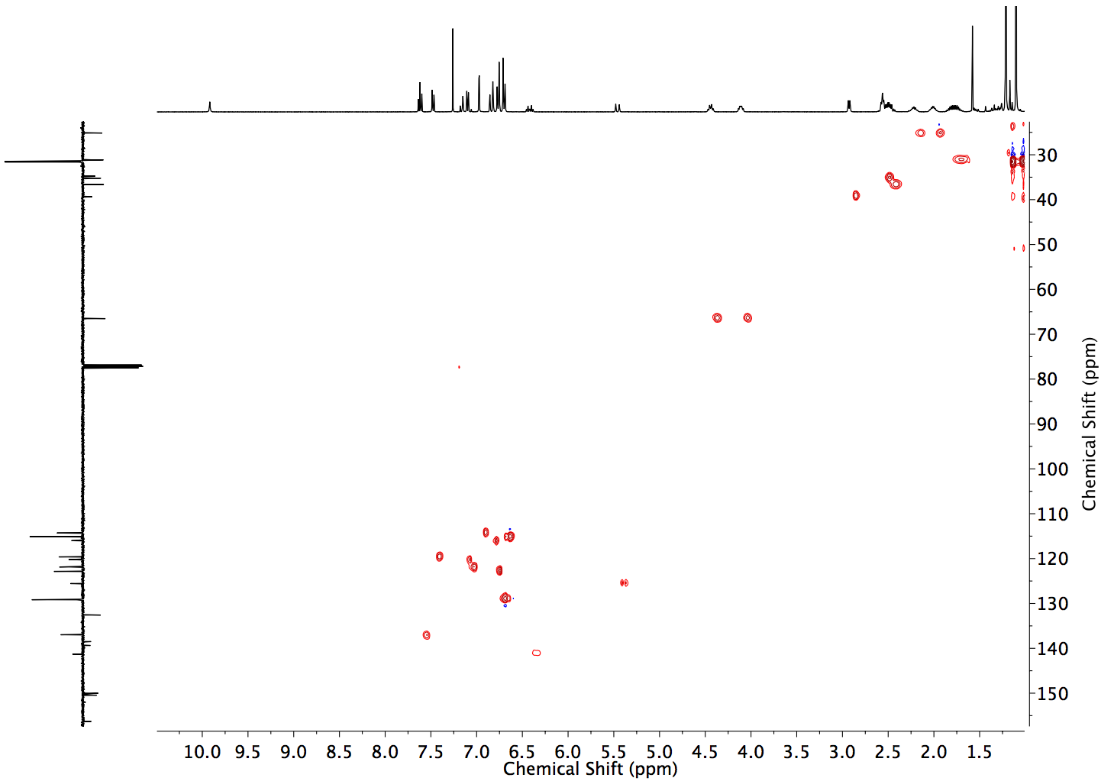

Figure S49 HSQC NMR ($CDCl_3$) of **10**

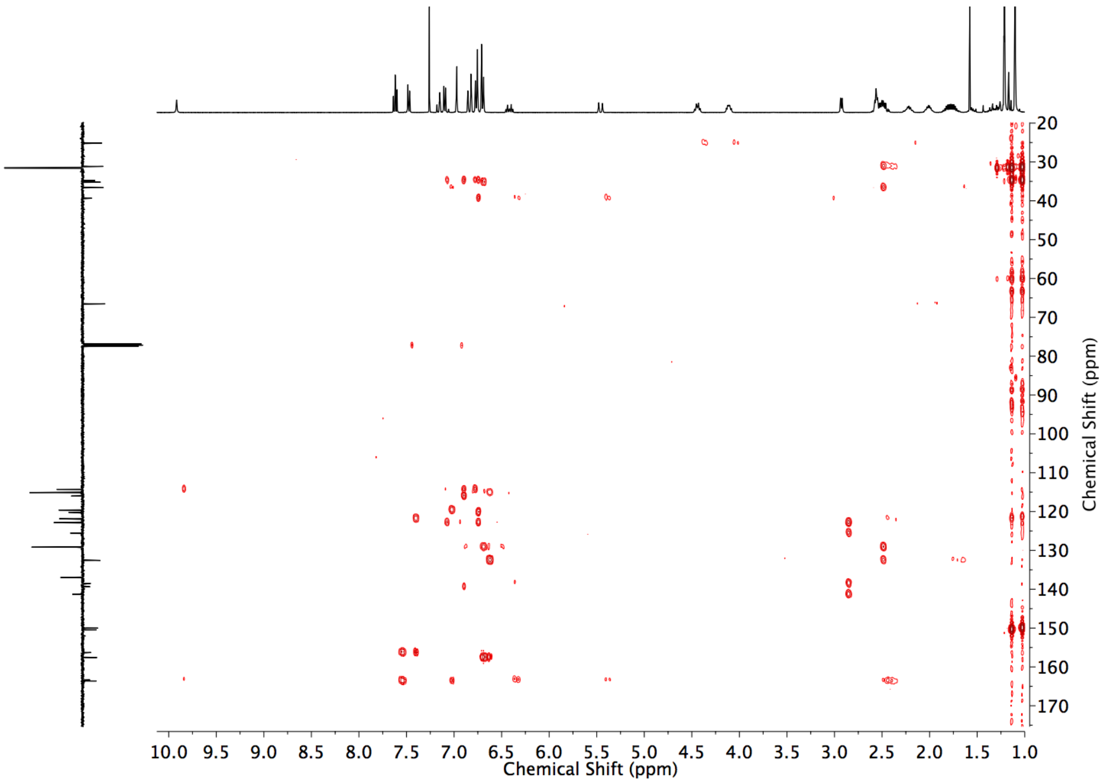

Figure S50 HMBC NMR ($CDCl_3$) of **10**

Rotaxane **11**

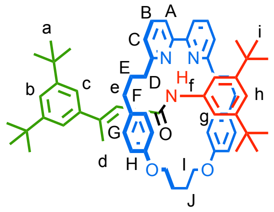

Prepared according to **general procedure A** with **1a** (12.0 mg, 0.025 mmol), $[Cu(MeCN)_4]PF_6$ (8.9 mg, 0.024 mmol), **2a** (6.9 mg, 0.03 mmol), and **3c** (7.7 mg, 0.03 mmol). Chromatography (petrol with a gradient of 0 to 30% Et_2O) gave **11** as a colourless oil (19.9 mg, 85 %). ^1H NMR (500 MHz, $CDCl_3$) δ: 10.29 (s, 1H, H_f), 7.66 (t, *J* =7.8, 2H, H_B), 7.55 (dd, *J* =7.8, 0.9, 2H, H_A), 7.24 (d, *J* =1.9, 2H, H_g), 7.17 (t, *J* =1.9, 1H, H_b), 7.13 (dd, *J* =7.8, 0.9, 2H, H_C), 7.05 (d, *J* =1.9, 2H, H_c), 6.93 (t, *J* =1.9, 1H, H_h), 7.74 (d, *J* =8.5, 4H, H_G), 6.61 (d, *J* =8.5, 4H, H_H), 6.54 (q, *J* =1.2, 1H, H_e), 4.72 – 4.64 (m, 2H, 2 of H_I), 4.21 – 4.15 (m, 2H, 2 of H_I), 2.59 – 2.43 (m, 8H, H_D, H_F), 2.35 – 2.25 (m, 2H, 2 of H_J), 2.12 (d, *J* =1.2, 3H, Hd), 2.09 – 1.98 (m, 2H, 2 oh H_J), 1.82 – 1.60 (m, 4H, H_E), 1.19 (s, 18H, H_a), 1.08 (s, 18H, H_i). ^{13}C NMR (126 MHz, $CDCl_3$) δ: 164.0, 163.2, 157.7, 156.5, 150.0, 149.9, 147.5, 142.8, 140.6, 137.0, 132.1, 128.9, 122.2, 122.1, 121.3, 120.5, 119.4, 115.3, 115.2, 113.3, 66.4, 36.8, 35.4, 34.9, 34.8, 31.6, 31.6, 31.4, 25.0, 17.0. HR-ESI-MS *m/z* = 940.6343 $[M+H]^+$ (calc. for $C_{64}H_{82}N_3O_3$ 940.6351).

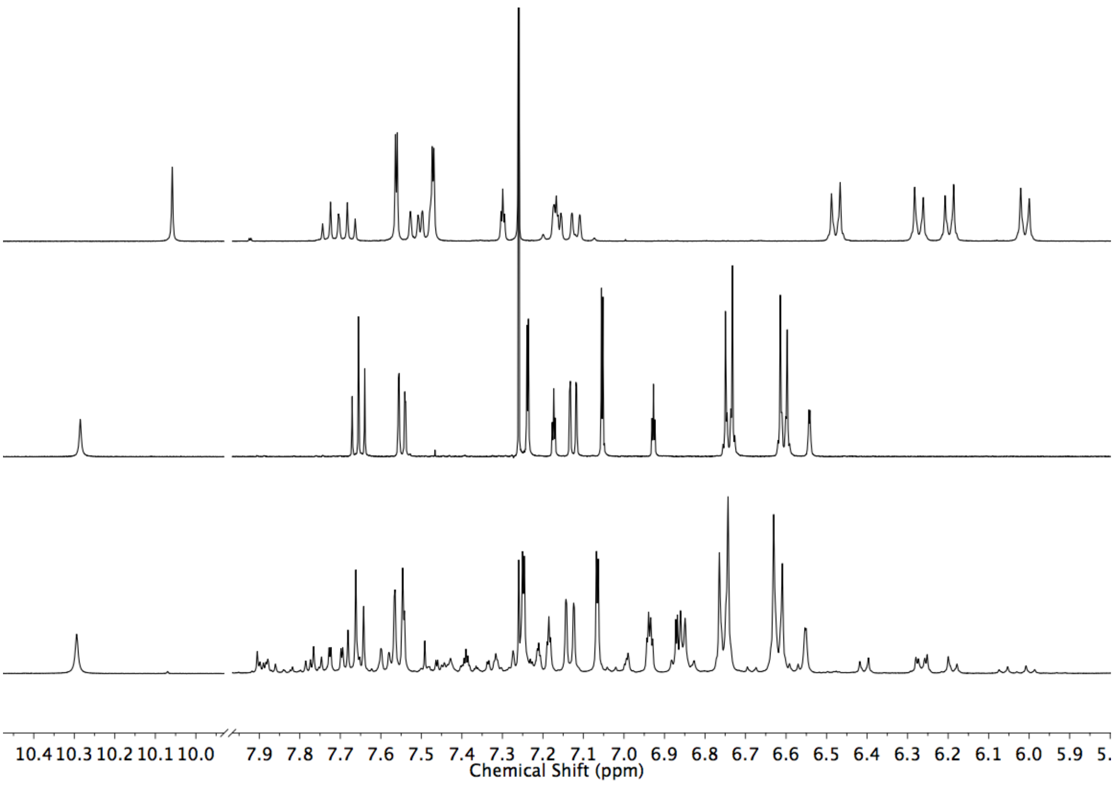

Figure S51 Stacked partial ^1H NMR (400 MHz, $CDCl_3$) spectra of **S9** (top), **11** (middle) and the crude reaction product before chromatography (bottom). Ratio of **11** : **S9** = 95 : 5.

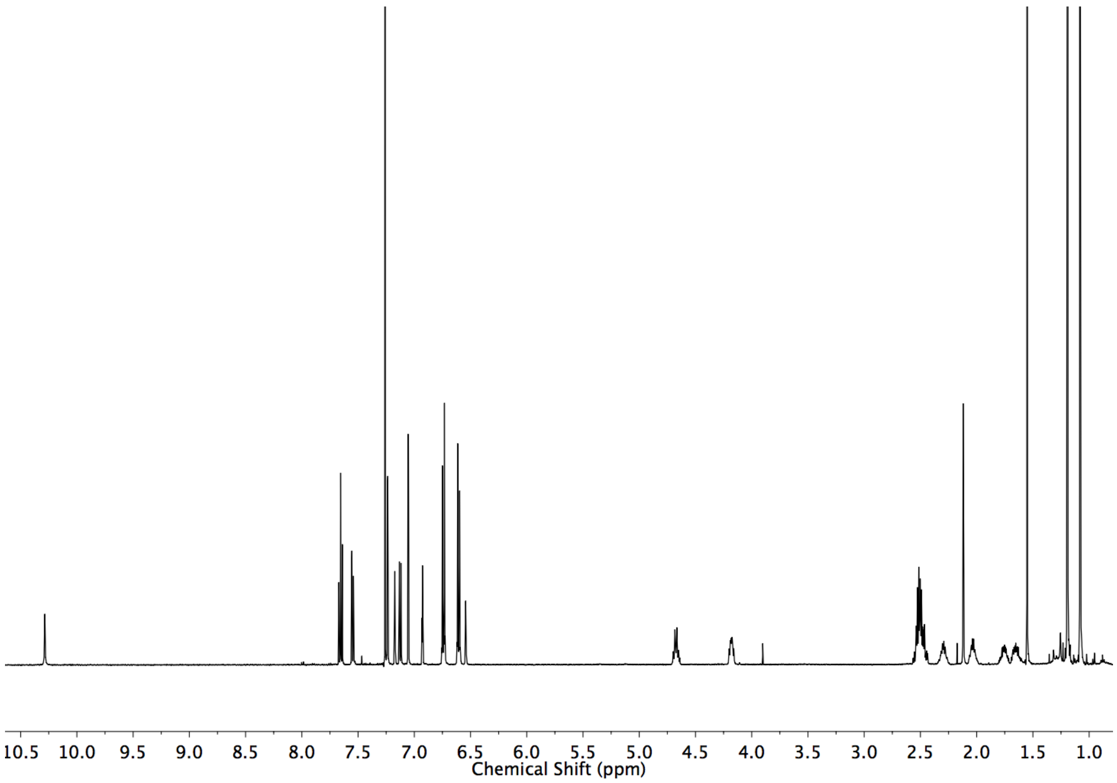

Figure S52 ^{1}H NMR ($CDCl_3$, 500 MHz) of **11**

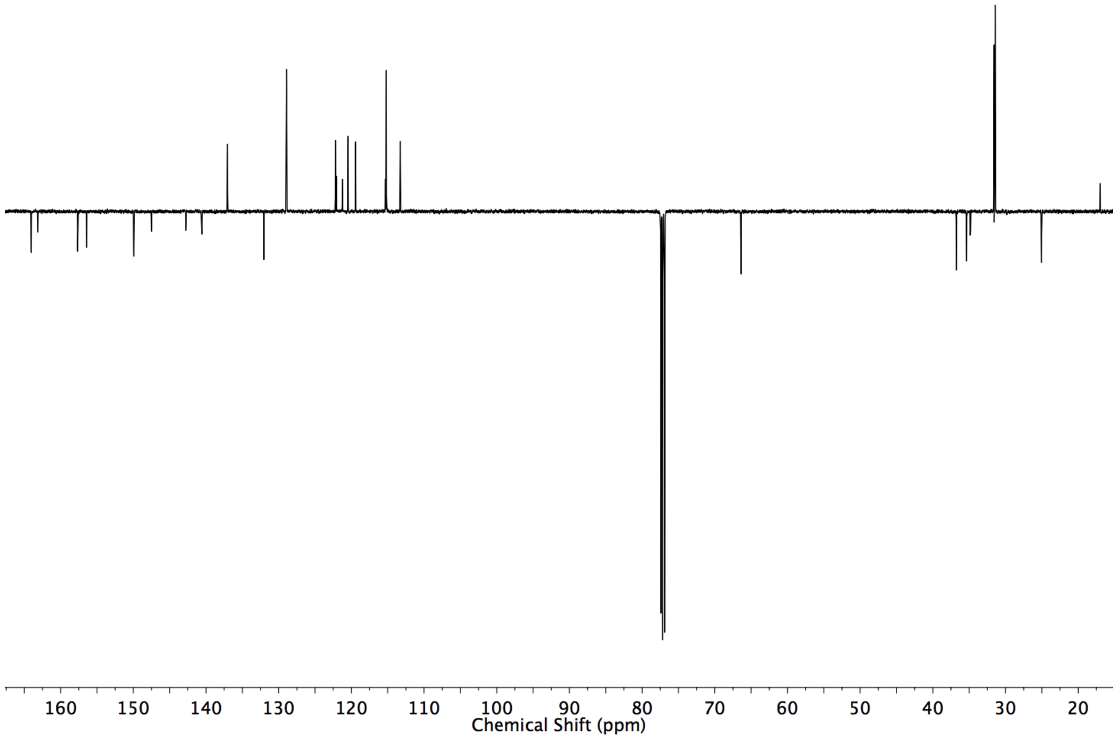

Figure S53 JMOD NMR ($CDCl_3$, 126 MHz) of **11**

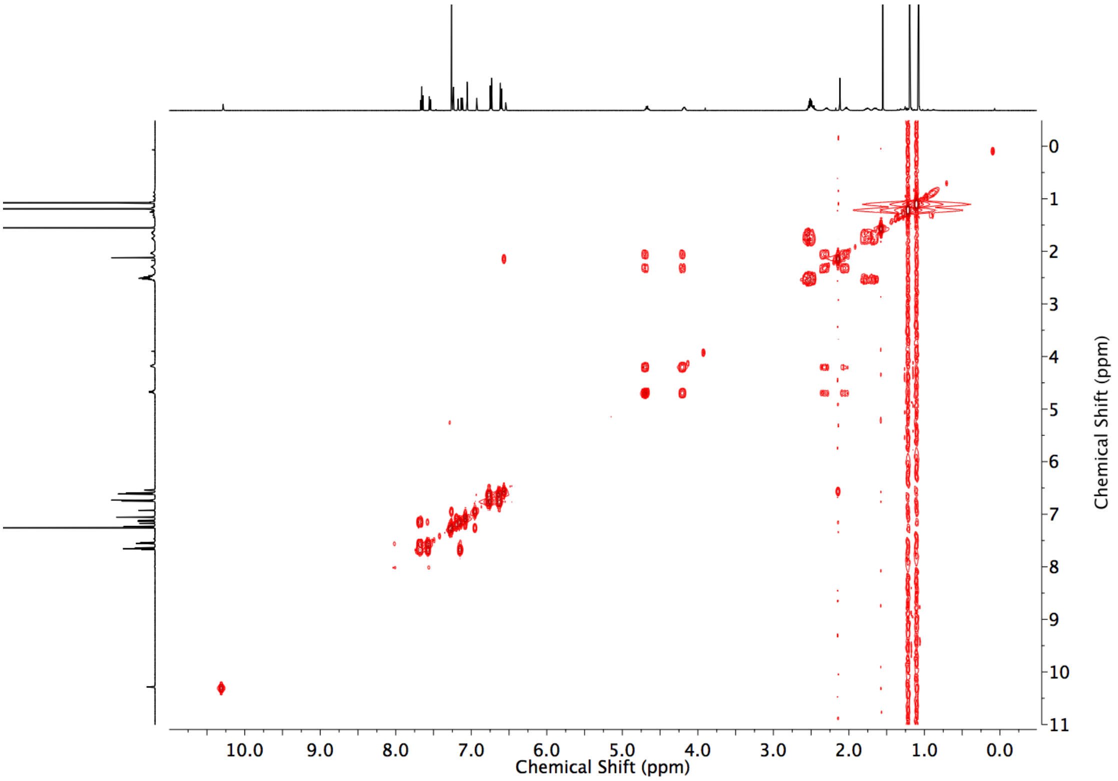

Figure S54 COSY NMR ($CDCl_3$) of **11**

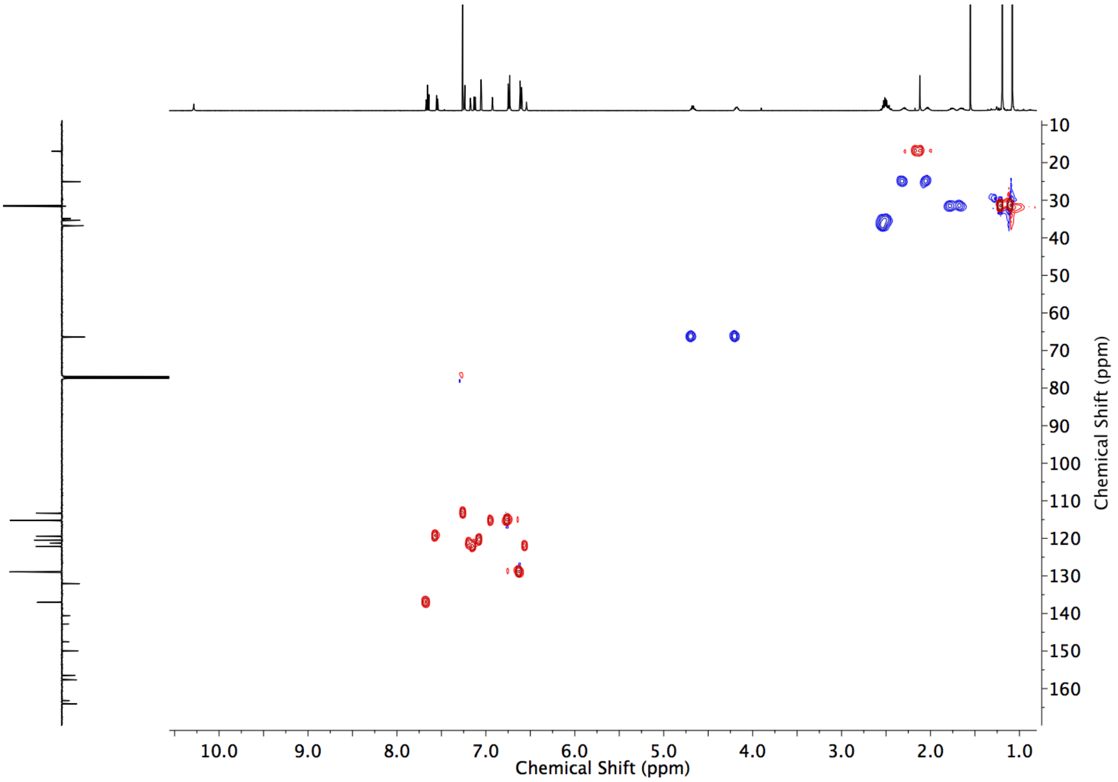

Figure S55 HSQC NMR ($CDCl_3$) of **11**

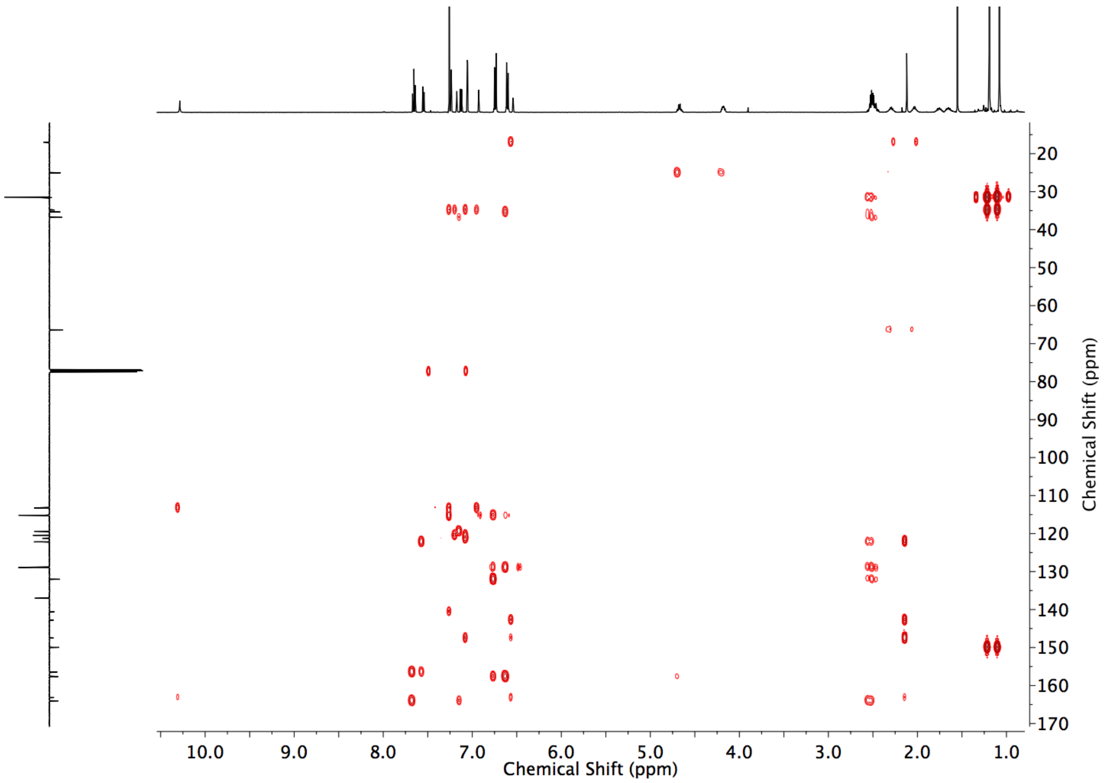

Figure S56 HMBC NMR ($CDCl_3$) of **11**

4. Synthesis of triazole rotaxanes

General Procedure B

N^{i}Pr$_2$Et (2 eq.) was added to a solution of **alkyne** (1.2 eq.), **azide** (1.2 eq.), **macrocycle** (1 eq.) and [Cu(MeCN)$_4$]PF$_6$ (0.96 eq.) in CH_2Cl_2 (80 mL/mmol) in a sealed microwave vial (CEM Ltd.). The deep red mixture was stirred at rt for 16 hours. The reaction mixture was diluted with CH_2Cl_2 (200 mL/mmol) and washed with EDTA-NH$_3$ solution (100 mL/mmol). The aqueous layer was extracted with CH_2Cl_2 (2 × 100 mL/mmol). Combined organic extracts were washed with brine (100 mL/mmol), dried ($MgSO_4$), filtered and the solvent removed *in vacuo*.

Rotaxane **4**

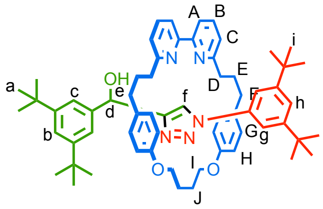

Prepared according to **general procedure B** with **1a** (12.0 mg, 0.025 mmol), [Cu(MeCN)$_4$]PF$_6$ (8.9 mg, 0.024 mmol), **2a** (7.0 mg, 0.03 mmol), and **3a** (7.3 mg, 0.03 mmol). Chromatography (petrol with a gradient of 0 to 50% Et$_2$O) gave **4** as a white foam (18.0 mg, 76%). ^{1}H NMR (500 MHz, CDCl$_3$): δ 10.08 (d, J =0.8, 1H, H$_f$), 7.73 (t, J =7.8 1H, 1 of H$_B$), 7.69 (t, J =7.8, 1H, 1 of H$_B$), 7.53 (dd, J =7.8, 1.0, 1H, 1 of H$_A$), 7.50 (dd, J =7.8, 1.0, 1H, 1 of H$_A$), 7.44 (dd, J =1.9, 0.6, 2H, H$_c$), 7.41 (d, J =1.8, 2H, H$_g$), 7.34 (t, J =1.9, 1H, H$_b$), 7.18 – 7.21 (m, 2H, 1 of H$_C$, H$_h$), 7.13 (dd, J =7.8, 0.8, 1H, 1 of H$_C$), 6.63 (d, J =8.6, 2H, 1 of H$_H$), 6.51 (d, J =8.6, 2H, 1 of H$_G$), 6.05 – 6.11 (m, 4H, 1 of H$_G$, 1 of H$_H$), 5.66 (br s, 1H, H$_d$), 4.64 – 4.77 (m, 1H, 1 of H$_I$), 4.49 – 4.60 (m, 1H, 1 of H$_I$), 4.41 (s, 1H, H$_e$), 4.21 – 4.30 (m, 1H, 1 of H$_I$), 3.91 – 4.00 (m, 1H, 1 of H$_I$), 2.12 – 2.60 (m, 10H, H$_D$, 2 of H$_J$, H$_F$), 1.96 – 2.07 (m, 2H, 2 of H$_J$), 1.53 – 1.79 (m, 4H, H$_E$), 1.32 (s, 18H, H$_a$), 1.19 (s, 18H, H$_i$). ^{13}C NMR (126 MHz, CDCl$_3$): δ 163.4, 163.1, 157.9, 157.1, 157.1, 156.9, 151.1, 150.0, 149.9, 142.6, 137.3, 137.1, 137.1, 131.7, 151.5, 128.3, 128.2, 122.4, 121.6, 121.5, 121.4, 120.6, 120.2, 120.1, 120.1, 115.4, 114.4, 114.3, 69.9, 66.8, 66.3, 36.9, 36.1, 35.1, 35.1, 35.0, 34.7, 32.3, 31.7, 31.4, 30.0, 25.1, 24.9. HR-ESI-MS m/z = 954.6257 [M+H]$^+$ (calc. for $C_{63}H_{80}N_5O_3$ 954.6256).

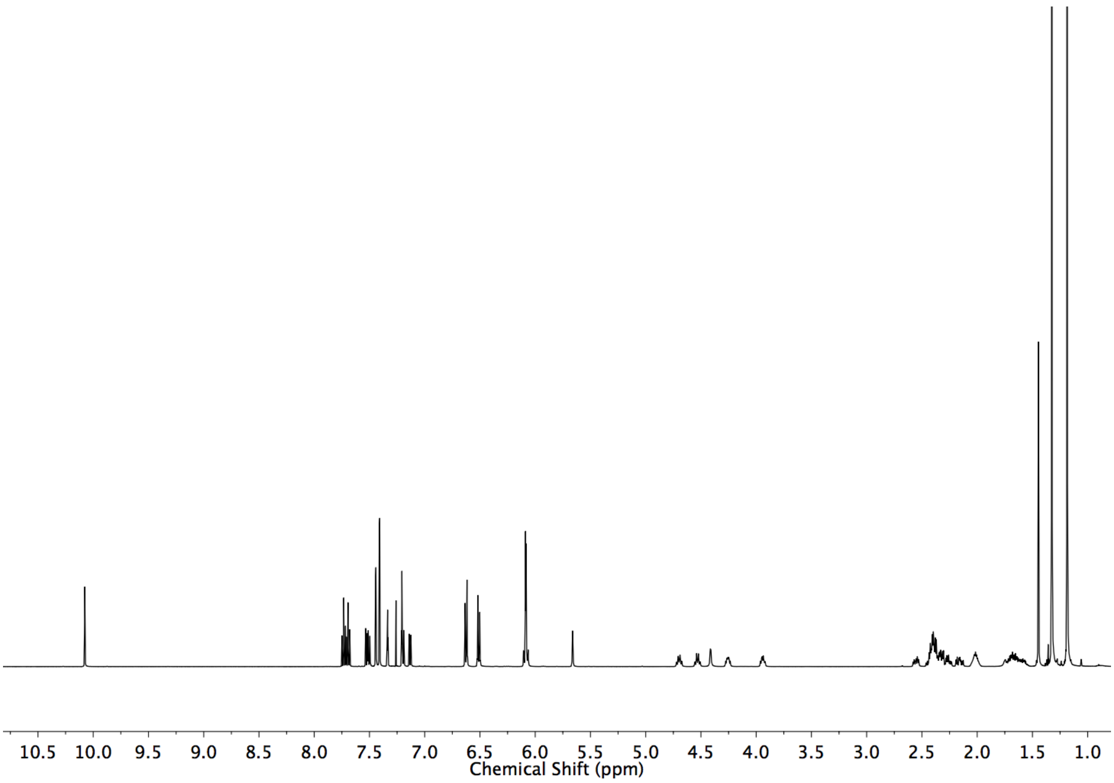

Figure S57 ^{1}H NMR ($CDCl_3$, 500 MHz) of **4**

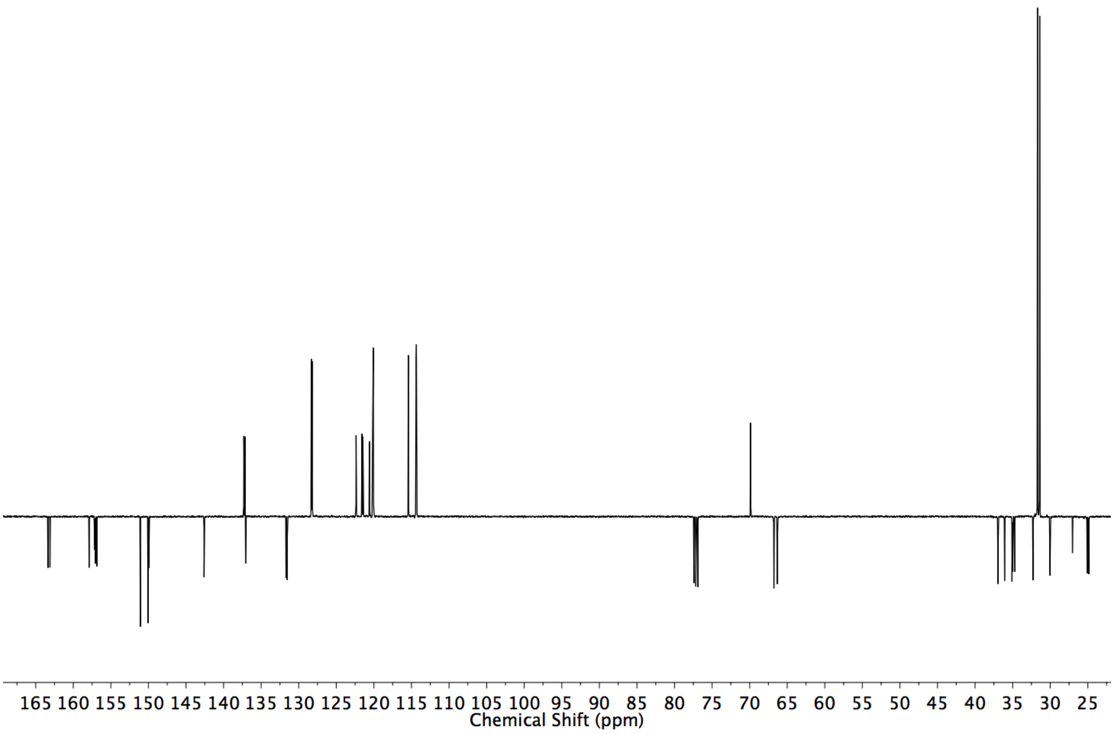

Figure S58 JMOD NMR ($CDCl_3$, 126 MHz) of **4**

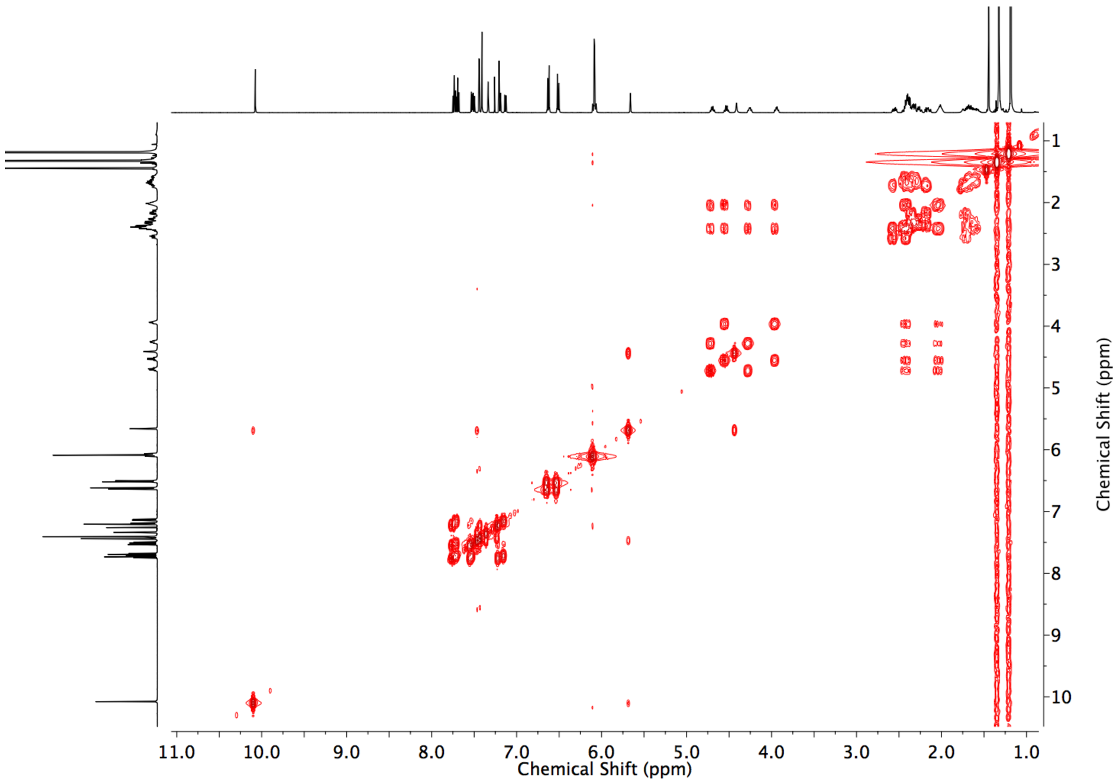

Figure S59 COSY NMR ($CDCl_3$) of **4**

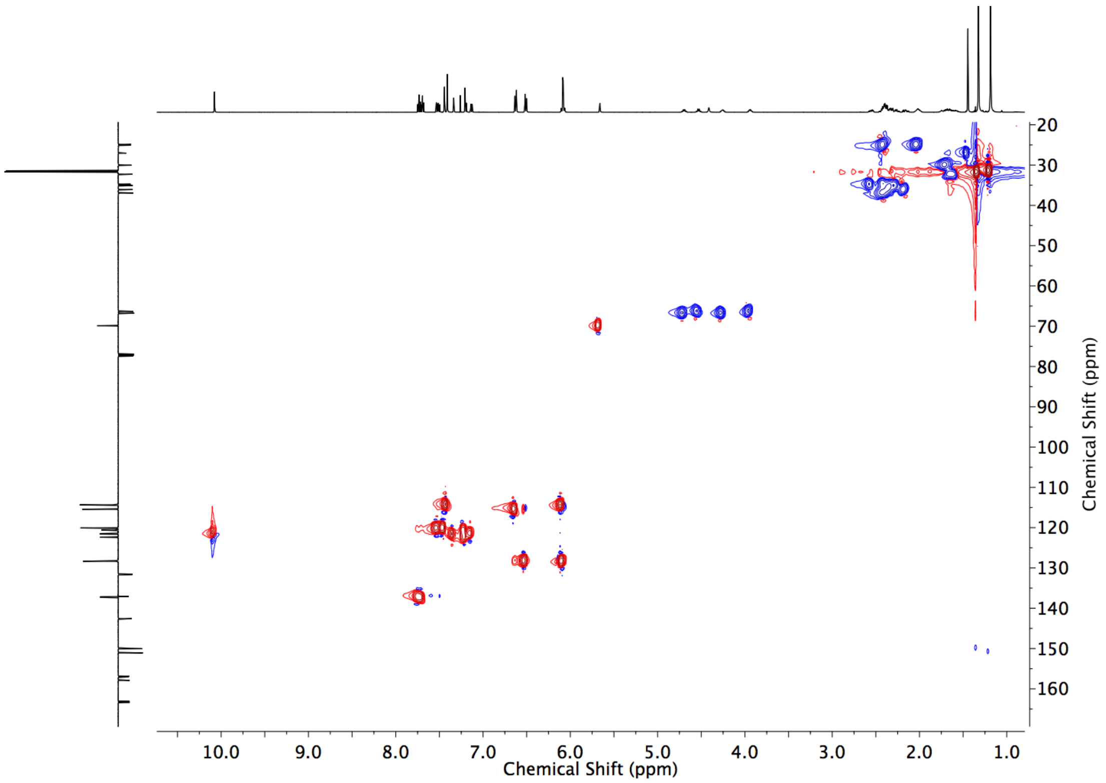

Figure S60 HSQC NMR ($CDCl_3$) of **4**

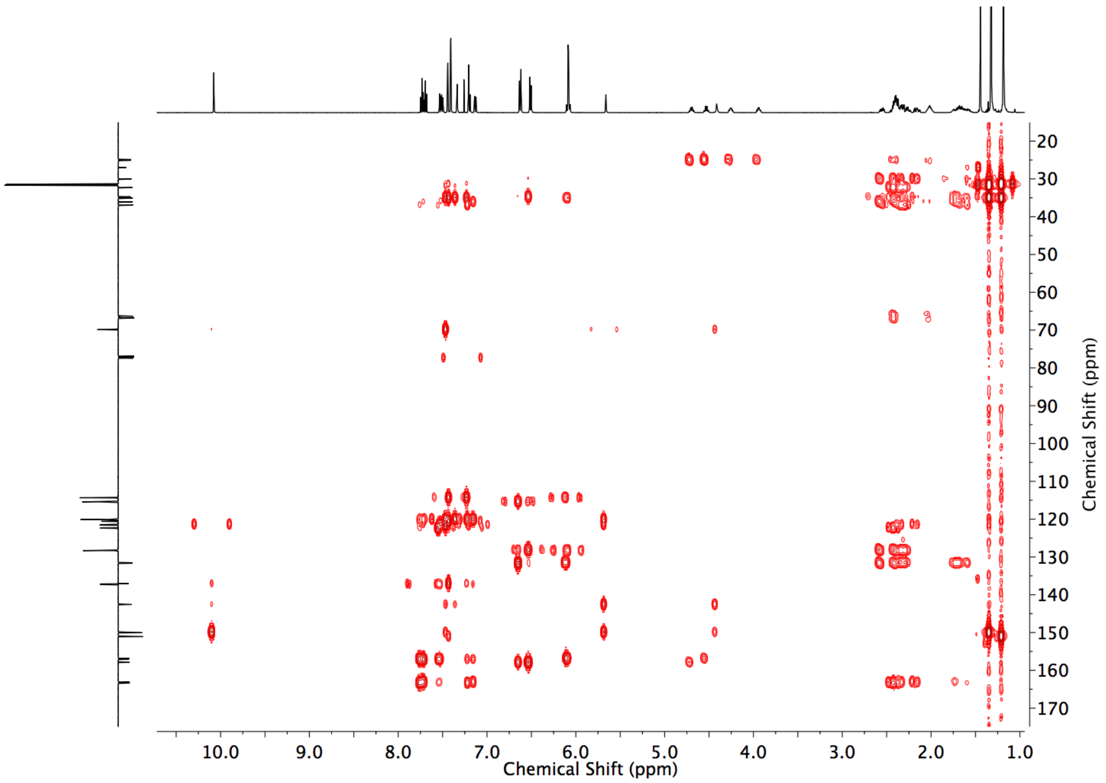

Figure S61 HMBC NMR ($CDCl_3$) of **4**

Rotaxane **S4**

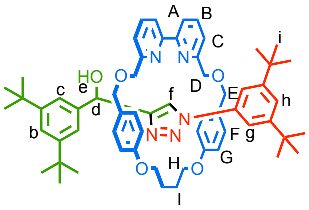

Prepared according to **general procedure B** with **1b** (48.3 mg, 0.1 mmol), $[Cu(MeCN)_4]PF_6$ (35.8 mg, 0.096 mmol), **2a** (29.3 mg, 0.12 mmol), and **3a** (27.8 mg, 0.12 mmol). Chromatography (petrol with a gradient of 0 to 50% Et_2O) gave **S4** as a white foam (90.0 mg, 97%). ^{1}H NMR (400 MHz, $CDCl_3$): δ 9.65 (s, 1H, H_f), 7.75 – 7.68 (m, 2H, H_B), 7.62 (dd, *J* =7.8, 1.0, 1H, 1 of H_A), 7.58 (dd, *J* =7.8, 1.0, 1H, 1 of H_A), 7.42 (dd, *J* =7.8, 1.0, 2H, H_C), 7.40 (d, *J* =1.9, 2.H, H_c), 7.28 (t, *J* =1.9, 1H, H_b), 7.14 (d, *J* =1.9, 2H, H_g), 7.10 (t, *J* =1.9, 1H, H_h), 6.70 (d, *J* =8.5, 2H, 2 of H_F), 6.54 (*J* =8.5, 2H, 2 of H_G), 6.23 (d, *J* =8.5, 2H, 2 of H_F), 5.97 (d, *J* =8.5, 2H, 2 of H_G), 5.62 (s, 1H, H_d), 4.68 (q, *J* =7.8, 1H, 1 of H_H), 4.57 (d, *J* =12.2, 1H, 1 of H_E), 4.48 (q, *J* =7.8, 1H, 1 of H_H), 4.36 (d, *J* =12.2, 1H, 1 of H_E), 4.21 (d, *J* =12.2, 1H, 1 of H_E), 4.16 (q, *J* =7.8, 1H, 1 of H_I), 4.02 (t, *J* =12.2, 2H, 1 of H_D, 1 of H_E), 3.95 (q, *J* =12.2, 2H, 2 of H_D), 3.83 (q, *J* =7.8, 1H, 1 of H_I), 3.77 (d, *J* =12.2, 1H, H_D), 2.40 – 2.33 (m, 2H, 2 of H_I), 2.00 – 1.83 (m, 2H, 2 of H_I), 1.27 (s, 18H, H_a or H_h), 1.09 (s, 18H, H_a or H_h). ^{13}C NMR (101 MHz, $CDCl_3$): δ 159.6, 159.6, 159.3, 158.6, 155.2, 155.1, 151.2, 147.9, 149.7, 142.3, 137.6, 137.4, 136.8, 129.4, 128.9, 127.9, 127.2, 121.8, 121.5, 120.9, 120.7, 120.4, 120.2, 120.2, 120.1, 115.3, 114.2, 114.0, 73.0, 72.9, 70.5, 70.0, 69.8, 66.7, 66.0, 34.9, 34.9, 31.6, 31.3, 24.8, 24.7. HR-ESI-MS *m/z* = 958.5843 $[M+H]^+$ (calc. for $C_{61}H_{76}H_5N_5$ 958.5841).

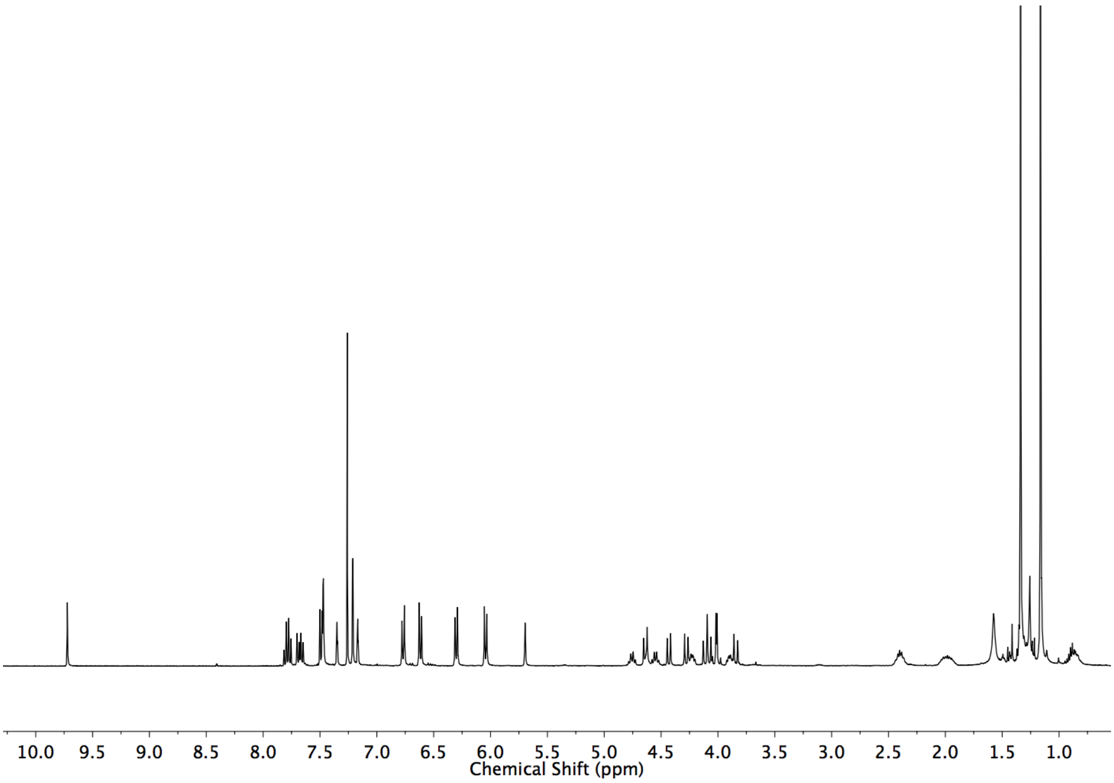

Figure S62 ^{1}H NMR ($CDCl_3$, 400 MHz) of **S4**

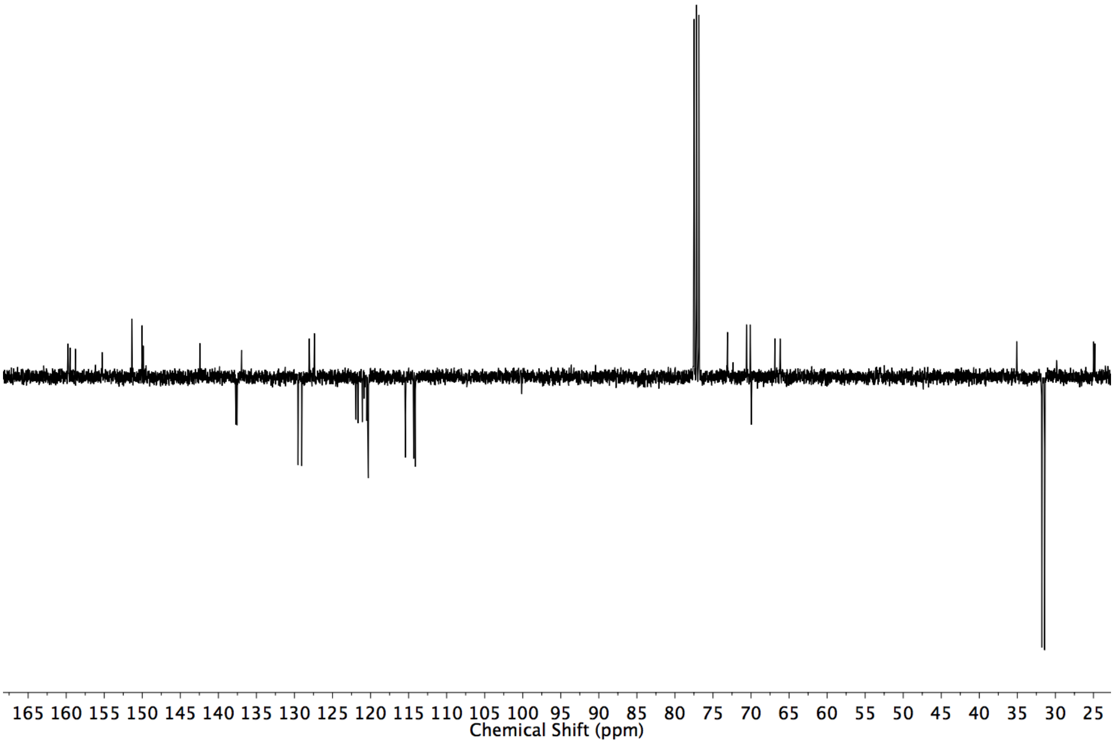

Figure S63 JMOD NMR ($CDCl_3$, 101 MHz) of **S4**

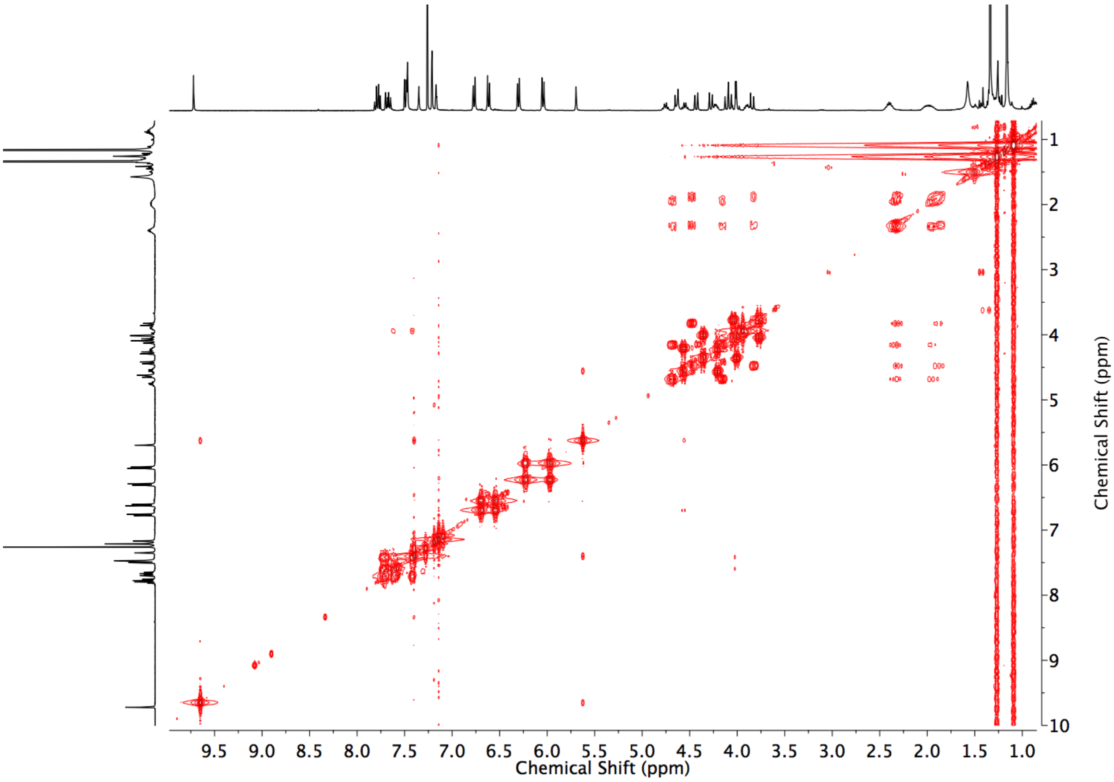

Figure S64 COSY NMR ($CDCl_3$) of **S4**

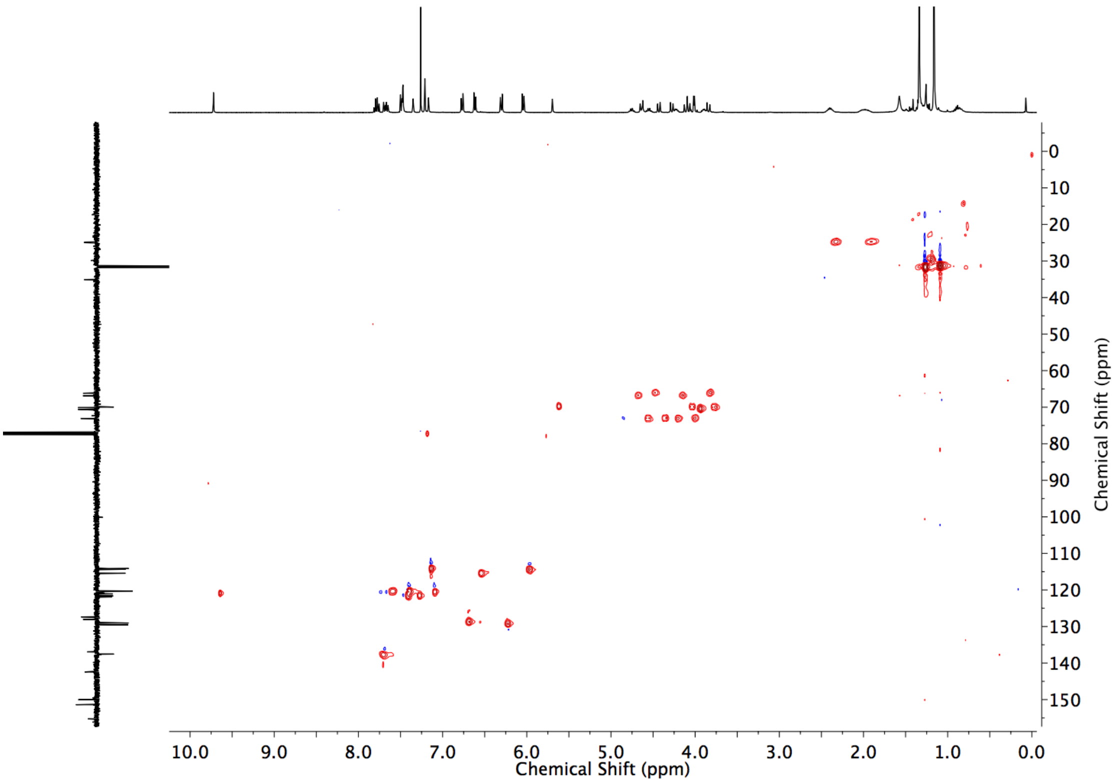

Figure S65 HSQC NMR ($CDCl_3$) of **S4**

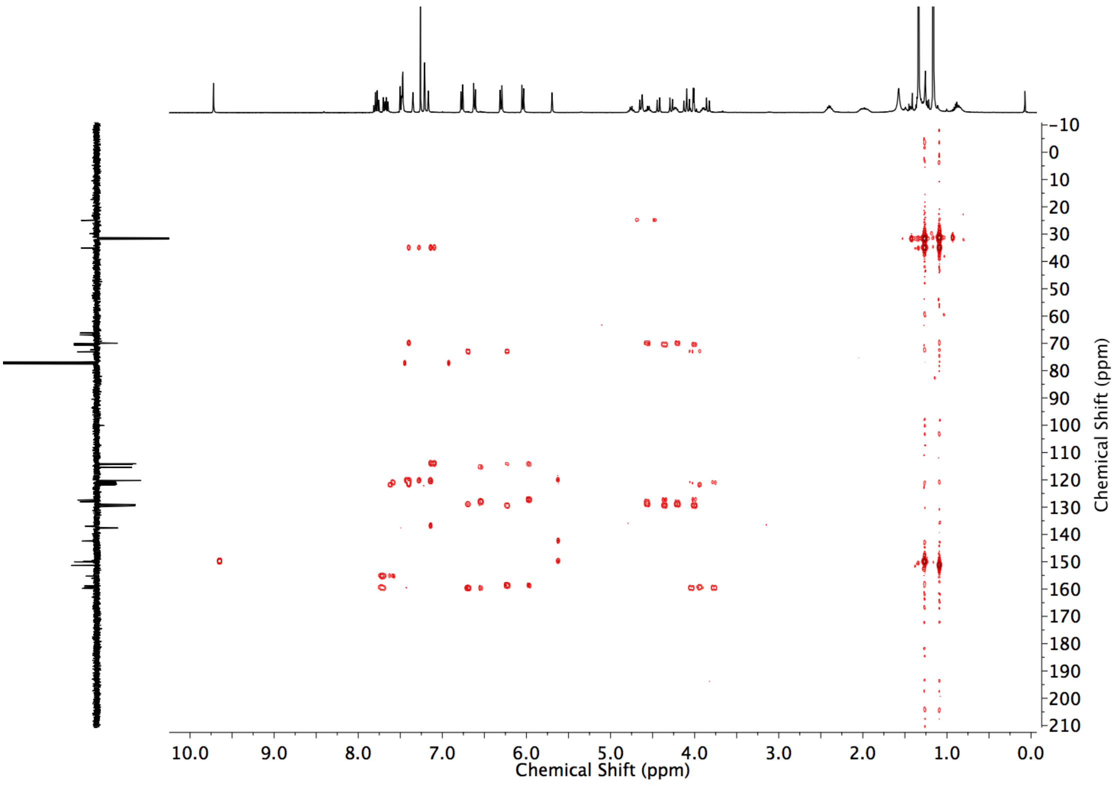

Figure S66 HMBC NMR ($CDCl_3$) of **S4**

Rotaxane **S5**

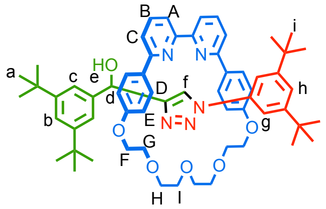

Prepared according to **general procedure B** with **1d** (12.0 mg, 0.025 mmol), $[Cu(MeCN)_4]PF_6$ (8.9 mg, 0.024 mmol), **2a** (6.9 mg, 0.03 mmol), and **3a** (7.3 mg, 0.03 mmol). Chromatography (petrol with a gradient of 0 to 50% Et_2O) gave **S5** as a white foam (9.0 mg, 37%). ^1H NMR (500 MHz, $CDCl_3$) δ: 9.91 (s, 1H, H_f), 7.93 (t, J = 7.8, 1H, 1 of H_B), 7.84 (t, J = 7.8, 1H, 1 of H_B), 7.79 (dd, J = 7.8, 0.9, 1H, 1 of H_A), 7.77 (dd, J = 7.8, 0.9, 1H, 1 of H_A), 7.64 (dd, J = 7.8, 0.9, 1H 1 of H_C), 7.46 (dd, J = 7.8, 0.9, 1H, 1 of H_C), 7.41 (d, J = 1.8, 2H, H_g), 7.29 (d, J = 8.7, 2H, 2 of H_E), 7.21 (t, J =1.8, 1H, H_h), 7.15 (t, J = 1.8, 1H, H_b), 7.11 (d, J = 1.8, 2H, H_c), 6.86 (d, J = 8.7, 2H, 2 of H_E), 6.35 (d, J = 8.7, 2H, 2 of H_D), 6.07 (d, J = 8.7, 2H, 2 of H_D), 5.00 (d, J = 2.3, 1H, H_d), 4.07 – 3.51 (m, 16H, H_F, H_G, H_H, H_I), 2.99 (d, J = 2.3, 1H, H_e), 1.12 (s, 18H, H_i), 1.09 (s, 18H, H_a). ^{13}C NMR (126 MHz, $CDCl_3$) δ 160.07, 159.47, 159.12, 158.45, 157.67, 157.40, 151.48, 150.44, 149.46, 142.21, 137.60, 137.53, 136.71, 132.80, 132.08, 129.54, 129.13, 125.67, 123.99, 121.90, 121.64, 120.64, 120.35, 120.15, 119.93, 119.62, 114.67, 114.33, 114.15, 70.14, 70.01, 69.91, 69.79, 69.64, 68.85, 68.22, 67.17, 66.09, 34.98, 34.79, 31.47, 31.33. HR-ESI-MS *m/z* = 974.5783 $[M+H]^+$ (calc. for $C_{61}H_{76}N_5O_6$ 974.5790).

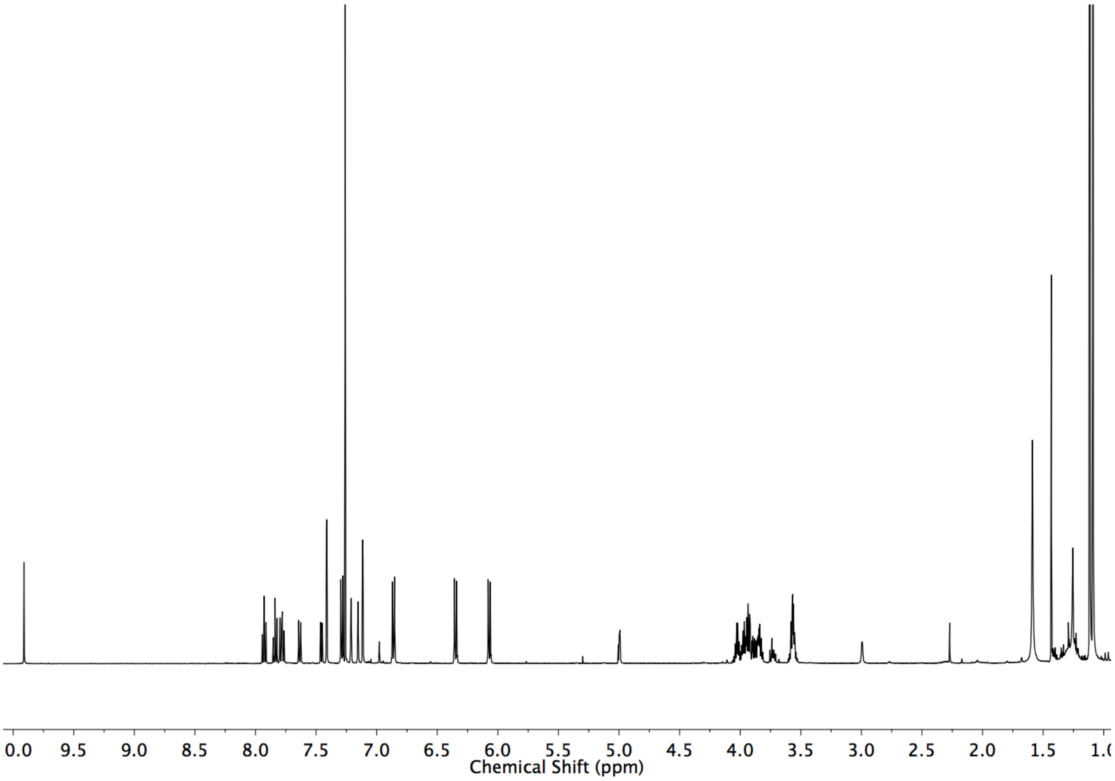

Figure S67 ^1H NMR ($CDCl_3$, 500 MHz) of **S5**

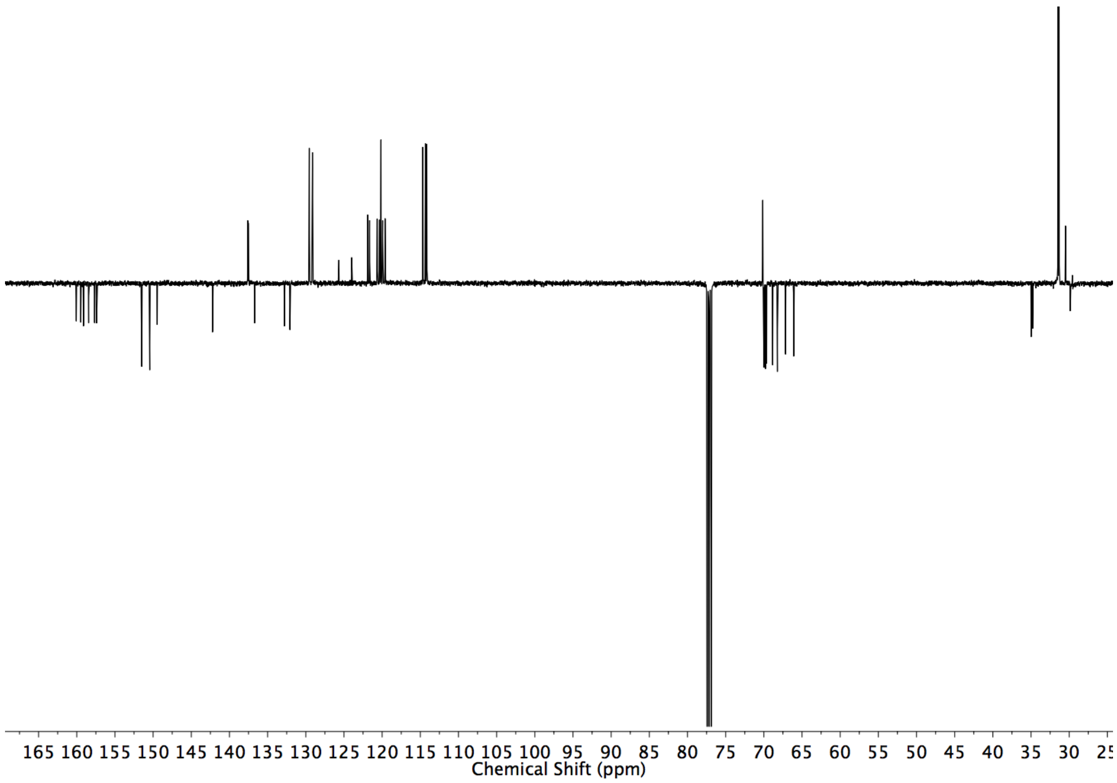

Figure S68 JMOD NMR ($CDCl_3$, 126 MHz) of **S5**

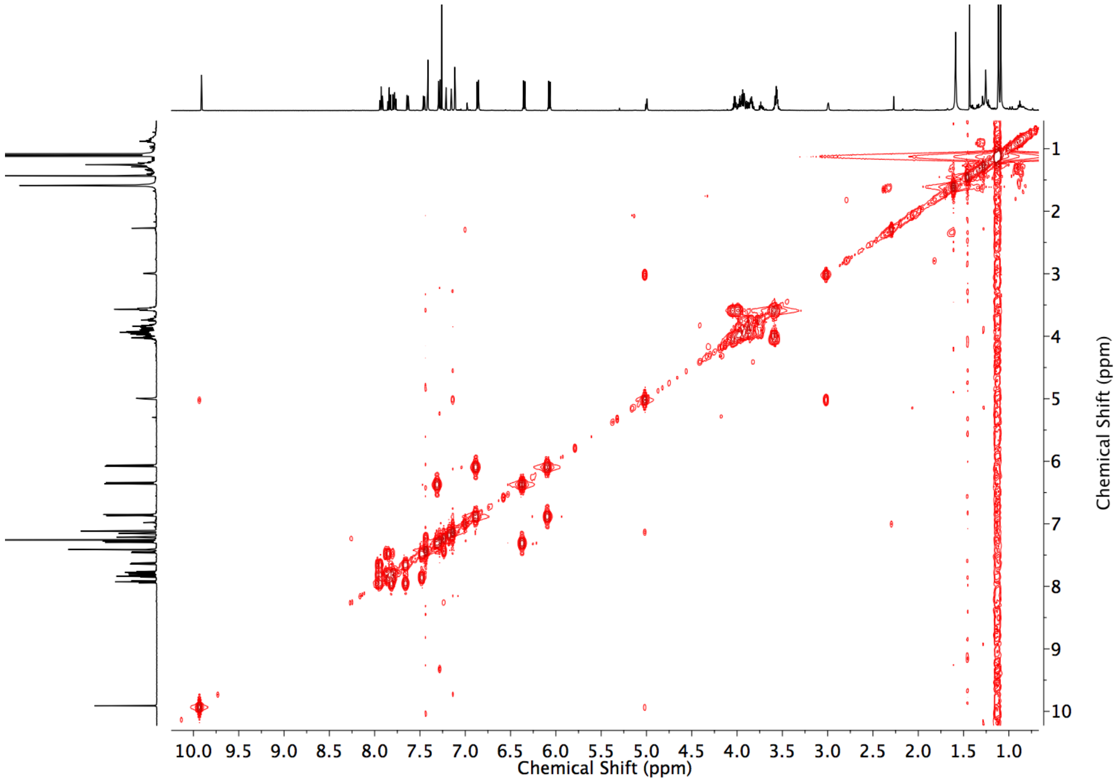

Figure S69 COSY NMR ($CDCl_3$) of **S5**

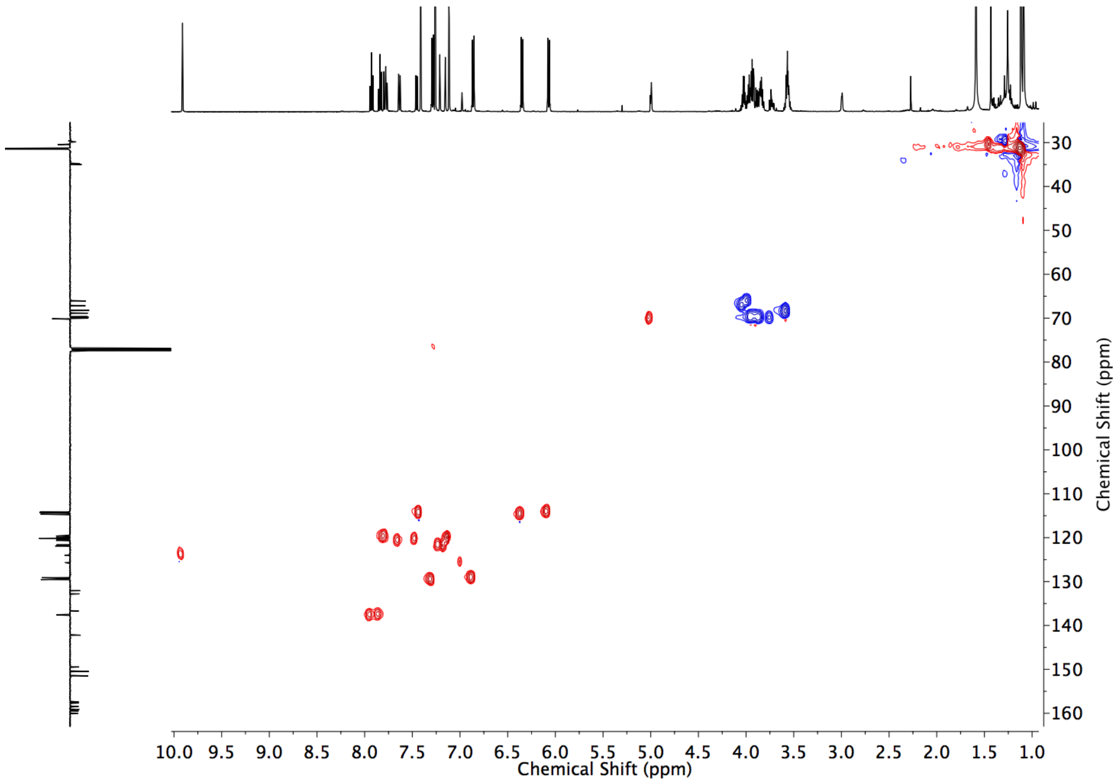

Figure S70 HSQC NMR ($CDCl_3$) of **S5**

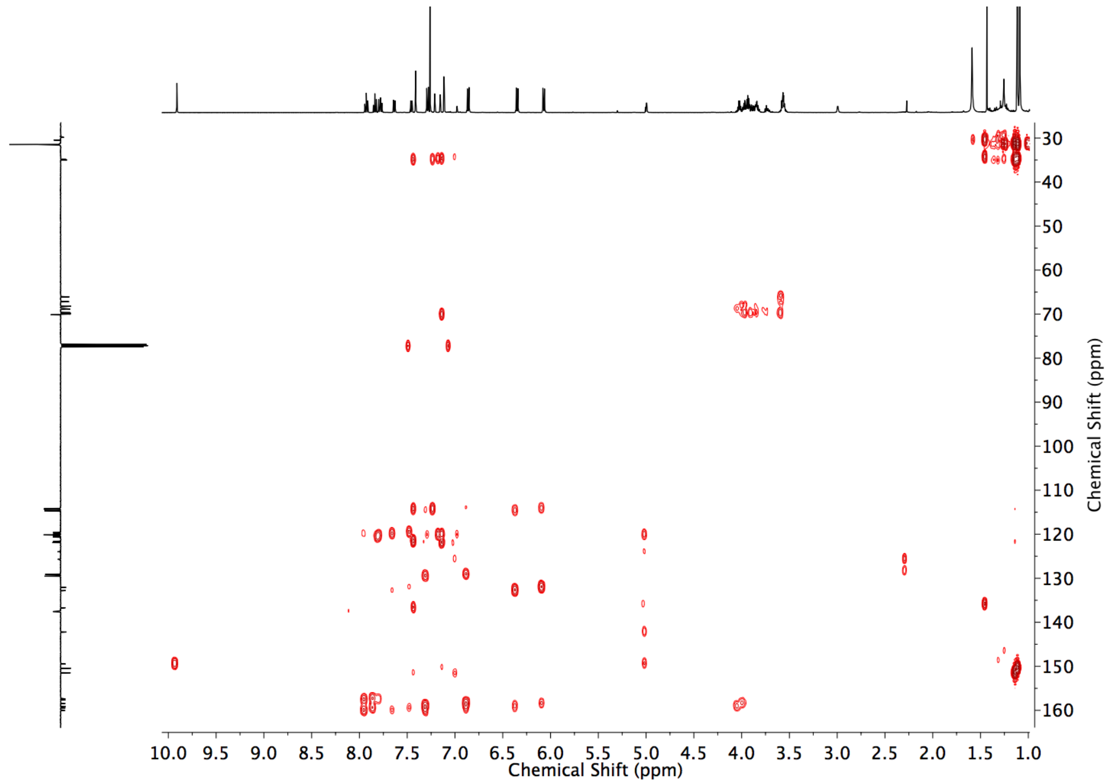

Figure S71 HMBC NMR ($CDCl_3$) of **S5**

Rotaxane **S6**

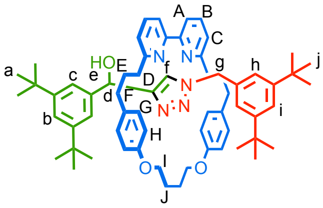

Prepared according to **general procedure B** with **1a** (12.0 mg, 0.025 mmol), $[Cu(MeCN)_4]PF_6$ (8.9 mg, 0.024 mmol), **2b** (7.3 mg, 0.03 mmol), and **3a** (7.3 mg, 0.03 mmol). Chromatography (petrol with a gradient of 0 to 50% Et_2O) gave **S6** as a white foam (17.0 mg, 70%). ^1H NMR (400 MHz, $CDCl_3$): δ 8.73 (s, 1H, H_f), 7.62 (t, *J* =7.8, 2H, H_B), 7.26 (app. s, 3H, H_b, H_c), 7.16 (t, *J* =1.8, 1H, H_i), 7.10 (app. t, *J* =7.7, 2H, H_C) 6.79 (d, *J* =1.8, 2H, H_h), 6.68 – 6.57 (m, 4H, 2 of H_G, 2 of H_H), 6.48 (s, 4H, 2 of H_G, 2 of H_H), 5.38 (br. s, 1H, H_d), 4.67 (d, *J* =3.7, 1H, H_e), 4.65 – 4.52 (m, 2H, 2 of H_I), 4.49 (d, *J* =13.9, 1H, 1 of H_g), 4.25 (d, *J* =13.9, 1H, 1 of H_g), 4.18 – 4.00 (m. 2H, 2 of H_I), 2.68 – 2.15 (m, 10H, H_D, H_F, 2 of H_J), 2.03 – 1.95 (m, 2H, 2 of H_J), 1.85 – 1.49 (m, 4H, H_E), 1.26 (s, 18H, H_a or H_j), 1.11 (s, 18H, H_a or H_j). ^{13}C NMR (101 MHz, $CDCl_3$): δ 162.8, 162.8, 157.6, 157.4, 156.9, 156.7, 150.7, 149.9, 149.2, 142.4, 137.1, 136.9, 133.2, 132.4, 132.3, 128.9, 128.7, 124.2, 123.3, 122.8, 121.7, 121.6, 121.5, 120.7, 120.0, 119.9, 115.2, 114.9, 69.8, 66.7, 66.5, 53.4, 36.6, 36.3, 34.9, 34.9, 34.7, 34.7, 31.7, 31.5, 31.1, 30.5, 25.1, 25.0. HR-ESI-MS *m/z* = 968.6402 $[M+H]^+$ (calc. for $C_{64}H_{82}N_5O_3$ 968.6412)

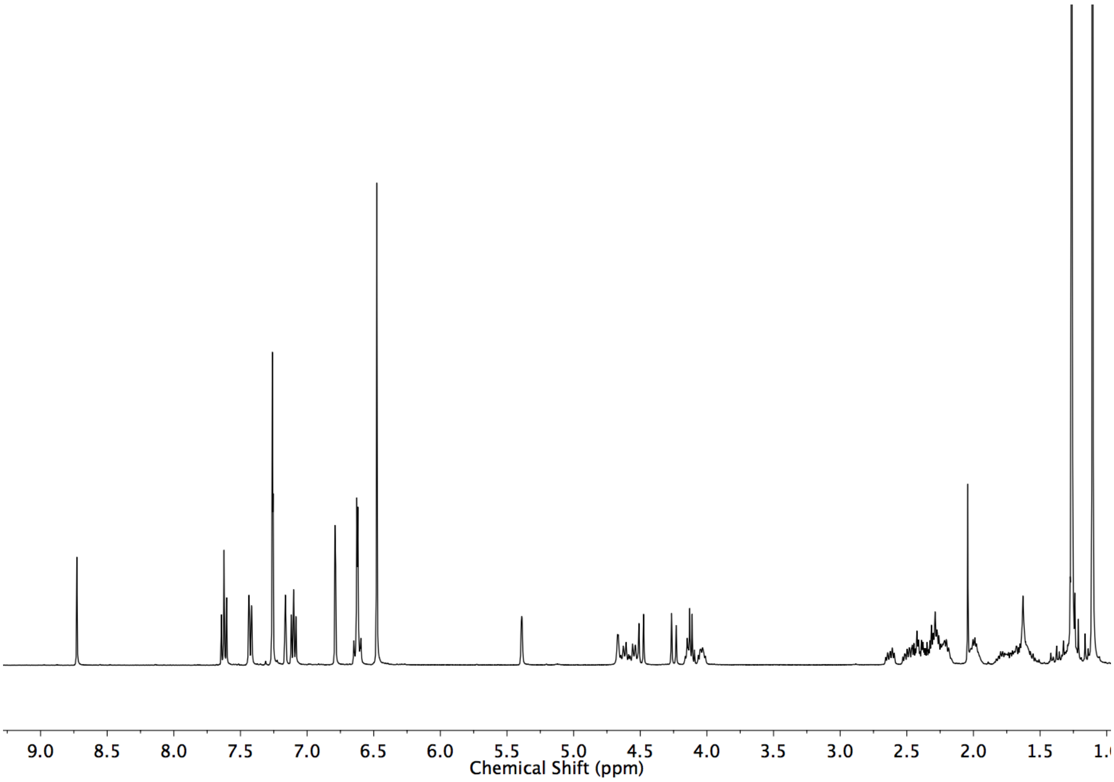

Figure S72 ^1H NMR ($CDCl_3$, 400 MHz) of **S6**

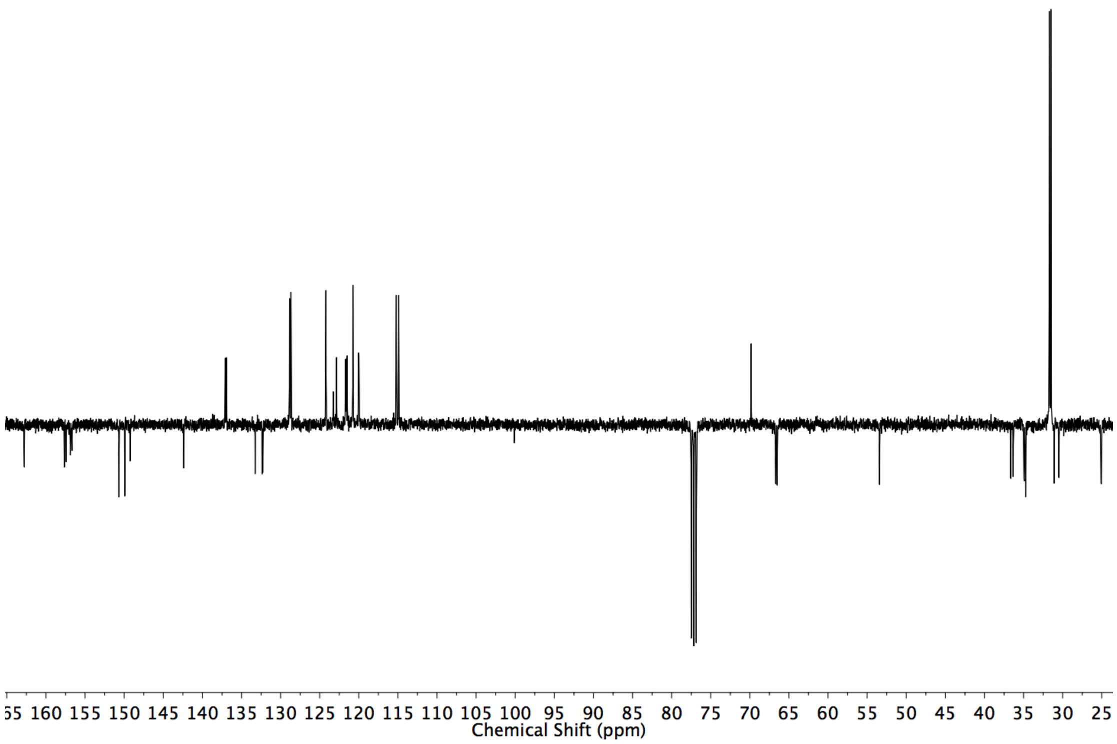

Figure S73 JMOD NMR ($CDCl_3$, 101 MHz) of **S6**

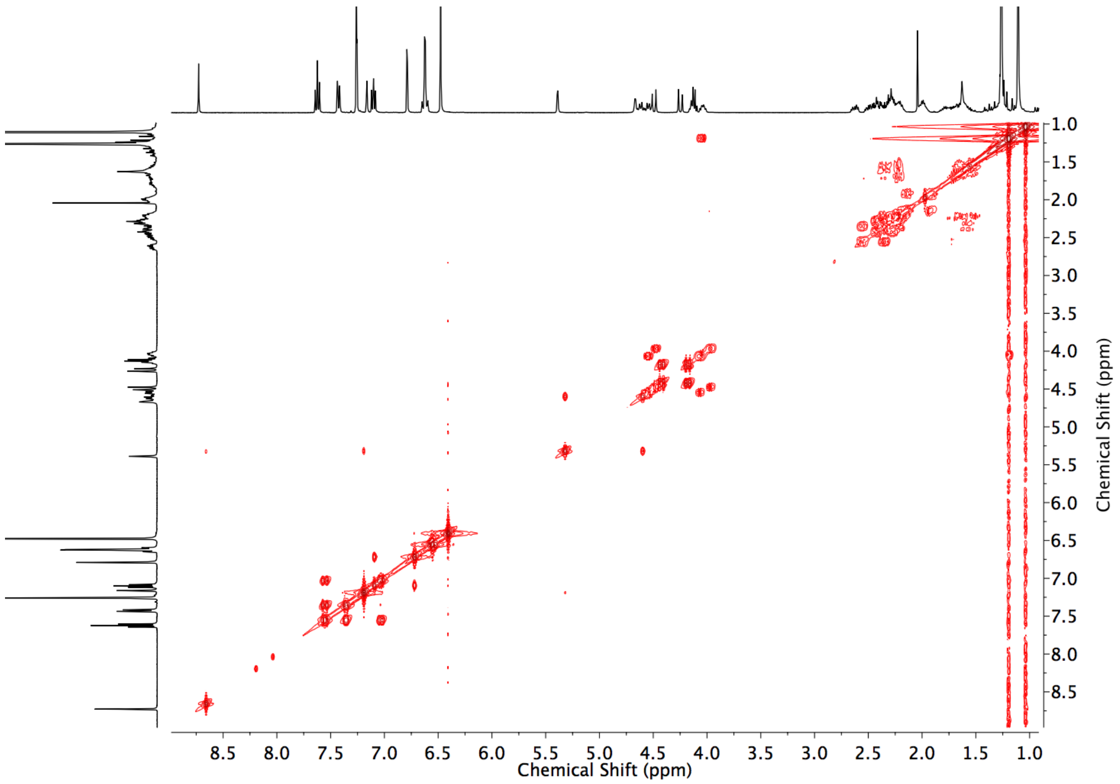

Figure S74 COSY NMR ($CDCl_3$) of **S6**

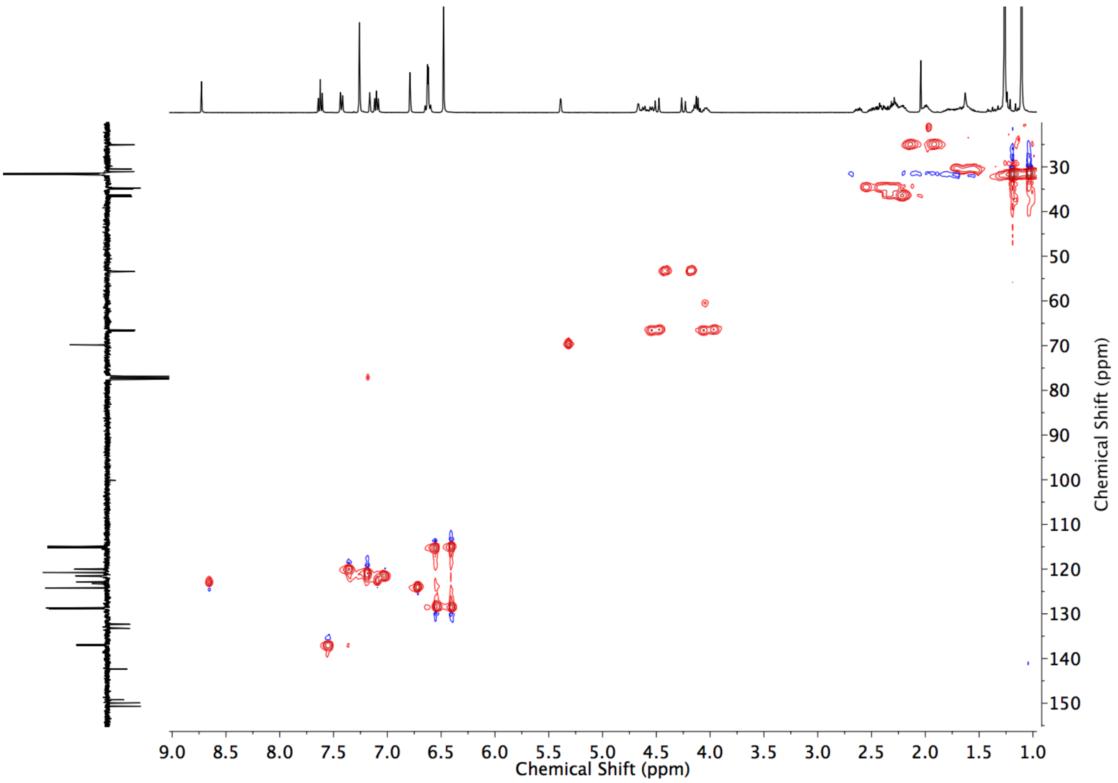

Figure S75 HSQC NMR ($CDCl_3$) of **S6**

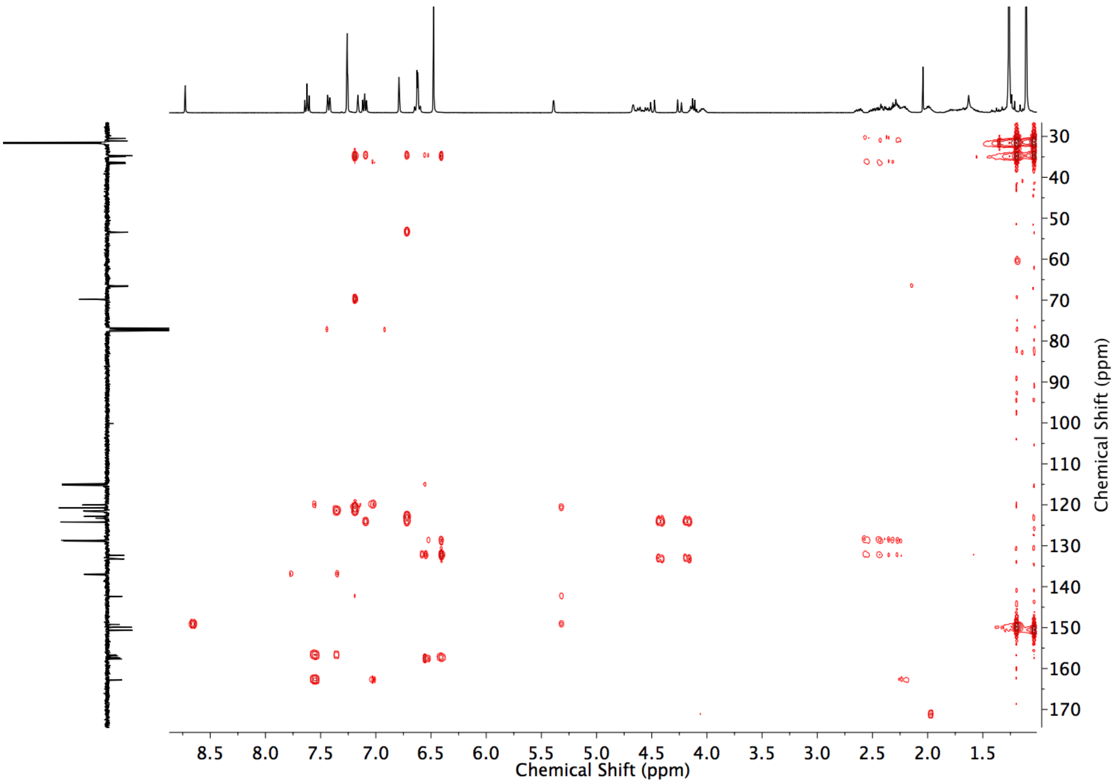

Figure S76 HMBC NMR ($CDCl_3$) of **S6**

Rotaxane **S7**

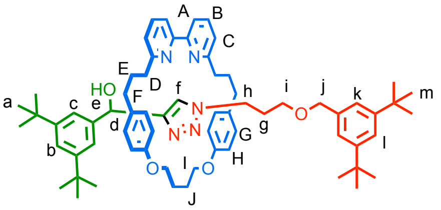

Prepared according to **general procedure B** with **1a** (12.0 mg, 0.025 mmol), $[Cu(MeCN)_4]PF_6$ (8.9 mg, 0.024 mmol), **2c** (9.1 mg, 0.03 mmol), and **3a** (7.3 mg, 0.03 mmol). After purification by column chromatography on silica (petrol with a gradient of 0 to 50% Et_2O) **S7** was obtained as a white foam (20.0 mg, 77%). ^{1}H NMR (400 MHz, $CDCl_3$): δ 8.32 (s, 1H, H_f), 7.63 (td, *J* =7.8, 2.6, 2H, H_B), 7.51 (d, *J* =7.7, 2H, H_A), 7.32 (t, *J* =1.9, 1H, H_l), 7.29 (t, *J* =1.9, 1H, H_b), 7.24 (t, *J* =1.9, 2H, H_c), 7.12 (d, *J* =7.7, H_C), 7.03 (d, *J* =1.9, 2H, H_k), 6.68 – 6.57 (m, 8H, H_G, H_H), 5.74 (d, *J* =4.5, 1H, H_d), 4.42 – 4.30 (m, 2H, 2 of H_I), 4.04 (s, 2H, H_j), 4.02 – 3.94 (m, 2H, 2 of H_J), 3.24 (d, *J* =4.7, 1H, H_e), 2.89 (t, *J* =6.7, 2H, H_i), 2.63 – 2.51 (m, 2H, H_g), 2.21 – 2.03 (m, 2H, 2 of H_J), 1.99 – 1.84 (m, 2H, 2 of H_J), 1.77 – 1.64 (m, 4H, H_E), 1.31 (s, 18H, H_a or H_m), 1.23 (s, 18H, H_a or H_m), 1.04 – 0.88 (m, 2H, H_h). ^{13}C NMR (101 MHz, $CDCl_3$) δ 162.8, 162.8, 157.6, 157.6, 157.5 (×2), 150.7, 150.7, 149.8, 142.2, 137.9, 137.0, 136.9, 133.0, 133.0, 129.4 (×2), 123.0, 122.0, 122.0, 121.7, 121.6, 121.6, 120.4, 120.4, 120.4, 115.1, 115.0, 73.0, 70.4, 67.6, 66.5 (×2), 46.7, 37.0 (×2), 36.8 (×2), 34.9, 34.9, 31.8, 31.7, 31.6, 28.8, 24.9, 24.9 (×2). LR-ESI-MS *m/z* = 1026.68 $[M+H]^+$ (calc. for $C_{67}H_{88}N_5O_4$ 1026.68).

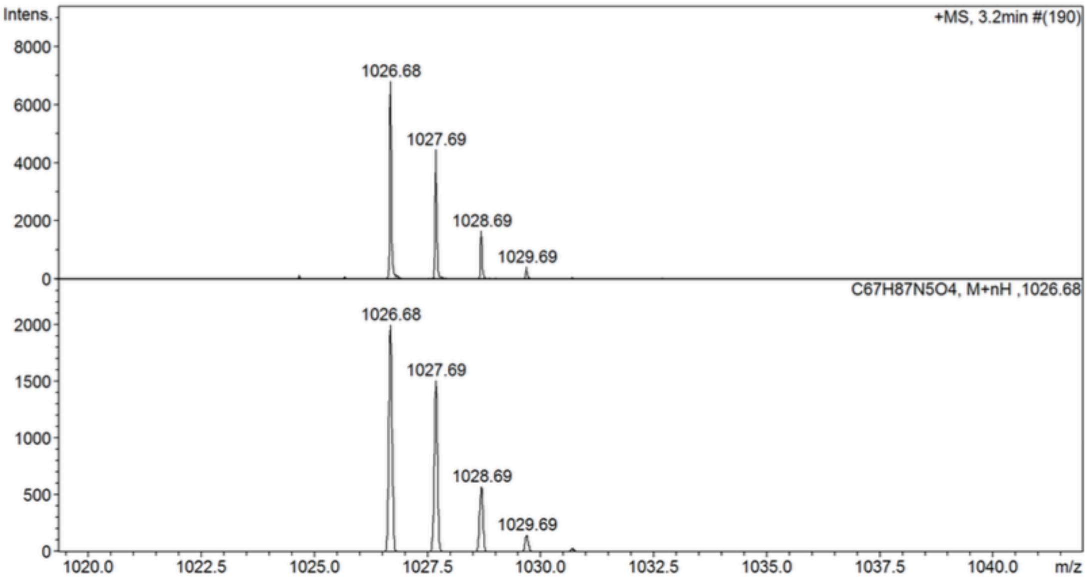

Figure S77 Observed (top) and calculated (bottom) isotopic patterns for $[M+H]^+$ of **S7**

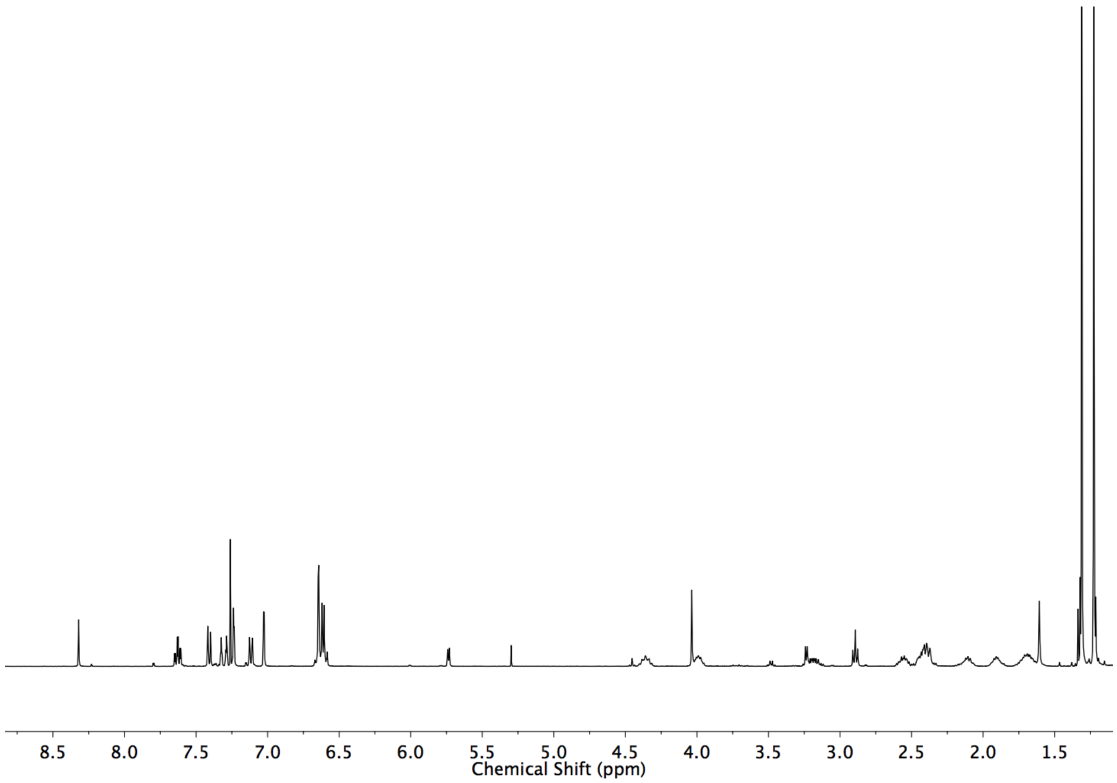

Figure S78 ^{1}H NMR ($CDCl_3$, 400 MHz) of **S7**

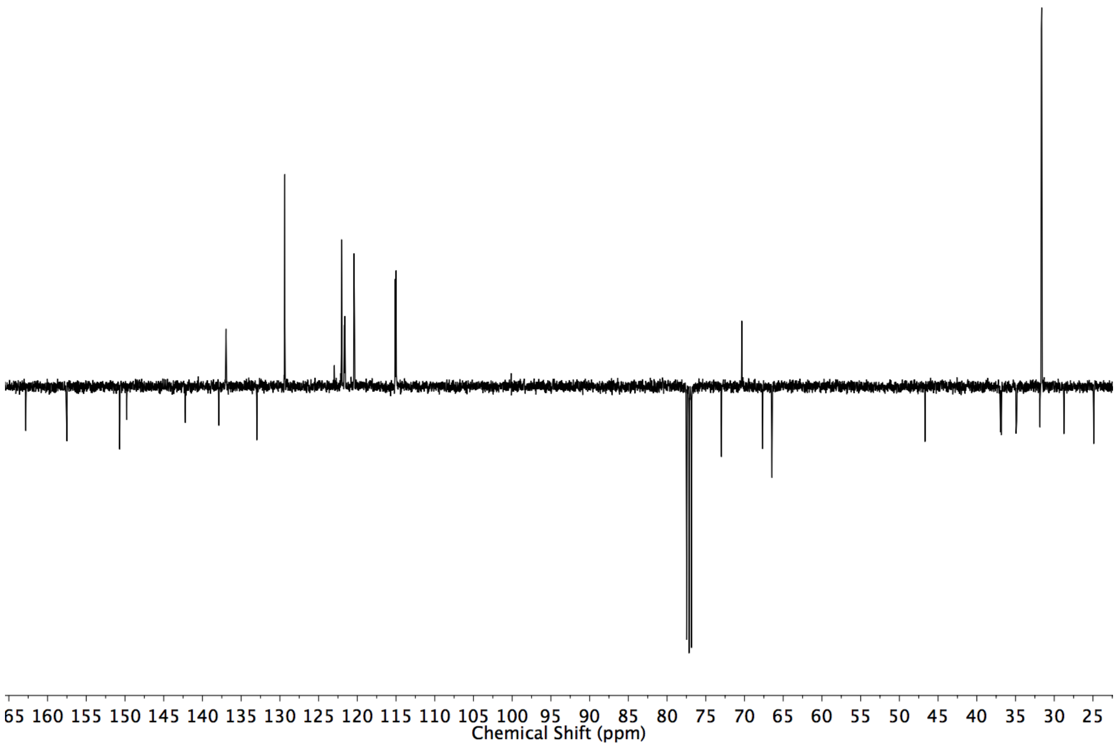

Figure S79 JMOD NMR ($CDCl_3$, 101 MHz) of **S7**

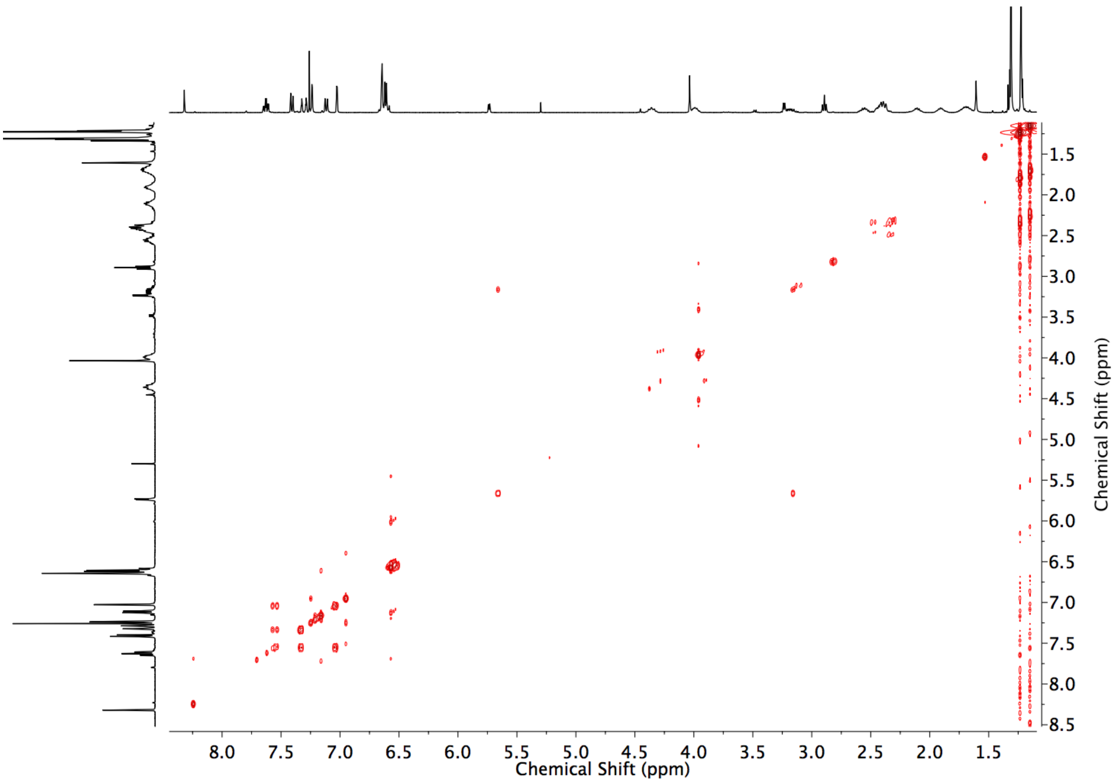

Figure S80 COSY NMR ($CDCl_3$) of **S7**

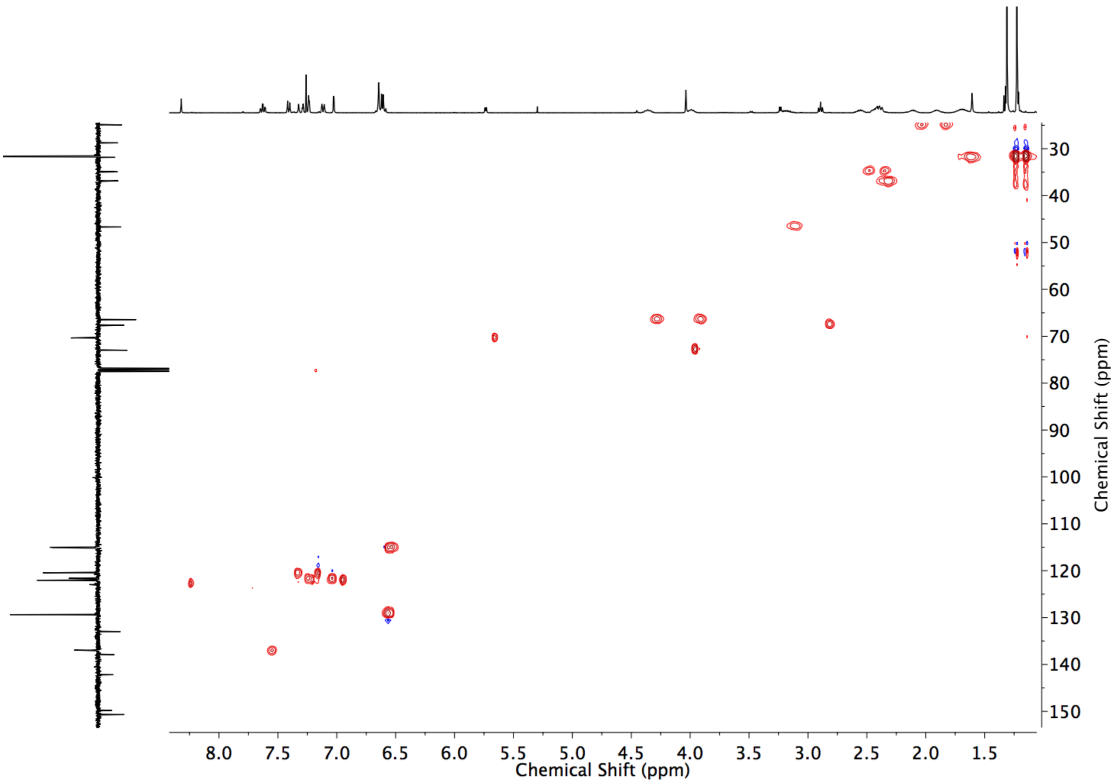

Figure S81 HSQC NMR ($CDCl_3$) of **S7**

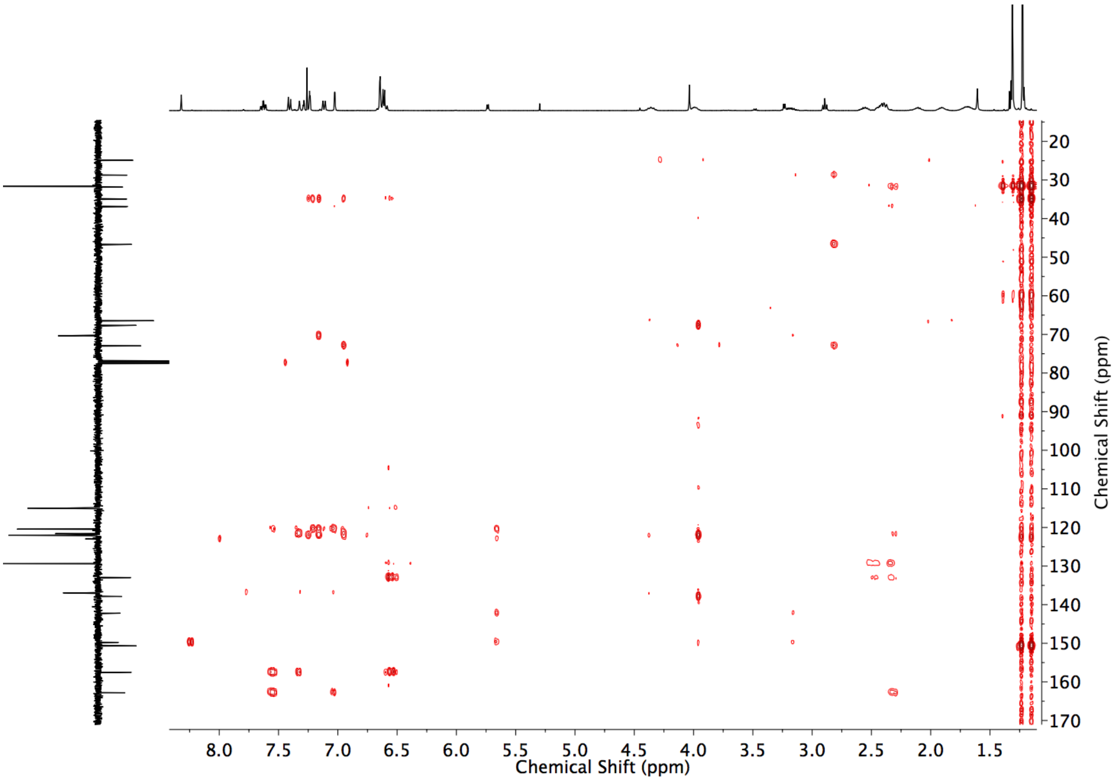

Figure S82 HMBC NMR ($CDCl_3$) of **S7**

Rotaxane **S8**

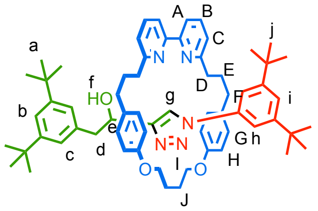

Prepared according to **general procedure B** vwith **1a** (19.1 mg, 0.04 mmol), $[Cu(MeCN)_4]PF_6$ (14.1 mg, 0.038 mmol), **2a** (10.4 mg, 0.045 mmol), and **3b** (11.6 mg, 0.045 mmol). Chromatography (petrol with a gradient of 0 to 30% Et_2O) gave **S8** as a white foam (31.0 mg, 80%). ^1H NMR (400 MHz, $CDCl_3$) δ: 10.13 (s, 1H, H_g), 7.66 (t, *J* =7.8, 2H, H_B), 7.51 (d, *J* =1.9, 2H, H_c), 7.44 (td, *J* =8.0, 0.9, 2H, H_A), 7.29 (t, *J* =1.9, 1H, H_i), 7.21 (t, *J* =1.9, 1H, H_b), 7.14 (dt, *J* =8.0, 0.9, 2H, H_C), 6.95 (d, *J* =1.9, 2H, H_c), 6.70 (s, 4H, 2 of H_G, 2 of H_H), 6.58 – 6.48 (m, 4H, 2 of H_G, 2 of H_H), 4.63 – 4.23 (m, 5H, H_I, H_e), 3.78 (s, 1H, H_f), 2.70 – 2.63 (m, 1H, 1 of H_d), 2.63 – 2.26 (m, 8H, H_D, H_F), 2.26 – 2.10 (m, 4H, H_J), 1.98 (dd, *J* =14.6, 11.2, 1H, 1 of H_d), 1.90 – 1.64 (m, 4H, H_E), 1.32 (s,18H, H_a), 1.21 (s, 18H, H_j). ^{13}C NMR (101 MHz, $CDCl_3$) δ 163.4, 163.4, 157.7, 157.7, 157.1, 157.1, 151.9, 149.8 (×2), 138.7 (×2), 137.4, 137.2, 137.1, 132.1, 128.8, 128.7, 123.7, 122.7, 122.0, 122.0, 121.0, 120.1, 120.0, 119.9, 115.4, 114.8, 114.4, 66.6, 66.6, 66.5, 43.6, 36.8 (×2), 35.4, 35.4, 35.2, 34.9, 32.0, 31.8, 31.4, 31.3, 25.1, 24.9. HR-ESI-MS *m/z* = 968.6392 $[M+H]^+$ (calc. for $C_{64}H_{82}N_5O_3$ 968.6412).

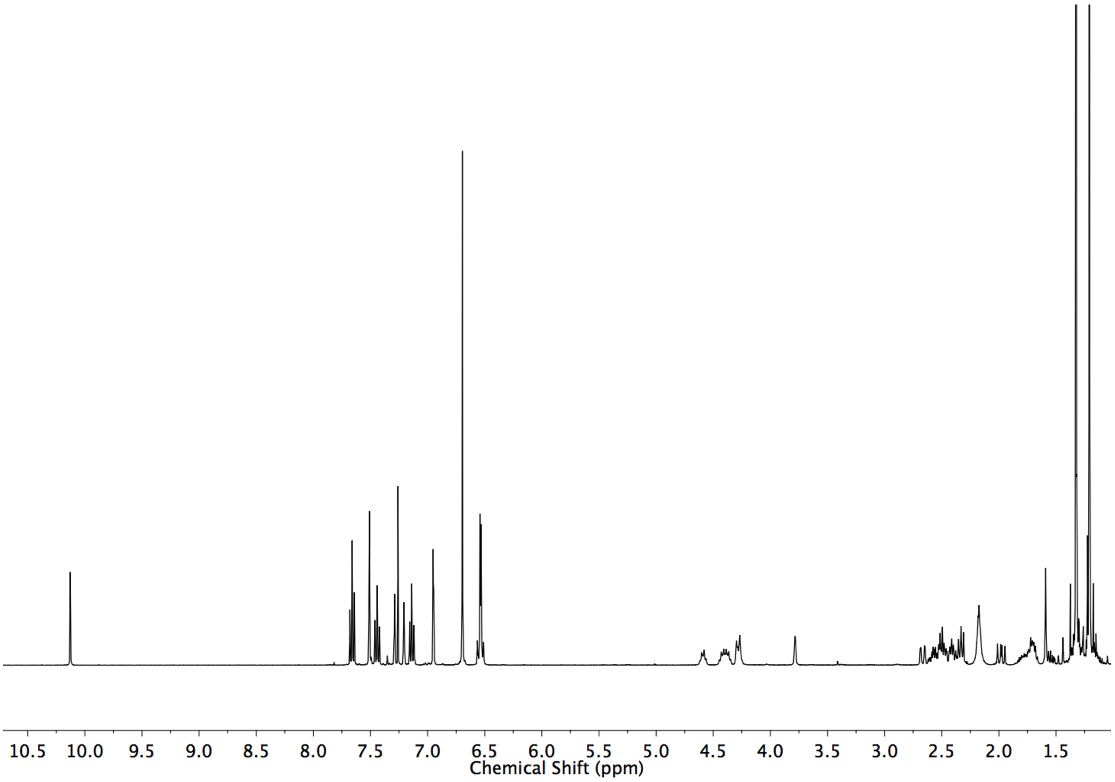

Figure S83 ^1H NMR ($CDCl_3$, 400 MHz) of **S8**

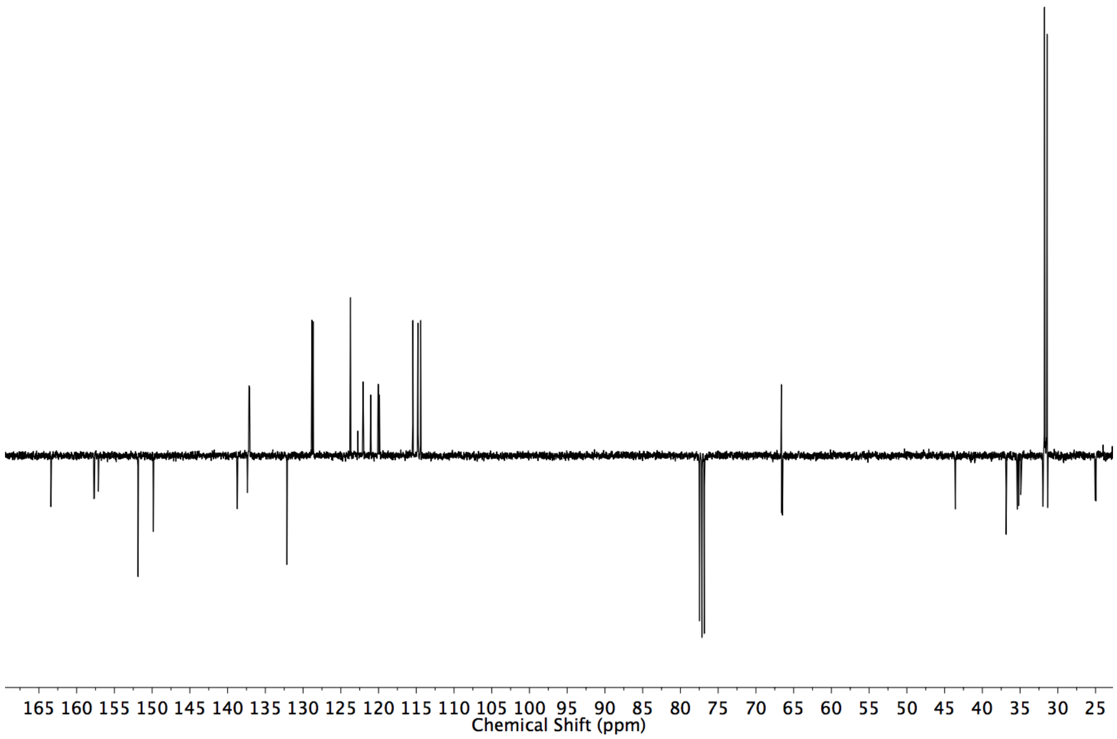

Figure S84 JMOD NMR ($CDCl_3$, 101 MHz) of **S8**

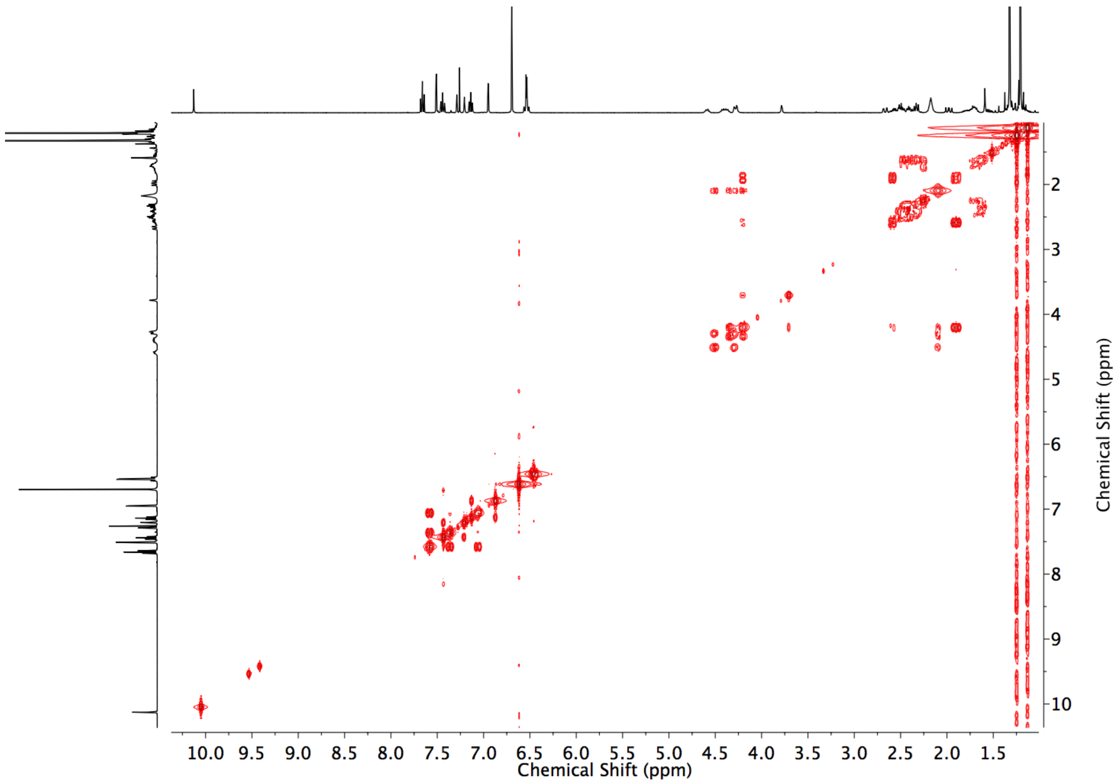

Figure S85 COSY NMR ($CDCl_3$) of **S8**

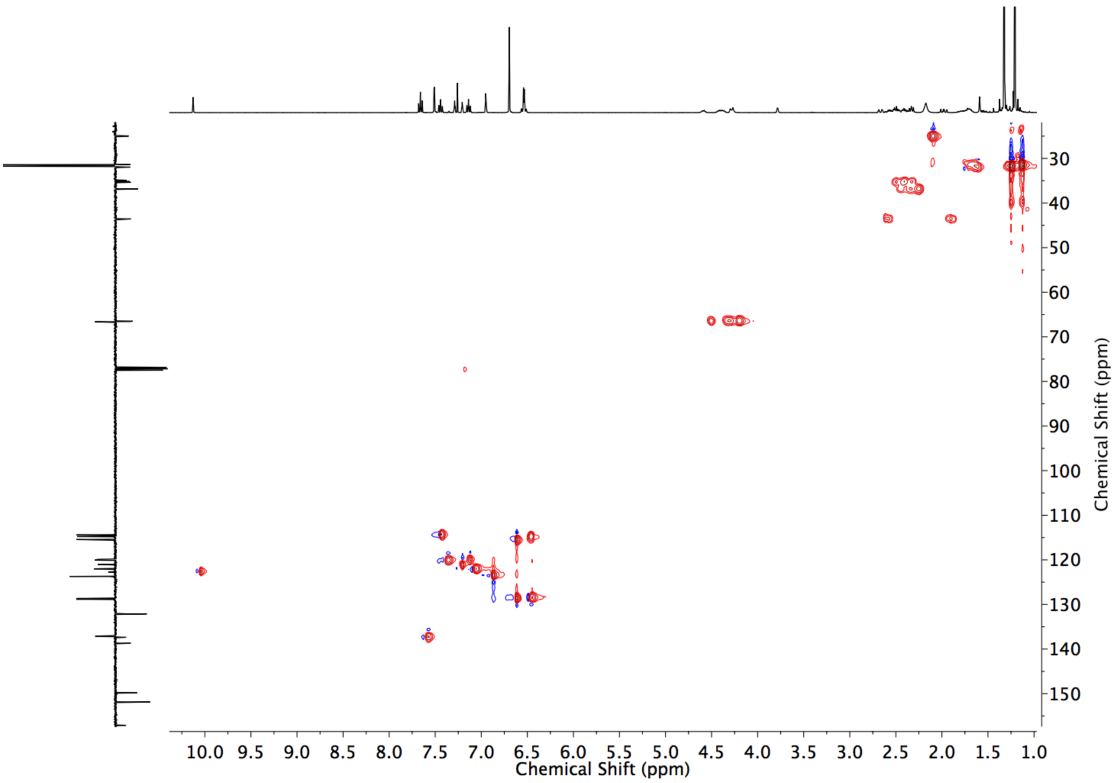

Figure S86 HSQC NMR ($CDCl_3$) of **S8**

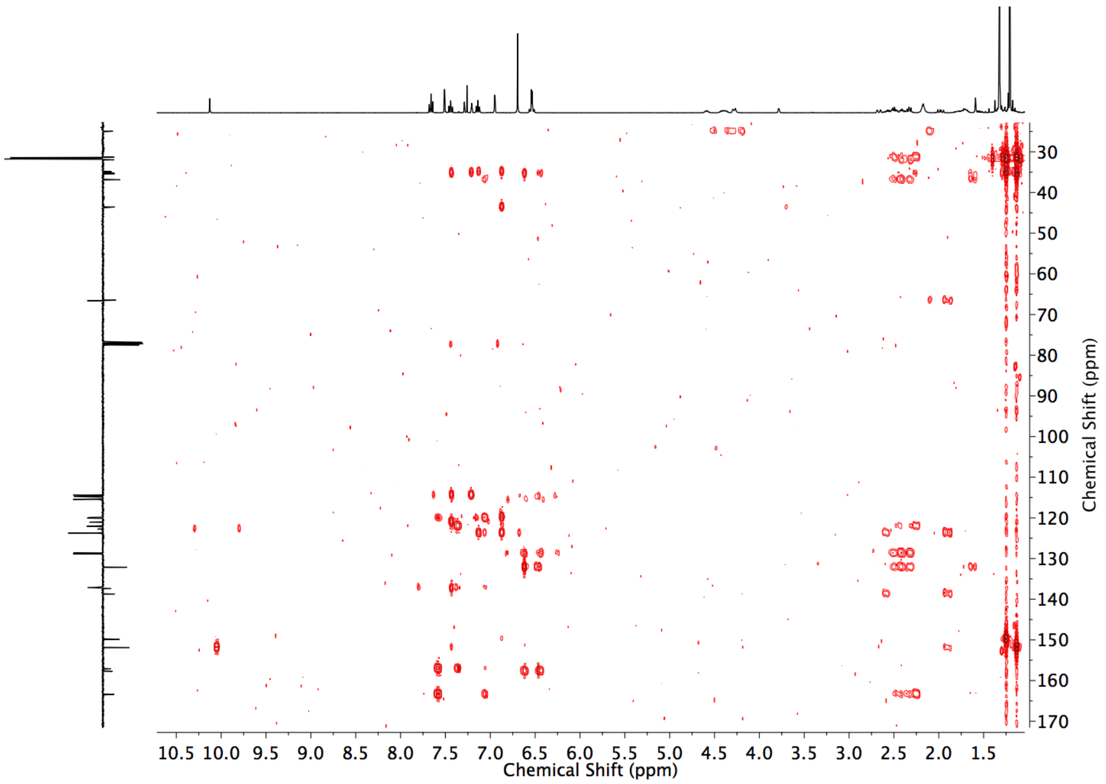

Figure S87 HMBC NMR ($CDCl_3$) of **S8**

Rotaxane **S9**

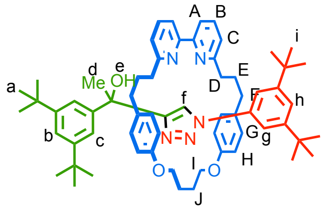

${}^{i}Pr_2NEt$ (9.0 µl, 0.05 mmol) was added to a solution of **3c** (6.5 mg, 0.025 mmol), **2a** (5.8mg, 0.025 mmol), **1a** (12.0 mg, 0.025 mmol) and $[Cu(MeCN)_4]PF_6$ (8.9 mg, 0.024 mmol) in CH_2Cl_2 (2.0 mL) in a microwave vial. The deep red mixture was stirred at rt for 16 h. TBACN (26.0 mg, 0.097 mmol) in CH_2Cl_2 was added to the mixture and stirred for five days at rt until the solution turned black. N_2 was bubbled through the reaction mixture to remove the solvent. The residue was diluted with CH_2Cl_2 (20 mL), and washed with H_2O (10 mL). The aqueous layer was extracted with CH_2Cl_2 (2 × 10 mL). The combined organic extracts were washed with brine (10 mL), dried ($MgSO_4$), filtered and the solvent removed *in vacuo*. Chromatography (petrol:CH_2Cl_2 1:1, with a gradient of 0 to 5% acetonitrile) gave **S9** as a white foam (20.0 mg, 84%).^{1}H NMR (400 MHz, $CDCl_3$) δ: 10.06 (s, 1H, H_f), 7.72 (app t, *J* = 7.8 1H, 1 of H_B), 7.68 (app t, *J* =7.7, 1H, one of H_B), 7.56 (d, *J* = 1.8, 2H, H_c), 7.52 (dd, *J* = 7.8, 0.9, 1H, one of H_A), 7.50 – 7.46 (m, 3H, H_g, one of H_A), 7.30 (t, *J* = 1.8, 1H, H_b), 7.18-7.10 (m, 3H, H_h, H_C), 6.48 (d, *J* = 8.6, 2H, 2 of H_G), 6.27 (d, *J* = 8.6, 2H, 2 of H_G) 6.20 (d, *J* = 8.6, 2H, 2 of H_H) 6.01 (d, *J* = 8.6, 2H, 2 of H_H), 4.81 (q, *J* = 7.9, 1H, 1 of H_I), 4.66 (q, *J* = 7.9, 1H, 1 of H_I), 4.19 – 4.10 (m, 1H, 1 of H_I) 4.03 – 3.81 (m, 2H, H_e, 1 of H_I), 2.52 – 1.92 (m, 12H, H_D, H_F, H_J), 1.70-1.50 (m, 7H, H_d, H_E), 1.24 (s, 18H, H_a), 1.09 (s, 18H, H_i). ^{13}C NMR (101 MHz, $CDCl_3$) δ 163.4, 163.4, 157.5, 157.3, 156.9, 153.4, 150.9, 150.9, 149.7, 137.1, 137.1, 137.0, 131.6, 131.5, 128.2, 122.1, 121.8, 121.1, 120.7, 120.4, 120.2,120.0, 129.4, 115.2, 113.6, 114.4, 72.3, 66.3, 66.3, 37.0, 36.8, 35.2, 35.1, 35.1, 31.8, 31.5, 31.4, 31.0, 25.0, 24.3. HR-ESI-MS *m/z* =968.6397 [M+H]$^+$ (calc. for $C_{64}H_{82}N_5O_3$ 968.6412).

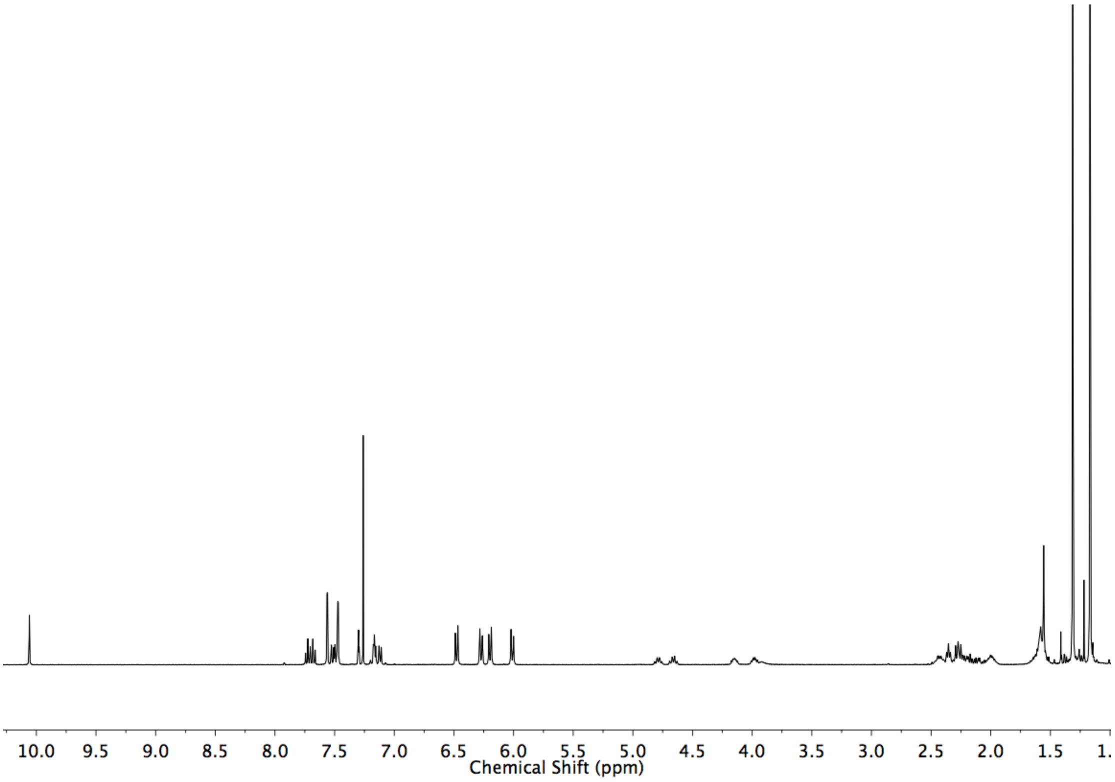

Figure S88 ^{1}H NMR ($CDCl_3$, 400 MHz) of **S9**

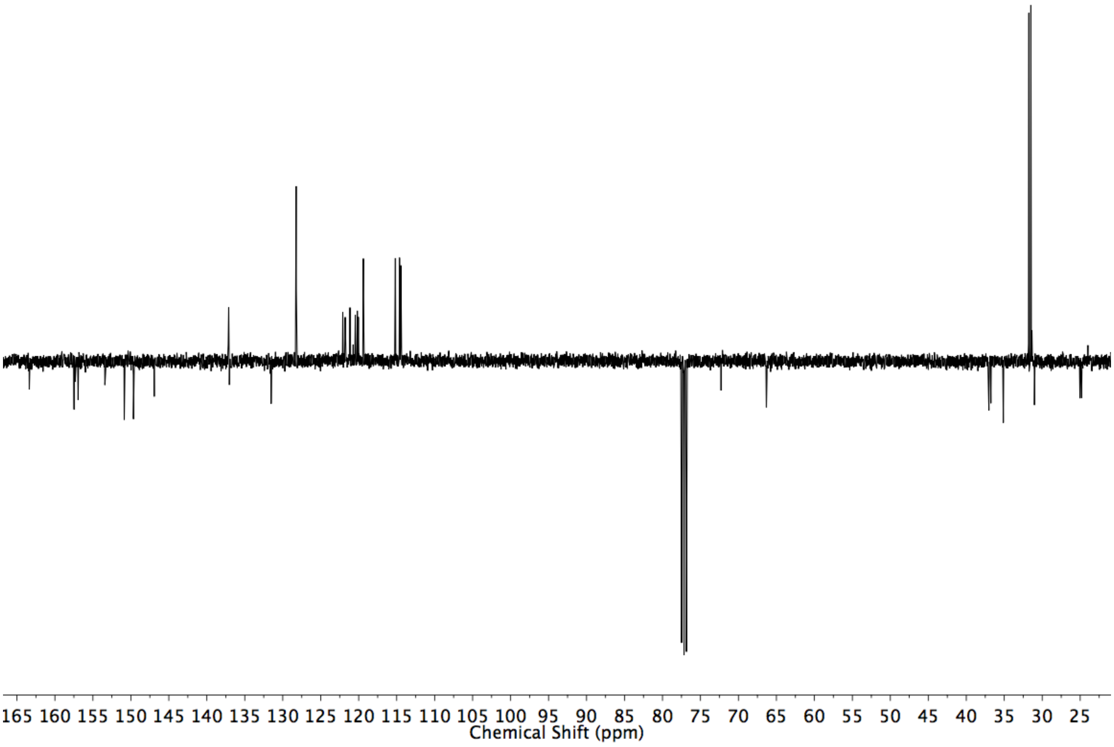

Figure S89 ^{13}C NMR ($CDCl_3$, 101 MHz) of **S9**

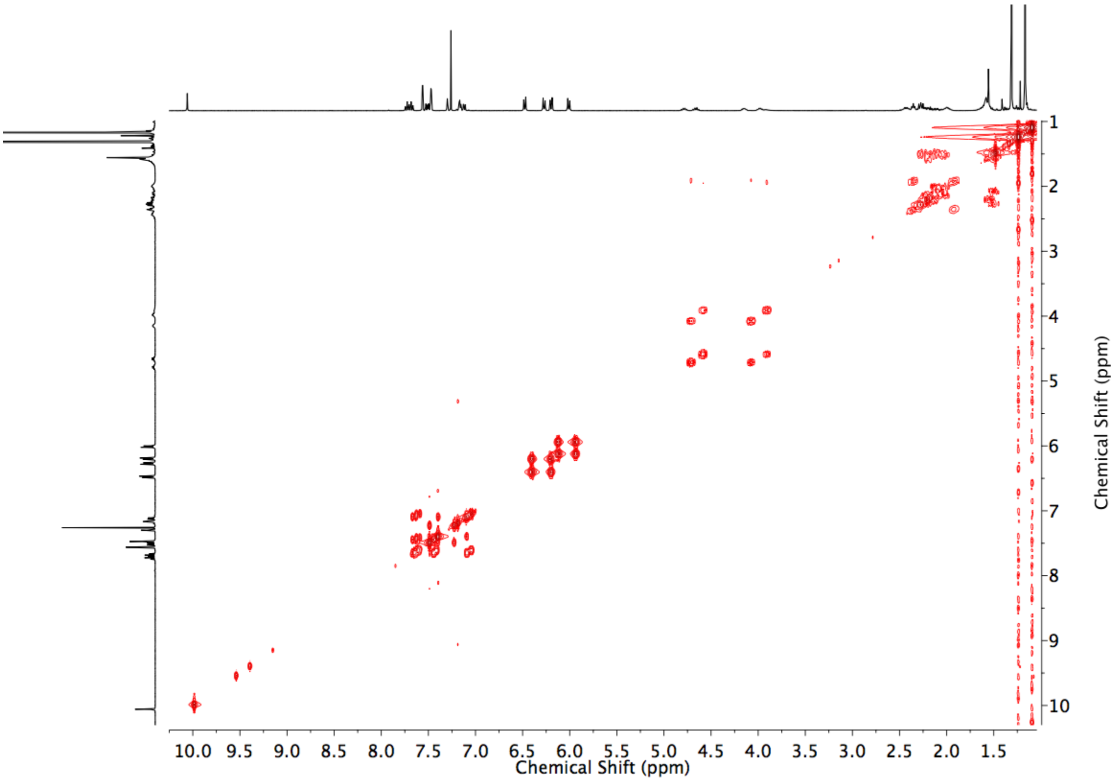

Figure S90 COSY NMR ($CDCl_3$) of **S9**

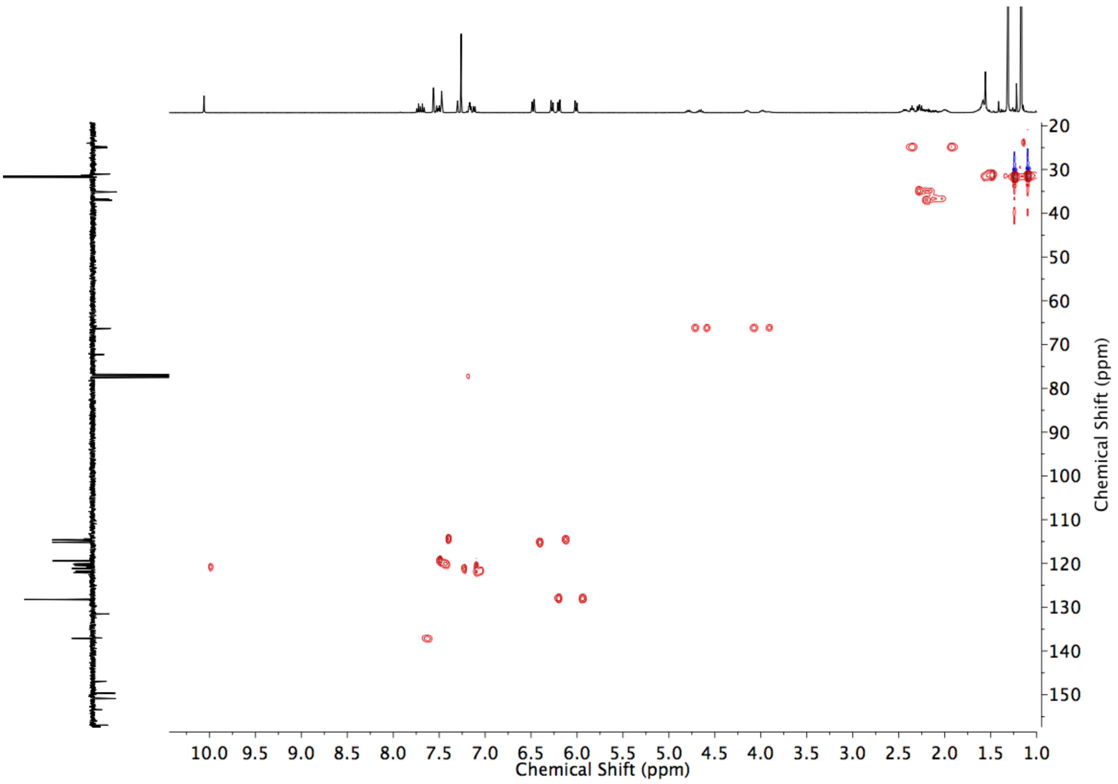

Figure S91 HSQC NMR ($CDCl_3$) of **S9**

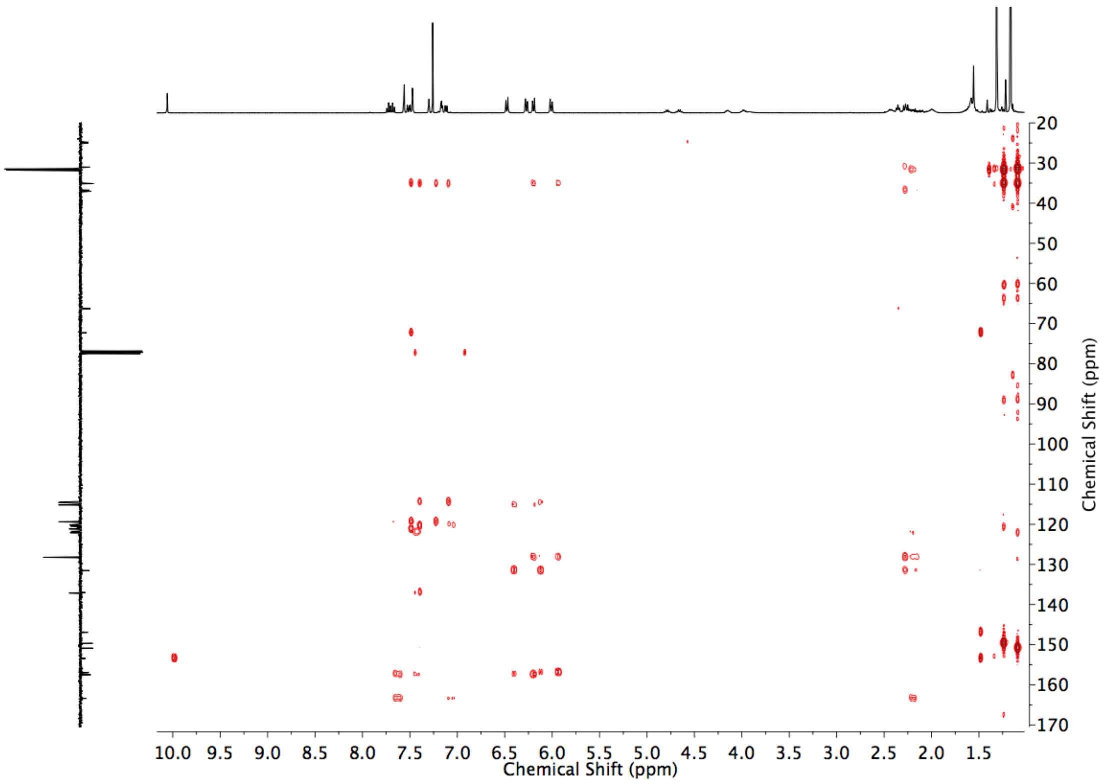

Figure S92 HMBC NMR ($CDCl_3$) of **S9**

Rotaxane **S10**

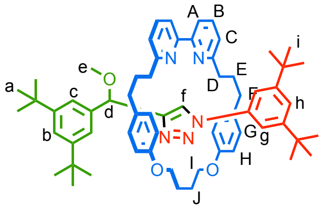

^{i}Pr$_2$NEt (9.0 µl, 0.05 mmol) was added to a solution of **3d** (6.5 mg, 0.025 mmol), **2a** (5.8mg, 0.025 mmol),**1a** (12.0 mg, 0.025 mmol), and [Cu(MeCN)$_4$]PF$_6$ (8.9 mg, 0.024 mmol), in CH_2Cl_2 (2.0 mL) in a sealed microwave vial (CEM Ltd.). The deep red mixture was stirred at rt for 16 h. KCN (10.0 mg, 0.153 mmol) in MeOH was added and the reaction mixture and stirred for two days at rt until the solution turned black. N_2 was bubbled through the reaction mixture to remove the solvent. The residue was diluted with CH_2Cl_2 (20 mL) and washed with H_2O (10 mL). The aqueous layer was extracted with CH_2Cl_2 (2 × 10 mL). The combined organic extracts were washed with brine (10 mL), dried ($MgSO_4$), filtered and the solvent removed *in vacuo*. Chromatography (petrol with a gradient of 0 to 50% EtOAc) gave **S10** as a white foam (4.7 mg, 19%). Decomposition to unidentified species was observed during purification, accounting for the low isolated yield. ^{1}H NMR (500 MHz, $CDCl_3$) δ: 10.24 (s, 1H, H_f), 7.73 (t, *J* =7.8, 1H, 1 of H_B), 7.69 (t, *J* = 7.8, 1H, 1 of H_B), 7.60 (d, *J* = 1.7, 2H, H_c), 7.54 (ddd, *J* = 7.8, 2.7, 0.9, 2H, H_A), 7.25 (t, *J* = 2.7, 1H, H_h), 7.21 (d, *J* = 2.7, 2H, H_g), 7.18 (t, *J* = 2.7, 1H, H_b), 7.12 (dd, *J* = 7.8, 1.0, 1H, 1 of H_C), 7.07 (dd, *J* = 7.8, 1.0, 1H, 1 of H_C), 6.44 (d, *J* = 8.6, 2H, 2H, 2 of H_H), 6.28 (d, *J* = 8.6, 2H, 2 of H_H), 6.18 (d, *J* = 8.6, 2H, 2 of H_G), 5.97 (d, *J* = 8.6, 2H, H_G), 4.91 (s, 1H, H_d), 4.89 – 4.81 (m, 1H, 1 of H_I), 4.81 – 4.73 (m, 1H, 1 of H_I), 4.19 – 4.11 (m, 1H, 1 of H_I), 4.10 – 4.06 (m, 1H, 1 of H_I), 3.00 (s, 3H , H_e), 2.48 – 1.95 (m, 2H, 2 of H_J), 2.36 – 2.25 (m, 14H, H_D, H_E, H_F, 2 of H_J), 1.17 (s, 36H, H_a, H_i). ^{13}C NMR (126 MHz, $CDCl_3$) δ 163.5, 163.5, 157.6, 157.5, 157.3, 157.1, 150.6, 150.4, 146.3, 141.0, 137.1, 137.0, 136.9, 131.5, 131.5, 128.2, 128.1, 124.2, 122.3, 121.9, 121.8, 121.0, 120.9, 119.9, 119.8, 115.21, 115.1, 114.9, 79.8, 66.7, 66.5, 56.7, 37.2, 37.0, 35.2, 35.2, 35.2, 34.9, 31.9, 31.5, 31.5, 31.4, 24.9, 24.9. HR-ESI-MS *m/z* =968.6402 [M+H]$^+$ (calc. for $C_{64}H_{82}N_5O_3$ 968.6412).

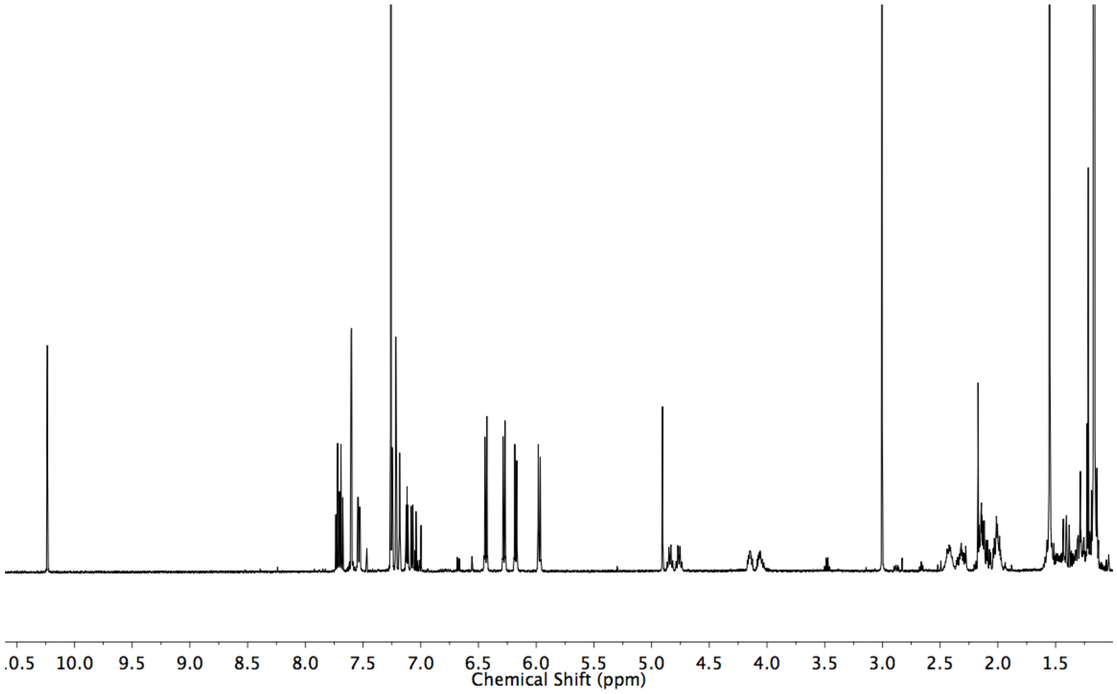

Figure S93 ^{1}H NMR ($CDCl_3$, 500 MHz) of **S10**

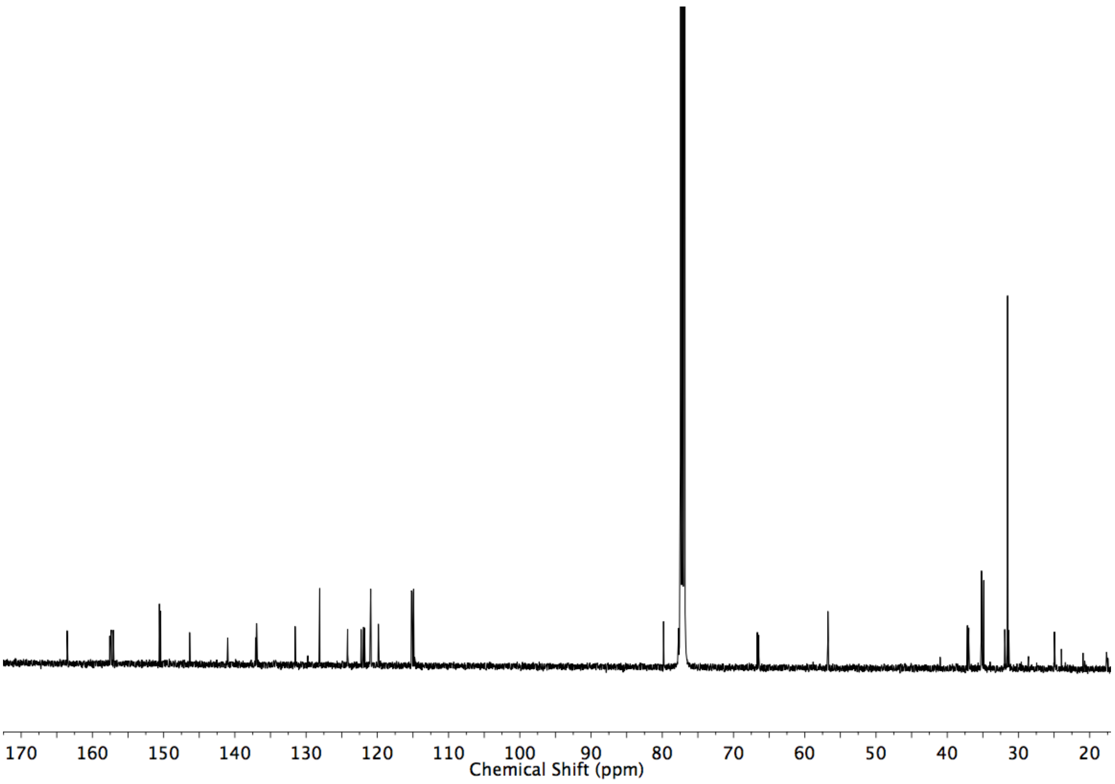

Figure S94 ^{13}C NMR ($CDCl_3$, 126 MHz) of **S10**

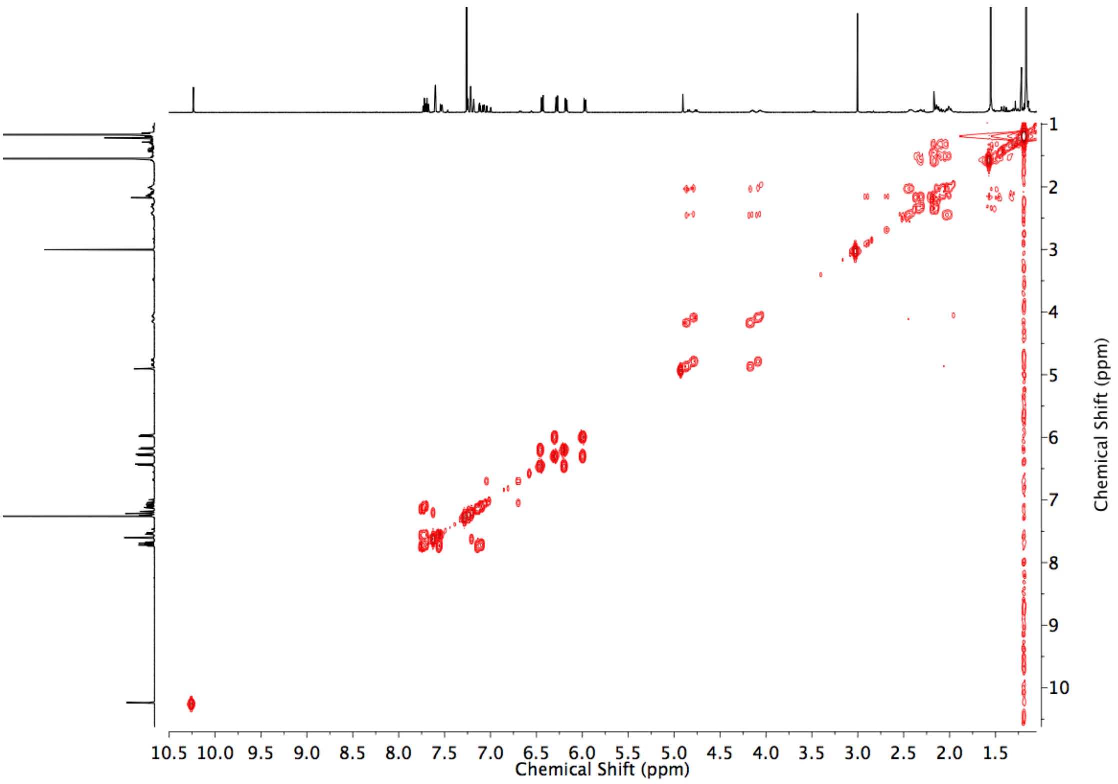

Figure S95 COSY NMR ($CDCl_3$) of **S10**

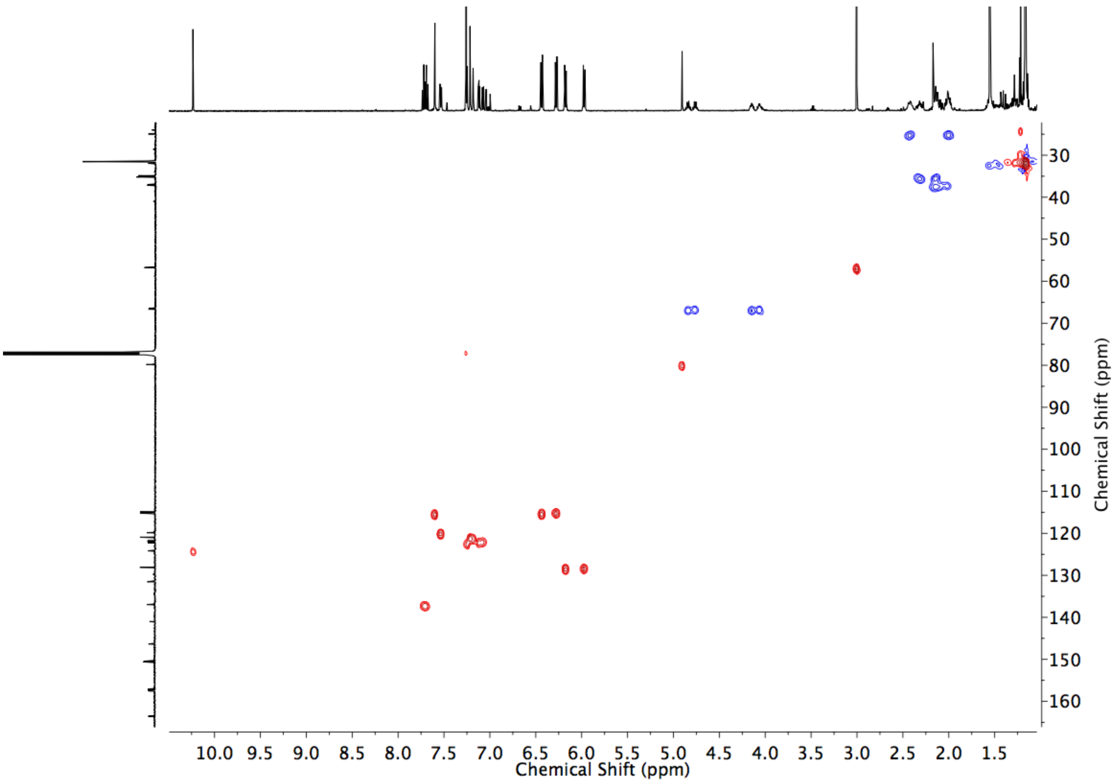

Figure S96 HSQC NMR ($CDCl_3$) of **S10**

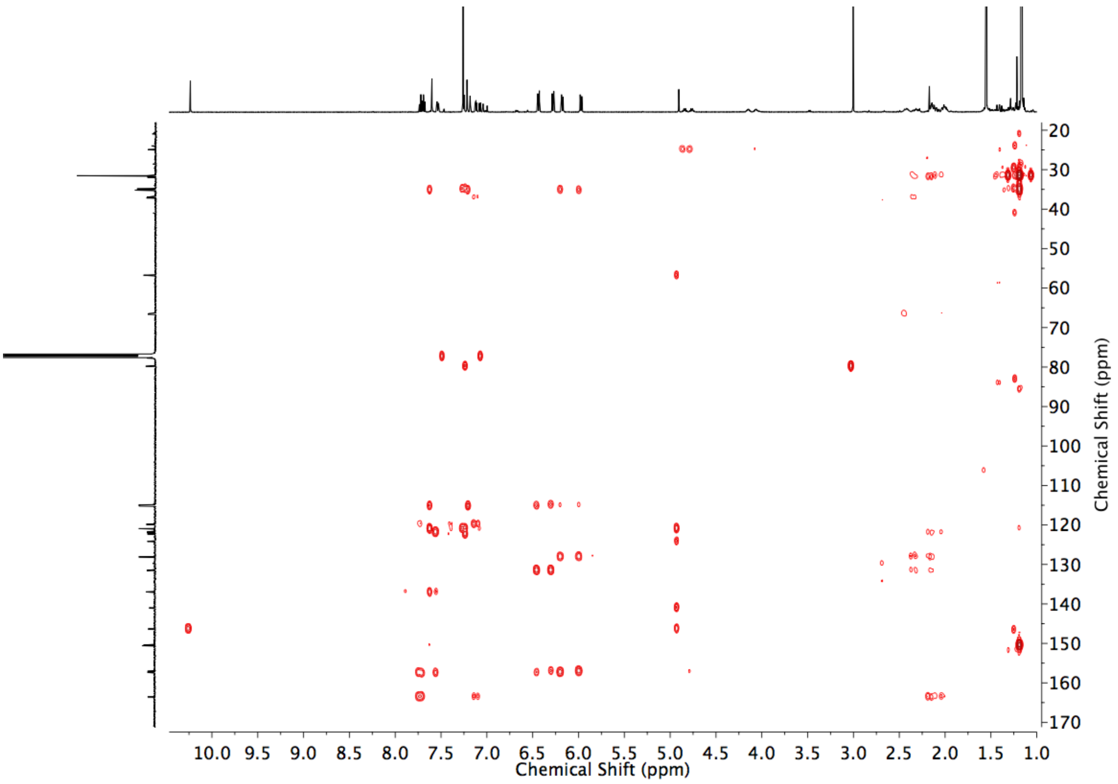

Figure S97 HMBC NMR ($CDCl_3$) of **S10**

Rotaxane **S11**

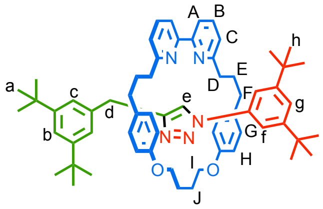

Prepared according to **general procedure B** with **1a** (12.0 mg, 0.025 mmol), $[Cu(MeCN)_4]PF_6$ (8.9 mg, 0.024 mmol), **2a** (7.0 mg, 0.03 mmol), and **3e** (7.0 mg, 0.03 mmol). Chromatography (petrol with a gradient of 0 to 40% Et_2O) gave **S11** as a white foam (17.0 mg, 72%). ^1H NMR (400 MHz, $CDCl_3$) δ: 10.09 (s, 1H, H_e), 7.69 (t, *J* =7.7, 2H, H_B), 7.51 (dd, *J* =7.8, 0.9, 2H, H_A), 7.45 (d, *J* =1.7, 2H, H_f), 7.18 (t, *J* =1.7, 1H, H_g), 7.16 (t, *J* =1.7, 1H, H_b), 7.09 (dd, *J* =7.8, 0.9, 2H, H_C), 6.95 (d, *J* =1.8, 2H, H_c), 6.40 (d, *J* =8.5, 4H, H_H), 6.16 (d, *J* =8.5, 54H, H_G), 4.72 (q, *J* =7.8, 2H, H_I), 4.26-4.09 (m, 2H, H_I), 3.45 (s, 2H, H_d), 2.45 – 1.98 (m, 12H, H_D, H_F, H_I), 1.57 – 1.29 (m, 4H, H_E), 1.16 (s, 18H, H_h), 1.12 (s, 18H, H_a). ^{13}C NMR (101 MHz, $CDCl_3$) δ: 163.5, 157.6, 157.3, 150.7, 150.5, 145.3, 139.6, 137.3, 136.9, 131.8, 128.2, 123.8, 123.1, 122.0, 120.8, 120.6, 119.7, 115.4, 115.1, 68.8, 37.0, 35.2, 35.1, 34.7, 33.2, 32.2, 31.5, 31.5, 25.0. HR-ESI-MS *m/z* = 938.6307 $[M+H]^+$ (calc. for $C_{63}H_{80}N_5O_2$ 938.6307).

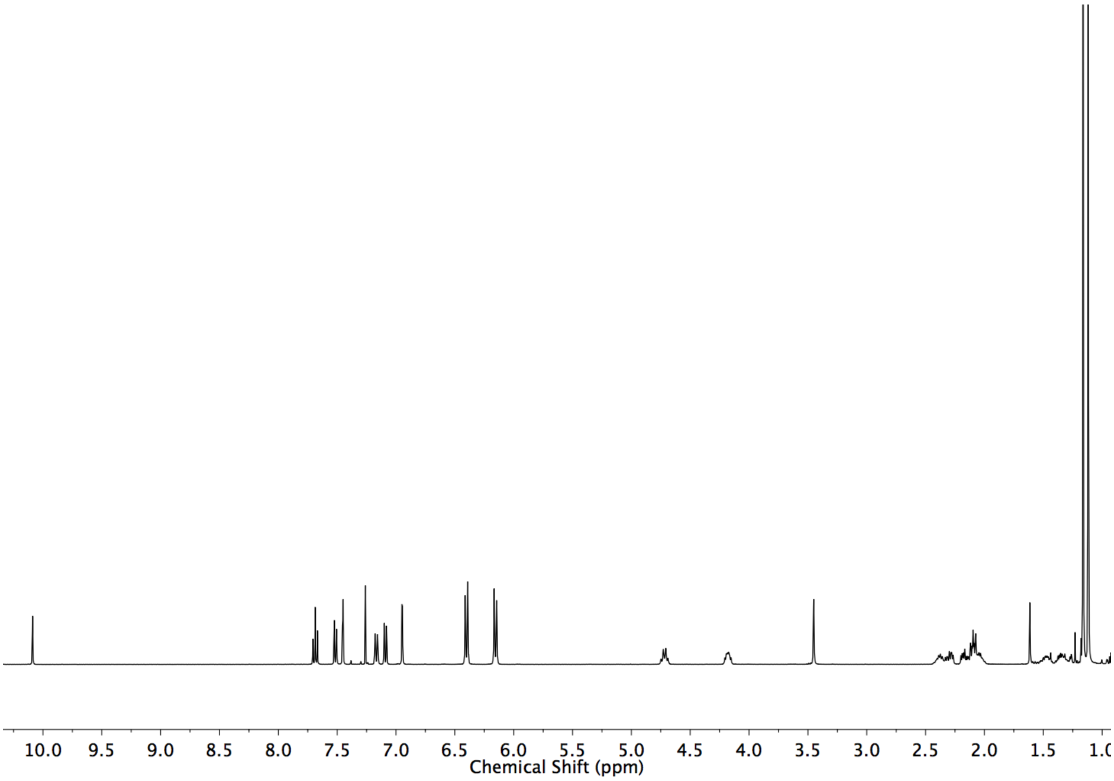

Figure S98 ^1H NMR ($CDCl_3$, 400 MHz) of **S11**

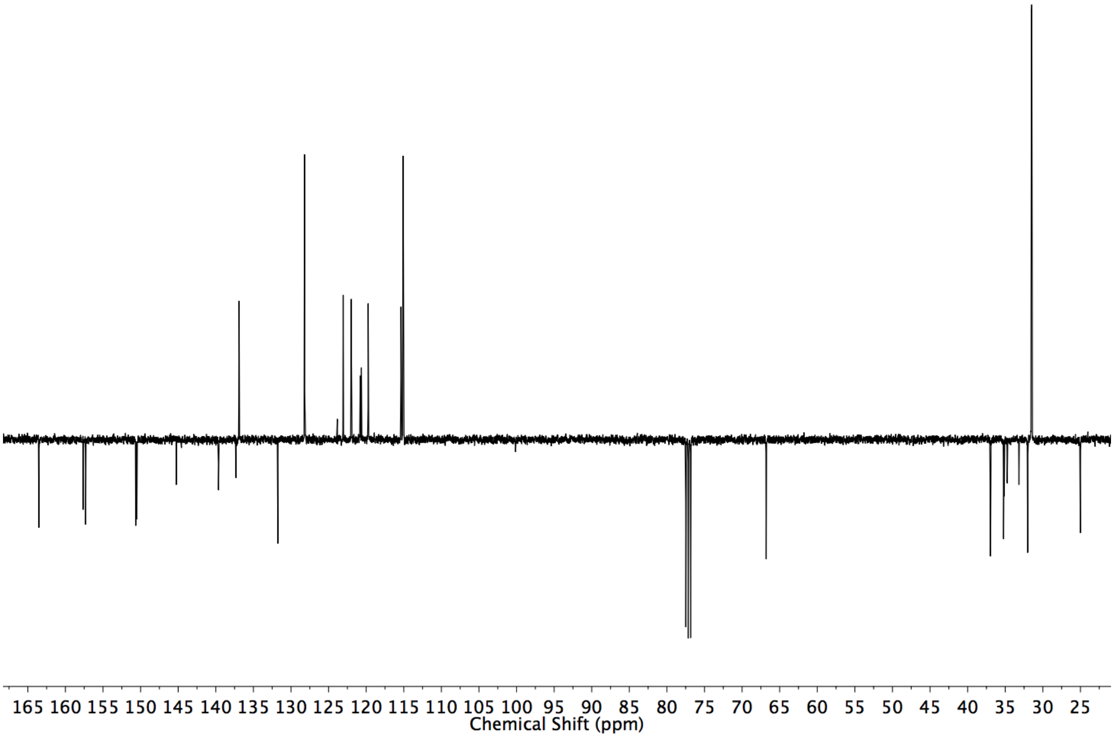

Figure S99 JMOD NMR ($CDCl_3$, 101 MHz) of **S11**

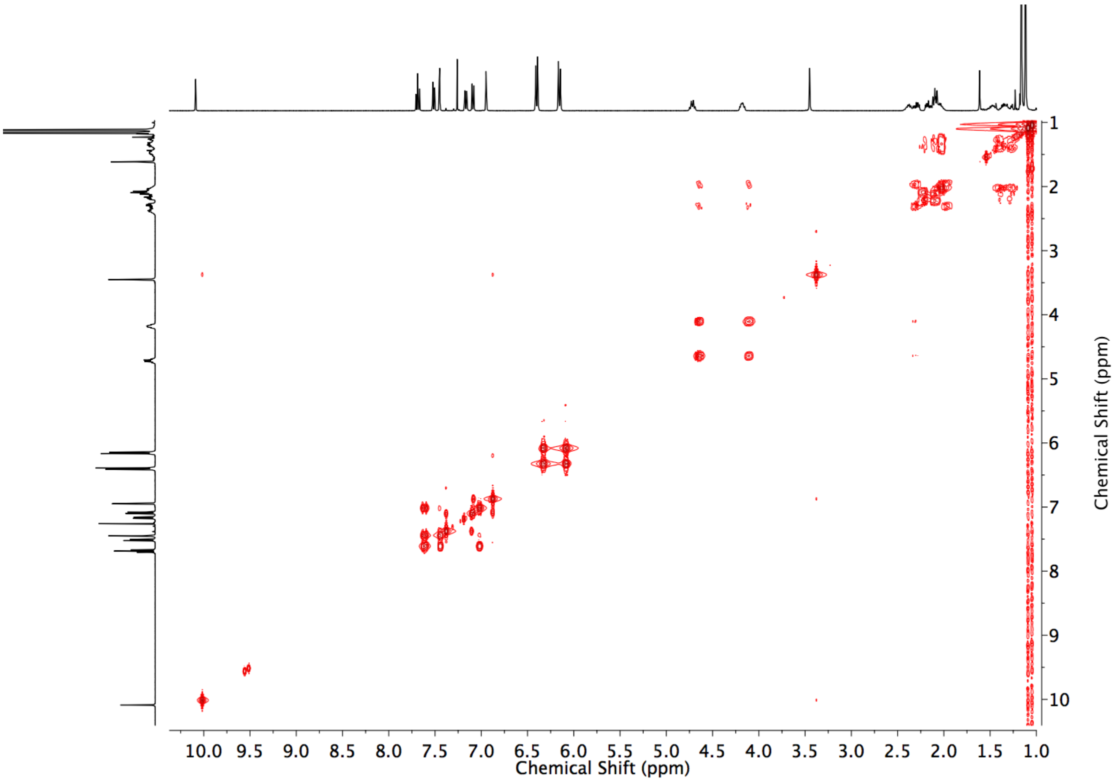

Figure S100 COSY NMR ($CDCl_3$) of **S11**

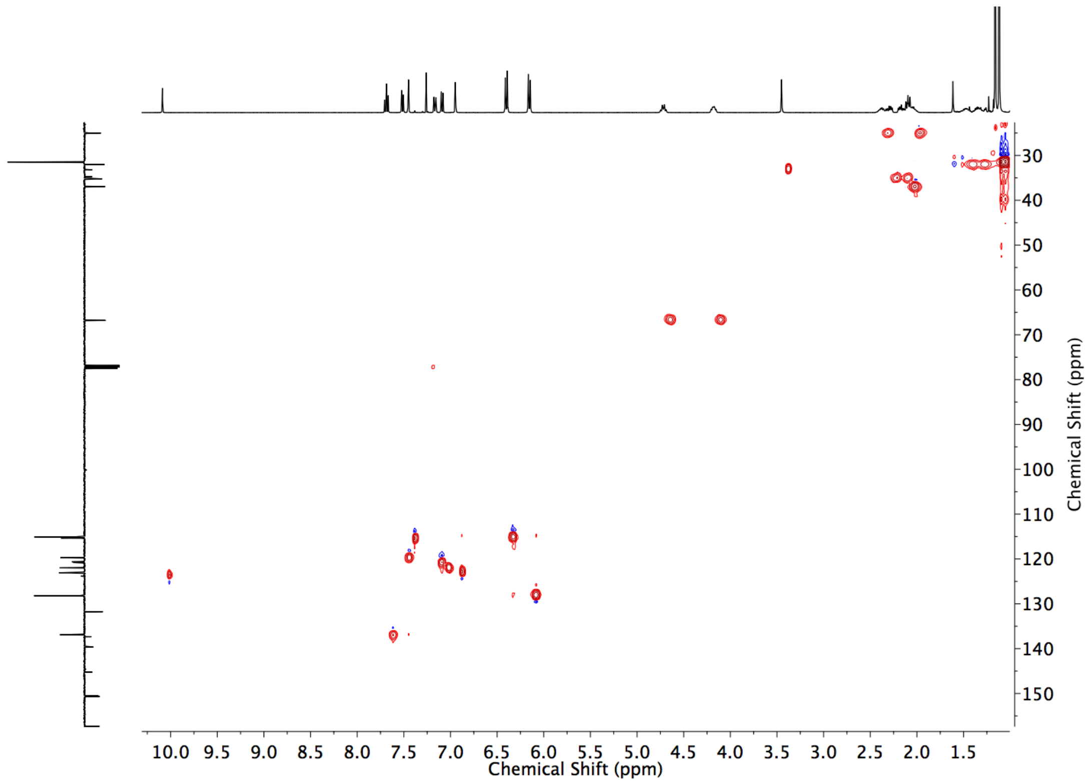

Figure S101 HSQC NMR ($CDCl_3$) of **S11**

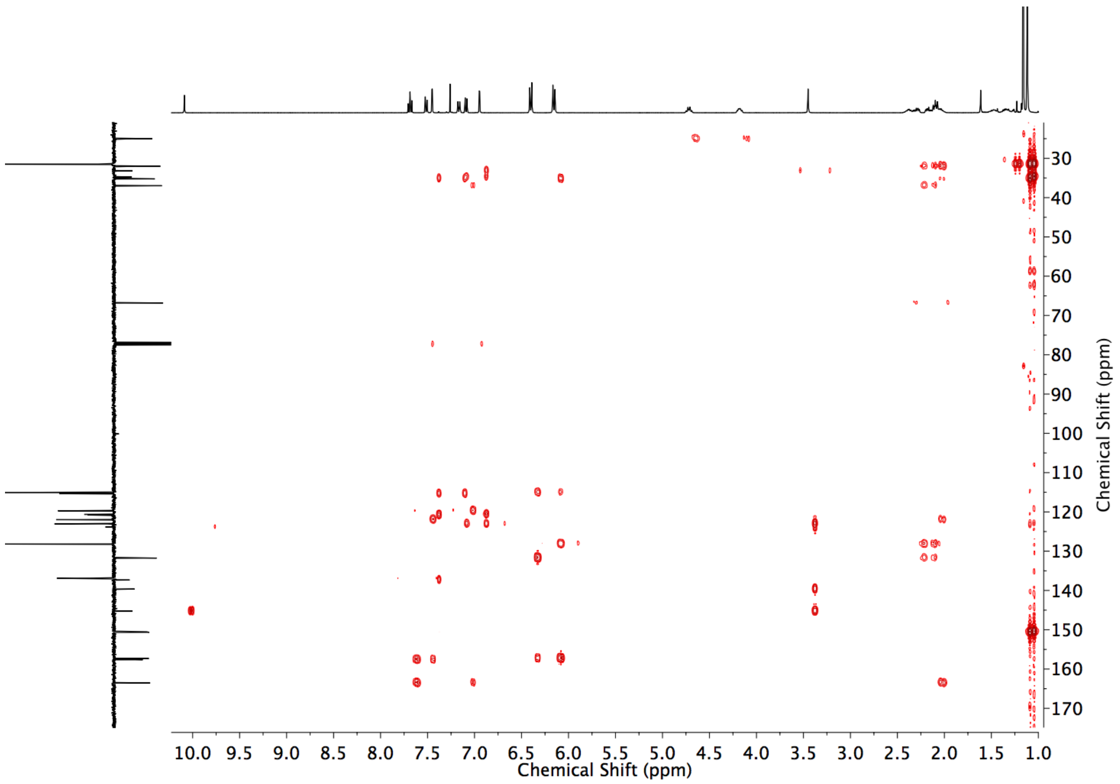

Figure S102 HMBC NMR ($CDCl_3$) of **S11**

Axle **S12**

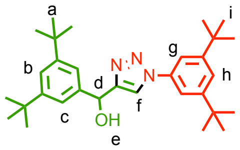

2a (24.4 mg, 0.1 mmol), **3a** (23.1 mg, 0.1 mmol), $CuSO_4.5H_2O$ (12.5 mg, 0.05 mmol), and sodium ascorbate (19.8 mg, 0.1 mmol) were placed in a round bottom flask. DMF (2mL) was added and the mixture was stirred for 16 h. The crude reaction mixture was diluted with Et_2O (10 mL), washed with H_2O (2 × 10 mL), brine (5 mL), dried ($MgSO_4$), filtered and the solvent removed *in vacuo*. Chromatography (petrol with 0 to 50% Et_2O) gave **S12** as a white foam (37.0 mg, 78%). ^{1}H NMR (400 MHz, $CDCl_3$): δ 7.74 (s, 1H, H_e), 7.48 (t, *J* =1.7, 1H, H_h), 7.47 (d, *J* =1.7, 2H, H_g) 7.40 (t, *J* =1.7, 1H, H_b), 7.38 (d, *J* =1.7, 2H, H_c), 6.11 (d, *J* =3.7, 1H, H_d), 2.85 (d, *J* =3.7, 1H, H_e), 1.35 (s, 18H, H_i), 1.33 (s, 18H, H_a). ^{13}C NMR (101 MHz, $CDCl_3$): δ 152.9, 151.9, 151.3, 141.2, 136.9, 123.1, 122.4, 121.0, 120.0, 115.7, 70.3, 35.3, 35.1, 31.6, 31.6. HR-ESI-MS *m/z* = 476.3624 $[M+H]^+$ (calc. for $C_{31}H_{46}N_3O$ 476.3635).

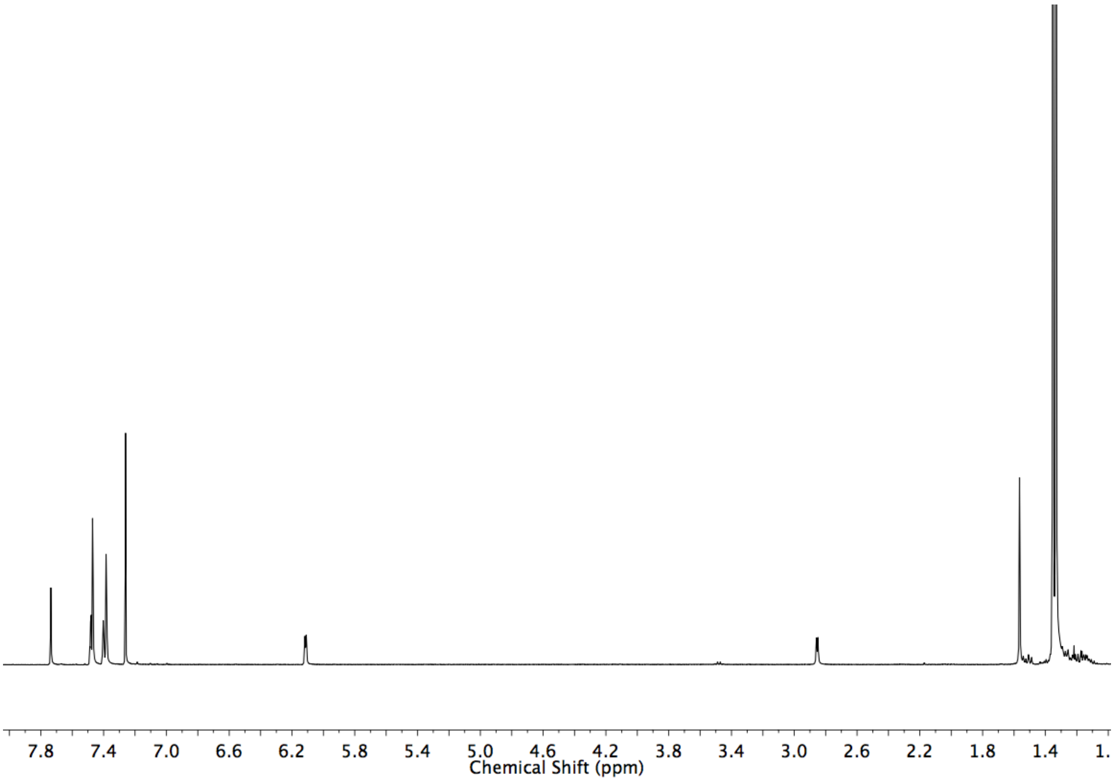

Figure S103 ^{1}H NMR ($CDCl_3$, 400 MHz) of **S12**

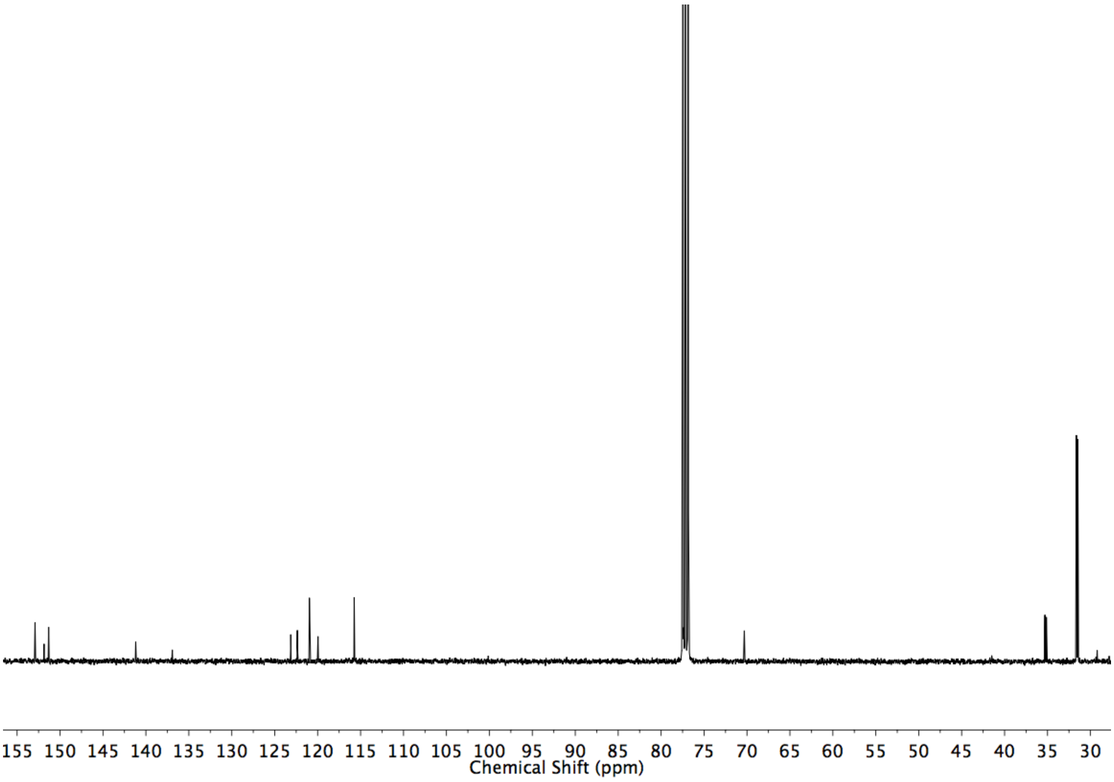

Figure S104 ^{13}C NMR ($CDCl_3$, 101 MHz) of **S12**

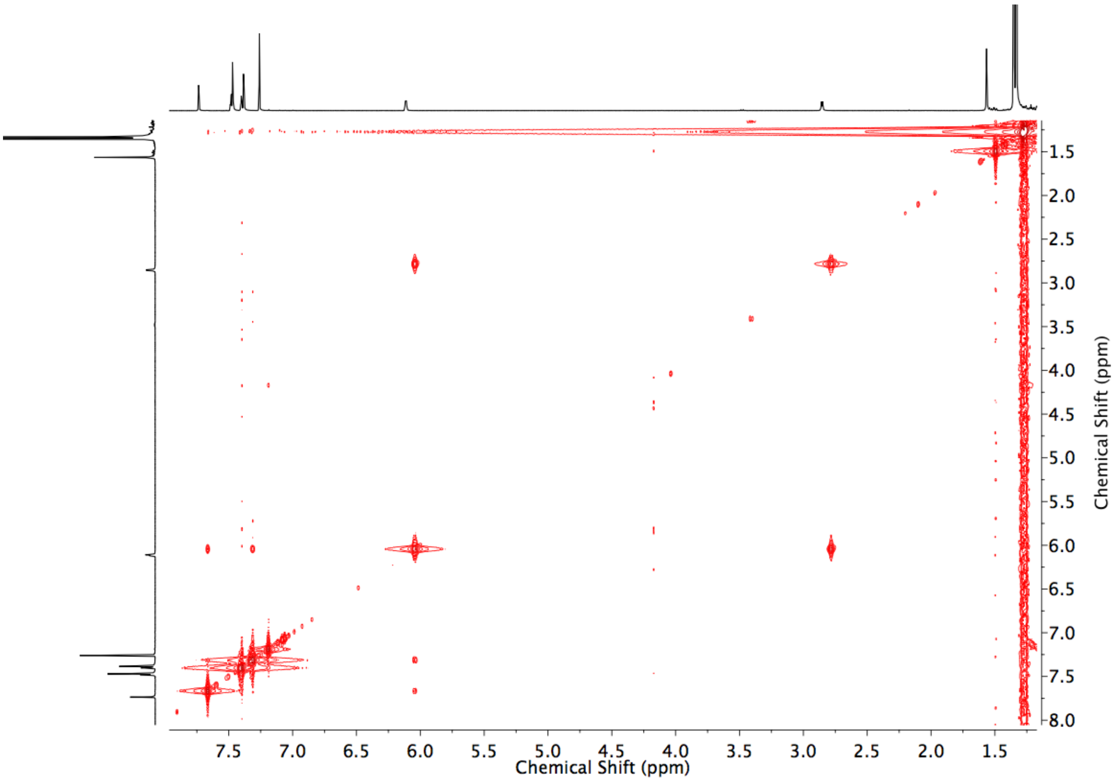

Figure S105 COSY NMR ($CDCl_3$) of **S12**

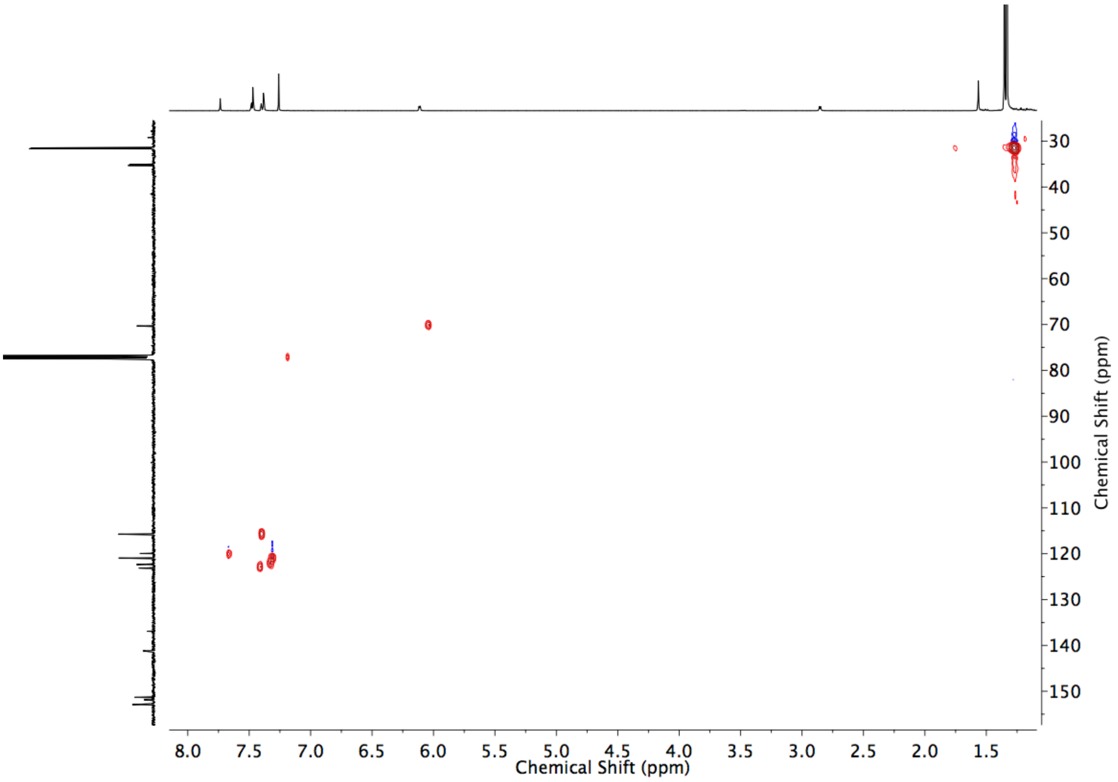

Figure S106 HSQC NMR ($CDCl_3$) of **S12**

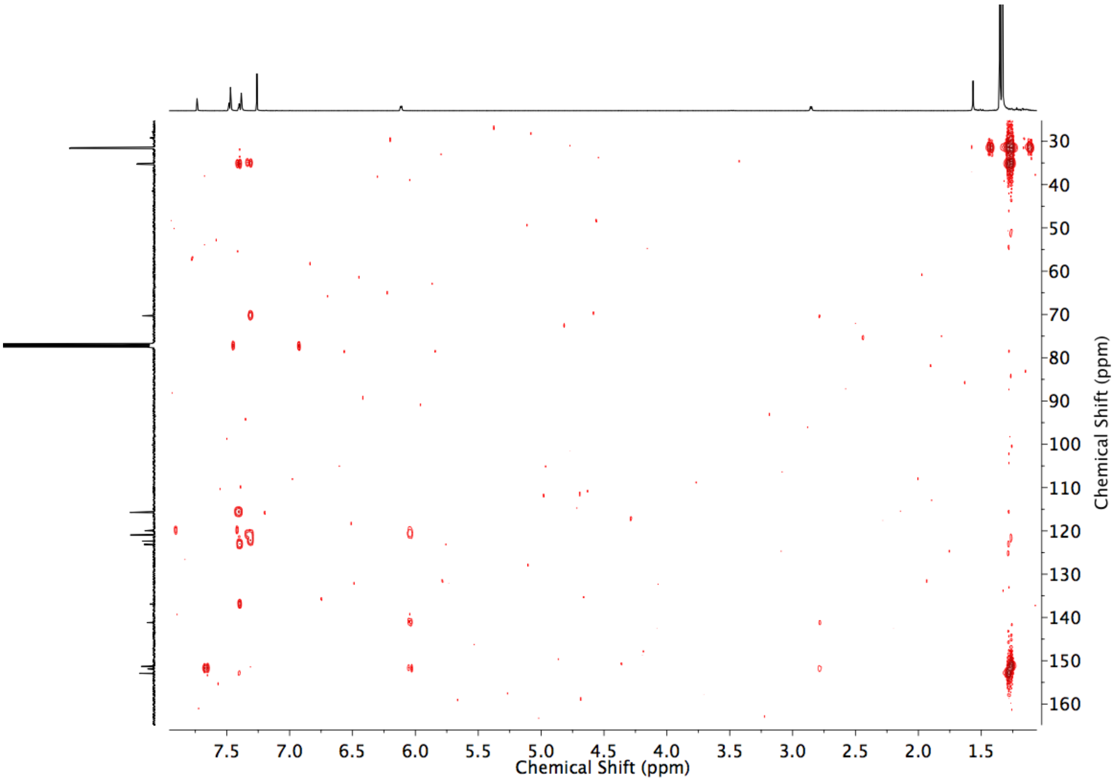

Figure S107 HMBC NMR ($CDCl_3$) of **S12**

Thread **S13**

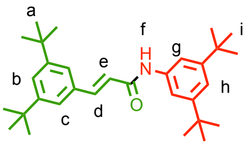

Rotaxane **6** (30.0 mg, 0.03 mmol) was dissolved in CH_2Cl_2 (1.0 mL). CF_3CO_2H (0.5 mL) was added and the mixture was stirred for 16 h at rt. The reaction mixture was diluted with CH_2Cl_2 (10 mL), washed with saturated $NaHCO_{3(aq)}$ (5 mL), brine (5 mL), dried ($MgSO_4$), filtered and the solvent removed *in vacuo*. Chromatography (petrol and 0 to 10% Et_2O) gave **S13** as a white foam (11.0 mg, 82%). ^1H NMR (400 MHz, $CDCl_3$): δ 7.79 (d, *J* = 15.5, 1H, H_d), 7.51 (br. s, 2H, H_g), 7.46 (t, *J* =1.7, 1H, H_b) 7.39 (d, *J* =1.7, 2H, H_c), 7.34 (br. s, 1H, H_f), 7.21 (br. s, 1H, H_h), 6.57 (d, *J* = 15.5, 1H, H_e), 1.35 (s, 18H, H_i), 1.34 (s, 18H, H_a). ^{13}C NMR (126 MHz, $CDCl_3$) δ 164.1, 151.7, 151.4, 143.3, 137.5, 134.0, 124.4, 122.3, 120.3, 118.6, 114.5, 35.0, 34.9, 31.4, 31.4. HR-ESI-MS *m/z* = 448.3570 $[M+H]^+$ (calc. for $C_{31}H_{46}NO$ 448.3574).

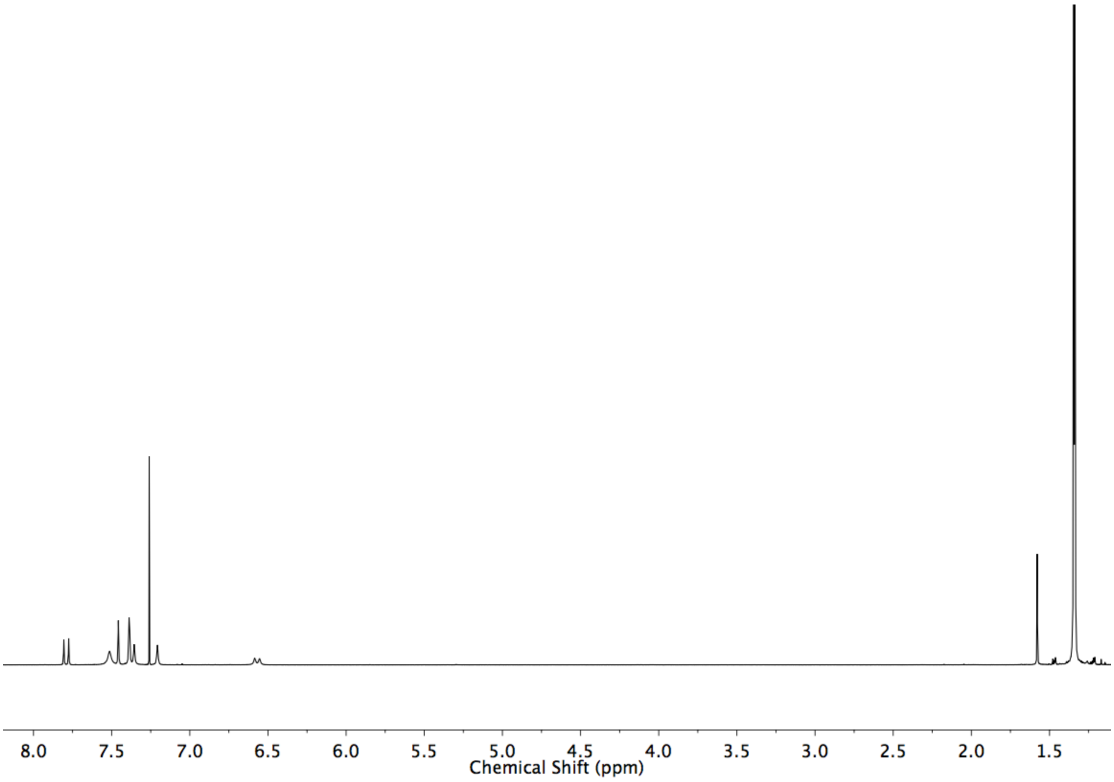

Figure S108 ^1H NMR ($CDCl_3$, 500 MHz) of **S13**

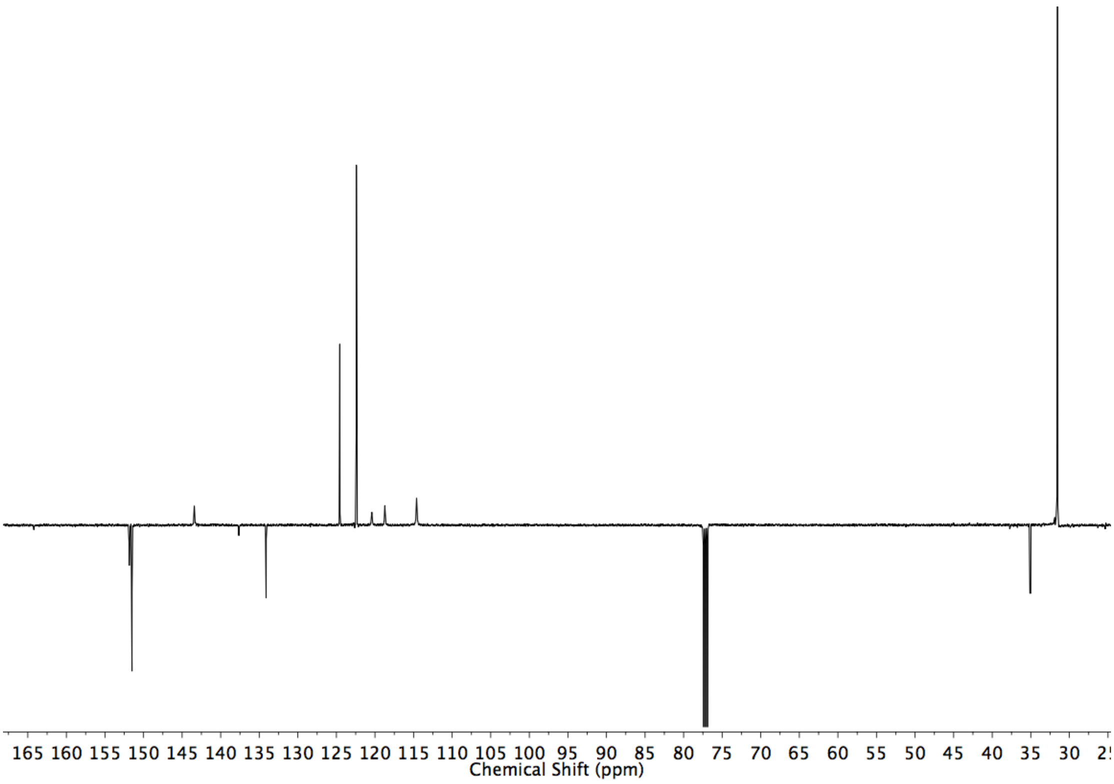

Figure S109 JMOD NMR ($CDCl_3$, 126 MHz) of **S13**

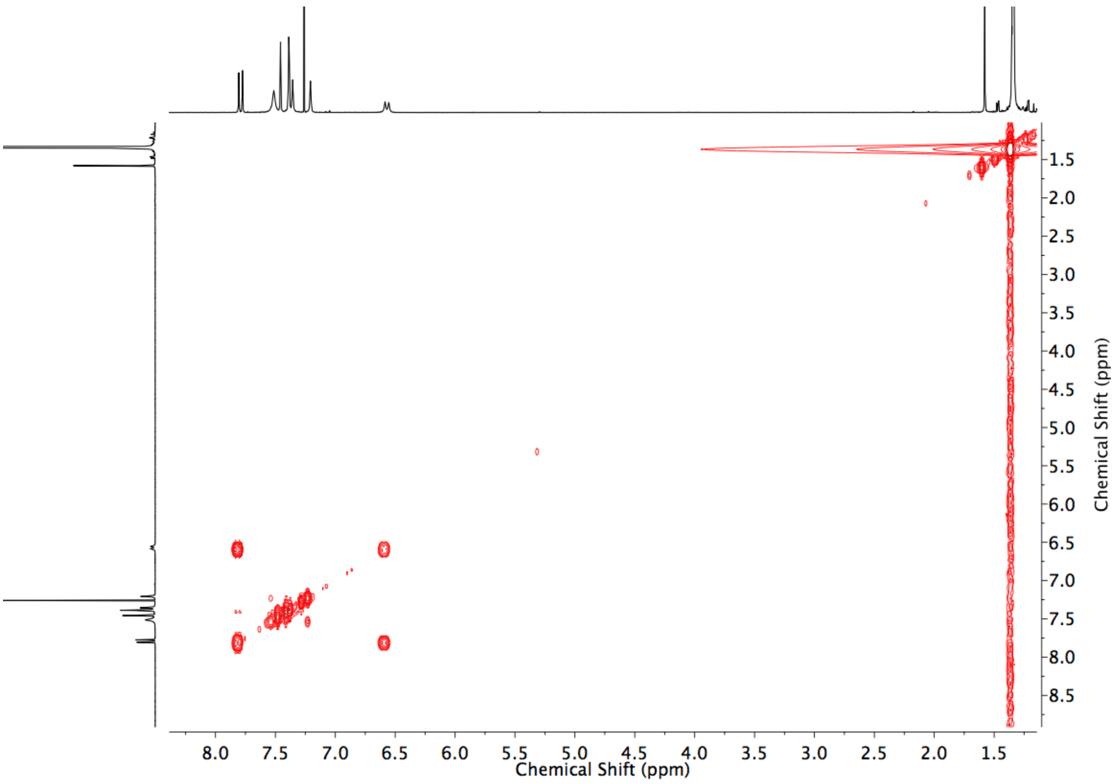

Figure S110 COSY NMR ($CDCl_3$) of **S13**

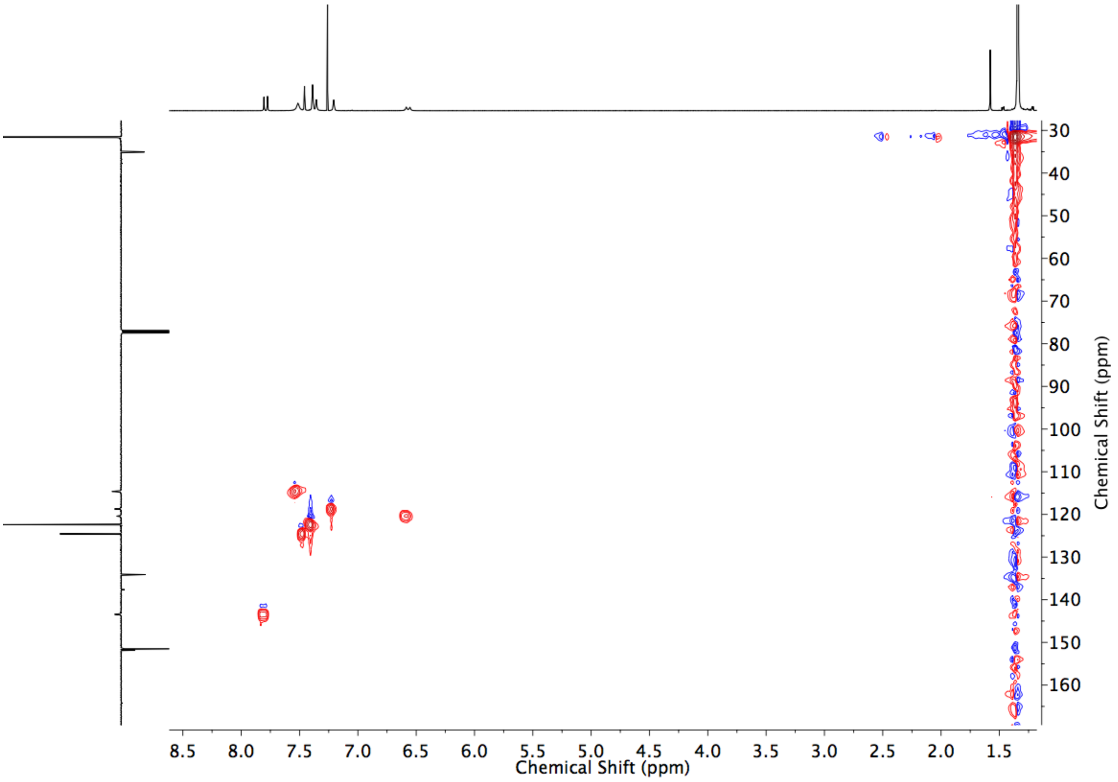

Figure S111 HSQC NMR ($CDCl_3$) of **S13**

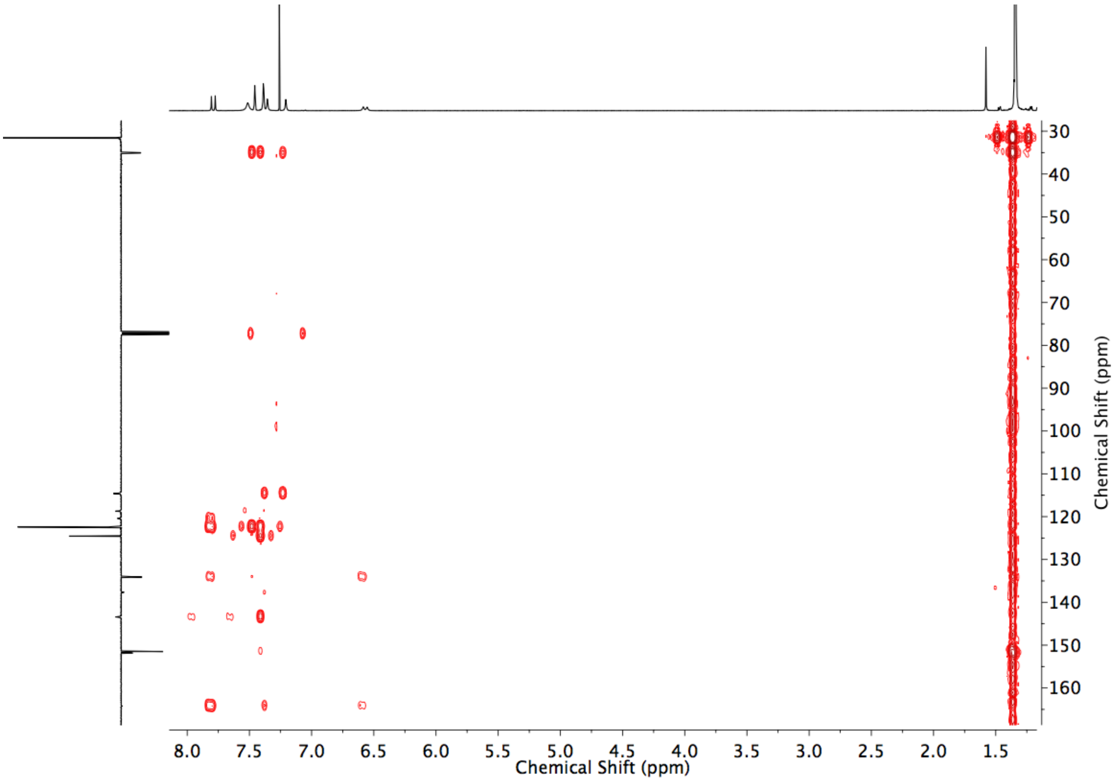

Figure S112 HMBC NMR ($CDCl_3$) of **S13**

5. Single crystal X-ray analysis of rotaxanes 4, 5, 6 and 8

Crystals of **4** were grown by vapour diffusion of Et_2O into a CH_2Cl_2 solution. Crystals of **5**, **6** and **8** were grown by vapour diffusion of pentane into a Et_2O solution.

Data were collected at 100 K using a FRE+ HF diffractometer equipped with a Saturn 724+ enhanced sensitivity detector. Cell determination, data collection, data reduction, cell refinement and absorption correction were performed with CrysalisPro. The structures **4** and **8** were solved using SUPERFLIP,[8,9] **5** was solved using ShelXT,[10] and **6** was solved using ShelXS.[11] All structures were refined against F_2 using anisotropic thermal displacement parameters for all non-hydrogen atoms using ShelXL[10] and software packages within. Hydrogen atoms were placed in calculated positions, except structure **5** H(1) which was located in the difference map, and all were refined using a riding model.

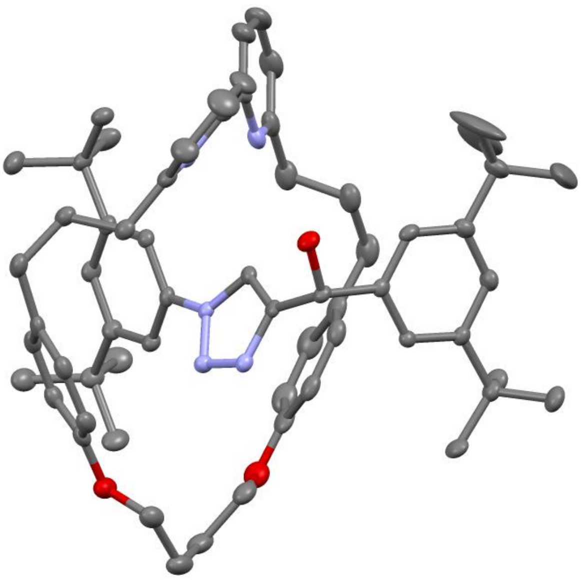

Figure S113 Ellipsoid plot of the asymmetric unit of **4** (ellipsoids shown at 50% probability). Hydrogen atoms omitted for clarity.

Compound	**4**
CCDC	1890755
Empirical formula	$C_{63}H_{79}N_5O_3$
Formula weight	954.31
Temperature/K	293(2)
Crystal system	monoclinic
Space group	$P2_1/n$
a/Å	13.8964(7)
b/Å	21.8200(10)
c/Å	18.2565(9)
α/°	90
β/°	94.479(5)
γ/°	90
Volume/Å^3	5518.8(5)
Z	4
ρ_{calc}g/cm^3	1.149
μ/mm^{-1}	0.070
F(000)	2064.0
Crystal size/mm^3	0.055 × 0.05 × 0.015
Radiation	MoKα (λ = 0.71075)
2Θ range for data collection/°	4.752 to 52.744
Index ranges	-17 ≤ h ≤ 17, -27 ≤ k ≤ 27, -22 ≤ l ≤ 22
Reflections collected	63066
Independent reflections	11291 [R_{int} = 0.0677, R_{sigma} = 0.0463]
Data/restraints/parameters	11291/0/656
Goodness-of-fit on F^2	1.004
Final R indexes [I>=2σ (I)]	R_1 = 0.0543, wR_2 = 0.1189
Final R indexes [all data]	R_1 = 0.0731, wR_2 = 0.1284
Largest diff. peak/hole / e Å^{-3}	0.54/-0.25

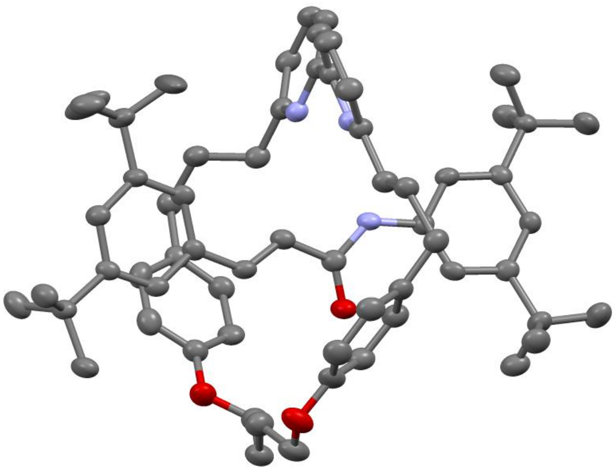

Figure S114 Ellipsoid plot of the asymmetric unit of **5** (ellipsoids shown at 50% probability). Hydrogen atoms omitted for clarity.

Compound	**5**
CCDC	**1890756**
Empirical formula	$C_{63}H_{79}N_3O_3$
Formula weight	926.29
Temperature/K	100(2)
Crystal system	orthorhombic
Space group	$Pca2_1$
a/Å	17.0247(4)
b/Å	26.2343(10)
c/Å	12.4688(4)
α/°	90
β/°	90
γ/°	90
Volume/Å^3	5569.0(3)
Z	4
ρ_{calc}g/cm^3	1.105
μ/mm^{-1}	0.067
F(000)	2008.0
Crystal size/mm^3	0.2 × 0.07 × 0.035
Radiation	MoKα (λ = 0.71075)
2Θ range for data collection/°	3.92 to 52.746
Index ranges	-11 ≤ h ≤ 21, -18 ≤ k ≤ 32, -15 ≤ l ≤ 15
Reflections collected	21233
Independent reflections	11078 [R_{int} = 0.0474, R_{sigma} = 0.0902]
Data/restraints/parameters	11078/1/637
Goodness-of-fit on F^2	1.026
Final R indexes [I>=2σ (I)]	R_1 = 0.0738, wR_2 = 0.1496
Final R indexes [all data]	R_1 = 0.1213, wR_2 = 0.1810
Largest diff. peak/hole / e Å^{-3}	0.31/-0.24

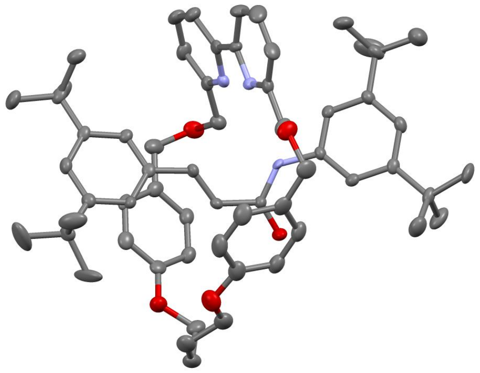

Figure S115 Ellipsoid plot of the asymmetric unit of **6** (ellipsoids shown at 50% probability). Hydrogen atoms omitted for clarity.

A "B–level" alert was detected using the IUCR checkcif algorithm. This was determined to be due to the geometry of the acrylamide combined with the macrocycle orientation which forces the amide proton and one of the alkene protons into close proximity. Thus, this alert is not a crystallographic error but an unusual feature of the interlocked structure which sterically constrains the covalent subcomponents in an otherwise energetically disfavoured arrangement.

Compound	**6**
CCDC	**1890757**
Empirical formula	$C_{61}H_{75}N_3O_5$
Formula weight	930.24
Temperature/K	100(2)
Crystal system	monoclinic
Space group	$P2_1$
a/Å	10.8046(5)
b/Å	16.8432(5)
c/Å	15.2219(6)
α/°	90
β/°	105.545(4)
γ/°	90
Volume/Å^3	2668.80(19)
Z	2
ρ_{calc}g/cm^3	1.158
μ/mm^{-1}	0.073
F(000)	1004.0
Crystal size/mm^3	0.1 × 0.05 × 0.02
Radiation	MoKα (λ = 0.71075)
2Θ range for data collection/°	5.876 to 52.744
Index ranges	$-13 \le h \le 13$, $-20 \le k \le 21$, $-19 \le l \le 19$
Reflections collected	25741
Independent reflections	10822 [R_{int} = 0.0558, R_{sigma} = 0.0948]
Data/restraints/parameters	10822/1/634
Goodness-of-fit on F^2	1.042
Final R indexes [I>=2σ (I)]	R_1 = 0.0718, wR_2 = 0.1599
Final R indexes [all data]	R_1 = 0.1045, wR_2 = 0.1737
Largest diff. peak/hole / e Å^{-3}	0.47/-0.21

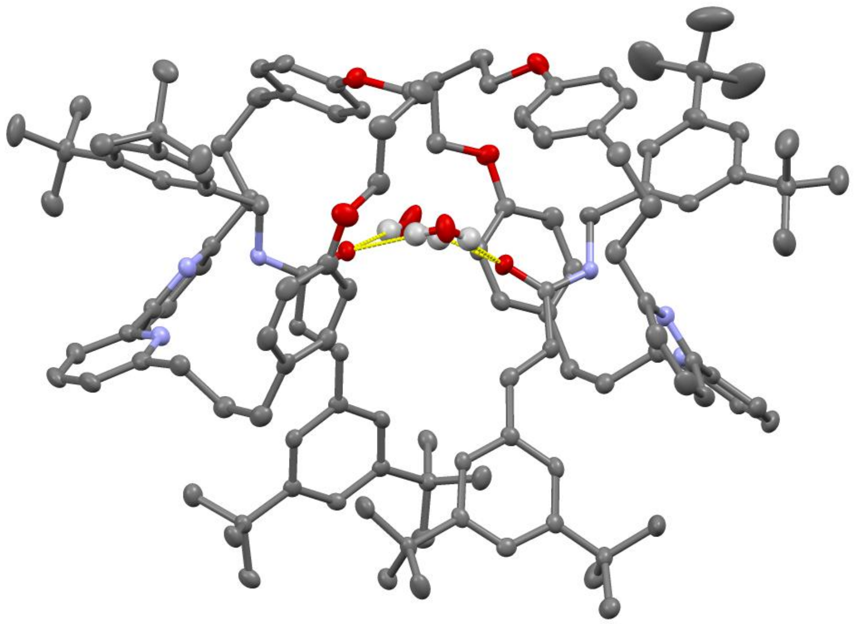

Figure S116 Ellipsoid plot of the asymmetric unit of **8** (ellipsoids shown at 50% probability). Hydrogen atoms omitted for clarity, except for H11C, H11D, H15L and H15M.

Compound	**8**
CCDC	**1890758**
Empirical formula	$C_{64}H_{83}N_3O_4$
Formula weight	958.33
Temperature/K	100(2)
Crystal system	triclinic
Space group	P-1
a/Å	15.5175(6)
b/Å	16.9490(5)
c/Å	23.3325(6)
α/°	105.257(2)
β/°	100.079(3)
γ/°	93.338(3)
Volume/Å^3	5793.7(3)
Z	4
ρ_{calc}g/cm^3	1.099
μ/mm^{-1}	0.067
F(000)	2080.0
Crystal size/mm^3	0.1 × 0.05 × 0.01
Radiation	MoKα (λ = 0.71075)
2Θ range for data collection/°	5.804 to 52.744
Index ranges	-19 ≤ h ≤ 17, -20 ≤ k ≤ 21, -29 ≤ l ≤ 25
Reflections collected	51024
Independent reflections	22793 [R_{int} = 0.0432, R_{sigma} = 0.0799]
Data/restraints/parameters	22793/0/1309
Goodness-of-fit on F^2	1.155
Final R indexes [I>=2σ (I)]	R_1 = 0.0877, wR_2 = 0.1845
Final R indexes [all data]	R_1 = 0.1236, wR_2 = 0.1990
Largest diff. peak/hole / e Å^{-3}	0.89/-0.50

6. Optimisation of reaction conditions for the rearrangement reaction

Table S1 Optimisation of the formation of **5** with respect to solvent, temp. and additive.

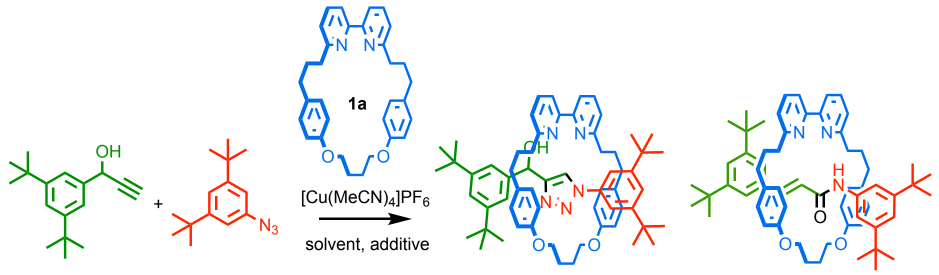

Entry	Time	Solvent	Temp	Additive	Conversion^a	Ratio 4 : 5^a
**1**	**16 h**	**CH_2Cl_2**	**RT**	**N^iPr_2Et (2 eq.)**	**100%**	**>99 : <1**
2	16 h	CH_2Cl_2 + 10% H_2O	RT	N^iPr_2Et (2 eq.)	100%	90 : 10
3	16 h	CH_2Cl_2 + 10% H_2O	RT	-	trace - 100%*	60 : 40
4	24 h	CH_2Cl_2	RT	-	0	-
5	24 h	THF	RT	-	0	-
6	48 h	THF + 10% H_2O	RT	-	24 - 100%*	<1 : >99
7	48 h	THF + 10% H_2O	RT	KF (0.9 eq.)	100%	<1 : >99
8	48 h	THF + 10% H_2O	RT	TBAF (0.9 eq.)	100%$^{\pm}$	50 : 50
9^b	20 min	THF + 10% H_2O	80 °C	-	66 - 80%*	<1 : >99
10^b	20 min	THF + 10% H_2O	80 °C	KF (0.9 eq)	>99%	4 : 96
11^b	20 min	THF + 10% H_2O	80 °C	TBAF (0.9 eq)	100%	57:43
**12^b**	**1 h**	**THF + 10% H_2O**	**70 °C**	**KF (0.9 eq)**	**100%**	**<1 : >99**
**13^c**	**1 h**	**THF + 10% H_2O**	**70 °C**	**KF (0.9 eq)**	**100%**	**<1 : >99**
14^b	1 h	THF + 10% H_2O	70 °C	KNO_3 (0.9 eq)	100%	7: 93
15^b	1 h	THF + 10% H_2O	70 °C	TBAF (0.9 eq)	100%	8 : 92
16^{b,d}	1 h	THF + 10% H_2O	70 °C	KF (0.9 eq)	100%	72 : 28
17^{b,d}	1 h	THF + 10% H_2O	70 °C	KNO_3 (0.9 eq)	100%	>99 : <1

^{a}Determined by ^1H NMR, ^{b}Performed in a microwave reactor. ^{c}Performed under thermal conditions in an oil bath. ^{d}[Cu(MeCN)$_4$]PF_6 was replaced by $CuSO_4$/NaAsc. *Conversion varied run-to-run. $^{\pm}$Decomposition to an unknown product was also observed.

The role of KF remains unclear; neither TBAF (entries 8, 11 and 15) nor KNO_3 (entries 14 and 17) produce the same outcome as KF under the same conditions, suggesting that both the cation and anion play a role. Interestingly at 70 °C, TBAF and KF appear more comparable (entry 12 vs 15). (note: at 80 °C [Entry 11] the lower selectivity of TBAF may be due to fluoride-mediated decomposition of the tetrabutyl ammonium cation)[12] Interestingly, when [Cu(MeCN)$_4$]PF_6 is replaced by $CuSO_4$/Na-ascorbate, KNO_3 outperforms KF, suggesting that the role of the inorganic salt is quite complex.

7. Kinetic Study of the reaction of 1a, 2a and 3a.

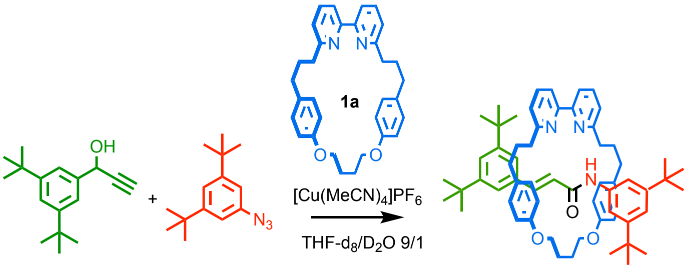

1a (7.3 mg, 0.0153 mmol), $[Cu(MeCN)_4]PF_6$ (5.5mg, 0.0147 mmol), **2a** (4.3mg, 0.0184 mmol), and **3a** (4.5 mg, 0.0184 mmol) were dissolved in D_2O-THF-d_8 (1 : 9, 1.2 mL). The solution was passed through a Celite plug and 0.6 ml was transferred into a NMR tube and analysed by ^1H NMR at 2 h intervals. The disappearance of the peak at 7.16 ppm (d, J = 1.7, 2H) and the appearance of the peak at 7.91 (d, J = 1.7 Hz, 1H) was monitored.

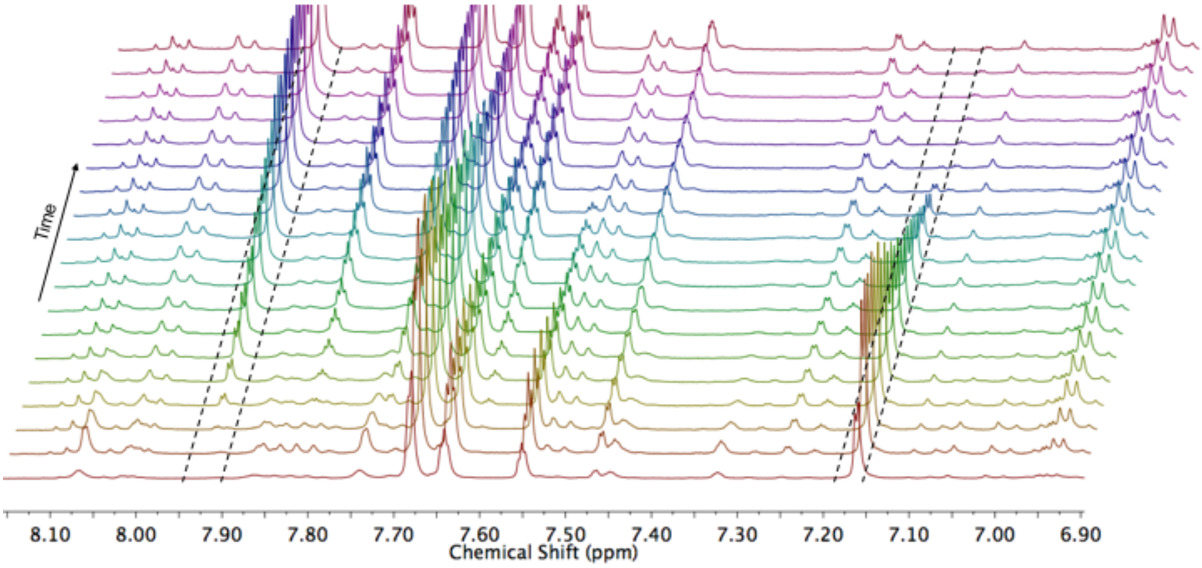

Figure S117 Stacked partial ^1H NMRs (400 MHz, THF-d_8/D_2O 9:1) showing formation of **5** and consumption of **2a.**

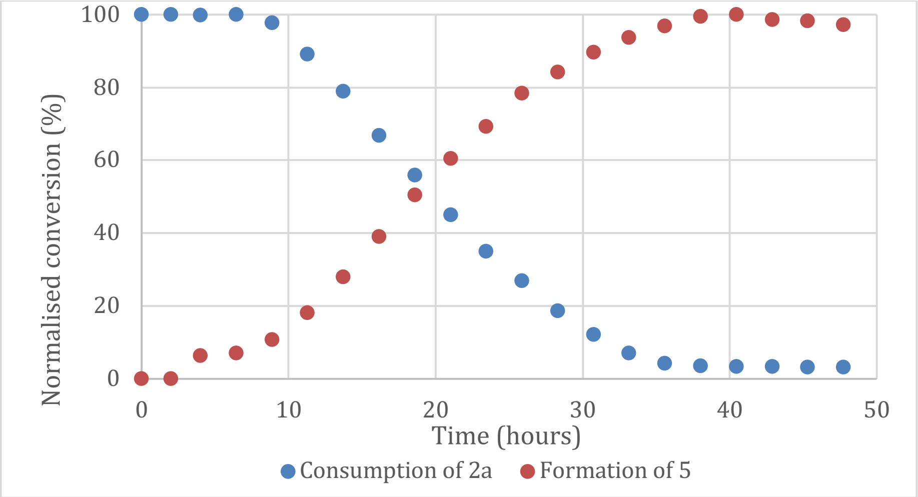

Figure S118 Consumption of **2a** and formation of 5 based on their normalised integrals with respect to the residual protonated THF signal (3.62 ppm)

8. Control experiments: axle formation under conditions optimised for the formation of acrylamide rotaxane 5

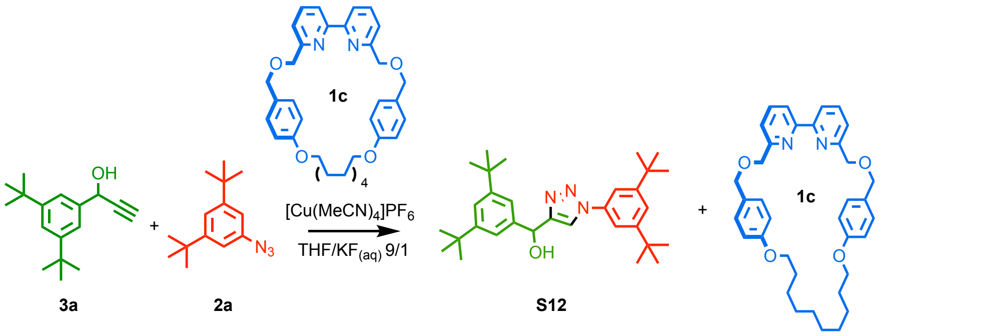

$KF_{(aq)}$ (0.2 mL, 0.1 M), was added to a solution of **3a** (7.3 mg, 0.030 mmol), **2a** (6.9 mg, 0.030 mmol) **1c** (14.3 mg, 0.025 mmol) and $[Cu(MeCN)_4]PF_6$ (8.9 mg, 0.024 mmol) in THF (1.8 mL) in a microwave vial. The orange mixture was stirred at 70 °C (μW) for 1 h. The reaction mixture was diluted with CH_2Cl_2 (10 mL) and washed with EDTA-NH_3 solution (10 mL). The aqueous layer extracted with CH_2Cl_2 (2 × 50 mL). The combined organic extracts were washed with brine (50 mL), dried ($MgSO_4$), filtered and the solvent removed *in vacuo*. Analysis by ^{1}H NMR revealed **S12** to be the sole product.

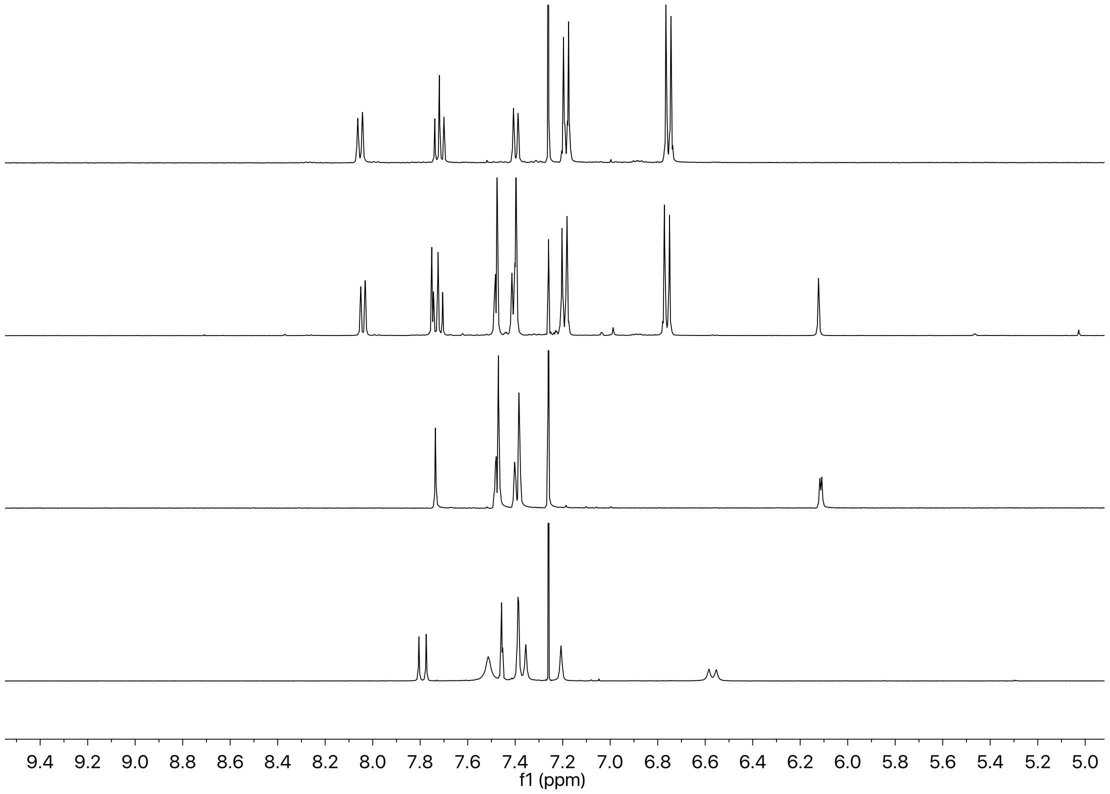

Figure S119 Stacked partial ^{1}H NMR (400 MHz, $CDCl_3$) spectra of (from top to bottom) **1c**, the crude reaction mixture, triazole axle **S12** and acrylamide **S13**.

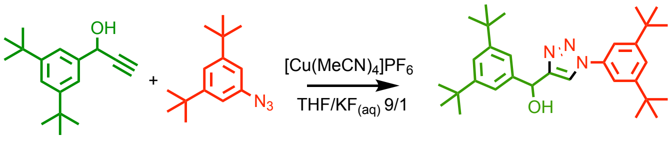

$KF_{(aq)}$ (0.2 mL, 0.1M), was added to a solution of **3a** (7.3 mg, 0.030 mmol), **2a** (6.9 mg, 0.030 mmol) and $[Cu(MeCN)_4]PF_6$ (8.9 mg, 0.024 mmol) in THF (1.8 mL) in a microwave vial. The yellow mixture was stirred at 70 °C (μW) for 1 h. The crude reaction mixture was diluted with CH_2Cl_2 (10 mL) and washed with EDTA-NH_3 solution (10 mL). The aqueous layer was extracted with CH_2Cl_2 (2 × 50 mL). The combined organic extracts were washed with brine (50 mL), dried ($MgSO_4$), filtered and the solvent removed *in vacuo*. Analysis by ^1H NMR revealed **S12** to be the sole product.

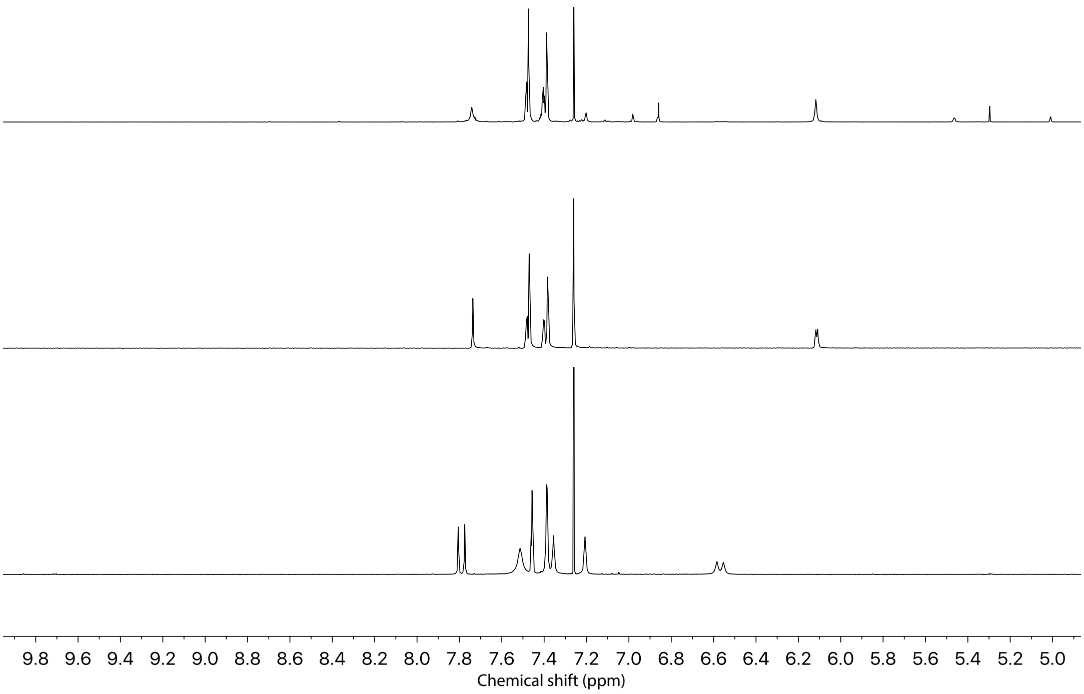

Figure S120 Stacked partial ^1H NMR (400 MHz, $CDCl_3$) of (from top to bottom) the crude reaction mixture, triazole axle **S12** and acrylamide **S13.**

9. Mechanistic studies: rearrangement of triazolide 12 under aqueous conditions

To investigate whether triazolide **12** is an intermediate *en route* to acrylamide rotaxane **11** we explored the reaction of **12** under various conditions and analysed the results by ^{1}H NMR:

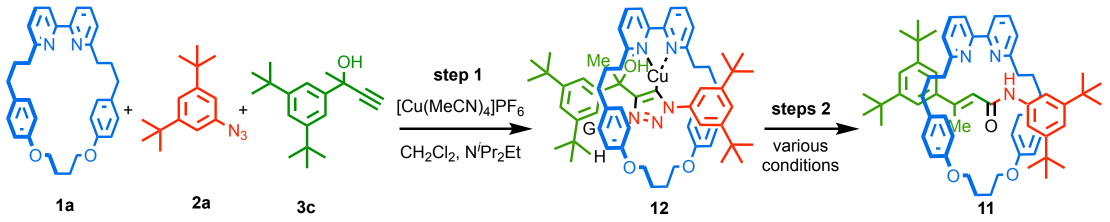

Step 1: $^{i}Pr_2NEt$ (40.0 µl, 0.23 mmol) was added to a solution of **3c** (25.6 mg, 0.10 mmol), **2a** (23.1 mg, 0.10 mmol), **1a** (48.0 mg, 0.10 mmol) and $[Cu(MeCN)_4]PF_6$ (36.6 mg, 0.098 mmol) in CH_2Cl_2 (8.0 mL). The deep-red solution was washed with H_2O (10 mL), brine (10 mL), dried ($MgSO_4$) and the solvent removed *in vacuo*. ^{1}H NMR analysis of the residue confirmed that the starting materials had been consumed to produce a new species whose ^{1}H NMR resonances are consistent with triazolide **12**; four signals are observed for macrocycle protons H_G and H_H (~ 6.2 ppm) due to the presence of the axle stereogenic centre, no triazole resonance was observed, and the spectrum is distinct from that of the $[Cu(\mathbf{11})]^+$. MS analysis of a portion of the solution supports this assignment (*m/z* =1030.9 $[M+H]^+$; calc. for $C_{64}H_{80}CuN_5O_3$ = 1030.9).

Step 2A: The residue from **step 1** was dissolved in a mixture of $KF_{(aq)}$ (0.1 M, 0.1 mL) and THF (0.9 mL). The orange mixture was stirred at 70 °C (µW) for 1 h. The reaction mixture was diluted with CH_2Cl_2 (20 mL) and washed with EDTA-NH_3 solution (10 mL). The aqueous layer was extracted with CH_2Cl_2 (2 × 10 mL). The combined organic extracts were washed with brine (10 mL), dried ($MgSO_4$) and the residue analysed by ^{1}H NMR.

Step 2B: The residue from **step 1** was dissolved in a mixture of $KF_{(aq)}$ (0.2 M, 0.05 mL), $HPF_{6(aq)}$ (0.2 M, 0.05 mL) and THF (0.9 mL). The orange mixture was stirred at 70 °C (µW) for 1 h. The reaction mixture was diluted with CH_2Cl_2 (20 mL) and washed with EDTA-NH_3 solution (10 mL). The aqueous layer was extracted with CH_2Cl_2 (2 × 10 mL). The combined organic extracts were washed with brine (10 mL), dried ($MgSO_4$) and the residue analysed by ^{1}H NMR.

Step 2C: The residue from **step 1** was dissolved in a mixture of $HPF_{6(aq)}$ (0.13 M, 0.1 mL, 1 eq,) and THF (0.9 mL). The orange mixture was stirred at 70 °C (µW) for 1 h. The reaction mixture was diluted with CH_2Cl_2 (20 mL) and washed with EDTA-NH_3 solution (10 mL). The aqueous layer was extracted with CH_2Cl_2 (2 × 10 mL). The combined organic extracts were washed with brine (10 mL), dried ($MgSO_4$) and the residue analysed by ^{1}H NMR.

Step 2D: The residue from **step 1** was dissolved in a mixture of $HPF_{6(aq)}$ (0.13 M, 0.1 mL, 1 eq,) and THF (0.9 mL). The orange mixture was stirred for 1 h at rt. The reaction mixture was diluted with CH_2Cl_2 (20 mL) and washed with EDTA-NH_3 solution (10 mL). The aqueous layer was extracted with CH_2Cl_2 (2 × 10 mL). The combined organic extracts were washed with brine (10 mL), dried ($MgSO_4$) and the solvent removed *in vacuo*. The residue was analysed by ^{1}H NMR.

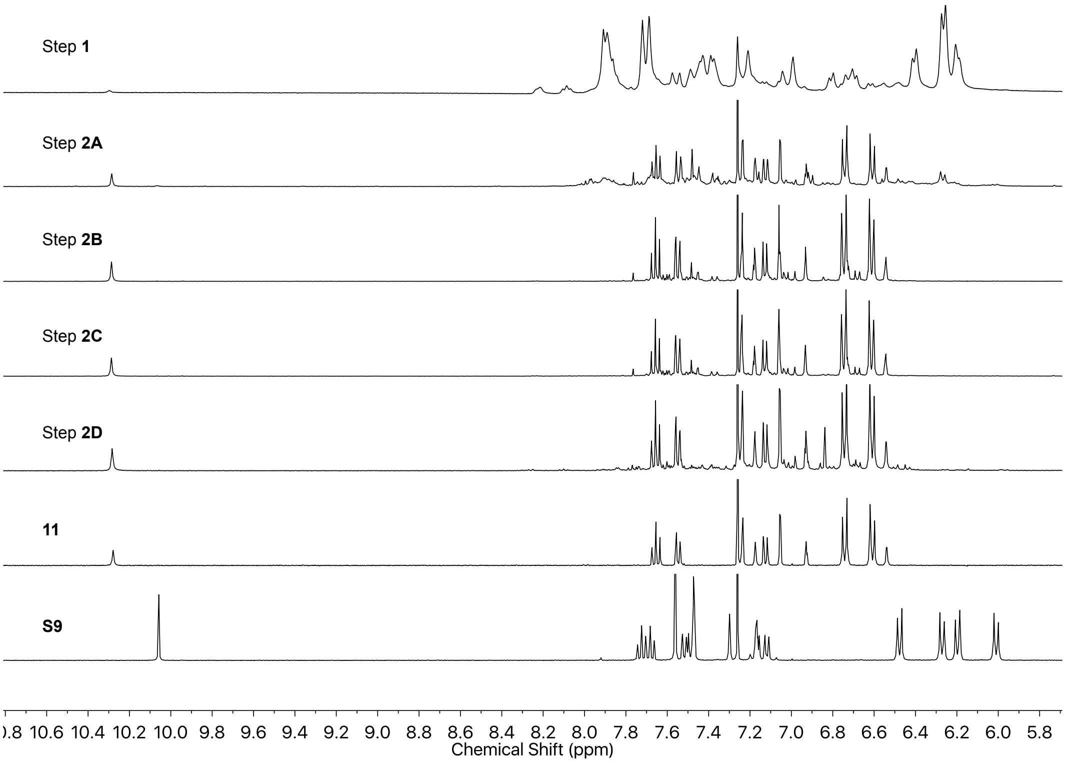

Figure S121 Stacked partial ^{1}H NMR (400 MHz, $CDCl_3$) of the products of **step 1** and **steps 2** alongside **11** and **S9** for comparison.

Production of **11** was confirmed by ^{1}H NMR. In all cases, triazole rotaxane **S9** was not observed. Due to broadening of the signals corresponding to triazolide **12**, conversion could not be quantified in the case of **step 2A**. However, the presence of the doublet at 6.21 ppm which is assigned to **12**, indicates that consumption of **12** is incomplete.

Control experiments

To rule out the conversion of **12** to rotaxane **S9** followed by reaction to produce **11**, control experiments with rotaxane **S9** were performed under the same conditions:

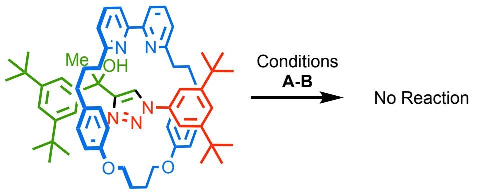

S9

Conditions A: Triazole rotaxane **S9** (1.6 mg, 0.0017 mmol) in THF (200 µL) was treated with a solution of $HPF_{6(aq)}$ (20 µL, 0.08 M, 0.0017 mmol) in a sealed microwave vial. The mixture was stirred at 70 °C (µW) for 1 h. The reaction was diluted with CH_2Cl_2 (10 mL) and washed with EDTA-$NH_{3(aq)}$ (5 mL). The aqueous layer was extracted with CH_2Cl_2 (2 × 10 mL). The combined organic extracts were washed with brine (10 mL), dried ($MgSO_4$), filtered and the solvent removed *in vacuo*. Analysis of the residue by ^{1}H NMR showed some decomposition but no formation of **11**.

Conditions B: Triazole rotaxane **S9** (1.6 mg, 0.0017 mmol) and $[Cu(MeCN)_4]PF_6$ (0.6 mg, 0.0016 mmol) were dissolved in THF (200 µL) and treated with a solution of $HPF_{6(aq)}$ (20

μL, 0.08 M, 0.0017 mmol), in a sealed microwave vial. The mixture was stirred at 70 °C (μW) for 1 h. The reaction mixture was diluted with CH_2Cl_2 (10 mL) and washed with EDTA-$NH_{3(aq)}$ (5 mL). The aqueous layer was extracted with CH_2Cl_2 (2 × 10 mL). The combined organic extracts were washed with brine (10 mL), dried ($MgSO_4$), filtered and the solvent removed *in vacuo*. Analysis of the residue by ^1H NMR showed a small amount of decomposition but no formation of **11**.

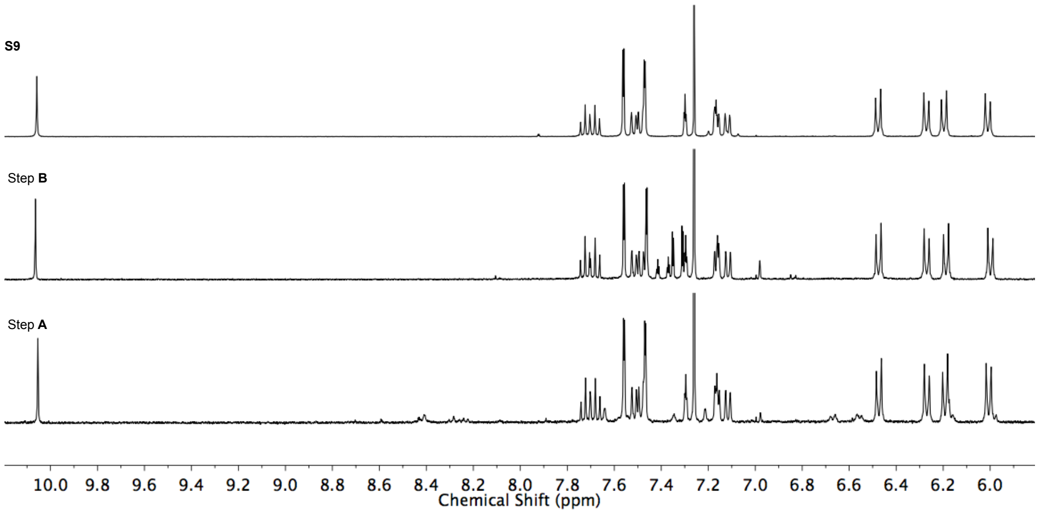

Figure S122 Stacked partial ^1H NMR (400 MHz, $CDCl_3$) of the product of **conditions A** and **B**, compared with triazole rotaxane **S9**.

10. Mechanistic studies: rearrangements triggered by Tf_2O under anhydrous conditions

i. *In situ* ^{1}H NMR analysis of the reaction of the product of **1a**, **2a** and **3c** with Tf_2O

To demonstrate the key role of the OH of triazolide **12** as a leaving group and to attempt to observe the proposed cumulene intermediate we explored the reaction of **12** with Tf_2O under anhydrous conditions and analysed the results by ^{1}H NMR:

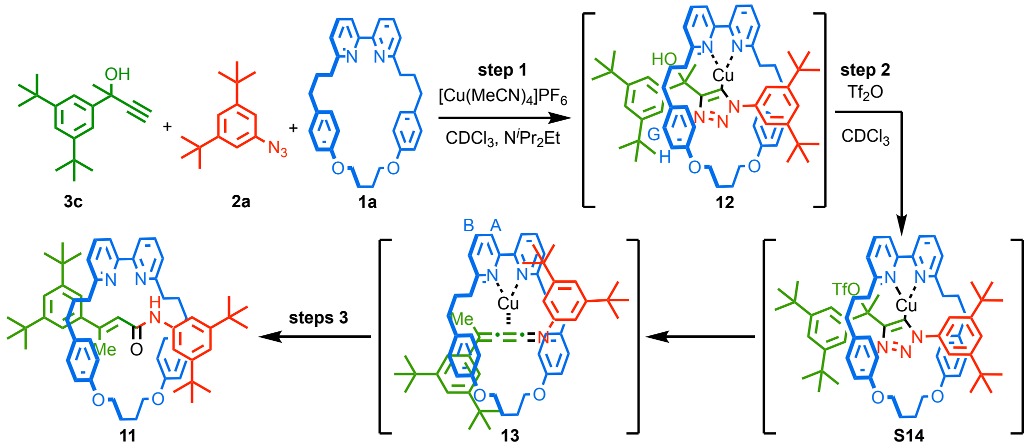

Step 1: $^iPr_2NEt$ (70 µl, 0.40 mmol) was added to a solution of **3c** (25.8 mg, 0.10 mmol), **2a** (23.1 mg, 0.10 mmol), **1a** (47.8 mg, 0.10 mmol) and $[Cu(MeCN)_4]PF_6$ (36.8 mg, 0.099 mmol) in $CDCl_3$ (4.0 mL) in a sealed microwave vial, and the mixture stirred at rt for 45 min. ^{1}H NMR analysis of the reaction mixture confirmed that the starting materials had been consumed to produce a new species whose ^{1}H NMR resonances are consistent with triazolide **12**; four signals are observed for macrocycle protons H_G and H_H (~ 6.2 ppm) due to the presence of the axle stereogenic centre, no triazole resonance was observed, and the spectrum is distinct from that of the [Cu(**11**)]$^+$. MS analysis of a portion of the solution supports this assignment (*m/z* =1030.9 [M+H]$^+$, calc. for $C_{64}H_{80}CuN_5O_3$ = 1030.9).

Step 2: A portion (2.0 mL) was removed and treated with Tf_2O (0.1 M in $CDCl_3$, 0.05 mL, 0.05 mmol) and the reaction mixture was stirred at rt for 20 mins. ^{1}H NMR analysis of the reaction mixture reveals the species assigned as **12** has largely been consumed to produce a new major species whose ^{1}H NMR resonances are consistent with cumulene **13**; macrocycle protons H_A and H_B (8.3 ppm and 8.1 ppm respectively) appear as single environments suggesting the axle stereogenic unit has been lost. This assignment is supported by LCMS analysis of a portion of the reaction mixture; the major species observed has *m/z* =984.8 (retention time = 3.02 min) which is consistent with **13** (calc. for $C_{64}H_{79}CuN_3O_2$ = 984.6). Fractions were also observed corresponding to macrocycle **1a** (2.14 min), rearranged product **11** and also **12**-OH, suggesting that **S14** persists in the reaction mixture ([M-OTf]$^+$), or that the corresponding cation is a stable intermediate in the case of **12**.

Step 3a: A portion of the solution produced in **Step 2** (0.5 mL, 0.0125 mmol) was treated with KCN (7.2 mg, 0.110 mmol) in H_2O (1 mL) and the mixture stirred at rt for 16 h. The reaction mixture was diluted with CH_2Cl_2 (20 mL), washed with H_2O (3 × 3 mL), dried ($MgSO_4$) and the solvent removed *in vacuo*. The crude residue was analysed by ^{1}H NMR to reveal **11** as the major product (**11** : **S12** ~ 95 : 5).

Step 3b: A portion of the solution produced in **Step 2** (0.5 mL, 0.0125 mmol) was treated with H_2O (1 mL) and the mixture stirred at rt for 16 h. The reaction mixture was diluted with CH_2Cl_2 (20 mL), washed with H_2O (3 × 3 mL), dried ($MgSO_4$) and the solvent removed *in vacuo*. The crude residue was analysed by ^{1}H NMR to reveal a spectrum remarkably

similar to that at the end of step 2, albeit sharper resonances were observed. This suggests that **13** survives treatment with H_2O in the absence of KCN.

Control experiment: A portion of the solution produced in **step 1** (0.5 mL, 0.0125 mmol) was treated with TfOH (1.1 μL, 0.0125 mmol), and diluted with $CDCl_3$ (25 μL). No significant change was observed by ^1H NMR (**control i**). The solution was treated with KCN (7.1 mg, 0.110 mmol) in H_2O (1 mL) and stirred for 16 h . The reaction mixture was diluted with CH_2Cl_2 (20 mL), washed with H_2O (3 × 3 mL), dried ($MgSO_4$) and the solvent removed *in vacuo*. ^1H NMR analysis (**control ii**) revealed the major product to be triazolide **12** (**11** and **S12** observed in trace amounts).

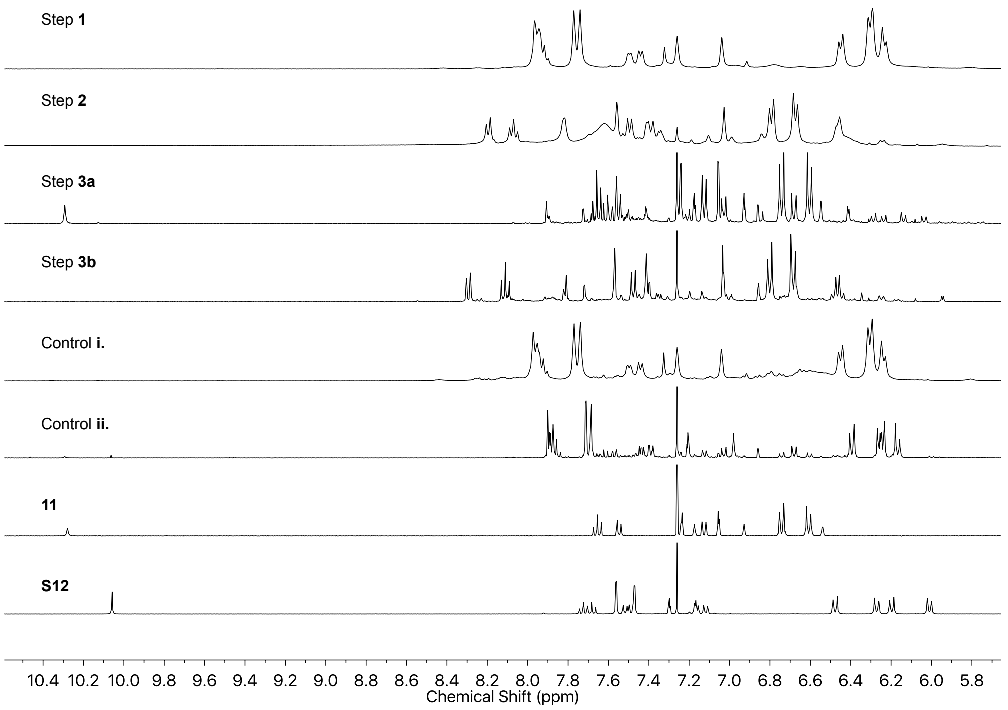

Figure S123 Stacked partial ^1H NMR (400 MHz, $CDCl_3$) of the products of **step 1**, **step 2**, **step 3a**, **step 3b**, **control i**, and **control ii**, compared with purified **11** and **S12**. At the end of **step 3a**, the ratio of **11** : **S12** = 95:5.

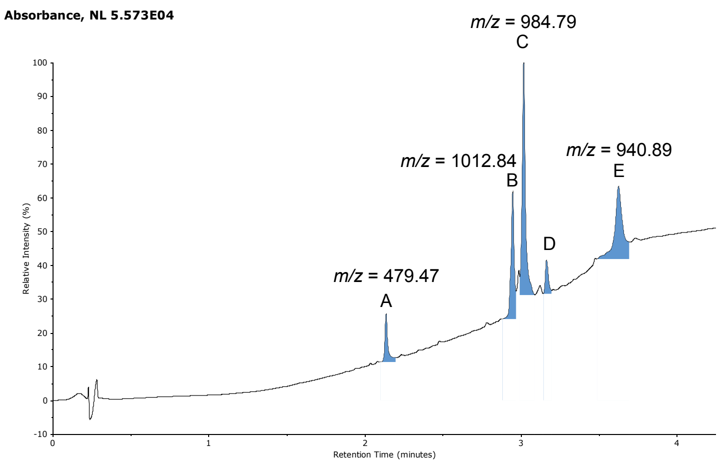

Figure S124 LCMS trace (UV = 254 nm, C18 column [1 : 4 MeCN-H_2O + 0.2% HCO_2H → MeCN + 0.2% HCO_2H), of the reaction mixture at the end of step 2. Note Peak D did not appear in the total ion count.

These experiments suggest that Tf_2O triggers the extrusion of N_2 from triazolide **12** and provide evidence that a cumulene of the form **13** is an intermediate in the reaction. The control experiment with TfOH rules out the *in situ* hydrolysis of Tf_2O to produce acid that then triggers the rearrangement. The slow reaction of **13** with H_2O in contrast with $KCN_{(aq)}$ suggests that the Cu^I ion stabilises the cumulene structure, although the lower pH of the KCN solution may also play a role in accelerating the nucleophilic attack. These results also suggest that the hydrolysis of the cumulene intermediate under the optimised reaction conditions is accelerated by H^+, given that **12** rearranges to **11** rapidly at rt in the presence of HPF_6 without added KF.

ii. *In situ* ^{1}H NMR analysis of the reaction of **1a**, **2a** and **3a** followed by Tf_2O

To provide evidence that the same mechanism observed for **12** is in operation with other substrates we investigated the reaction of **1a**, **2a** and **3a** under anhydrous conditions with Tf_2O and analysed the results by ^{1}H NMR:

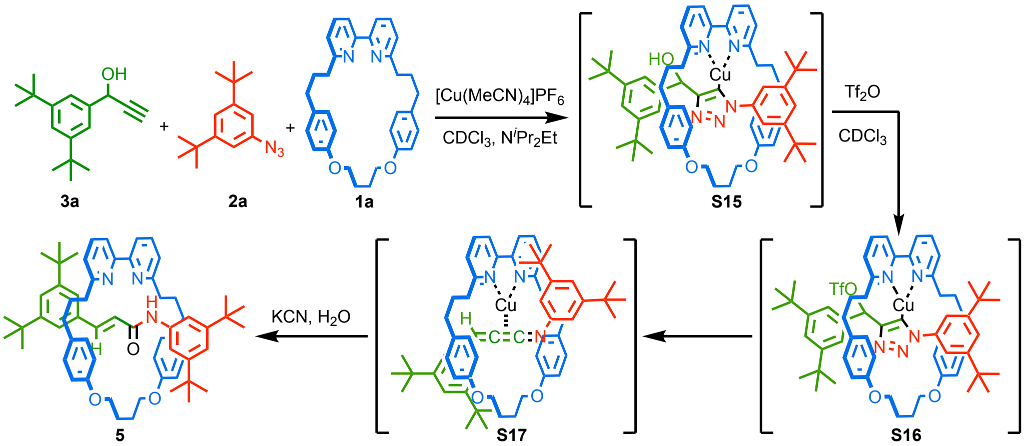

Following the same procedure as above with **1a**, **2a** and **3a** gave a similar outcome as above. After **step 1** a new species was observed that was consistent with triazolide **S15**. Treatment with Tf_2O (**step 2**) led to ~70% conversion of **S15** to produce a species assigned as cumulene **S17** (ratio of signals at ~5.0 ppm [2H of **S15**] to doublet at ~8.2 ppm [2H of **S16**] = 1 : 2.6). Treatment of this product with $KCN_{(aq)}$ (**step 3a**) gave acrylamide **5** as the major product (**5** : **4** = 90 : 10). Conversely, treatment of a solution of cumulene **S17** with H_2O (**step 3b**) resulted in incomplete consumption of **S17** to produce **5** (**5** : **4** = 90 : 10).

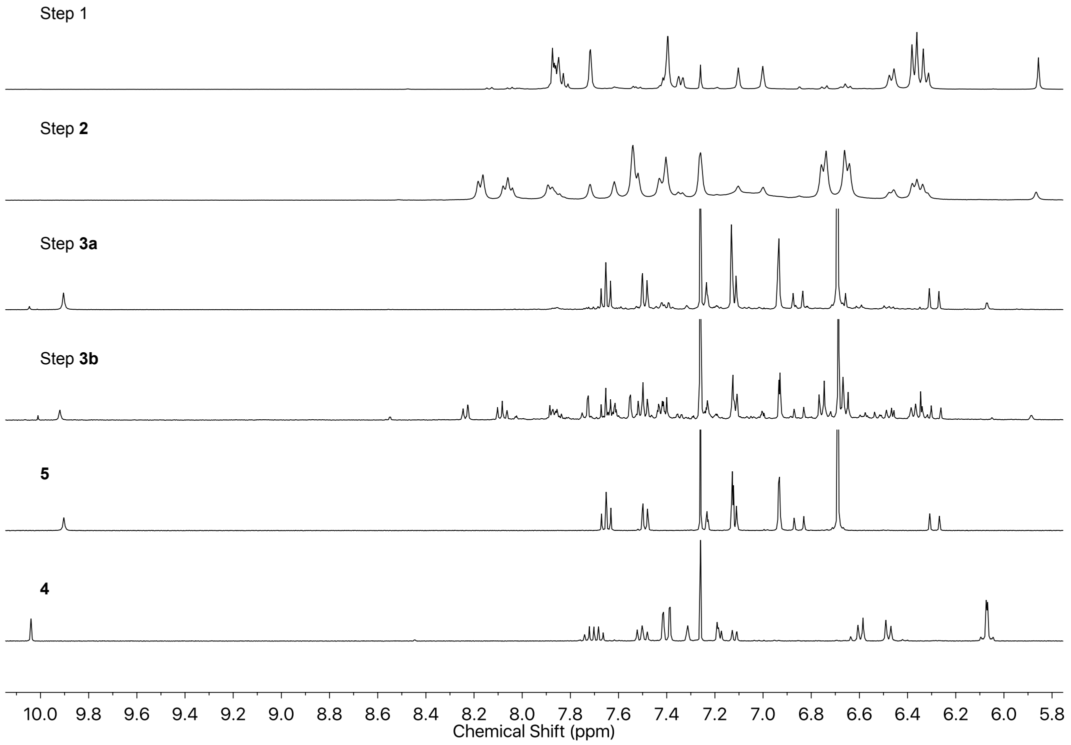

Figure S125 Stacked partial ^{1}H NMR (400 MHz, $CDCl_3$) of **step 1**, **step 2**, **step 3a** (**5** : **4** 90:10) and **step 3b** (incomplete, **5** : **4** 90:10), compared with spectra of isolated **5** and **4**.

11. Preliminary computational analysis of the mechanism of the rearrangement process

In order to provide further information on the pathway of the rearrangement process and in an attempt to identify the role of the Cu^{I} ion in the process, we carried out a preliminary computational investigation of the reaction pathway.

i. Preparation of a truncated model **Ia** of triazolide **S15**

Preliminary molecular modelling was carried out to assess the feasibility of the mechanism proposed based on the studies in sections S6-S10. Due to the large size of the interlocked intermediates of the reaction a truncated model was used.

A model of triazolide **S15** was prepared using Spartan '10 (Wavefunction Ltd.) and a conformation search performed using mechanics (MMFF, vacuum). The lowest energy structure identified was optimised using the PM6 semi-empirical method (vacuum). The model was then truncated to provide a starting point (**Ia**) for DFT calculations below (**Figure S126**).

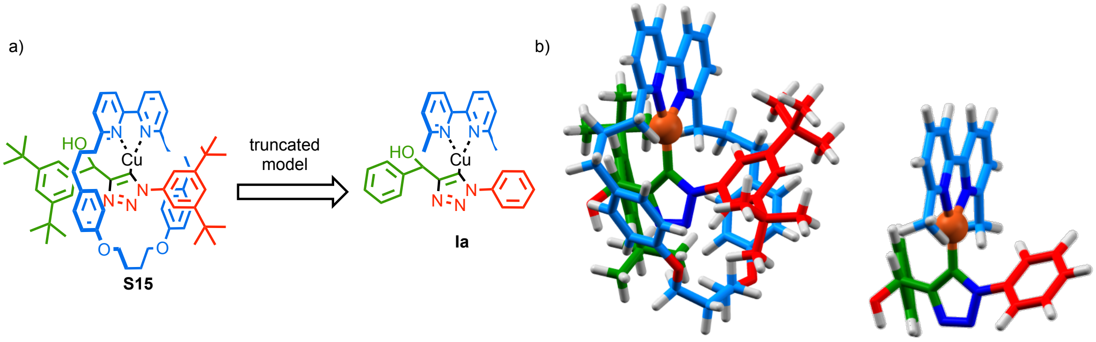

Figure S126 a) Structures and b) models of **S15** and truncated triazolide model **Ia**

ii. DFT evaluation of the pathway of N_2 loss from truncated triazolide model **Ia**

Gaussian '09 (DFT-rB3LYP-631G) was used for subsequent calculations with the default H_2O solvation model.[13] The reaction pathway obtained, computed structures obtained and their relative energies are shown in **Figure S127** and **Figure S128**.

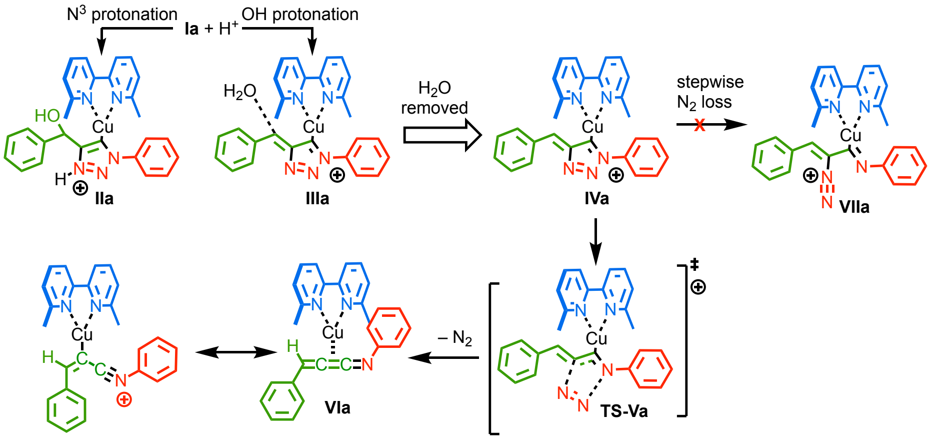

Figure S127 Intermediates in the calculated path from truncated axle **Ia** to cumulene **VI**.

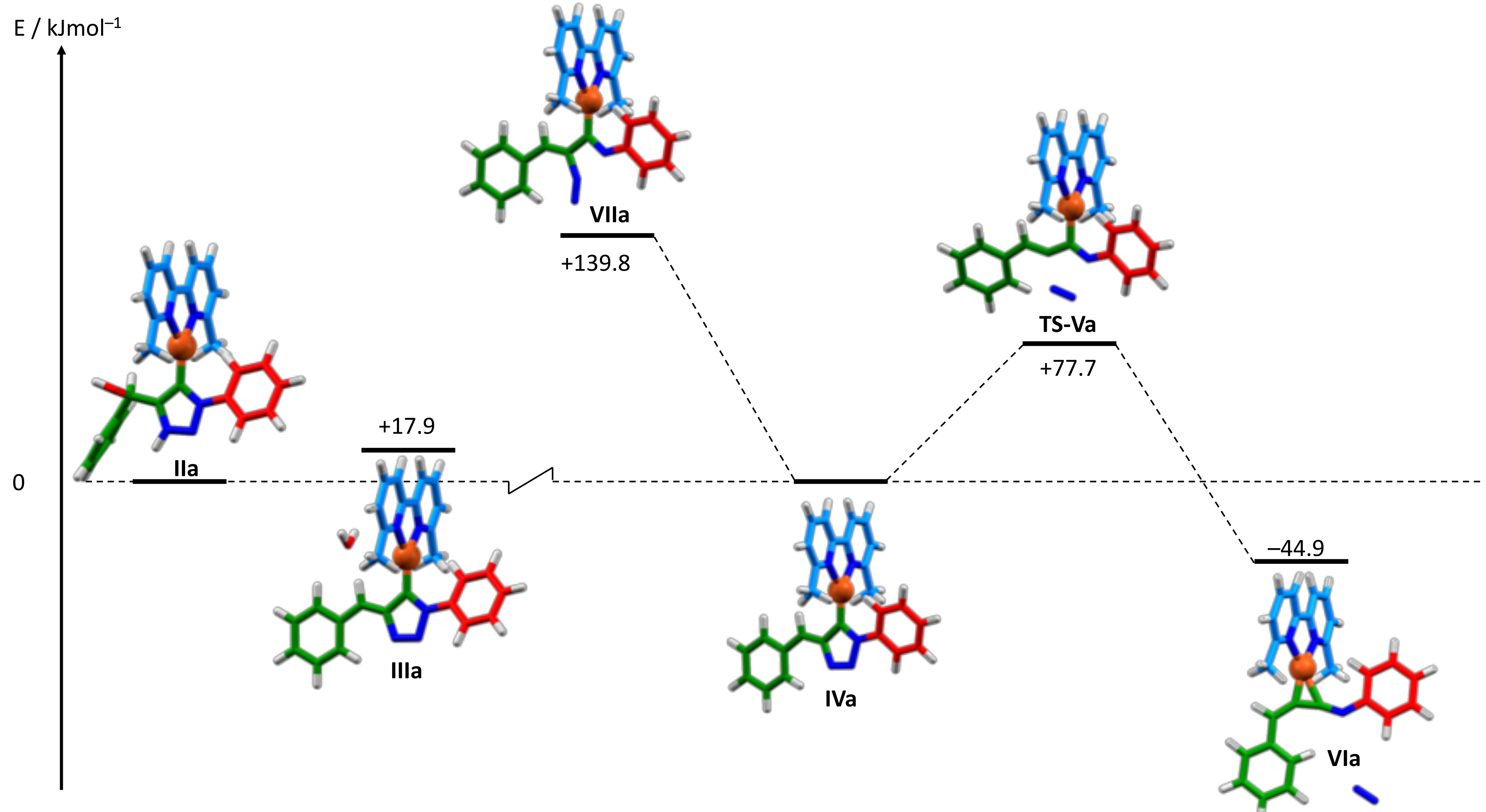

Figure S128 Calculated (Gaussian '09, rB3LYP-631G, default H_2O solvent model) structures and energies of intermediates **IIa** to **VIIa**.

H^+ was added to either N^3 of the triazole or the OH of structure **Ia** and the geometry of these species was optimised. No stable structure could be identified for the O-protonated species in which the C-O bond was maintained. The energy difference between N-protonated (**IIa**) and O-protonated (**IIIa**) models was found to be 17.9 kJmol^{-1}.

The departed H_2O leaving group was removed from the model of the carbocation and the geometry was optimised again (**IVa**). This structure was used as a starting point for further calculations and its computed energy was used as the new baseline.

A scan (unrestricted, 10 steps of 0.1 Å) was performed using the C-N^3 bond length as the redundant coordinate. The outcome of this scan indicated that an energy maximum was reached with C-N^3 = 1.82276 Å (**Figure S129a**). A transition state calculation was performed using the maximum energy structure found in this scan as a starting point. A species (**TS-Va**) with a single imaginary frequency was identified. An IRC calculation (20 steps forward and 20 steps reverse, final structures optimised, **Figure S129b**) confirmed that this transition state connected the starting cation (**IV**) and a cumulene species (**VI**) in which N_2 had been extruded. The reaction is exergonic by 44.9 kJmol^{-1} and **TS-Va** lies 77.7 kJmol^{-1} above cation **Va**.

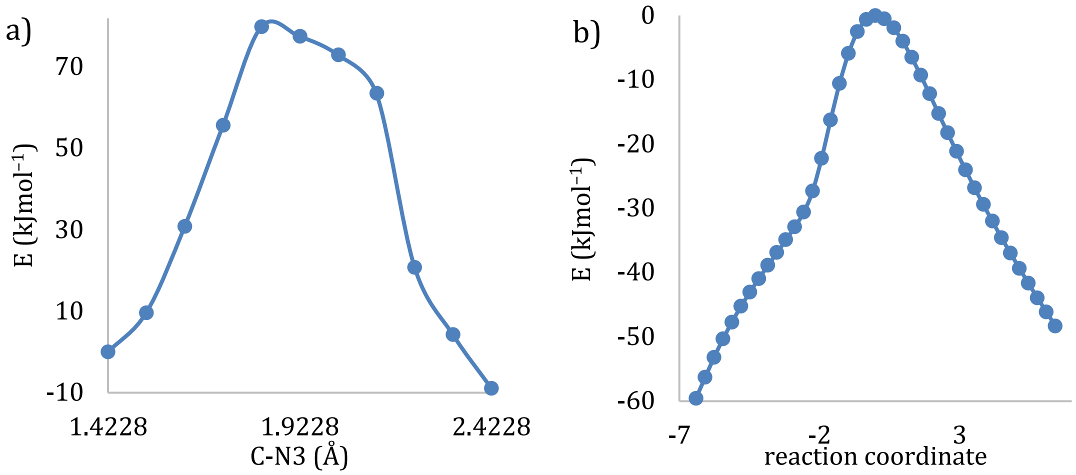

Figure S129 a) Plot of the scan (unrestricted with N^1-N^2 as redundant coordinate) of the N^1-N^2 length in 0.1 Å steps vs energy. b) Plot of the IRC scan for **TS-V**.

Examining the calculated structure of cumulene (**VI**), it is worth noting that a) the Cu^I-ion remains associated with the π-system and b) the C-C-C-N unit is not linear. This may indicate that the true structure lies somewhere between the limiting resonance structures in which the Cu^I-ion engages the cumulene through a π-metal interaction and a σ-metal interaction (**Figure S127Figure S129**).

We were unable to locate a transition state for the stepwise opening of the triazole to give vinyl diazonium **VIIa**, the expected intermediate if N_2 loss were to proceed in a stepwise manner. This is unsurprising as this species was found to lie 139.8 kJmol^{-1} above that of cation **IVa** (i.e. 62.1 kJmol^{-1} above **TS-Va**) when prepared and optimised directly. The IRC plot shows an inflection before **TS-Va** and examining the computed structures, it is clear that whereas early in the process the reaction coordinate is primarily associated with the stretching of the N^1-N^2 bond, as would be expected en route to **VIIa**, the inflection point is associated with the stretching of the C-N^3 bond starting to contribute to the pathway.

iii. DFT evaluation of the pathway of N_2 loss from truncated triazole model **Ib**

Finally, for comparison, we repeated the above calculations for the corresponding reaction of triazole starting material **Ib** (pathway shown in **Figure S130**, calculated structures and energies shown in **Figure S131**). The reaction of **Ib** was found to proceed via a stepwise pathway in which vinyl diazonium **VIIb** is an intermediate lying in a shallow minimum ~1 kJmol^{-1} below the transition state which is itself found 49.1 kJmol^{-1} above **IVb**. The transition state for loss of N_2 from **VIIb** was found to lie ~ 90.9 kJmol^{-1} above this intermediate and thus, including the pre-equilibrium between **IVb** and **VIIb**, the transition state of the rate limiting loss of N_2 lies 139 kJmol^{-1} above cation **IVb**. Furthermore, an IRC calculation suggests that the product of this pathway is best represented by limiting the limiting resonance structures shown rather than a cumulene structure analogous to **VIa** as migration of the C-H bond was not found to take place spontaneously during loss of N_2.

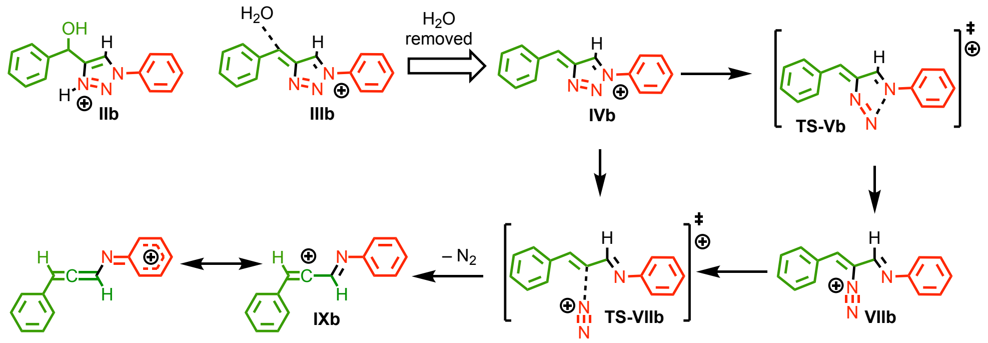

Figure S130 Intermediates in the calculated path from truncated axle **Ia** to cumulene **VI**.

iv. Conclusions

Comparing the computed reaction pathways for loss of N_2 from **Ia** and **Ib**, the electron rich C-Cu^I bond of the triazolide appears favour the opening of the triazole compared with the C-H bond of the simple triazole in three ways: i) the stability of the intermediate formed by protonation of the OH group (**III**) compared to the intermediate protonated on N (**II**) is enhanced by the Cu-C bond, biasing this pre-equilibration step towards key reactive intermediate **III** (**IV**); ii) the pathway of the ring opening process is altered as the electron rich Cu-C bond is eliminated during the loss of N_2, leading directly to a stable cumulene structure instead of a stepwise process via vinyl diazonium **VIIa**; iii) the C-H bond does not participate in N_2 loss. As a consequence, loss of N_2 leads to unstable vinyl cation product **IX** with a consequently higher reaction barrier.

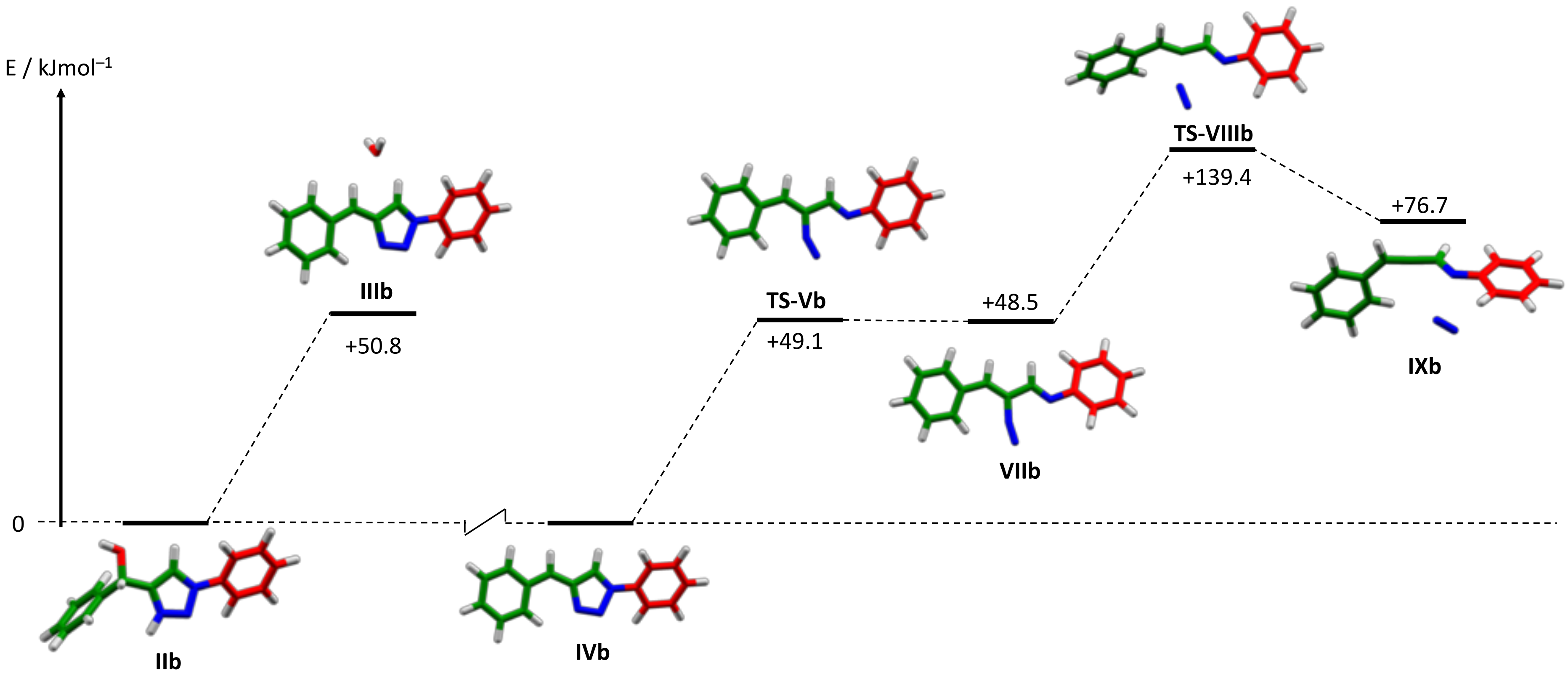

Figure S131 Calculated (Gaussian '09, rB3LYP-631G, default H_2O solvent model) structures and energies of intermediates **IIb** to **IXb**.

12. References

[1] J. Hornung, D. Fankhauser, L. D. Shirtcliff, A. Praetorius, W. B. Schweizer, F. Diederich, *Chem. – A Eur. J.* **2011**, *17*, 12362.

[2] W. Zhu, D. Ma, *Chem. Commun.* **2004**, 888.

[3] J. J. Gassensmith, L. Barr, J. M. Baumes, A. Paek, A. Nguyen, B. D. Smith, *Org. Lett.* **2008**, *10*, 3343.

[4] E. A. Neal, S. M. Goldup, *Angew. Chem. Int. Ed.* **2016**, *55*, 12488.

[5] J. E. M. Lewis, R. J. Bordoli, M. Denis, C. J. Fletcher, M. Galli, E. A. Neal, E. M. Rochette, S. M. Goldup, *Chem. Sci.* **2016**, *7*, 3154.

[6] L. M. Urner, M. Sekita, N. Trapp, W. B. Schweizer, M. Wörle, J.-P. Gisselbrecht, C. Boudon, D. M. Guldi, F. Diederich, *European J. Org. Chem.* **2015**, *2015*, 91.

[7] Y. Itoh, R. Kitaguchi, M. Ishikawa, M. Naito, Y. Hashimoto, *Bioorg. Med. Chem.* **2011**, *19*, 6768.

[8] L. Palatinus, G. Chapuis, *J. Appl. Crystallogr.* **2007**, *40*, 786.

[9] L. Palatinus, S. J. Prathapa, S. Van Smaalen, *J. Appl. Crystallogr.* **2012**, *45*, 575.

[10] G. M. Sheldrick, *Acta Crystallogr. Sect. C Struct. Chem.* **2015**, *71*, 3.

[11] G. M. Sheldrick, *Acta Crystallogr. Sect. A Found. Crystallogr.* **2008**, *64*, 112.

[12] R. K. Sharma, J. L. Fry, *J. Org. Chem.* **1983**, *48*, 2112.

[13] M. J. Frisch, G. W. Trucks, H. B. Schlegel, G. E. Scuseria, M. A. Robb, J. R. Cheeseman, G. Scalmani, V. Barone, B. Mennucci, G. A. Petersson, H. Nakatsuji, M. Caricato, X. Li, H. P. Hratchian, A. F. Izmaylov, J. Bloino, G. Zheng, J. L. Sonnenberg, M. Hada, M. Ehara, K. Toyota, R. Fukuda, J. Hasegawa, M. Ishida, T. Nakajima, Y. Honda, O. Kitao, H. Nakai, T. Vreven, J. A. Montgomery, Jr., J. E. Peralta, F. Ogliaro, M. Bearpark, J. J. Heyd, E. Brothers, K. N. Kudin, V. N. Staroverov, R. Kobayashi, J. Normand, K. Raghavachari, A. Rendell, J. C. Burant, S. S. Iyengar, J. Tomasi, M. Cossi, N. Rega, J. M. Millam, M. Klene, J. E. Knox, J. B. Cross, V. Bakken, C. Adamo, J. Jaramillo, R. Gomperts, R. E. Stratmann, O. Yazyev, A. J. Austin, R. Cammi, C. Pomelli, J. W. Ochterski, R. L. Martin, K. Morokuma, V. G. Zakrzewski, G. A. Voth, P. Salvador, J. J. Dannenberg, S. Dapprich, A. D. Daniels, Ö. Farkas, J. B. Foresman, J. V. Ortiz, J. Cioslowski, and D. J. Fox, *Gaussian 09* (Gaussian, Inc., Wallingford CT, 2009).
